# Synthesis of Fluorinated Glycotope Mimetics Derived from *Streptococcus pneumoniae* Serotype 8 CPS

**DOI:** 10.3390/ijms26041535

**Published:** 2025-02-12

**Authors:** Daniel Gast, Sebastian Neidig, Maximilian Reindl, Anja Hoffmann-Röder

**Affiliations:** Department of Chemistry, Ludwig-Maximilians-Universität München, Butenandtstrasse 5-13, Haus F, 81377 Munich, Germany

**Keywords:** glycans, glycoconjugates, fluorinated carbohydrates, glycotope mimetics, *Streptococcus pneumoniae* serotype 8, synthesis, late-stage fluorination, CPS fragments

## Abstract

Fluorination of carbohydrates is a promising strategy to produce glycomimetics with improved pharmacological properties, such as increased metabolic stability, bioavailability and protein-binding affinity. Fluoroglycans are not only of interest as inhibitors and chemical probes but are increasingly being used to develop potential synthetic vaccine candidates for cancer, HIV and bacterial infections. Despite their attractiveness, the synthesis of fluorinated oligosaccharides is still challenging, emphasizing the need for efficient protocols that allow for the site-specific incorporation of fluorine atoms (especially at late stages of the synthesis). This is particularly true for the development of fully synthetic vaccine candidates, whose (modified) carbohydrate antigen structures (glycotopes) per se comprise multistep synthesis routes. Based on a known minimal protective epitope from the capsular polysaccharide of *S. pneumoniae* serotype 8, a panel of six novel F-glycotope mimetics was synthesized, equipped with amine linkers for subsequent conjugation to immunogens. Next to the stepwise assembly via fluorinated building blocks, the corresponding 6F-substituted derivatives could be obtained by microwave-assisted, nucleophilic late-stage fluorination of tri- and tetrasaccharidic precursors in high yields. The described synthetic strategy allowed for preparation of the targeted fluorinated oligosaccharides in sufficient quantities for future immunological studies.

## 1. Introduction

Since the first vaccination attempts by Edward Jenner and Louis Pasteur more than 200 years ago, strategies have been established to effectively support the immune system by active immunization to build protective immune responses against pathogens [1]. In the case of bacterial pathogens, conserved glycan structures of capsular polysaccharides (CPSs) can be used as target structures for recognition by the immune system [2,3]. The CPS completely covers the bacterial cell wall as a protective layer and its specific composition is crucial for the pathogen’s virulence. Moreover, as CPS fragments are highly exposed on the cell surface, they can serve as the first line of recognition for the immune system to enable the formation of specific antibodies against these glycan structures [4,5]. To ultimately protect against the disease caused by the pathogen, these immune responses must be further enhanced, which usually requires covalent binding of the respective pathogen-associated glycan structure to suitable immunogens [6,7,8]. This concept of glycoconjugate vaccines has been subject of intensive research since the 1980s and already led to global breakthroughs in protection against life-threatening infections caused by e.g., *Streptococcus pneumoniae*, *Neisseria meningitidis* and *Haemophilus influenzae* [9,10,11]. However, given the existing resistances to antibiotics and the persistent problems caused by insufficient immunizations of risk groups such as infants and the elderly, there is still a high demand for new and/or improved vaccine candidates [12,13].

Most of the currently available glycoconjugate vaccines used in clinical applications contain polysaccharide structures from natural sources and are therefore inherently heterogeneous [14]. Not only does this mean variability in structure from batch to batch, but it can also be associated with varying degrees of purity, necessitating stringent analytical quality control. To overcome these drawbacks, synthetic production of immunologically active carbohydrate epitopes (so-called glycotopes) has been increasingly pursued [15,16,17]. Since assembly of these epitopes proceeds in a stepwise manner, the corresponding structures can be precisely determined and verified at any time by analytical means, so that manufacturing without contamination is guaranteed [18,19,20,21]. To minimize synthetic efforts, minimal protective epitope structures are preferably used, which can be identified using microarray technology [13,22,23]. An additional advantage of this synthetic approach is the possibility to introduce chemical modifications in a site-specific manner, to make the epitope more immunogenic and to improve its bioavailability [15,24,25]. One particularly promising strategy involves the selective modification of carbohydrate epitopes with fluorine substituents [26,27,28,29,30,31]. As has been impressively demonstrated in several vaccine examples, the use of fluorine substituents can not only improve the enzymatic stability of the carbohydrate fragment but also enhances its “non-self” character and thus increases its antigenicity [32,33,34,35,36,37,38,39,40,41,42,43,44].

*Streptococcus pneumoniae* (SP) is a Gram-positive bacterium commonly found to colonize the upper respiratory tract in humans [45]. It is the cause of invasive diseases such as pneumonia, sepsis, meningitis and otitis media in newborns or infants, and these infections are still a major health risk for infants, immunocompromised patients and the elderly, making worldwide access to effective vaccines against *S. pneumoniae* particularly important [46,47]. SP bacteria can be classified into more than 97 serotypes based on capsular polysaccharides, and SP serotype 8 (ST8) is highly virulent, repeatedly associated with high antibiotic resistance and outbreaks of invasive pneumococcal disease [48,49]. The absence of ST8 in commercial pneumococcal glycoconjugate vaccines (e.g., Prevnar 13) highlights the need for the development of effective preventive therapies against this pathogen [50,51,52,53].

**Figure 1:**
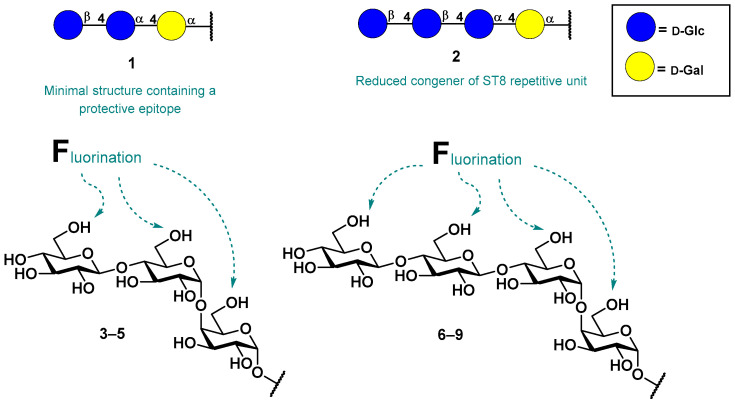
Depiction of previously reported ST8 glycotope **1** and **2**. Targeted collection of fluorinated tri- and tetrasaccharidic glycotope mimetics **3**–**9**.

In 2017, Schumann et al. conducted a comprehensive study in which they identified trisaccharide β-D-Glc-(1,4)-α-D-Glc-(1,4)-α-D-Gal (**1**) as the minimal, protective epitope of ST 8 (Figure 1) [54]. While the low immunogenicity of this glycotope hampered its use in semi-synthetic glycoconjugate vaccines, a corresponding vaccine candidate with a “reduced derivative of the repetitive ST8 unit”, i.e., containing the glycan structure β-D-Glc-(1,4)-β-D-Glc-(1,4)-α-D-Glc-(1,4)-α-D-Gal (**2**) carrying a terminal glucose moiety instead of a glucuronic acid, led to promising immune responses in a rabbit model [54]. In continuation of our previous work on fluorinated antigen structures [32,33,36,55,56,57,58,59], a series of new fluorinated glycotope mimetics is herein reported based on the minimal (protective) epitopes **1** and **2** identified by Schumann et al. [54] (Figure 1).

**Figure 2:**
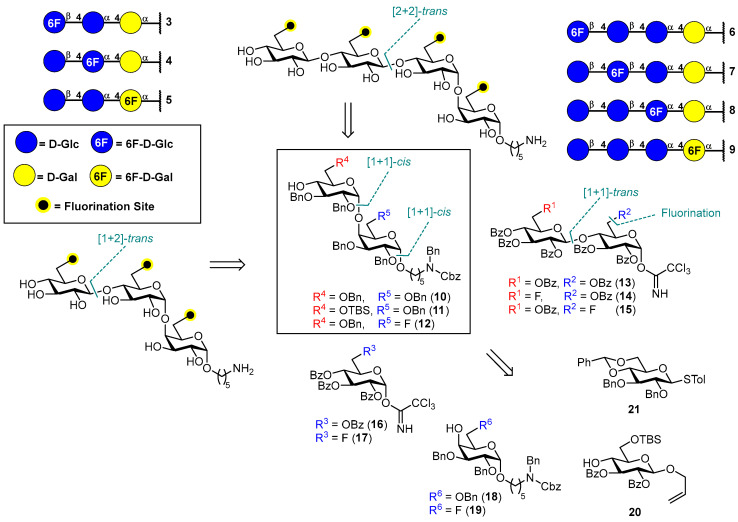
Retrosynthetic analysis and key building blocks for the assembly of fluorinated glycotopes **3**–**9**.

The assumption that the C6 positions of the saccharide units are certainly the most exposed positions of these glycotopes for recognition by immune cells prompted us to synthesize glycan mimetics containing such C6-fluorinated glycan units in the saccharide motif [35]. Given that fluorine incorporation, in general, can affect immune recognition of an epitope, we systematically prepared a series of previously unknown tri- and tetrasaccharide mimetics, each of which contained a C6 monofluorinated glycan unit and featured a linker at the reducing end for subsequent conjugation steps (Figure 2). The resulting F-glycotope mimetics **3**–**9** will be useful tools for the systematic investigation of the influence of F-substitutions on antibody recognition as well as for the design of synthetic vaccines upon further attachment to immunogens.

## 2. Results and Discussion

### 2.1. Retrosynthetic Analysis

Fluorinated trisaccharide fragments **3**–**5** as well as the corresponding tetrasaccharide fragments **6**–**9** should be assembled analogously to previous synthetic approaches from the functionalized disaccharide precursors **10**–**15** using a [1 + 2] or [2 + 2] strategy (Figure 2) [54,60]. In this way, all CPS fragments can be traced back to a central D-Glc-(1→4)-α-D-Gal acceptor unit with either orthogonal ether protecting groups at C6’ and C6 (a benzylic ether vs. a silyl ether in compounds **10** and **11**) or a benzylic ether and a fluorine substituent in these positions as in **12**. The use of a participating protecting group at C2 of the respective donor units should allow subsequent 1,2-*trans* glycosylation reactions with these disaccharide accepting units to proceed in a stereochemically controlled way. Hence, building blocks **10**–**12** could be used together with donors **16** [61] and **17** in a Schmidt glycosylation reaction [62] to produce trisaccharides **3**–**5**, while tetrasaccharides **6**–**9** would necessitate acceptors **10** and **12** as well as cellobiosyl building blocks **13**–**15**. The latter can either be obtained directly from commercially available native D-cellobiose or can be synthesized from suitable monosaccharide building blocks **16**, **17** and **20** by 1,2-*trans*-selective Schmidt glycosylation reactions. Additionally, a protecting group pattern should be used, which allows selective deprotection at specific C6 positions at a later stage of the synthesis, for example, by using orthogonal (silyl) ethers together with benzylidene acetals and benzoyl groups.

The most challenging step, however, is the formation of the α-D-Glc-(1→4)-α-D-Gal fragment in building blocks **10**–**12**, as the two 1,2-*cis*-glycosidic bonds cannot be controlled by neighboring group effects. Therefore, 4,6-benzylidene acetal protected glycosyl donors were used, as these favor 1,2-*cis* linkages [63] and allow the selective release of the C4 position for subsequent glycosylation steps. Thus, thioglucoside **21** [64] and allyl glucoside **20** can be identified as common precursor moieties in addition to the known 5-amino pentanol linker structure [65]. In summary, the six monosaccharide units **16**–**21** shown in Figure 2 are used as key building blocks in the following syntheses.

### 2.2. Synthesis of Galactosyl Acceptors ***18*** and ***19***

**Scheme 1:**
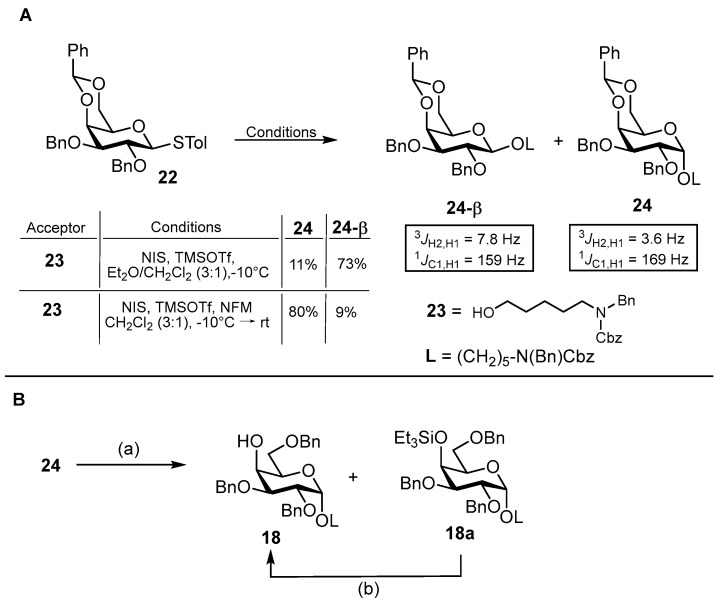
Synthesis of galactosyl acceptor **18**. (**A**) Reagents and conditions for linker glycosylation. ^3^*J*_H2,H1_ and ^1^*J*_H1,C1_ are the coupling constants of glycosylation products **24** and **24-β**. (**B**) Regioselective benzylidene acetal opening of **24**. Reagents and conditions: (a) NIS, Et_3_SiH, TfOH, CH_2_Cl_2_, −78 °C, 89% (**18** + **18a**); (b) 18a, CSA, MeOH, CH_2_Cl_2_, rt, 84% (overall yield).

In line with the strategic considerations above, galactosyl acceptor **18** was assembled from the known galactosyl derivative **22** [66] in two steps (Scheme 1A). Although it is well known that stereoselective 1,2-*cis* glycosylation reactions using highly reactive primary acceptors such as amino alcohols are challenging because their selectivity is prone to erode [67], the addition of co-solvents with the ability to induce α-stereoselectivity (e.g., THF, Et_2_O or dioxane) has often been found to be beneficial [68]. However, initial glycosylation experiments with 5-amino pentanol linker **23** using an ether-modulated approach predominantly gave rise to the formation of undesired 1,2-*trans* configured product **24-β** (73%, Scheme 1A, SI Figure S1). To further promote the formation of the desired 1,2*-cis* glycosidic bond, exogenous nucleophiles were to be employed. A recently published protocol was followed, in which *N*-formylmorpholine (NFM) is used as an α-directing modulator [69]. To our delight, the reaction of thioglycoside **22** with linker **23** in the presence of NFM, the promoter system *N*-iodosuccinimide (NIS) and trimethylsilyl triflate (TMSOTf) provided the desired α-glycoside **24** as the major product (80% yield) together with only minor amounts of **24-β** (9% isolated yield). Both anomers were conveniently separated by standard column chromatography and the desired α-anomeric configuration of **24** was confirmed by NMR spectroscopy.

The observed scalar coupling constant ^3^*J*_H2,H1_ = 3.6 Hz can be related to a dihedral angle of approximately 60°, which corresponds to an anomeric α-linkage in **24** [70]. Additional support was provided by ^1^H-^13^C-coupled heteronuclear single quantum correlation (HSQC) experiments showing ^1^*J*_H1,C1_ coupling constants of 169 Hz for **24** and 159 Hz (^3^*J*_H2,H1_ = 7.8 Hz) for **24-β**. After the α-glycosidic bond had been successfully installed at the anomeric center of **24**, subsequent regioselective benzylidene opening reaction with triethyl silane (Et_3_SiH) and triflic acid (TfOH) in CH_2_Cl_2_ [71] ultimately provided an acceptor **18**. In some cases, significant amounts of a 4-*O*SiEt_3_ by-product **18a** were obtained, but these could be easily converted into **18** by treatment with camphor sulfonic acid (CSA) in a solvent mixture of MeOH and CH_2_Cl_2_. With this additional step, the desired product was obtained in a total yield of 84% (Scheme 1B, SI). Interestingly, the formation of the by-product **18a** was observed only upon scaling-up and neutralization with triethylamine.

**Scheme 2:**
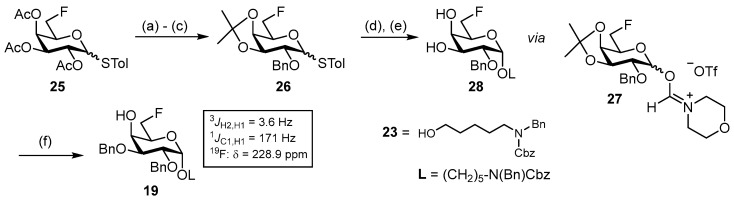
Synthesis of the fluorinated galactosyl acceptor **19**. Reagents and conditions. (a) NaOMe, MeOH, rt, quant.; (b) 2,2-dimethoxypropane, *p*-TsOH, acetone, rt; (c) NaH, BnBr, DMF, 0 °C → rt, 75% over three steps; (d) NIS, TMSOTf, NFM, CH_2_Cl_2_, −10 °C, then **23**, −10 °C → rt; (e) 80% aq. AcOH, 80 °C, 42% over two steps; (f) DBTO, toluene, 85 °C, then TBAB, BnBr, 100 °C, 86%.

A fluorinated galactosyl acceptor **19** was synthesized in 11 steps from commercially available D-galactose (Scheme 2). Hence, by employing known precursor **25** [59] in a series of standard protecting group manipulations (Zemplén transesterification, 3,4-*O*-isopropylidenylation and C2 etherification), the orthogonally protected thioglycoside **26** was obtained in 75% yield over three steps (SI Figure S2). This compound was then subjected to the NFM-modulated linker glycosylation step using **23** as described above. However, the reaction was, this time, significantly slower than the corresponding NFM-modulated glycosylation of donor **22**. While thin layer chromatography (TLC) monitoring indicated smooth formation of the intermediate imidinium species **27** at −10 °C, subsequent attack by the nucleophile **23** was impaired and substantial amounts of **27** remained detectable even after two days at ambient temperature. Nevertheless, hydrolysis and chromatographic purification furnished the desired α-linked product, which could then be converted into diol **28** by acidic cleavage of the isopropylidene acetal group.

Ultimately, the desired 6F-galactosyl acceptor **19** was obtained using a regioselective 3-OBn etherification protocol with the help of dibutyltin oxide (DBTO) and tetrabutylammonium bromide (TBAB) [72]. Again, the required anomeric α-linkage of compound **19** was verified by its coupling constants of ^3^*J*_H2,H1_ = 3.6 Hz and ^1^*J*_H1,C1_ = 171 Hz.

### 2.3. Synthesis of Disaccharide Acceptors ***10***, ***11*** and ***12***

**Scheme 3:**
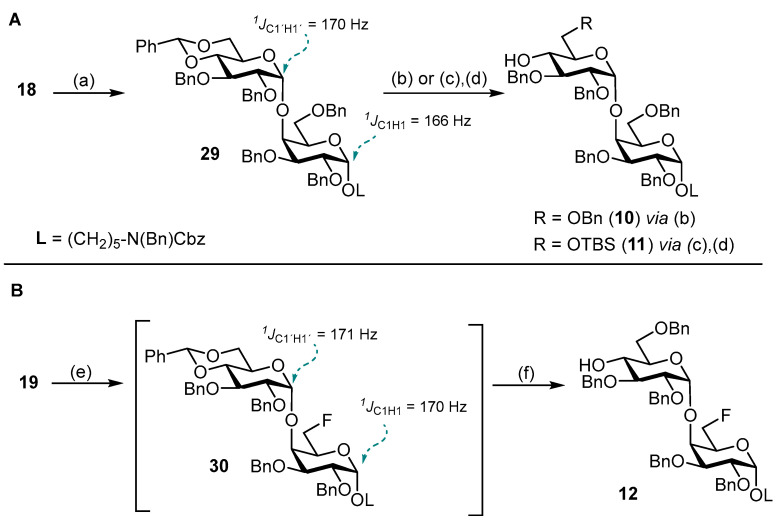
(**A**) Synthesis of disaccharide acceptors **10** and **11**. Reagents and conditions. (a) **21**, TMSOTf, NIS, CH_2_Cl_2_, −30 °C, 93%; (b) BH_3_·NMe_3_, BF_3_·OEt_2_, CH_2_Cl_2_, MeCN, −10 °C, 80%; (c) EtSH, *p*-TsOH, CH_2_Cl_2_, rt; (d) TBSCl, imidazole, DMF, rt, 83% over two steps. (**B**) Synthesis of fluorinated disaccharide acceptor **12**. Reagents and conditions. (e) **21**, TMSOTf, NIS, CH_2_Cl_2_, −30 °C → 0 °C; (f) BH_3_·NMe_3_, BF_3_·OEt_2_, CH_2_Cl_2_, MeCN, −10 °C, 62% over two steps.

Taking advantage of the inherent 1,2*-cis* directing ability of 4,6-*O*-benzylidene acetal protective groups in glucosidation reactions [73], the thioglucoside donor **21** was coupled α-stereoselectively with a yield of 93% to the galactosyl acceptor **18** in the presence of NIS and TMSOTf (Scheme 3A, SI Figure S3). Spectroscopic proof of the desired 1,2*-cis* linkage of **29** was obtained by ^1^H-^13^C coupled HSQC and heteronuclear multiple bond correlation (HMBC) experiments revealing the requisite coupling constants of ^3^*J*_H1′,H2′_ = 3.5 Hz, ^1^*J*_C1,H1_ = 166 Hz and ^1^*J*_C1′,H1′_ = 170 Hz. The disaccharide **29** was then converted into the 6-OBn acceptor **10** by regioselective opening of the benzylidene acetal protective group using a borane–trimethylamine complex (BH_3_·NMe_3_) in the presence of BF_3_·OEt_2_ [74]. The regioselectivity of this step was once more demonstrated by NMR spectroscopy, showing a clear cross-peak between C6 (δ = 69.1 ppm) and the CH_2_ group of the benzyl ether (δ = 4.38/4.20 ppm) in the HMBC spectrum. Furthermore, the anomeric configurations were reconfirmed on the basis of the ^1^*J*_C1,H1_ = 169 Hz and ^1^*J*_C1′,H1′_ = 169 Hz coupling constants.

An acceptor building block **11**, in contrast, was obtained from disaccharide **29** after acid-mediated cleavage of the benzylidene acetal protective group and subsequent regioselective blocking of the primary 6′-OH group with *t*-butyldimethylsilyl chloride (TBSCl) in a yield of 83% over two steps (Scheme 3A, SI Figure S3).

The corresponding fluorinated disaccharide acceptor **12** resulted from a similar sequence of NIS/TMSOTf-mediated glucosidation to the compound **30**, followed by regioselective opening of the benzylidene acetate protective group (Scheme 3B, SI Figure S3). Although the glycosylation reaction of the compound **19** with the thioglucoside donor **21** went smoothly, the desired product **30** could not be isolated in pure form. Instead, about 10% of a contaminant were detected in the NMR spectrum, which probably resulted from the degradation of the glycoside donor **21** and could not be separated by column chromatography. Likewise, the use of a corresponding PTFA glycosyl donor did not lead to any further improvement, and thus only a moderate yield of 62% of the fluorinated acceptor **12** was finally obtained after opening the benzylidene acetal protective group with BH_3_·NMe_3_ and BF_3_·OEt_2_.

### 2.4. Synthesis of Fluorinated Cellobiosyl Donors ***14*** and ***15***

**Scheme 4:**
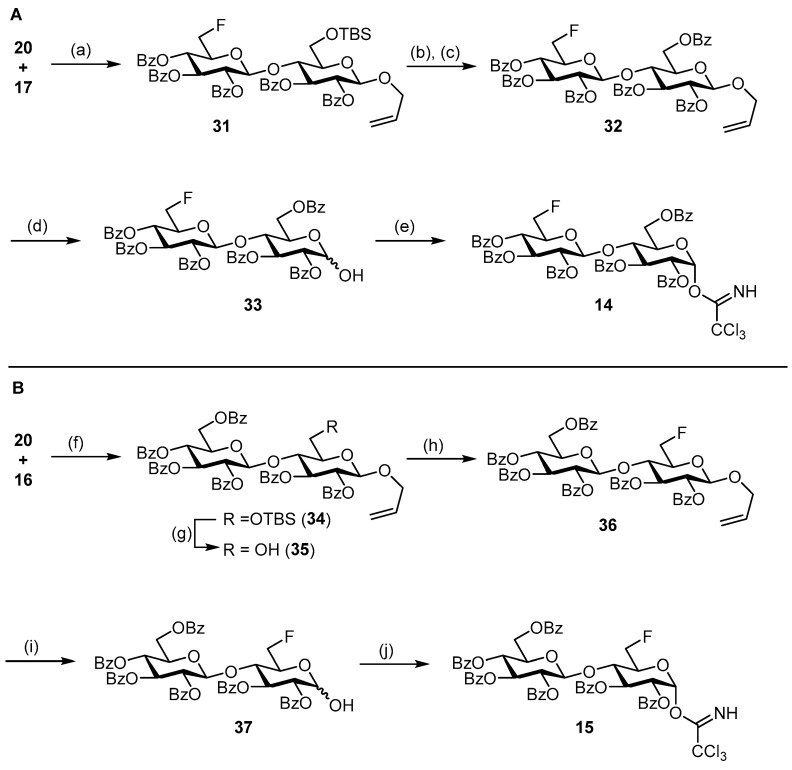
(**A**) Synthesis of fluorinated cellobiosyl donor **14**. Reagents and conditions. (a) TMSOTf, CH_2_Cl_2_, 0 °C → rt, 74%; (b) *p*-TsOH, MeOH, CH_2_Cl_2_, 50 °C; (c) BzCl, pyridine, 50 °C, 70% over two steps; (d) PdCl_2_, MeOH/THF 60 °C, 97%; (e) TCA, DBU, CH_2_Cl_2_, 0 °C → rt, 87%. (**B**) Synthesis of fluorinated cellobiosyl donor **15**. Reagents and conditions. (f) TMSOTf, CH_2_Cl_2_, 0 °C → rt, 95%; (g) 80% AcOH, MeCN, 60 °C, 90%; (h) DAST, 2,4,6-collidine, CH_2_Cl_2_, 80 °C, 100 W, 93%; (i) PdCl_2_, MeOH/THF 60 °C, 98%; (j) TCA, DBU, CH_2_Cl_2_, 0 °C → rt, 89%.

In addition to the perbenzoylated cellobiosyl trichloroacetimidate donor **13**, which can be readily obtained from native D-cellobiose by a sequence of standard protecting group transformations (SI Figure S4), two new fluorinated congeners **14** and **15** were prepared (Scheme 4). The synthesis of the 6’F-cellobiose precursor **14** started with the formation of the β-D-Glc-(1→4)-β-D-Glc glycosidic bond using the glucosyl acceptor **20** and the fluorinated trichloroacetimidate donor **17** (SI, Figure S5) under Schmidt conditions (Scheme 4A, SI). Initial results indicated that the choice of the protective group at C6 of the acceptor unit is critical for this reaction, as the use of a benzyl ether in this position always led to inseparable product mixtures. Fortunately, the reaction of the 6-OTBS-protected glucosyl acceptor **20** with the fluorinated glucosyl donor **17**, both of which are accessible from commercially available 1,2,3,4,6-Penta-*O*-acetylglucose via known allyl glucoside **S3** [75] (SI Figure S5), yielded the desired β-configured disaccharide **31** in 74% (Scheme 4A, SI Figure S6). The requisite β-anomeric linkage was confirmed by NMR (^3^*J*_H2′,H1′_ = 8.0 Hz) and subsequent acid-mediated desilylation followed by esterification yielded benzoyl ester **32** in 70% over two steps. Conversion to the final 6′F-cellobiosyl trichloroacetimidate donor **14** was accomplished by anomeric deallylation to **33**, using PdCl_2_ in MeOH/THF [76] and subsequent 1,8-diazabicyclo[5.4.0]undec-7-ene (DBU)-mediated *O*-alkylation with trichloroacetonitrile (TCA) providing **14** in a yield of 84% over two steps.

The synthesis of the cellobiosyl donor **15** likewise began with the formation of the β-1→4-*O*-glycosidic bond, yielding **34** in an isolated yield of 95% and as the β-anomer (^3^*J*_H1′,H2′_ = 8.0 Hz, Scheme 4B, SI Figure S7). Subsequent deprotection of the silyl group under acidic conditions gave free alcohol **35**, which was subjected to a microwave-assisted fluorination protocol with diethylaminosulfur trifluoride (DAST) [55,56] to obtain the desired F-building block **36** in a very good yield. Again, regioselectivity of the fluorination reaction was confirmed on the basis of the ^1^*J*_F,C6_ = 174 Hz coupling constant and the ^19^F signal at δ = −230 ppm for **36**, both of which are in good agreement with previously reported data for C6 fluorinated glucose derivatives [77]. A two-step protocol of Pd-catalyzed anomeric deallylation to **37** and base-promoted *O*-alkylation with TCA as in the previous procedure ultimately yielded the desired 6F-trichloroacetimidate donor **15**.

### 2.5. Synthesis of Fluorinated Trisaccharides ***3***, ***4*** and ***5***

**Scheme 5:**
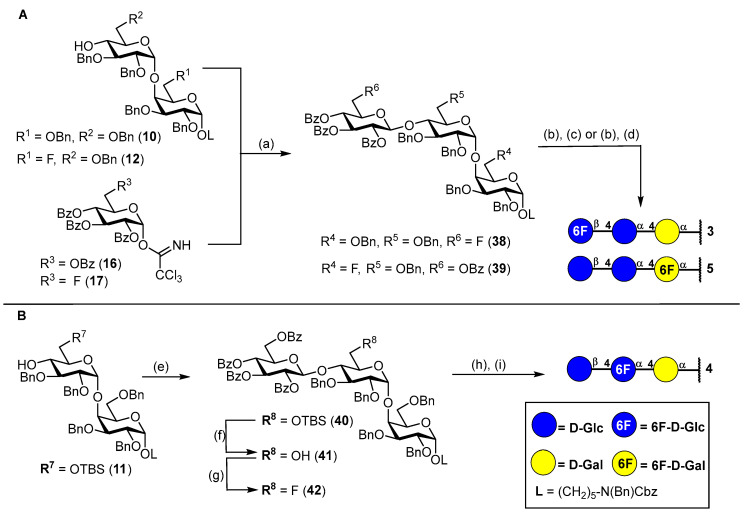
(**A**) Synthesis of fluorinated trisaccharides **3** and **5**. Reagents and conditions. (a) TMSOTf, CH_2_Cl_2_, 0 °C, 77% (**38**), 87% (**39**); (b) NaOMe, MeOH/THF, rt; (c) H_2_, Pd(OH)_2_/C, CH_2_Cl_2_, *t*-BuOH, H_2_O, rt, 89% over two steps (**3**); (d) H_2_, Pd/C, AcOH, THF, MeOH, H_2_O, rt, 92% over two steps (**5**). (**B**) Synthesis of trisaccharidic precursors **40**–**41** and the targeted fluorinated trisaccharide **4**. Reagents and conditions. (e) **16**, TMSOTf, CH_2_Cl_2_, 0 °C, 93%; (f) *p*-TsOH, MeOH, CH_2_Cl_2_, 50 °C, 89%; (g) DAST, 2,4,6-collidine, CH_2_Cl_2_, 80 °C, 100 W, 75%; (h) NaOMe, MeOH/THF, rt; (i) H_2_, Pd/C, AcOH, THF, MeOH, H_2_O, rt, 90% over two steps.

The targeted fluorinated trisaccharides were synthesized by reacting at first the two disaccharide acceptors **10** and **12** with the corresponding glucosyl donors **17** and **16**, respectively, in a TMSOTf-mediated Schmidt glycosylation reaction (Scheme 5A). The anomeric β-stereoselectivity of the newly formed glycosidic bonds in **38** (^1^*J*_C1,H1_ = 165 Hz, β-Glc) and **39** (^1^*J*_C1,H1_ = 166 Hz, β-Glc) was fully controlled by the participating benzoyl protective group at C2 of the donors **16** and **17**. Subsequent global deprotections were performed by a two-step protocol based on literature-known procedures. Hence, cleavage of the benzoyl esters using NaOMe in MeOH/THF was conducted prior to Pd-catalyzed hydrogenolysis [54,78] of the benzyl ether protective groups furnishing the two F-containing trisaccharides **3** and **5** in good yields.

Analogously, the trisaccharide **4**, which contains a fluorine substituent at the C6 of the bridging α-glucose unit, was built up by β-selective coupling of the remaining non-fluorinated building block **11** with the donor **16**, yielding **40** in 93% yield (Scheme 5B). In this case, however, the desired F-substituent was incorporated at a late stage of the synthesis. There exist only a few reports of such late nucleophilic fluorinations, which were mainly restricted to disaccharide motifs, and to the best of our knowledge, microwave-assisted DAST-fluorination protocols have not yet been applied to higher oligosaccharides.

Thus, the silyl ether of the compound **40** was cleaved under acidic conditions and the resulting alcohol **41** was subjected to a deoxyfluorination reaction using DAST under microwave irradiation. After column chromatography, the desired trisaccharide **42** was isolated in a good yield of 75% with full structural integrity. Thorough analysis of the NMR spectra confirmed the correct stereochemical and regioselective linkages of all the trisaccharide building blocks in **42** (^1^*J*_C1,H1_ = 172 Hz, α-Gal; ^1^*J*_C1′,H1′_ = 171 Hz, β-Glc ^1^*J*_C1″,H1″_ = 164 Hz, β-Glc). In addition, global deprotection was again achieved by a similar two-step protocol, ultimately leading to the successful formation of the target compound **4**.

### 2.6. Synthesis of Fluorinated Tetrasaccharides ***6***–***9***

**Scheme 6:**
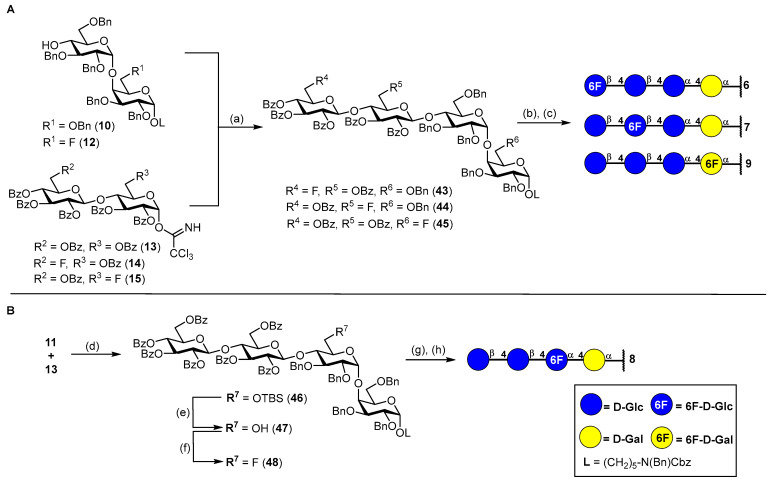
(**A**) Synthesis of fluorinated tetrasaccharides **6**, **7** and **9**. Reagents and conditions. (a) TMSOTf, MS 4 Å, CH_2_Cl_2_, 0 °C, 87% (**43**), 0 °C → rt, 83% (**44**), −20 °C → rt, 84% (**45**); (b) NaOMe, MeOH, rt; (c) H_2_, Pd/C, MeOH, THF, H_2_O, AcOH, rt, 91% over two steps (**6**), 94% over two steps (**7**), 85% over two steps (**9**). (**B**) Synthesis of tetrasaccharidic precursors **46**–**48** and conversion to the targeted fluorinated tetrasaccharide **8**. Reagents and conditions. (d) TMSOTf, MS 4Å, CH_2_Cl_2_, −20 °C → rt; (e) *p*-TsOH, MeOH, CH_2_Cl_2_, 50 °C, 65% over two steps (**47**); (f) DAST, 2,4,6-collidine, CH_2_Cl_2_, 80 °C, 100 W, 75%; (g) NaOMe, MeOH, rt; (h) H_2_, Pd/C, MeOH, THF, H_2_O, AcOH, rt, 93% over two steps.

The fluorinated tetrasaccharide fragments **6**–**9** were assembled in the same way as the trisaccharide congeners, using either fluorinated cellobiosyl donors **14** and **15**, respectively, or a corresponding native derivative **13** [79] in combinations with disaccharide acceptors **10**–**12** (Scheme 6). The devised Schmidt glycosylations to the fully protected fluorinated tetrasaccharides **43**–**45** proceeded again smoothly, β-stereoselectively and with high yields. However, in contrast to the non-fluorinated donor **13**, which was successfully reacted at temperatures of −20 °C to obtain **43**, the two (less reactive) fluorinated cellobiosyl donors **14** and **15** required reaction temperatures of 0 °C and above to generate tetrasaccharides **44** and **45** (Scheme 6A). Moreover, compound **48**, which features the 6F substituent on the reducing Gal unit, was again obtained by a late fluorination step. For this purpose, the non-fluorinated tetrasaccharide **46** was first assembled from donor **13** and acceptor **11** before it was subjected to desilylation at 6’OH under acidic conditions to give **47** (65% over two steps, Scheme 6B). Subsequent microwave-assisted DAST-mediated deoxyfluorination provided the desired F-derivative **48** in an isolated yield of 75%. NMR analysis showed that in all cases (**43**–**46**) only the desired β-anomeric products were formed, due to participation of the 2-OBz ester protective groups of the donor compounds.

Finally, global deprotection was accomplished via the two-step procedure described above, yielding the desired fluorinated tetrasaccharides **6**–**9** in high yields after reverse-phase flash column chromatography. The targeted fluorinated tri- and tetrasaccharides **3**–**9** correspond to the minimal protective epitope-containing ST8 glycan structure and might thus be useful glycotope mimetics for immunological studies. In addition to the expected improved chemical and metabolic stability due to fluorination, increased antigenicity of the glycotopes **3**–**9** and the formation of cross-reactive antibodies after their conjugation to immunogens could be assumed. Similar effects have been observed in the past with carbohydrate cancer vaccines and, if confirmed, should advance the general design and production of synthetic pneumococcal vaccines. The necessary attachment of the modified epitopes to immunogens and preliminary vaccination studies are planned and will be published elsewhere.

## 3. Materials and Methods

If not otherwise noted, all reactions were magnetically stirred and conducted in oven-dried glassware. Moisture sensitive reactions were performed in an argon atmosphere, using standard Schlenk techniques. Solvents for moisture sensitive reactions (diethyl ether, dichloromethane and tetrahydrofuran) were dried according to standard procedures and distilled prior to use or purchased as extra dry reagents (*N*,*N*-dimethylformamide) from Acros Organics (Geel, Belgium). Commercially available reagents were purchased from Sigma-Aldrich (via Merck KGaA, Darmstadt, Germany), TCI Deutschland GmbH (Eschborn, Germany) and were used without further purification.

Analytical thin-layer chromatography (TLC) was used for monitoring reactions. TLC was performed on pre-coated silica gel 60 F254 aluminum plates (Merck KGaA, Darmstadt, Germany) and visualized by exposure to ultraviolet light (UV, 254 nm) and/or staining with a 1:1 mixture of 1 M ethanolic H_2_SO_4_ and 4-methoxyphenol in EtOH (3%). Alternatively, staining was performed with Seebach’s reagent [Cerium phosphomolybdic acid (5.0 g), conc. H_2_SO_4_ (16 mL), water (200 mL) and Cerium (IV) sulfate (2.0 g)]. If not otherwise stated, purification of substances was achieved by standard flash column chromatography on silica (35–70 μm particle size) from Acros Organics (Geel, Belgium). Amberlite^®^ IR120 and Celite^®^ Hyflo Supercel were purchased from Merck KGaA (Darmstadt, Germany).

Microwave-assisted syntheses were carried out in a *Discover microwave* from CEM GmbH (Kamp-Lintfort, Germany). The corresponding reaction conditions were noted in the respective experiments.

Analytical RP-HPLC was performed using a JASCO system (PU-2080 Plus, LG-2080-02-S, DG-2080-53 and MD-2010 Plus, JASCO Deutschland GmbH, Pfungstadt, Germany) on a *Phenomenex luna* column (C18, 5 μm, 250 mm × 4.6 mm). As eluent, a gradient of water (A) and acetonitrile (B) containing 0.1% TFA with a flow rate of 1 mL/min was applied.

NMR spectra (^1^H, ^13^C, ^19^F, 2D NMR) were recorded on Varian 400 MHz and 600 MHz spectrometers (Agilent Technologies, Santa Clara, CA, USA) or on a Bruker Avance III 800 MHz spectrometer equipped with a Cryo-Probe^TM^ (Bruker Corporation, Billerica, MA, USA). The chemical shifts are indicated in parts per million (ppm) and relative to the signal of the deuterated solvent. The following abbreviations are applied to denote the multiplicities: s (singlet), d (doublet), t (triplett), q (quartett) and m (multiplet). The assignment of proton and carbon signals was accomplished by additional COSY, HSQC and HMBC experiments. Anomeric configurations were verified with proton-coupled HSQC experiments when required.

High resolution (HR-ESI) mass spectra were recorded on a Thermo Finnigan LTQ FT spectrometer (Thermo Fisher Scientific, Waltham, MA, USA) either in positive or negative ionization mode. MALDI-TOF spectra were recorded on a Bruker Daltonics Autoflex II Time-of-flight spectrometer (Bruker Corporation, Billerica, MA, USA) equipped with a N_2_-laser (λ = 337 nm).

Optical rotations were measured on a PerkinElmer polarimeter 241 (PerkinElmer, Inc., Waltham, MA, USA) at the Sodium-D-line (589 nm) and at the given temperature in °C. Concentrations, c, are given in g/100 mL, and the solvents used are stated in brackets (CHCl_3_).

### 3.1. Synthesis of Fluorinated Trisaccharides ***3***–***5***

*N*-(Benzyl)-benzyloxycarbonyl-5-aminopentyl-(2,3,4-tri-*O*-benzoyl-6-deoxy-6-fluoro-β-D-glucopyranosyl)-(1→4)-(2,3,6-tri-*O*-benzyl-α-D-glucopyranosyl)-(1→4)-2,3,6-tri-*O*-benzyl-α-D-galactopyranoside (**38**). Acceptor **10** (280 mg, 235 μmol, 1.0 eq.) and fluorinated glucosyl donor **17** (210 mg, 329 μmol, 1.4 eq.) were combined and co-evaporated with toluene (2 × 10 mL). Educts were dried 1 h under high vacuo and subsequently dissolved in dry CH_2_Cl_2_ (15 mL). Freshly activated 4 Å molecular sieves were added, and the mixture was stirred for 1 h at ambient temperature. The reaction was chilled to 0 °C and TMSOTf (4.00 μL, 24.0 μmol, 0.1 eq.) was added. After the reaction was deemed complete (approximately 2.5 h) it was neutralized by the addition of NEt_3_ (300 μL) and filtered through a pad of Celite^®^ Hyflo Supercel. The solvents were removed under reducd pressure and the crude product was subjected to column chromatography (*c*Hex/EtOAc *v*/*v* = 5:1) to obtain **38** (300 mg, 0.18 mmol, 77%) as a colorless oil. *R_f_* = 0.28 (*c*Hex/EtOAc *v*/*v* = 3:1 + 1% NEt_3_). RP HPLC (Luna, 0.1% TFA; 0 min 50% B → 10 min 100% B, flow: 1 mL/min) *t_R_* = 22.19 min, λ = 230 nm. [*α*]_D_^22^ = −9.9° (*c* = 0.6; CHCl_3_). ^1^H NMR (600 MHz, CDCl_3_) δ = 7.97–7.92 (m, 2H, Ar-H), 7.78–7.76 (m, 2H, Ar-H), 7.68–7.64 (m, 2H, Ar-H), 7.58–7.11 (m, 49H, Ar-H), 5.53 (t, *J*_H3″,H2″_ = *J*_H3″,H4″_ = 9.5 Hz, 1H, H-3″), 5.47–5.41 (m, 2H, H-2″, H-4″), 5.20–5.11 (m, 3H, 2 × CH_Cbz_, CH_Bn_), 4.98 (d, *J*_H1′,H2′_ = 3.5 Hz, 1H, H-1′), 4.82 (d, *J*_CH,CH_ = 11.9 Hz, 1H, CH_Bn_), 4.78 (d, *J*_CH,CH_ = 10.6 Hz, 1H, CH_Bn_), 4.71–4.64 (m, 3H, H-1″, 2 × CH_Bn_), 4.59 (d, *J*_CH,CH_ = 12.5 Hz, 1H, CH_Bn_), 4.56–4.50 (m, 2H, H-1, CH_Bn_), 4.50–4.38 (m, 3H, H-6a’’, 2 × NCH_Bn_), 4.37–4.25 (m, 2H, H-6b’’, CH_Bn_), 4.20–4.15 (m, 3H, 3 × CH_Bn_), 4.12–4.00 (m, 5H, H-4′, H-5′,H-4, 2 × CH_Bn_), 3.93–3.87 (m, 2H, H-3′, H-6a), 3.78–3.69 (m, 3H, H-2, H-3, H-5), 3.59–3.51 (m, 3H, H-5″, H-6a’,H-2′), 3.51–3.40 (m, 1H, CH_Linker_), 3.41–3.37 (m, 1H, H-6b), 3.35–3.24 (m, 1H, CH_Linker_), 3.23–3.11 (m, 2H, 2 × CH_Linker_), 2.98 (dd, *J*_H6b,H6a_ = 11.0 Hz, *J*_H6b,H5_ = 1.6 Hz, 1H, H-6b’), 1.56–1.41 (m, 4H, 4 × CH_Linker_), 1.33–1.13 (m, 2H, 2 × CH_Linker_). ^13^C NMR (150 MHz, CDCl_3_) δ = 165.8 (C=O), 165.2 (C=O), 164.7 (C=O), 156.8/156.3 (C=O_Cbz_), 139.5, 139.1, 138.7, 138.4, 138.2, 138.1, 137.0/136.9 (7 × Cq), 133.6, 133.3, 133.2, 129.9, 129.8 (2C), 129.1 (2C), 129.0 (2C), 128.7 (3C), 128.6 (2C), 128.4 (5C), 128.2, 128.0 (2C), 127.7, 127.6, 127.5, 127.4, 127.3 (28 × C-Ar), 100.2 (C-1″), 100.0 (C-1′), 97.9 (C-1), 81.2 (d, *J*_C6″,F_ = 175.8 Hz, C-6″), 80.2 (C-3′), 79.7 (C-2′), 77.7 (C-3/C-2/C-5), 77.3 (2C, C-4, C-4′)*, 75.5 (CH_Bn_), 75.0 (C-3/C-2/C-5), 74.3 (CH_Bn_), 73.7 (CH_Bn_), 73.6 (CH_Bn_), 73.3 (C-3″), 73.0 (CH_Bn_), 72.9 (d, *J*_C5″,F_ = 19.9 Hz, C-5″), 72.5 (CH_Bn_), 72.2 (C-2″), 70.5 (C-5′), 69.5 (C-3/C-2/C-5), 69.1 (d, *J*_C4″,F_ = 6.5 Hz, C-4″), 68.0 (2C, C-6, CH_Linker_), 67.3 (CH_Cbz_), 67.2 (C-6′), 50.6/50.3 (NCH_Bn_), 47.3/46.3 (CH_Linker_), 29.2 (CH_Linker_) 28.1/27.7 (CH_Linker_), 23.5 (CH_Linker_). Due to signal overlap, 70 out of 101 carbon atoms were assigned. *Assigned from HSQC due to signal superimposition with solvent peak. ^19^F NMR (377 MHz, CDCl_3_) δ = -230.5 (td, *J*_F,H6a_ = *J*_F,H6b_ = 46.9 Hz, *J*_F,H5_ = 22.1 Hz). ^1^H-^13^C-coupled HSQC (CDCl_3_) *J*_C1,H1_ = 171 Hz, *J*_C1′H1′_ = 171 Hz, *J*_C1″,H1″_ = 161 Hz. HRMS (ESI^+^) calculated for C_101_H_106_O_20_FN_2_F^+^ [M + NH_4_]^+^: 1686.7352; found: 1686.7282.

5-Aminopentyl-(6-deoxy-6-fluoro-β-D-glucopyranosyl)-(1→4)-(α-D-glucopyranosyl)-(1→4)-α-D-galactopyranoside (**3**). To a stirred solution of the fluorinated trisaccharide **38** (95 mg, 56.9 μmol, 1.0 eq.) in a mixture of MeOH/THF (*v*/*v* = 1:1, 7 mL), NaOMe (1.00 mL, 1.5 M in MeOH) was added. The reaction solution was stirred 6 h at ambient temperature before being neutralized by addition of Amberlite^®^ IR120 The reaction mixture was filtered, and the solvents were removed under reduced pressure. The crude residue was dried for 17 h under high vacuo. Subsequently, the crude residue was dissolved in a mixture of CH_2_Cl_2_/*t*-BuOH/H_2_O (*v*/*v* = 1:6:2 25 mL), and Pd(OH)_2_/C (100 mg) was added under Ar atmosphere. The reaction was then purged three times with H_2_ and stirred for 36 h at ambient temperature. The catalyst was removed by filtration through Celite^®^ Hyflo Supercel, and the solvents were removed under reduced pressure. The crude product was dissolved in H_2_O/MeOH (*v*/*v* = 4:1) and subjected to RP column chromatography (H_2_O/MeOH, *v*/*v* = 4:1) to obtain **3** (30 mg, 50.7 μmol, 89% over 2 steps) after lyophilization. ^1^H NMR (800 MHz, DMSO-d_6_) δ = 8.13–8.05 (m, 2H, NH_2_), 4.82 (d, *J*_H1′,H2′_ = 3.7 Hz, 1H, H-1′), 4.65 (d, *J*_H1,H2_ = 3.6 Hz, 1H, H-1), 4.63– 4.45 (m, 2H, H-6a″, H-6b″), 4.35 (d, *J*_H1″,H2″_ = 7.9 Hz, 1H, H-1″), 4.15 (dt, *J*_H5′,H4′_ = 10.1 Hz, *J*_H5′,H6a′_ = *J*_H5′,H6b′_ = 3.0 Hz, 1H, H-5′), 3.86 (bs, 1H, H-4), 3.74–3.67 (m, 2H, H-6a, H-6a′), 3.67–3.64 (m, 2H, H-3, H-5), 3.61–3.51 (m, 4H, H-2, H-3′, H-6b/H-6b′, CH_Linker_), 3.49–3.41 (m, 2H, H- 5″, H-6b/H-6b′), 3.38 (t, *J*_H4′,H3′_ = *J*_H4′,H5′_ = 9.5 Hz, 1H, H-4′), 3.31 (dt, *J*_CH,CH_ = 9.6 Hz, *J*_CH,CH_ = 6.1 Hz, 1H, CH_Linker_), 3.26 (dd, *J*_H2′,H3′_ = 9.7 Hz, *J*_H2′,H1′_ = 3.7 Hz, 1H, H-2′), 3.22 (t, *J*_H3″,H2″_ = *J*_H3″,H4″_ = 8.9 Hz, 1H, H-3″), 3.11 (t, *_J_*_H4″,H3″_ = *J*_H4″,H5″_ = 9.4 Hz, 1H, H-4″), 2.99 (t, *J*_H2″,H1″_ = *J*_H2″,H3″_ = 8.5 Hz, 1H, H-2″), 2.73 (q, *J*_CH,CH_ = 6.7 Hz, 2H, CH_Linker_), 1.60–1.46 (m, 4H, CH_Linker_), 1.41–1.32 (m, 2H, CH_Linker_). ^13^C NMR (200 MHz, DMSO-d_6_) δ = 102.9 (C-1″), 99.4 (C-1′), 99.0 (C-1), 82.7 (d, *J*_C6″,F_ = 169.1 Hz, C-6″), 79.6 (C-4′), 77.1 (C- 4), 76.3 (C-3″), 74.6 (d, *J*_C5″,F_ = 17.1 Hz, C-5″), 73.2 (C-2″), 72.2 (C-2′), 71.2 (C-3′), 70.9 (C-3/C-5), 70.1 (C-5′), 68.8/68.7 (2C, C-4″, C-3/C-5), 68.4 (C-2), 66.8 (CH_Linker_), 59.4 (C-6/C-6′), 58.9 (C-6/C-6′), 38.7 (CH_Linker_), 28.6 (CH_Linker_), 26.7 (CH_Linker_), 22.8 (CH_Linker_). ^19^F NMR (377 MHz, DMSO-d6) δ = -232.1 (td, *J*_F,H6a_ = *J*_F,H6b_ = 47.8 Hz, *J*_F,H5_ = 23.8 Hz). ^1^H-^13^C-coupled HSQC (DMSO-d6) *J*_C1,H1_ = 167 Hz, *J*_C1′H1′_ = 170 Hz, *J*_C1″,H1″_ = 161 Hz. HRMS (ESI^+^) calculated for C_23_H_43_FNO_15_^+^ [M + H]^+^: 592.2611; found: 592.2610.

*N*-(Benzyl)-benzyloxycarbonyl-5-aminopentyl-(2,3,4,6-tetra-*O*-benzoyl-β-D-glucopyranosyl)-(1→4)-(2,3,6-tri-*O*-benzyl-α-D-glucopyranosyl)-(1→4)-2,3-di-*O*-benzyl-6-deoxy-6-fluoro-α-D-galactopyranoside (**39**). Disaccharide acceptor **12** (100 mg, 90.6 μmol, 1.0 eq.) and donor **16** (101 mg, 136 μmol, 1.5 eq.) were combined and co-evaporated with toluene (2 × 10 mL). Starting materials were dried for 1 h under high vacuo and subsequently dissolved in dry CH_2_Cl_2_ (10 mL). Freshly activated 4 Å molecular sieves were added, and the mixture was stirred for 1 h at ambient temperature. The reaction was cooled to 0 °C and TMSOTf (1.60 μL, 9.00 μmol, 0.1 eq.) was added in one portion. After the TLC indicated complete conversion of acceptor **12**, the reaction was neutralized by addition of NEt_3_ (100 μL) and filtered through a pad of Celite^®^ Hyflo Supercel. The filtrate was washed with 1 M HCl (10 mL), sat. aq. NaHCO_3_ (10 mL) and brine (10 mL) and dried with MgSO_4_. The solvents were removed under reduced pressure, and the crude product was subjected to column chromatography (*c*Hex/EtOAc *v*/*v* = 4:1) to obtain **39** (133 mg, 79.0 μmol, 87%) as a colorless oil. R*_f_*= 0.23 (*c*Hex/EtOAc *v*/*v* = 3:1 + 1% NEt_3_). RP HPLC (Luna, 0.1% TFA; 0 min 50% B → 10 min 100% B, flow: 1 mL/min) *t_R_* = 25.48 min, λ = 230 nm. [*α*]_D_^22^ = +8.4° (*c* = 0.3; CHCl_3_). ^1^H NMR (800 MHz, CD_2_Cl_2_) δ = 7.97–7.95 (m, 2H, Ar-H), 7.90–7.87 (m, 2H, Ar-H), 7.77–7.74 (m, 2H, Ar-H), 7.71–7.69 (m, 2H, Ar-H), 7.55–7.52 (m, 2H, Ar-H), 7.50–7.47 (m, 2H, Ar-H), 7.47–7.09 (m, 43H, Ar-H), 5.61 (t, *J*_H3″,H4″_ = *J*_H3″,H2″_ = 9.5 Hz, 1H, H-3″), 5.57 (t, *J*_H4″,H3″_ = *J*_H4″,H5″_ = 9.6 Hz, 1H, H-4″), 5.51 (dd, *J*_H2″,H3″_ = 9.6 Hz, *J*_H2″,H1″_ = 8.1 Hz, 1H, H-2″), 5.22 (d, *J*_CH,CH_ = 11.0 Hz, 1H, CH_Bn_), 5.13 (d, *J*_CH,CH_ = 20.4 Hz, 2H, CH_Cbz_), 4.86 (d, *J*_H1″,H2″_ = 8.1 Hz, 1H, H-1″), 4.83 (d, *J*_H1′,H2′_ = 3.5 Hz, 1H, H-1′), 4.76 (d, *J*_CH,CH_ = 11.1 Hz, 1H, CH_Bn_), 4.70 (d, *J*_CH,CH_ = 11.3 Hz, 1H, CH_Bn_), 4.68–4.53 (m, 5H, H-1, H-6a, 3 × CH_Bn_), 4.49–4.45 (m, 3H, CH_Bn_, 2 × NCH_Bn_), 4.39 (dd, *J*_H6a″,H6b″_ = 11.9 Hz, *J*_H6a″,H5″_ = 3.3 Hz, 1H, H-6a″), 4.38–4.35 (m, 1H, CH_Bn_), 4.34–4.25 (m, 2H, H-6b, H-6b″), 4.19 (d, *J*_CH,CH_ = 11.9 Hz, 1H, CH_Bn_), 4.15 (d, *J*_CH,CH_ = 12.0 Hz, 1H, CH_Bn_), 4.06 (dd, *J*_H4′,H5′_ = 10.3 Hz, *J*_H4′,H3′_ = 8.8 Hz, 1H, H-4′), 4.00 (dt, *J*_H5′,H3′_ = 10.3 Hz, *J*_H5′,H6a′_ = *J*_H5′,H6b′_ = 2.0 Hz, 1H, H-5′), 3.99–3.95 (m,1H, H-4), 3.89–3.79 (m, 3H, H-5″, H-3′, H-5), 3.73–3.67 (m, 2H, H-2, H-3), 3.56 (dd, *J*^H6a′,H6b′^ = 11.0 Hz, *J*_H6a′,H5′_ = 2.3 Hz, 1H, H-6a′), 3.52–3.42 (m, 2H, H-2′, CH_Linker_), 3.33–3.24 (m, 1H, CH_Linker_), 3.23–3.15 (m, 2H, 2 × CH_Linker_), 3.01 (dd, *J*_H6b′,H6a′_ = 11.0 Hz, *J*_H6b′,H5′_ = 1.6 Hz, 1H, H-6b′), 1.58–1.44 (m, 4H, 4 × CH_Linker_), 1.33–1.16 (m, 2H, 2× CH_Linker_). ^13^C-NMR (200 MHz, CD_2_Cl^2^) δ = 166.4 (C=O), 166.0 (C=O), 165.6 (C=O), 165.2 (C=O), 157.1/156.5 (C=O_Cbz_), 140.2, 139.4, 139.0, 138.9, 138.7, 137.8/137.7 (6 × Cq), 134.0, 133.8, 133.7, 133.5, 130.3, 130.2, 130.1, 130.0, 129.6 (2C), 129.5, 129.3, 129.0 (2C), 128.9 (2C), 128.8 (3C), 128.5, 128.3, 128.2 (2C), 128.1, 128.0, 127.9, 127.7, 127.5 (28 × C-Ar), 100.9 (C-1″), 100.4 (C-1′), 98.4 (C-1), 81.6 (d, *J*C6,F = 164.4 Hz, C-6), 80.6 (C-3′), 80.2 (C-2′), 77.9 (C-4′), 77.5 (C-2/C-3), 77.2 (C-4), 75.6 (2C, CH_Bn_, C-2/C-3), 75.0 (CH_Bn_), 74.1 (CH_Bn_), 73.8 (2C, CH_Bn_, C-3″), 72.8 (2C, CH_Bn_, C-2″), 72.4 (C-5″), 71.1 (C-5′), 70.4 (C-4″), 69.3 (d, *J*_C5,F_ = 24.9 Hz, C-5), 68.6 (CH_Linker_), 67.8 (C-6′), 67.5 (CH_Cbz_), 63.6 (C-6″), 51.0/50.6 (NCH_Bn_), 47.7/46.9 (CH_Linker_), 29.7 (CH_Linker_), 28.5/28.0 (CH_Linker_), 23.9 (CH_Linker_). Due to signal overlap, 69 out of 101 carbon atoms were assigned. ^19^F NMR (377 MHz, CD_2_Cl_2_) δ = −230.8 – −231.3 (m). ^1^H-^13^C-coupled HSQC (CD_2_Cl_2_) *J*_C1,H1_ = 171 Hz, *J*_C1′H1′_ = 171 Hz, *J*_C1″,H1″_ = 166 Hz. HRMS (ESI^+^) calculated for C_101_H_104_FN_2_O_21_^+^ [M + NH_4_]^+^: 1700.7144; found: 1700.7185.

5-Aminopentyl-(β-D-glucopyranosyl)-(1→4)-(α-D-glucopyranosyl)-(1→4)-6-deoxy-6-fluoro-α-D-galacto-pyranoside (**5**). To a stirred solution of the fluorinated trisaccharide **39** (100 mg, 59.4 μmol, 1.0 eq.) in a mixture of MeOH/THF (*v*/*v* = 1:1, 6 mL), NaOMe (1.78 mL, 1.5 M in MeOH) was added. The reaction solution was stirred for 4 h at ambient temperature before being neutralized by the addition of Amberlite^®^ IR120. The reaction mixture was filtered, and the solvents were removed under reduced pressure. The crude residue was dried for 17 h under high vacuo. Subsequently, the crude residue was dissolved in a mixture of MeOH/THF/H_2_O/AcOH (*v*/*v* = 10:5:4:1, 10 mL), and Pd/C (50 mg) was added under Ar atmosphere. The reaction was purged three times with H_2_ and stirred for 27 h at ambient temperature. The catalyst was filtered off by Celite^®^ Hyflo Supercel, and the solvents were removed under reduced pressure. The crude product was dissolved in H_2_O/MeOH (*v*/*v* = 4:1) and subjected to RP column chromatography (H_2_O/MeOH, *v*/*v* = 4:1) to obtain **5** (32 mg, 54.5 μmol, 92% over two steps) after lyophilization. ^1^H NMR (800 MHz, DMSO-d_6_) δ = 4.77–4.57 (m, 4H, H-1, H-1′, H-6a, H-6b), 4.23 (d, *J*_H1″,H2″_ = 7.9 Hz, 1H, H-1″), 4.10–4.03 (m, 1H, H-5′/H-5″), 3.96–3.90 (m, 1H, H-5), 3.83 (s, 1H, H-4), 3.71–3.64 (m, 3H, H-3, H-6a′, H-6a″), 3.61–3.53 (m, 3H, H-2, CH_Linker_, H-6b′/H-6b″), 3.50 (t, *J*_H3′,H2′_ = *J*_H3′,H4′_ = 9.2 Hz, 1H, H-3′), 3.42 (dd, *J*_H6b′/H-6b″,H6a′/H-6a″_ = 11.7 Hz, *J*_H6b′/H-6b″,H5′/H5″_ = 6.4 Hz, 1H, H-6b′/H-6b″), 3.38–3.31 (m, 2H, H-4′, CH_Linker_), 3.29–3.23 (m, 1H, H-2′), 3.16 (s, 2H, H-3″, H-5′/H-5″), 3.06 (t, *J*_H4″, H3″_ = *J*_H4″, H5″_ = 9.3 Hz, 1H, H-4″), 2.98 (t, *J*_H2″, H1″_ = *J*_H2″, H3″_ = 8.6 Hz, 1H, H-2″), 2.68 (s, 2H, 2 × CH_Linker_), 1.56–1.47 (m, 4H, 4 × CH_Linker_), 1.38–1.32 (m, 2H, 2 × CH_Linker_). ^13^C NMR (200 MHz, DMSO-d_6_): δ [ppm] =103.1 (C-1″), 100.1 (C-1′), 99.0 (C-1), 82.8 (d, *J*_C6,F_ = 163.2 Hz, C-6), 79.9 (C-4′), 78.4 (d, *J*_C4,F_ = 6.4 Hz, C-4), 76.8 (C-3″, C-5′/C-5″), 76.5 (C-3″, C-5′/C-5″), 73.3 (C-2″), 72.1 (C-2′), 71.4 (C-3′), 70.4 (C-5′/C-5″), 70.0 (C-4″) 69.7 (d, *J*_C5,F_ = 21.1 Hz, C-5), 68.3 (2C, C-3, C-2), 67.2 (CH_Linker_), 61.0 (C-6′/C-6″), 59.7 (C-6′/C-6″), 39.5 (CH_Linker_)*, 28.6 (CH_Linker_), 28.5 (CH_Linker_), 22.9 (CH_Linker_). *Assigned from HSQC due to signal superimposition with solvent peak. ^19^F NMR (377 MHz, DMSO-d_6_) δ = −227.4 (td, *J*_F,H6_ = 47.4 Hz, *J*_F,H5_ = 14.5 Hz). ^1^H-^13^C-coupled HSQC (DMSO-d_6_) *J*_C1,H1_ = 167 Hz, *J*_C1′H1′_ = 168 Hz, *J*_C1″,H1″_ = 159 Hz. HRMS (ESI^+^) calculated for C_23_H_43_FNO_15_^+^ [M + H]^+^: 592.2611; found: 592.2606.

*N*-(Benzyl)-benzyloxycarbonyl-5-aminopentyl-(2,3,4,6-tetra-*O*-benzoyl-β-D-glucopyranosyl)-(1→4)-(2,3-di-*O*-benzyl-6-*O*-*tert*-butyldimethylsilyl-α-D-glucopyranosyl)-(1→4)-2,3,6-tri-*O*-benzyl-α-D-galactopyranoside (**40**). Acceptor **11** (170 mg, 0.14 mmol, 1.0 eq.) and donor **16** (156 mg, 0.21 mmol, 1.5 eq.) were combined and co-evaporated with toluene (2 × 10 mL). Educts were dried for 1 h under high vacuo and subsequently dissolved in dry CH_2_Cl_2_ (10 mL). Freshly activated 4 Å molecular sieves were added, and the mixture was stirred for 1 h at ambient temperature. The reaction was cooled to 0 °C, and TMSOTf (3.60 μL, 20.0 μmol, 0.1 eq.) was added in one portion. After the reaction was deemed complete by TLC, it was neutralized by the addition of NEt_3_ (100 μL) and filtered through a pad of Celite^®^ Hyflo Supercel. The solvents were removed under reduced pressure, and the crude product was subjected to column chromatography (*c*Hex/EtOAc *v*/*v* = 6:1) to obtain **40** (240 mg, 0.13 mmol, 93%) as a colorless oil. *R_f_* = 0.43 (*c*Hex/EtOAc *v*/*v* = 3:1). RP HPLC (Luna, 0.1% TFA; 0 min 50% B → 10 min 100% B, flow: 1 mL/min) *t_R_* = 39.67 min, λ = 230 nm. [*α*]_D_^22^ = −10.0° (*c* = 1.1, CHCl_3_). ^1^H NMR (600 MHz, CD_2_Cl_2_) δ = 8.02–7.98 (m, 2H, Ar-H), 7.92–7.88 (m, 2H, Ar-H), 7.85–7.83 (m, 2H, Ar-H), 7.80–7.75 (m, 2H, Ar- H), 7.53 (dtd, *J*_CH,CH_ = 8.3 Hz, *J*_CH,CH_ = 7.3 Hz, *J*_CH,CH_ = 1.4 Hz, 2H, Ar-H), 7.47–7.40 (m, 4H, Ar-H), 7.39–7.15 (m, 41H, Ar-H), 5.83 (t, *J*_H3″,H4″_ = *J*_H3″,H2″_ = 9.6 Hz, 1H, H-3″), 5.67–5.60 (m, 2H, H-2″, H-4″), 5.31–5.26 (m, 2H, H-1″, CH_Bn_), 5.13 (d, *J*_CH,CH_ = 12.1 Hz, 2H, CH_Cbz_), 4.91 (d, *J*_H1′,H2′_ = 3.6 Hz, 1H, H-1′), 4.75 (d, *J*_CH,CH_ = 11.1 Hz, 1H, CH_Bn_), 4.72 (d, *J*_CH,CH_ = 11.4 Hz, 1H, CH_Bn_), 4.63 (s, 1H, H-1), 4.58–4.52 (m, 3H, 3× CH_Bn_), 4.49 (dd, *J*_H6a″,H6b″_ = 12.0 Hz, *J*_H6a″,H5″_ = 3.0 Hz, 1H, H-6a″), 4.45 (s, 2H, NCH_Bn_), 4.35–4.30 (m, 2H, H-6b″, CH_Bn_), 4.29–4.22 (m, 3H, 3 × CH_Bn_), 4.13–4.07 (m, 3H, H-5″, H-4′, H-5′), 4.00 (s, 1H, H-4), 3.91 (dd, *J*_H3′,H2′/H-4′_ = 9.8 Hz, *J*_H3′,H2′/H-4′_ = 8.1 Hz, 1H, H-3′), 3.87–3.70 (m, 4H, H-6a′, H-6a, H-2, H-3), 3.68–3.64 (m, 1H, H-5), 3.51–3.45 (m, 1H, CH_Linker_), 3.42–3.34 (m, 3H, H-2′, H-6b′, H-6b), 3.32–3.23 (m, 1H, CH_Linker_) 3.22–3.11 (m, 2H, 2× CH_Linker_), 1.60–1.41 (m, 4H, 4× CH_Linker_), 1.33–1.14 (m, 2H, 2× CH_Linker_), 0.95 (s, 9H, Si-*t-*Bu), 0.09 (s, 3H, Si-CH_3_), 0.02 (s, 3H, Si-CH_3_). ^13^C NMR (150 MHz, CD_2_Cl_2_) δ = 166.4 (C=O), 166.1 (C=O), 165.7 (C=O), 165.3 (C=O), 157.1/156.5 (C=O_Cbz_), 140.3, 139.4, 139.2, 139.0, 138.9, 137.8/137.7 (6 × Cq), 134.0, 133.9, 133.8, 133.5, 130.4, 130.3, 130.1 (2C), 129.7, 129.6, 129.5, 129.0 (3C), 128.9 (2C), 128.8 (3C), 128.7, 128.5, 128.2, 128.1 (2C), 128.0 (2C), 127.5, 127.4 (28 × C-Ar), 101.0 (C-1″), 99.7 (C-1′), 98.3 (C-1), 80.6 (2C, C-2′, C-3′), 77.7 (C-4′), 77.6 (C-5), 76.5 (C-4), 75.7 (CH_Bn_), 75.6 (C-2), 75.1 (CH_Bn_), 74.2 (C-3″), 73.6 (CH_Bn_), 73.4 (CH_Bn_), 73.0 (C-2″/C-4″), 72.8 (C-5″), 72.3 (CH_Bn_), 71.9 (C-5′), 70.5 (C-2″/C-4″), 69.8 (C-3), 68.4 (2C, C-6, CH_Linker_), 67.4 (CH_Cbz_), 63.8 (C-6″), 61.5 (C-6′), 51.0/50.6 (NCH_Bn_), 47.7/46.9 (CH_Linker_), 29.7 (CH_Linker_), 28.6/28.0 (CH_Linker_), 26.4 (3C, Si-*t-*Bu), 23.9 (CH_Linker_), 18.7 (Cq, Si-*t-*Bu), −4.60 (Si-CH_3_), −4.80 (Si-CH_3_). Due to signal overlap, 75 out of 107 carbon atoms were assigned. HRMS (ESI^+^) calculated for C_107_H_119_N_2_O_22_Si^+^ [M + NH_4_]^+^: 1812.8052; found: 1812.8062.

*N*-(Benzyl)-benzyloxycarbonyl-5-aminopentyl-(2,3,4,6-tetra-*O*-benzoyl-β-D-glucopyranosyl)-(1→4)-(2,3-di-*O*-benzyl-α-D-glucopyranosyl)-(1→4)-2,3,6-tri-*O*-benzyl-α-D-galactopyranoside (**41**). To a stirred solution of **40** (180 mg, 100 μmol, 1.0 eq.) in a mixture of CH_2_Cl_2_/MeOH (*v*/*v* = 1:1, 20 mL), *p*-TsOH·H_2_O (19.0 mg, 100 μmol, 1.0 eq.) was added. The reaction solution was stirred 7 h at 50 °C and 12 h at ambient temperature. After the complete conversion of the starting material was observed by TLC, the reaction was neutralized by the addition of NEt_3_ (200 μL). The organic solvents were removed under reduced pressure, and the crude residue was dissolved in CH_2_Cl_2_ (75 mL), washed with 1 M HCl (30 mL), sat. aq. NaHCO_3_ (30 mL) and brine (20 mL) and dried with MgSO_4_. The solvent was removed under reduced pressure, and the crude product was subjected to column chromatography (*c*Hex/EtOAc *v*/*v* = 2:1) to obtain **41** (150 mg, 89.2 μmol, 89%) as a colorless oil. *R_f_* = 0.22 (*c*Hex/EtOAc *v*/*v* = 2:1). RP HPLC (Luna, 0.1% TFA; 0 min 50% B → 10 min 100% B, flow: 1 mL/min) *t_R_* = 20.19 min, λ = 230 nm. [*α*]_D_^22^ = +15.0° (*c* = 0.3, CHCl_3_). ^1^H NMR (800 MHz, CDCl_3_) δ = 7.95 (d, *J*_CH,CH_ = 7.7 Hz, 2H, Ar-H), 7.87 (d, *J*_CH,CH_ = 7.7 Hz, 2H, Ar-H), 7.85–7.83 (m, 2H, Ar-H), 7.80–7.77 (m, 2H, Ar-H), 7.50 (td, *J*_CH,CH_ = 7.4 Hz, *J*_CH,CH_ = 1.4 Hz, 1H, Ar-H), 7.48–7.46 (m, 1H, Ar-H), 7.42–7.13 (m, 45H, Ar-H), 5.87 (t, *J*_H3″,H4″_ = *J*_H3″,H2″_ = 9.7 Hz, 1H, H-3″), 5.68 (t, *J*_H4″,H3″_ = *J*_H4″,H5″_ = 9.8 Hz, 1H, H-4″), 5.65–5.60 (m, 1H, H-2″), 5.21–5.13 (m, 4H, H-1″, 2 × CH_Cbz_, CH_Bn_), 4.87 (d, *J*_H1′,H2′_ = 3.5 Hz, 1H, H-1′), 4.85 (d, *J*_CH,CH_ = 11.3 Hz, 1H, CH_Bn_), 4.72 (d, *J*_CH,CH_ = 11.9 Hz, 1H, CH_Bn_), 4.62–4.57 (m, 3H, H-1, 2 × CH_Bn_), 4.53 (d, *J*_CH,CH_ = 12.4 Hz, 1H, CH_Bn_), 4.46 (d, *J*_CH,CH_ = 26.7 Hz, 2H, NCH_Bn_), 4.44–4.38 (m, 2H, H-6a″, CH_Bn_), 4.32–4.28 (m, 2H, H-6b″, CH_Bn_), 4.26–4.19 (m, 2H, 2 × CH_Bn_), 4.05 (dd, *J*_H5′,H4′_ = 10.2 Hz, *J*_H5′,H6′_ = 2.4 Hz, 1H, H-5′), 4.03 (dt, *J*_H5″,H4″_ = 8.9 Hz, *J*_H5″,H6a″_ = *J*_H5″,H6b″_ = 4.1 Hz, 1H, H-5″), 3.97 (t, *J*_H3′,H2′_ = *J*_H3′,H2′_ = 9.2 Hz, 1H, H-3′), 3.95–3.89 (m, 2H, H-4, H-4′), 3.86–3.81 (m, 1H, H-6a), 3.80–3.74 (m, 2H, H-2, H-3), 3.74–3.70 (m, 1H, H-5), 3.60–3.55 (m, 1H, H-6a′), 3.53–3.41 (m, 3H, H-2′, H-6b, CH_Linker_), 3.39–3.28 (m, 2H, H-6b′, CH_Linker_), 3.22 (t, *J*_CH,CH_ = 7.7 Hz, 1H, CH_Linker_), 3.14 (t, *J*_CH,CH_ = 7.5 Hz, 1H, CH_Linker_), 1.59–1.41 (m, 4H, 4 × CH_Linker_), 1.34–1.11 (m, 2H, 2 × CH_Linker_). ^13^C NMR (200 MHz, CDCl_3_) δ = 166.1 (C=O), 165.9 (C=O), 165.2 (C=O), 165.0 (C=O), 156.8/156.3 (C=O_Cbz_), 139.4, 138.7, 138.4 (2C), 138.1 (2C,), 137.0/136.9 (7 × Cq), 133.4 (2C), 133.3, 133.0, 129.9 (2C), 129.8 (2C), 129.7, 129.0, 128.9 (2C), 128.6 (2C), 128.5 (3C), 128.4 (4C), 128.3, 128.2, 128.0, 127.9 (2C), 127.7, 127.6 (2C), 127.5, 127.4, 127.3, 127.2, 127.0 (34 × C-Ar), 101.5 (C-1″), 99.6 (C-1′), 97.7 (C-1), 80.2 (C-3′), 80.0 (C-2′), 78.2 (C-4′), 77.8 (C-4), 77.4 (C-5)*, 75.3 (C-2), 75.1 (CH_Bn_), 74.1 (CH_Bn_), 73.5 (CH_Bn_), 73.4 (C-3″), 73.0 (CH_Bn_), 72.6 (CH_Bn_), 72.5 (C-2″), 72.3 (C-5″), 71.1 (C-5′), 69.9 (C-4″), 69.5 (C-3), 68.4 (C-6), 68.1/68.0 (CH_Linker_), 67.3/67.2 (CH_Cbz_), 63.2 (C-6″), 60.5 (C-6′), 50.6/50.3 (NCH_Bn_), 47.2/46.3 (CH_Linker_), 29.2/29.1 (CH_Linker_), 28.0/27.6 (CH_Linker_), 23.5 (CH_Linker_). Due to signal overlap, 76 out of 101 carbon atoms were assigned. *Assigned from HSQC due to signal superimposition with solvent peak. HRMS (ESI^+^) calculated for C_101_H_102_NO_22_^+^ [M + H]^+^: 1697.7153; found: 1697.7183.

*N*-(Benzyl)-benzyloxycarbonyl-5-aminopentyl-(2,3,4,6-tetra-*O*-benzoyl-β-D-glucopyranosyl)-(1→4)-(2,3-di-*O*-benzyl-6-deoxy-6-fluoro-α-D-glucopyranosyl)-(1→4)-2,3,6-tri-*O*-benzyl-α-D-galactopyranoside (**42**). To a stirred solution of **41** (150 mg, 89.2 μmol, 1.0 eq.) in dry CH_2_Cl_2_ (4 mL), 2,4,6-collidine (48.0 μL, 357 μmol, 4.0 eq.) and DAST (24.0 μL, 179 μmol, 2.0 eq.) were added. The reaction was subjected to microwave irradiation (80 °C, 100 W) for 1 h and subsequently poured into MeOH (50 mL). The solvents were removed under reduced pressure, obtaining a brown oil, which was dissolved in CH_2_Cl_2_ (50 mL). The organic layer was washed with sat. aq. NaHCO_3_ (20 mL), 1 M HCl (20 mL) and brine (15 mL) and dried with MgSO_4_. The solvent was removed under reduced pressure, and the crude product was subjected to column chromatography (*c*Hex/EtOAc *v*/*v* = 4:1) to obtain **42** (113 mg, 67.2 μmol, 75%) as a colorless oil. *R_f_* = 0.46 (*c*Hex/EtOAc *v*/*v* = 2:1). RP HPLC (Luna, 0.1% TFA; 0 min 50% B → 10 min 100% B, flow: 1 mL/min) *t_R_* = 21.80 min, λ = 230 nm. [*α*]_D_^22^ = +21.4° (*c* = 0.3, CHCl_3_). ^1^H NMR (600 MHz, CDCl_3)_ δ = 7.95 (dd, *J*_CH,CH_ = 8.3 Hz, *J*_CH,CH_ = 1.4 Hz, 2H, Ar-H), 7.84 (td, *J*_CH,CH_ = 7.8 Hz, *J*_CH,CH_ = 7.3 Hz *J*_CH,CH_ = 1.4 Hz, 4H, Ar-H), 7.81–7.76 (m, 2H, Ar-H), 7.54–7.11 (m, 47H, Ar-H), 5.85 (t, *J*_H3″,H4″_ = *J*_H3″,H2″_ = 9.7 Hz, 1H, H-3″), 5.67 (t, *J*_H4″,H3″_ = *J*_H4″,H5″_ = 9.7 Hz, 1H, H-4″), 5.62 (dd, *J*_H2″,H3″_ = 9.8 Hz, *J*_H2″,H1″_ = 8.0 Hz, 1H, H-2″), 5.21–5.14 (m, 3H, CH_Cbz_, CH_Bn_), 5.11 (d, *J*_H1″,H2″_ = 8.0 Hz, 1H, H-1″), 4.95 (d, *J*_H1′,H2′_ = 3.5 Hz, 1H, H-1′), 4.90 (d, *J*_CH,CH_ = 11.5 Hz, 1H, CH_Bn_), 4.71 (d, *J*_CH,CH_ = 12.0 Hz, 1H, CH_Bn_), 4.67 (d, *J*_CH,CH_ = 12.2 Hz, 1H, CH_Bn_), 4.64 (bs, 1H, H-1), 4.60 (d, *J*_CH,CH_ = 11.9 Hz, 1H, CH_Bn_), 4.55 (d, *J*_CH,CH_ = 12.3 Hz, 1H, CH_Bn_), 4.47 (d, *J*_CH,CH_ = 17.2 Hz, 2H, NCH_Bn_), 4.44–4.27 (m, 4H, H6a/b′, H6a″, 2 × CH_Bn_), 4.26–4.20 (m, 3H, H-6b″, 2 × CH_Bn_), 4.19–4.09 (m, 1H, H-5′), 4.02–3.96 (m, 3H, H-5″, H-3′, H-4), 3.96–3.91 (m, 1H, H-4′), 3.91–3.70 (m, 5H, H-6a, H-6a/b′, H-3, H-2, H-5), 3.54–3.49 (m, 1H, CH_Linker_), 3.48 (dd, *J*_H2′,H3′_ = 9.6 Hz, *J*_H2′,H1′_ = 3.5 Hz, 1H, H-2′), 3.41 (dd, *J*_H6b,H6a_ = 9.5 Hz, *J*_H6b,H5_ = 5.9 Hz, 1H, H-6b), 3.38–3.29 (m, 1H, CH_Linker_), 3.27–3.10 (m, 2H, 2 × CH_Linker_), 1.61–1.43 (m, 4H, 4 × CH_Linker_), 1.33–1.15 (m, 2H, 2 × CH_Linker_). ^13^C NMR (150 MHz, CDCl_3_) δ = 166.1 (C=O), 165.9 (C=O), 165.2 (C=O), 165.1 (C=O), 156.8/156.3 (C=O_Cbz_), 139.4, 138.6, 138.4, 138.3, 138.2, 138.1 137.0/136.9 (7 × Cq), 133.4 (2C), 133.3, 133.0, 129.9 (2C), 129.8 (2C), 129.7, 129.1, 129.0, 128.9, 128.6 (2C), 128.5 (2C), 128.4 (2C), 128.3, 128.0 (3C), 127.7 (2C), 127.6, 127.5, 127.4, 127.2, 126.8 (29 × C-Ar), 101.6 (C-1″), 99.5 (C-1′), 97.7 (C-1), 81.2 (d, *J*_C6′,F_ = 170.7 Hz, C-6′), 80.2 (C-5″/C-3′/C-4), 79.8 (C-2′), 77.7 (d, *J*_C4′,F_ = 5.2 Hz C-4′), 77.3 (2C, C-5″/C-3′/C-4, C-3/C-2/C-5), 75.2 (C-3/C-2/C-5), 74.9 (CH_Bn_), 74.2 (CH_Bn_), 73.4 (2C, C-3″, CH_Bn_), 73.1 (CH_Bn_), 72.8 (CH_Bn_), 72.6 (C-2″), 72.4 (C-5″/C-3′/C-4), 70.0 (d, *J*_C5′,F_ = 17.3 Hz, C-5′), 69.8 (C-4″), 69.3 (C-3/C-2/C-5), 68.1 (CH_Linker_), 67.9 (C-6), 67.3 (CH_Cbz_), 63.1 (C-6″), 50.6/50.3 (NCH_Bn_), 47.3/46.3 (CH_Linker_), 29.2 (CH_Linker_), 28.1/27.7 (CH_Linker_), 23.5 (CH_Linker_). Due to signal overlap, 71 out of 101 carbon atoms were assigned. ^19^F-NMR (377 MHz, CDCl_3_) δ = -234.9 (td, *J*_F,H6a_ = *J*_F,H6b_ = 48.1 Hz, *J*_F,H5_ = 34.3 Hz). ^1^H-^13^C-coupled HSQC (CDCl_3_) *J*_C1,H1_ = 172 Hz, *J*_C1′H1′_ = 171 Hz, *J*_C1″,H1″_ = 164 Hz. HRMS (ESI^+^) calculated for C_101_H_104_O_21_N_2_F^+^ [M + NH_4_]^+^: 1700.7144; found: 1700. 7186.

5-Aminopentyl-(β-D-glucopyranosyl)-(1→4)-(6-deoxy-6-fluoro-α-D-glucopyrano-syl)-(1→4)-α-D-galactopyranoside (**4**). To a stirred solution of **42** (70 mg, 41.6 μmol, 1.0 eq.) in MeOH/THF (*v*/*v* = 1:1, 8 mL), NaOMe (1.25 mL, 0.5 M in MeOH) was added. The solution was stirred for 18 h at ambient temperature before being neutralized by the addition of Amberlite^®^ IR120. The reaction mixture was filtered, and the solvents were removed under reduced pressure. The crude residue was dried for 17 h under high vacuo and then dissolved in a mixture of MeOH/THF/H_2_O/AcOH (*v*/*v* = 19:5:4:1, 15 mL). Pd/C (40 mg) was added under Ar atmosphere, and the reaction was purged three times with H_2_. After stirring for 72 h at ambient temperature, the catalyst was removed by filtration through a short plug of Celite^®^ Hyflo Supercel, and the solvents were removed under reduced pressure. The crude product was then dissolved in H_2_O/MeOH (*v*/*v* = 4:1) and subjected to RP column chromatography (H_2_O/MeOH, *v*/*v* = 4:1) to obtain **4** (22 mg, 37.4 μmol, 90% over 2 steps) after lyophilization. ^1^H NMR (800 MHz, DMSO-d_6_): δ = 4.88–4.78 (m, 2H, H-1′, H-6a′), 4.64 (d, *J*_H1,H2_ = 3.7 Hz, 1H, H-1), 4.52–4.40 (m, 2H, H-6b′, H-5′), 4.13 (d, *J*_H1″,H2″_ = 7.9 Hz, 1H, H-1″), 3.86 (d, *J*_H4,H3_ = 3.3 Hz, 1H, H-4), 3.75 (dd, *J*_H6a,H6b_ = 10.5 Hz, *J*_H6a,H5_ = 8.3 Hz, 1H, H-6a), 3.70–3.65 (m, 2H, H-6a″, H-3), 3.62 (dd, *J*_H5,H6b_ = 8.3 Hz, *J*_H5,H6a_ = 5.9 Hz, 1H, H-5), 3.59–3.50 (m, 3H, H-2, H-3′, CH_Linker_), 3.46–3.40 (m, 2H, H-6b, H-6b″), 3.33–3.25 (m, 3H, H-2′, H-4′, CH_Linker_), 3.19–3.13 (m, 2H, H-5″, H-3″), 3.05 (t, *J*_H4″,H3″_ = *J*_H4″,H5″_ = 9.2 Hz, 1H, H-4″), 2.99 (t, *J*_H2″,H1″_ = *J*_H2″,H3″_ = 8.5 Hz, 1H, H-2″), 2.63 (t, *J*_CH,CH_ = 7.2 Hz, 2H, 2 × CH_Linker_), 1.56–1.43 (m, 4H, 4 × CH_Linker_), 1.34 (p, *J*_CH,CH_ = 7.5 Hz, 2H, 2 × CH_Linker_). ^13^C NMR (200 MHz, DMSO-d_6_) δ = 103.6 (C-1″), 99.2 (C-1′), 99.0 (C-1), 81.8 (d, *J*_C6′,F_ = 168.3 Hz, C-6′), 79.4 (d, *J*_C4′,F_ = 4.3 Hz, C-4′), 76.9 (C- 3″/C-5″), 76.5 (C-3″/C-5″), 76.4 (C-4), 73.3 (C-2″), 71.9 (C-2′), 71.3 (C-3′), 71.0 (C-5), 70.1 (C-4″), 68.5 (C-3), 68.4 (d, *J*_C5′,F_ = 17.4 Hz, C-5′), 68.3 (C-2), 67.0, (CH_Linker_), 61.0 (C-6″), 58.7 (C-6), 40.0 (CH_Linker_), 29.7(CH_Linker_), 28.8(CH_Linker)_, 23.0 (CH_Linker_). ^19^F NMR (377 MHz, DMSO-d_6_) δ = −235.0 (td, *J*F,H6 = 47.7 Hz, *J*F,H5 = 33.6 Hz). ^1^H-^13^C-coupled HSQC (DMSO-d_6_) *J*_C1,H1_ = 167 Hz, *J*_C1′H1′_ = 169 Hz, *J*_C1″,H1″_ = 160 Hz. HRMS (ESI^+^) calculated for C_23_H_43_FNO_15_^+^ [M + H]^+^: 592.2611; found: 592.2608.

### 3.2. Synthesis of Fluorinated Tetrasaccharides ***6***–***9***

*N*-(Benzyl)benzyloxycarbonyl-5-aminopentyl-(2,3,4-tri-*O*-benzoyl-6-deoxy-6-fluoro-β-D-glucopyranosyl)-(1→4)-(2,3,6-tri-*O*-benzoyl-β-D-glucopyranosyl)-(1→4)-(2,3,6-tri-*O*-benzyl-α-D-glucopyranosyl)-(1→4)-2,3,6-tri-*O*-benzyl-α-D-galactopyranoside (**43**). The donor **14** (200 mg, 180 μmol, 1.5 eq.) and acceptor **10** (143 mg, 120 μmol, 1.0 eq.) were combined, co-evaporated with dry toluene (15 mL) and dried under high vacuo for 1 h. Starting materials were dissolved in dry CH_2_Cl_2_ (10 mL) and stirred for 1 h over freshly activated 4 Å molecular sieves. The reaction mixture was cooled to 0 °C, and TMSOTf (2.19 μL, 12.0 μmol, 0.1 eq.) was added in one portion. The reaction was stirred 1.5 h at 0 °C before another portion of TMSOTf (3.50 μL, 19.2 μmol, 0.16 eq.) was added. After further stirring for 0.5 h, the reaction was deemed complete by TLC and was neutralized by the addition of NEt_3_ (100 μL). The mixture was diluted with CH_2_Cl_2_ and filtered through a pad of Celite^®^ Hyflo Supercel. The filtrate was washed with 1 M HCl (10 mL), sat. aq. NaHCO_3_ (10 mL) and brine (10 mL) and was dried over MgSO_4_. The crude product was subjected to column chromatography (cHex/EtOAc *v*/*v* = 4:1) to obtain **43** (225 mg, 105 μmol, 87%) as a colorless oil. *R_f_* = 0.26 (cHex/EtOAc *v*/*v* = 3:1 + 1% NEt_3_). RP HPLC (Luna, 0.1% TFA; 0 min 50% B → 10 min 100% B, flow: 1 mL/min): *t_R_* = 25.62 min, λ = 230 nm. ${\left[\alpha \right]}_{D}^{24}$= +12.6° (*c* = 0.3, CHCl_3_). ^1^H NMR (800 MHz, CD_2_Cl_2_) δ = 8.05–7.99 (m, 6H, Ar-H), 7.84–7.79 (m, 2H, Ar-H), 7.77–7.72 (m, 4H, Ar-H), 7.62–7.59 (m, 1H, Ar-H), 7.56–7.49 (m, 3H, Ar-H), 7.47–7.39 (m, 8H, Ar-H), 7.38–7.22 (m, 34H, Ar-H), 7.21–7.15 (m, 7H, Ar-H), 7.14–7.09 (m, 2H, Ar-H), 7.07–7.00 (m, 3H, Ar-H), 5.68 (t, J_H3″′,H4″′_ = J_H3″′,H2‴_ = 9.6 Hz, 1H, H-3‴), 5.49–5.46 (m, 1H, H-3″), 5.45–5.40 (m 2H, H-2‴, H-2″), 5.23–5.17 (m, 2H, H-4‴, CH_Bn_), 5.14 (d, J_CH,CH_ = 20.3 Hz, 2H, CH_Cbz_), 4.90 (d, J_H1′,H2′_ = 3.7 Hz, 1H, H-1′), 4.81 (d, J_H1‴,H2‴_ = 7.9 Hz, 1H, H-1‴), 4.68 (d, J_H1″,H2″_ = 8.0 Hz, 1H, H-1″), 4.66 (d, J_CH,CH_ = 11.2 Hz, 1H, CH_Bn_), 4.63 (d, J_CH,CH_ = 11.3 Hz, 1H, CH_Bn_), 4.59 (s, 1H, H-1), 4.52–4.47 (m, 3H, 4 × CH_Bn_), 4.46 (s, 2H, NCH_Bn_), 4.42 (dd, J_H6a″,H6b″_ = 11.8 Hz, J_H6a″,H5″_ = 1.8 Hz, 1H, H-6a″), 4.31 (d, J_CH,CH_ = 12.5 Hz, 1H, CH_Bn_), 4.28 (dd, J_H6b″,H6a″_ = 11.7 Hz, J_H6b″,H5″_ = 5.0 Hz, 1H, H-6b″), 4.25–4.20 (m, 3H, 3 × CH_Bn_), 4.18 (dd, J_H4″,H5″_ = 9.9 Hz, J_H4″,H3″_ = 8.8 Hz, 1H, H-4″), 4.11 (d, J_CH,CH_ = 11.9 Hz, 1H, CH_Bn_), 4.06 (dt, J_H5′,H4′_ = 10.3 Hz, J_H5′,H6a′_ = J_H5′,H6b′_ = 2.0 Hz, 1H, H-5′), 4.02–3.94 (m, 3H, H-4′, H-4, H-6a‴), 3.83–3.79 (m, 2H, H-3′, H-6a), 3.79–3.66 (m, 4H, H-2, H-3, H-5, H-6b‴), 3.63 (dddd, J_H5‴,F_ = 20.5 Hz, J_H5‴,H4‴_ = 10.2 Hz, J_H5‴,H6a‴_ = 5.3 Hz, J_H5‴,H6b‴_ = 2.3 Hz, 1H, H-5‴), 3.53 (dd, J_H6a′,H6b′_ = 10.8 Hz, J_H6a′,H5′_ = 2.4 Hz, 1H, H-6a′), 3.52–3.44 (m, 1H, CH_Linker_), 3.43 (ddd, J_H5″,H4″_ = 10.0 Hz, J_H5″,H6b″_ = 4.9 Hz, J_H5″,H6b″_ = 1.8 Hz, 1H, H-5″), 3.39–3.34 (m, 2H, H-2′, H-6b), 3.32–3.24 (m, 1H, CH_Linker_), 3.23–3.14 (m, 2H, 2 × CH_Linker_), 3.01 (dd, J_H6b′,H6a′_ = 10.8 Hz, J_H6b′,H5′_ = 1.6 Hz, 1H, H-6b′), 1.55–1.43 (m, 4H, 4 × CH_Linker_), 1.33–1.15 (m, 2H, 2 × CH_Linker_). ^13^C-NMR (200 MHz, CD_2_Cl_2_) δ = 166.2 (C=O), 165.9 (C=O), 165.8 (C=O), 165.4 (C=O), 165.3 (2C, 2 × C=O), 157.1/156.5 (C=O_Cbz_), 140.2, 139.4, 139.1, 139.0, 138.9, 138.8, 138.6 137.8/137.7 (8 × Cq), 134.1 (2C), 133.8 (2C), 133.7, 133.6, 130.4 (2C), 130.3, 130.2 (2C), 130.1, 129.7, 129.4, 129.2 (3C), 129.1, 129.0 (3C), 128.9 (3C), 128.8 (3C), 128.7 (2C), 128.6, 128.4, 128.3, 128.2 (2C), 128.1, 128.0 (2C), 127.9 (2C), 127.7, 127.6, 127.3 (42 × C-Ar), 101.0 (C-1‴), 100.7 (C-1″), 99.9 (C-1′), 98.3 (C-1), 81.3 (J_C6‴,F_ = 171.3 Hz, C-6‴), 80.7 (C-3′), 80.1 (C-2′), 77.9 (C-4′), 77.8 (C-2/C-3), 77.0 (C-4), 76.7 (C-4″), 75.5 (C-2/C-3), 75.4 (CH_Bn_), 74.8 (CH_Bn_), 74.0 (C-3″), 73.7 (2C, 2 × CH_Bn_), 73.6 (d, J_C5‴,F_ = 19.8 Hz, C-5‴), 73.4 (2C, C-3‴, C-5″), 73.2 (CH_Bn_), 72.9 (C-2″), 72.6 (CH_Bn_), 72.3 (C-2‴), 70.9 (C-5′), 69.8 (C-5), 68.9 (d, JC4‴,F = 7.0 Hz, C-4‴), 68.4 (C-6), 68.3 (CH_Linker_), 67.9 (C-6′), 67.4 (CH_Cbz_), 63.2 (C-6″), 51.0/50.6 (NCH_Bn_), 47.7/46.9 (CH_Linker_), 29.7 (CH_Linker_), 28.5/28.0 (CH_Linker_), 23.9 (CH_Linker_). Due to signal overlap, 94 out of 128 carbon atoms were assigned. ^19^F NMR (377 MHz, CD_2_Cl_2_) δ = −229.95 (td, J_F,H6a‴_ = J_F,H6b‴_ = 46.7 Hz, J_F,H5‴_ = 20.3 Hz). ^1^H-^13^C-coupled HSQC (CD_2_Cl_2_) J_C1,H1_ = 169 Hz, J_C1′H1′_ = 170 Hz, J_C1″,H1″_ = 165 Hz, J_C1‴,H1‴_ = 161 Hz. MALDI-TOF calculated for C_129_H_129_FNO_29_^+^ [M + H + MeOH]^+^: 2175.87; found: 2175.90.

5-Aminopentyl-(6-deoxy-6-fluoro-β-D-glucopyranosyl)-(1→4)-(β-D-glucopyranosyl)-(1→4)-(α-D-glucopyranosyl)-(1→4)-α-D-galactopyranoside (**6**). To a stirred solution of tetrasaccharide **43** (110 mg, 51.4 μmol, 1.0 eq.) in a mixture of MeOH/THF (*v*/*v* = 1:1, 6 mL), NaOMe (1.50 mL, 0.5 M in MeOH) was added. The reaction solution was stirred for 5 h at ambient temperature before being neutralized by the addition of Amberlite^®^ IR120.The reaction mixture was filtered, and the solvents were removed under reduced pressure. The crude residue was dried for 17 h under high vacuo. The crude residue was then dissolved in a mixture of MeOH/THF/H_2_O/AcOH (*v*/*v* = 19:5:4:1, 10 mL), and Pd/C (50 mg) was added under Ar atmosphere. The reaction was purged three times with H_2_ and was stirred for 40 h at ambient temperature. The catalyst was filtered off by Celite^®^ Hyflo Supercel, and the solvents were removed under reduced pressure. The crude product was dissolved in H_2_O/MeOH (*v*/*v* = 4:1) and subjected to RP column chromatography (H_2_O/MeOH, *v*/*v* = 4:1) to obtain **6** (36 mg, 46.6 μmol, 91% over two steps) after lyophilization. ^1^H NMR (800 MHz, DMSO-d_6_) δ = 4.82 (d, *J*_H1′,H2′_ = 3.7 Hz, 1H, H-1′), 4.65 (d, *J*_H1,H2_ = 3.6 Hz, 1H, H-1), 4.62–4.46 (m, 2H, H-6a‴, H-6b‴), 4.36 (d, *J*_H1‴,H2‴_ = 7.9 Hz, 1H, H-1‴), 4.31 (d, *J*_H1″,H2″_ = 7.9 Hz, 1H, H-1″), 4.13 (dt, *J*_H5′,H6b′_ = *J*_H5′,H4′_ = 10.3 Hz, *J*_H5′,H6a′_ = 3.1 Hz, 1H, H-5′), 3.87–3.85 (m, 1H, H-4), 3.77 (d, *J*_H6a″,H6b″_ = 11.4 Hz, 1H, H-6a″), 3.72 (dd, *J*_H6a, H6b_ = 10.6 Hz, *J*_H6a, H5_ = 8.0 Hz, 1H, H-6a), 3.68 (dd, *J*_H6a′,H6b′_ = 11.9 Hz, *J*_H6a′,H5′_ = 3.5 Hz, 1H, H-6a′), 3.66–3.62 (m, 2H, H-3, H-5), 3.61–3.52 (m, 5H, H-3′, H-2, H-6b′, H-6b″, CH_Linker_), 3.50–3.42 (m, 2H, H-5‴, H-6b), 3.39–3.29 (m, 5H, CH_Linker_, H-3″, H-4′, H-4″, H-5″), 3.26 (dd, *J*_H2′,H3′_ = 9.6, *J*_H2′,H1′_ = 3.7 Hz, 1H, H-2′), 3.20 (t, *J*_H3‴,H2‴_ = *J*_H3‴,H4‴_ = 9.0 Hz, 1H, H-3‴), 3.10 (t, *J*_H4‴,H3‴_ = *J*_H4‴,H5‴_ = 9.4 Hz, 1H, H-4‴), 3.06 (t, *J*_H2″,H1″_ = *J*_H2″,H3″_ = 8.1 Hz, 1H, H-2″), 3.00 (t, *J*_H2‴,H1‴_ = *J*_H2‴,H3‴_ = 8.5 Hz, 1H, H-2‴), 2.67 (s, 2H, 2 × CH_Linker_), 1.57–1.43 (m, 4H, 4 × CH_Linker_), 1.35 (p, *J*_CH,CH_ = 7.6 Hz, 2H, 2 × CH_Linker_). ^13^C NMR (200 MHz, DMSO-d_6_) δ = 102.9 (C-1‴), 102.7 (C-1″), 99.4 (C-1′), 99.0 (C-1), 82.7 (d, *J*_C6‴,F_ = 169.0 Hz, C-6‴), 79.9 (C-4′), 79.7 (C-4″), 77.2 (C-4), 76.2 (C-3‴), 74.8 (C-3″/C-5″), 74.7 (d, *J*_C5‴,F_ = 17.1 Hz, C-5‴), 74.5 (C-3″/C-5″), 73.1 (2C, C-2″, C-2‴), 72.1 (C-2′), 71.4 (C-3′), 71.0 (C-3), 70.2 (C-5′), 68.8 (C-5), 68.7 (*J*_C4‴,F_ = 6.6 Hz, C-4‴), 68.5 (C-2), 66.9 (CH_Linker_), 60.2 (C-6″), 59.7 (C-6′), 59.0 (C-6), 39.4 (CH_Linker_)*, 28.8 (CH_Linker_), 28.7 (CH_Linker_), 22.9 (CH_Linker_). *Assigned from HSQC due to signal superimposition with solvent peak. ^19^F NMR (377 MHz, DMSO-d_6_) δ = −232.1 (td, *J*_F,H6a‴_ = *J*_F,H6b‴_ = 47.8 Hz, *J*_F,H5‴_ = 23.4 Hz). ^1^H-^13^C-coupled HSQC (DMSO-d_6_) *J*_C1,H1_ = 167 Hz, *J*_C1′H1′_ = 169 Hz, *J*_C1″,H1″_ = 158 Hz, *J*_C1‴,H1‴_ = 161 Hz. HRMS (ESI^+^) calculated for C_29_H_53_O_20_NF^+^ [M + H]^+^: 754.3139; found: 754.3134.

*N*-(Benzyl)-benzyloxycarbonyl-5-aminopentyl-(2,3,4,6-tetra-*O*-benzoyl-β-D-glucopyranosyl)-(1→4)-(2,3-di-*O*-benzoyl-6-deoxy-6-fluoro-β-D-glucopyranosyl)-(1→4)-(2,3,6-tri-*O*-benzyl-α-D-glucopyranosyl)-(1→4)-2,3,6-tri-*O*-benzyl-α-D-galactopyranoside (**44**). The donor **15** (210 mg, 188 μmol, 1.5 eq.) and acceptor **10** (150 mg, 126 μmol, 1.0 eq.) were combined, co-evaporated with dry toluene (15 mL) and dried under high vacuo for 1 h. Starting materials were dissolved in dry CH_2_Cl_2_ (10 mL) and stirred for 1 h over freshly activated 4 Å molecular sieves. The reaction was cooled to 0 °C, and TMSOTf (2.30 μL, 12.6 μmol, 0.1 eq.) was added in one portion. The reaction was stirred 1.5 h at 0 °C before another portion of TMSOTf (2.30 μL, 12.6 μmol, 0.1 eq.) was added. The reaction was slowly warmed to ambient temperature and stirred until complete conversion of the donor **15** was observed by TLC. The reaction was stopped by the addition of NEt_3_ (100 μL) and was diluted with CH_2_Cl_2_ and filtered through Celite^®^ Hyflo Supercel. The filtrate was washed with 1 M HCl (10 mL), sat. aq. NaHCO_3_ (10 mL) and brine (10 mL) and dried with MgSO_4_. The crude product was subjected to column chromatography (*^c^*Hex/EtOAc *v*/*v* = 3:1) to obtain **44** (225 mg, 104 μmol, 83%) as a colorless oil. *Rf* = 0.27 (*^c^*Hex/EtOAc *v*/*v* = 2:1 + 1% NEt_3_). RP HPLC (Luna, 0.1% TFA; 0 min 50% B → 10 min 100% B, flow: 1 mL/min): *t_R_* = 25.80 min, λ = 230 nm. ${\left[\alpha \right]}_{D}^{24}$ = +7.2° (*c* = 0.3, CHCl_3_). ^1^H NMR (800 MHz, CDCl3) δ = 8.05–8.02 (m, 2H, Ar-H), 7.91–7.89 (m, 2H, Ar-H), 7.88–7.85 (m, 2H, Ar-H), 7.77 (ddt, *J*_CH,CH_ = 10.9 Hz, *J*_CH,CH_ = 6.8 Hz, *J*_CH,CH_ = 1.4 Hz, 5H, Ar-H), 7.66–7.63 (m, 2H, Ar-H), 7.62–7.58 (m, 1H, Ar-H), 7.50–7.09 (m, 56H, Ar-H), 5.79 (t, *J*_H3‴,H4‴_ = *J*_H3‴,H2‴_ = 9.7 Hz, 1H, H-3‴), 5.57 (dd, *J*_H2‴,H3‴_ = 9.9 Hz, *J*_H2‴,H1‴_ = 7.9 Hz, 1H, H-2‴), 5.44 (t, *J*_H4‴,H3‴_ = *J*_H4‴,H5‴_ = 9.6 Hz, 1H, H-4‴), 5.32 (t, *J*_H3″,H4″_ = *J*_H3″,H2″_ = 9.3 Hz, 1H, H-3″), 5.29 (dd, *J*_H2″,H3″_ = 9.9 Hz, *J*_H2″,H1″_ = 7.8 Hz, 1H, H-2″), 5.15 (d, *J*_CH,CH_ =18.6 Hz, 2H, CH_Cbz_), 5.05 (d, *J*_CH,CH_ = 10.1 Hz, 1H, CH_Bn_), 4.92 (d, *J*_H1′,H2′_ = 3.7 Hz, 1H, H-1′), 4.89 (d, *J*_H1‴,H2‴_ = 7.9 Hz, 1H, H-1‴), 4.77 (d, *J*_CH,CH_ = 11.9 Hz, 1H, CH_Bn_), 4.68 (d, *J*_CH,CH_ = 10.1 Hz, 1H, CH_Bn_), 4.61 (d, *J*_CH,CH_ = 11.9 Hz, 1H, CH_Bn_), 4.59 (d, *J*_CH,CH_ = 12.1 Hz, 1H, CH_Bn_), 4.53 (t, *J*_CH,CH_ = 11.7 Hz, 2H 2 × CH_Bn_), 4.50–4.42 (m, 4H, NCH_Bn_, H-1, H-6a″), 4.42–4.34 (m, 1H, H-6b″), 4.31 (d, *J*_H1″,H2″_ = 7.8 Hz, 1H, H-1″), 4.28 (d, *J*_CH,CH_ = 12.5 Hz, 1H, CH_Bn_), 4.18–4.10 (m, 4H, H-4″, 3 × CH_Bn_), 4.03 (dt, *J*_H5′,H4′_ = 10.0 Hz, *J*_H5′,H6′_ = 2.0 Hz, 1H, H-5′), 3.98 (s, 1H, H-4), 3.93–3.78 (m, 7H, H-3′, H-4′, H-5‴, H-6a, H-6a‴, H-6b‴, CH_Bn_), 3.74–3.66 (m, 3H, H-2, H-3, H-5), 3.48–3.38 (m, 3H, H-2′, H-6a′, CH_Linker_), 3.38–3.33 (m, 1H, H-6b), 3.34–3.23 (m, 1H, CH_Linker_), 3.19 (t, *J*_CH,CH_ = 7.7 Hz, 1H, CH_Linker_), 3.11 (t, *J*_CH,CH_ = 7.6 Hz, 1H, CH_Linker_), 2.96–2.85 (m, 2H, H-5″, H-6b′), 1.56–1.44 (m, 4H, 4 × CH_Linker_), 1.32–1.08 (m, 2H, 2 × CH_Linker_). ^13^C NMR (200 MHz, CDCl3) δ = 165.8 (2C, 2 × C=O), 165.4 (C=O), 165.1 (C=O), 164.8 (C=O), 164.7 (C=O), 156.8/156.3 (C=O_Cbz_), 139.2, 139.0, 138.6, 138.4, 138.3, 138.1, 138.0, 137.7, 137.0/136.9 (9 × Cq), 133.8, 133.5, 133.4, 133.2, 133.1, 129.9, 129.8, 129.7 (2C), 129.6, 129.5, 129.2, 129.1 (2C), 129.0, 128.8 (2C), 128.7 (2C), 128.5, 128.4 (4C), 128.3, 128.2, 128.0, 127.9, 127.7, 127.6, 127.5, 127.4 (2C), 127.3 (34 × C-Ar), 101.2 (C-1‴), 100.2 (C-1″), 99.9 (C-1′), 97.9 (C-1), 80.6 (d, *J*_C6″,F_ = 173.7 Hz, C-6″), 80.1 (C-3′), 79.6 (C-2′), 77.5 (C-3/C-5), 77.2 (C-4)*, 77.1 (C-4′)*, 75.7 (CH_Bn_), 75.4 (d, *J*_C4″,F_ = 6.1 Hz, C-4″), 74.9 (C-2), 74.2 (CH_Bn_), 73.7 (CH_Bn_), 73.6 (d, *J*_C5″,F_ = 19.3 Hz, C-5″), 73.5 (CH_Bn_), 73.2 (CH_Bn_), 73.1 (C-3‴/C-3″), 73.0 (C-3‴/C-3″), 72.5 (CH_Bn_), 72.3 (C-2″), 72.2 (C-5‴), 72.0 (C-2‴), 70.5 (C-5′), 69.5 (C-4‴), 69.4 (C-3/C-5), 68.1/68.0 (CH_Linker_), 67.9 (C-6), 67.3/67.2 (CH_Cbz_), 67.0 (C-6′), 62.7 (C-6‴), 50.6/50.3 (NCH_Bn_), 47.3/46.3 (CH_Linker_), 29.2/29.1 (CH_Linker_), 28.0/27.6 (CH_Linker_), 23.5 (CH_Linker_). Due to signal overlap, 87 out of 128 carbon atoms were assigned. *Assigned from HSQC due to signal superimposition with solvent peaks. ^19^F NMR (377 MHz, CDCl3) δ = −230.5 (td, *J*_F,H6″_ = 47.8 Hz, *J*_F,H5″_ = 30.8 Hz). ^1^H-^13^C-coupled HSQC (CD_2_Cl_2_) *J*_C1,H1_ = 170 Hz, *J*_C1′H1′_ = 169 Hz, *J*_C1″,H1″_ = 164 Hz, *J*_C1‴,H1‴_ = 162 Hz. MALDI-TOF calculated for C_128_H_124_FNO_28_^+^ [M + NH_4_]^+^: 2160.87; found: 2160.95.

5-Aminopentyl-(β-D-glucopyranosyl)-(1→4)-(6-deoxy-6-fluoro-β-D-glucopyranosyl)-(1→4)-(α-D-glucopyranosyl)-(1→4)-α-D-galactopyranoside (**7**). To a stirred solution of **44** (100 mg, 46.7 μmol, 1.0 eq.) in MeOH/THF (*v*/*v* = 1:1, 10 mL), NaOMe (1.40 mL, 0.5 M in MeOH) was added. The reaction solution was stirred for 17 h at ambient temperature before being neutralized by the addition of Amberlite^®^ IR120. The reaction was filtered, and the solvents were removed under reduced pressure. The crude residue was dried for 17 h under high vacuo. The crude residue was then dissolved in a mixture of MeOH/THF/H_2_O/AcOH (*v*/*v* = 19:5:4:1, 40 mL), and Pd/C (50 mg) was added under Ar atmosphere. The reaction was purged three times with H_2_ and was stirred for 40 h at ambient temperature. The catalyst was filtered off by Celite^®^ Hyflo Supercel, and the solvents were removed under reduced pressure. The crude product was dissolved in H_2_O/MeOH (*v*/*v* = 4:1) and subjected to RP column chromatography (H_2_O/MeOH, *v*/*v* = 4:1) to obtain **7** (33 mg, 44.0 μmol, 94% over two steps) after lyophilization. ^1^H NMR (800 MHz, DMSO-d_6_) δ = 4.82 (d, *J*_H1′,H2′_ = 3.9 Hz, 1H, H-1′), 4.78–4.62 (m, 3H, H-6a″, H-6b″, H-1), 4.40 (d, *J*_H1″,H2″_ = 7.9 Hz, 1H, H-1″), 4.19 (d, *J*_H1‴,H2‴_ = 7.9 Hz, 1H, H-1‴), 4.16 (dt, *J*_H5′H4*′*_ = 10.1 Hz, *J*_H5′H6a*′*_ = *J*_H5′H6b*′*_ = 3.2 Hz, 1H, H-5′), 3.86 (s, 1H, H-4), 3.75–3.62 (m, 6H, H-6a, H-6a′, H-6a‴,H-3, H-5, H-5″), 3.61–3.52 (m, 4H, CH_Linker_, H-2, H-3′, H-6b‴), 3.44 (dd, *J*_H6b,H6a_ = 10.6 Hz, *J*_H6b,H5_ = 5.8 Hz, 1H, H-6b), 3.42–3.37 (m, 3H, H-6b′, H-4′, H-3″), 3.35–3.29 (m, 2H, H-4″, CH_Linker_), 3.26 (dd, *J*_H2′,H3′_ = 9.7 Hz, *J*_H2′,H1′_ = 3.6 Hz, 1H, H-2′), 3.21–3.12 (m, 2H, H-5‴, H-3‴), 3.07 (t, *J*_H2″,H1″_ = *J*_H2″,H3″_ = 8.4 Hz, 1H, H-2″), 3.04 (t, *J*_H4‴,H3‴_ = *J*_H4‴,H5‴_ = 9.2 Hz, 1H, H-4‴), 2.99 (t, *J*_H2‴,H1‴_ = *J*_H2‴,H3‴_ = 8.5 Hz, 1H, H-2‴), 2.67 (s, 2H, 2 × CH_Linker_), 1.55–1.46 (m, 4H, 4 × CH_Linker_), 1.38–1.32 (m, 2H, 2 × CH_Linker_). ^13^C NMR (200 MHz, DMSO-d_6_) δ = 103.4 (C-1‴), 102.6 (C-1″), 99.4 (C-1′), 99.0 (C-1), 82.1 (d, *J*_C6″,F_ = 167.5 Hz, C-6″), 79.8 (C-4′), 79.1(d, *J*_C4″,F_ = 5.5 Hz, C-4″), 77.2 (C-4), 77.0 (C-3‴/C-5‴), 76.4 (C-3‴/C-5‴), 74.6 (C-3″), 73.2 (C-2‴), 72.9–72.8 (2C, C-2″, C-5″ [d, *J*_C5″,F_ = 16.7 Hz]), 72.2 (C-2′), 71.3 (C-3′), 71.0 (C-3/C-5), 70.1 (C-4‴), 70.0 (C-5′), 68.8 (C-3/C-5), 68.5 (C-2), 66.9 (CH_Linker_), 61.1 (C-6′), 59.5 (C-6‴), 59.0 (C-6), 39.4 (CH_Linker_)*, 28.7 (CH_Linker_), 28.6 (CH_Linker_), 22.9 (CH_Linker_). *Assigned by HSQC due to signal superimposition with solvent peak. ^19^F NMR (377 MHz, DMSO-d_6_) δ = −231.9 (td, *J*_F,H6a″_ = *J*_F,H6b″_ = 47.5 Hz, *J*_F,H5″_ = 23.2 Hz). ^1^H-^13^C-coupled HSQC (DMSO-d_6_) *J*_C1,H1_ = 166 Hz, *J*_C1′H1′_ = 170 Hz, *J*_C1″,H1″_ = 160 Hz, *J*_C1‴,H1‴_ = 161 Hz. HRMS (ESI^+^) calculated for C_29_H_53_O_20_NF^+^ [M + H]^+^: 754.3139; found: 754.3127.

*N*-(Benzyl)benzyloxycarbonyl-5-aminopentyl-(2,3,4,6-tetra-*O*-benzoyl-β-D-glucopyranosyl)-(1→4)-(2,3,6-tri-*O*-benzoyl-β-D-glucopyranosyl)-(1→4)-(2,3,6-tri-*O*-benzyl-α-D-glucopyranosyl)-(1→4)-2,3-di-*O*-benzyl-6-deoxy-6-fluoro-α-D-galactopyranoside (**45**). The donor **13** (165 mg, 136 μmol, 1.5 eq.) and acceptor **12** (100 mg, 96.6 μmol, 1.0 eq.) were combined, co-evaporated with dry toluene (5 mL) and dried under high vacuo for 1 h. The starting materials were then dissolved in dry CH_2_Cl_2_ (10 mL), and freshly dried 4 Å molecular sieves were added. The mixture was stirred for 40 min at ambient temperature, cooled to 0 °C, and TMSOTf (4.00 μL, 23.0 μmol, 0.1 eq.) was added. The reaction was slowly warmed to room temperature and stirred for 1.5 h. Another portion of TMSOTf (10.0 μL, 57.5 μmol, 0.25 eq.) was added, and the reaction was stirred further 15 min before being stopped by the addition of NEt_3_ (100 μL). The mixture was diluted with CH_2_Cl_2_ and filtered through a short plug of Celite^®^ Hyflo Supercel. The filtrate was washed with 1 M HCl (10 mL), sat. aq. NaHCO_3_ (10 mL) and brine (10 mL) and was dried over MgSO_4_. The crude product was subjected to column chromatography (*^c^*Hex/EtOAc *v*/*v* = 3:1) to obtain **45** (165 mg, 76.4 μmol, 84%) as a colorless oil. *Rf* = 0.39 (*^c^*Hex/EtOAc *v*/*v* = 3:1 + 1% NEt_3_). RP HPLC (Luna, 0.1% TFA; 0 min 50% B → 10 min 100% B, flow: 1 mL/min): *t_R_* = 23.76 min, λ = 230 nm. [*α*]_D_^22^ = +30.0° (*c* = 0.33; CHCl_3_). ^1^H NMR (800 MHz, CDCl3) δ = 8.07–8.03 (m, 2H, Ar-H), 7.94–7.88 (m, 6H, Ar-H), 7.75–7.71 (m, 4H, Ar-H), 7.69–7.64 (m, 2H, Ar-H), 7.51 (t, *J*_CH,CH_ = 7.1 Hz, 2H, Ar-H), 7.49–7.12 (m, 49H, Ar-H), 7.11 (dd, *J*_CH,CH_ = 7.2, *J*_CH,CH_ = 2.3 Hz, 2H, Ar-H), 7.02–6.94 (m, 3H, Ar-H), 5.65 (t, *J*_H3‴,H4‴_ = *J*_H3‴,H2‴_ = 9.6 Hz, 1H, H-3‴), 5.53 (dd, *J*_H2‴,H3‴_ = 9.7 Hz, *J*_H2‴,H1‴_ = 7.9 Hz, 1H, H-2‴), 5.35–5.27 (m, 3H, H-4‴, H-2″,H-3″), 5.15 (d, *J*_CH,CH_ = 19.9 Hz, 2H, CH_Cbz_), 5.11 (d, *J*_CH,CH_ = 11.0 Hz, 1H, CH_Bn_), 4.78 (d, *J*_H1′,H2′_ = 3.6 Hz, 1H, H-1′), 4.73–4.62 (m, 3H, H-1‴, H-6a, CH_Bn_), 4.62–4.54 (m, 3H, 3 × CH_Bn_), 4.51–4.48 (m, 2H, 2 × CH_Bn_), 4.48–4.43 (m, 3H, NCH_Bn_, H-1), 4.39–4.35 (m, 1H, H-1″), 4.35–4.31 (m, 2H, H-6a″, CH_Bn_), 4.32–4.18 (m, 2H, H-6b″, H-6b), 4.10 (d, *J*_CH,CH_ = 12.0 Hz, 1H, CH_Bn_), 4.06–4.01 (m, 1H, H-4″), 3.95–3.89 (m, 4H, H-5′, H-4, H-6a‴, CH_Bn_), 3.88 (t, *J*_H4′,H3′_ = *J*_H4′,H5′_ = 9.5 Hz, 1H, H-4′), 3.85–3.78 (m, 1H, H-5), 3.76 (t, *J*_H3′,H4′_ = *J*_H3′,H2′_ = 9.3 Hz, 1H, H-3′), 3.73–3.68 (m, 1H, H-3), 3.65 (dd, *J*_H2,H3_ = 10.0 Hz, *J*_H2,H1_ = 3.8 Hz, 1H, H-2), 3.63–3.58 (m, 2H, H-5‴, H-6b‴), 3.49–3.39 (m, 2H, H-6a′, CH_Linker_), 3.37 (dd, *J*_H2′,H3′_ = 9.8 Hz, *J*_H2′,H1′_ = 3.6 Hz, 1H, H-2′), 3.34–3.24 (m, 1H, CH_Linker_), 3.23–3.18 (m, 2H, H-5″, CH_Linker_), 3.15–3.11 (m, 1H, CH_Linker_), 2.88 (dd, *J*_H6b′,H6a′_ = 10.7 Hz, *J*_H6b′,H5′_ = 1.9 Hz, 1H, H-6b′), 1.65–1.40 (m, 4H, 4 × CH_Linker_), 1.25–1.09 (m, 2H, 2 × CH_Linker_). ^13^C NMR (200 MHz, CDCl3) δ = 165.8 (C=O), 165.7 (2C, 2 × C=O), 165.4 (C=O), 165.1(C=O), 164.9 (2C, 2 × C=O), 156.8/156.3 (C=O_Cbz_), 139.5, 138.9, 138.3, 138.2, 138.1, 138.0, 137.7, 137.0/136.9 (8 × Cq), 133.6, 133.5, 133.3 (2C), 133.1, 129.9 (2C), 129.8 (2C), 129.7, 129.6, 129.2 (2C), 128.9, 128.8, 128.7 (2C), 128.6, 128.5 (3C), 128.4 (3C), 128.0, 127.8, 127.6, 127.5, 127.3 (29 × C-Ar), 100.9 (C-1‴), 100.3 (C-1′), 100.1 (C-1″), 98.0 (C-1), 80.8 (d, *J*_C6,F_ = 160.8 Hz, C-6), 80.2 (C-3′), 78.9 (C-2′), 77.3 (C-4′)*, 77.1 (C-3)*, 77.0 (C-4)*, 76.3 (C-4″), 75.0 (CH_Bn_), 74.9 (C-2), 74.3 (CH_Bn_), 73.8 (CH_Bn_), 73.6 (CH_Bn_), 73.3 (C-3″), 72.9 (C-3‴), 72.7 (C-5″), 72.6 (CH_Bn_), 72.3 (2C, C-5‴, C-2″), 71.9 (C-2‴), 70.6 (C-5′), 69.7 (C-4‴), 68.7 (d, *J*_C5,F_ = 25.3 Hz, C-5), 68.2 (CH_Linker_), 67.3 (CH_Cbz_), 66.8 (C-6′), 62.8 (C-6″), 62.7 (C-6‴), 50.6/50.3 (NCH_Bn_), 47.2/46.3 (CH_Linker_), 29.2/29.1 (CH_Linker_), 28.0/27.6 (CH_Linker_), 23.5 (CH_Linker_). Due to signal overlap, 81 out of 128 carbon atoms were assigned. *Assigned by HSQC due to signal superimposition with solvent peak. ^19^F NMR (377 MHz, CDCl_3_) δ = −230.6 – −231.1 (m). ^1^H-^13^C-coupled HSQC (CDCl_3_) *J*_C1,H1_ = 170 Hz, *J*_C1′H1′_ = 168 Hz, *J*_C1″,H1″_ = 163 Hz, *J*_C1‴,H1‴_ = 163 Hz. MALDI-TOF calculated for C_129_H_129_FNO_29_^+^ [M + H + MeOH]^+^: 2189.85; found: 2189.95.

5-Aminopentyl-(β-D-glucopyranosyl)-(1→4)-(β-D-glucopyranosyl)-(1→4)-(α-D-glucopyranosyl)-(1→4)-6-deoxy-6-fluoro-α-D-galactopyranoside (**9**). To a stirred solution of tetrasaccharide **45** (100 mg, 46.4 μmol, 1.0 eq.) in a mixture of MeOH/THF (*v*/*v* = 1:1, 10 mL), NaOMe (1.40 mL, 0.5 M in MeOH) was added. The reaction solution was stirred for 8 h at ambient temperature before being neutralized by the addition of Amberlite^®^ IR120. The reaction mixture was filtered, and the solvents were removed under reduced pressure. The crude residue was dried for 17 h under high vacuo. The crude residue was dissolved in a mixture of MeOH/THF/H_2_O/AcOH (*v*/*v* = 19:5:4:1, 20 mL), and Pd/C (50 mg) was added under Ar atmosphere. The reaction was purged three times with H_2_ and stirred for 48 h at ambient temperature. The catalyst was removed by filtration through Celite^®^ Hyflo Supercel and the solvents were removed under reduced pressure. The crude product was dissolved in H_2_O/MeOH (*v*/*v* = 4:1) and subjected to RP column chromatography (H_2_O/MeOH, *v*/*v* = 4:1) to obtain **9** (30 mg, 39.5 μmol, 85% over two steps) after lyophilization. ^1^H NMR (800 MHz, DMSO-d_6_) δ = 4.78–4.57 (m, 4H, H-1, H-1′, H-6a, H-6b), 4.30 (d, *J*_H1″,H2″_ = 7.9 Hz, 1H, H-1″), 4.24 (d, *J*_H1‴,H2‴_ = 7.9 Hz, 1H, H-1‴), 4.10–4.07 (m, 1H, H-5′), 3.96–3.90 (m, 1H, H-5), 3.83 (bs, 1H, H-4), 3.77 (d, *J*_H-6a″;H-6b″_ = 11.2 Hz, 1H, H-6a″), 3.71–3.65 (m, 3H, H-3, H-6a′,H-6a‴), 3.61–3.54 (m, 4H, H-2, H-6b′, H-6b″, CH_Linker_), 3.51 (t, *J*_H3′,H2′_ = *J*_H3′,H4′_ = 9.1 Hz, 1H, H-3′), 3.41 (dd, *J*_H6b‴,H6a‴_ = 11.7, *J*_H6b‴,H5‴_ = 6.6 Hz, 1H, H-6b‴), 3.38–3.31 (m, 5H, H-4′, H-3″, H-4″, H-5″, CH_Linker_), 3.26 (dd, *J*_H2′,H3′_ = 9.6 Hz, *J*_H2′,H1′_ = 3.7 Hz, 1H, H-2′), 3.21–3.13 (m, 2H, H-3‴,H-5‴), 3.07–3.03 (m, 2H, H-4‴, H-2″), 2.98 (t, *J*_H2‴,H1‴_ = *J*_H2‴,H3‴_ = 8.5 Hz, 1H, H-2‴), 2.65 (s, 2H, 2 × CH_Linker_), 1.56–1.46 (m, 4H, 4 × CH_Linker_), 1.42–1.29 (m, 2H, 2 × CH_Linker_). ^13^C NMR (200 MHz, DMSO-d_6_) δ = 103.3 (C-1‴), 102.7 (C-1″), 100.1 (C-1′), 99.0 (C-1), 82.8 (d, *J*_C6,F_ = 163.2Hz, C-6), 80.3 (C-4″), 79.8 (C-4′), 78.4 (d, *J*_C4,F_ = 5.7 Hz, C-4), 76.9 (C-5‴), 76.5 (C-3‴), 74.8 (2C, C-3″, C-5″), 73.3 (C-2‴), 73.0 (C-4‴/C-2″), 72.0 (C-2′), 71.3 (C-3′), 70.4 (C-5′), 70.0 (C-4‴/C-2″), 69.7 (d, *J*_C5,F_ = 21.1 Hz, C-5), 68.3 (2C, C-2, C-3), 67.2 (CH_Linker_), 61.0 (C-6‴), 60.3 (C-6″), 59.6 (C-6′), 39.6 (CH_Linker_)*, 29.2 (CH_Linker_), 28.6 (CH_Linker_), 22.9 (CH_Linker_). *Assigned by HSQC due to signal superimposition with solvent peak. ^19^F NMR (377 MHz, DMSO-d_6_) δ = −227.4 (td, *J*_F,H6a_ = *J*_F,H6b_ = 47.1 Hz, *J*_F,H5_ = 14.4 Hz). ^1^H-^13^C-coupled HSQC (DMSO-d_6_) *J*_C1,H1_ = 169 Hz, *J*_C1′H1′_ = 168 Hz, *J*_C1″,H1″_ = 160 Hz, *J*_C1‴,H1‴_ = 161 Hz. HRMS (ESI^+^) calculated for C_29_H_53_FNO_20_^+^ [M + H]^+^: 754.3139; found: 754.3128.

*N*-(Benzyl)-benzyloxycarbonyl-5-aminopentyl-(2,3,4,6-tetra-*O*-benzoyl-β-D-glucopyranosyl)-(1→4)-(2,3,6-tri-*O*-benzoyl-β-D-glucopyranosyl)-(1→4)-(2,3-di-*O*-benzyl-α-D-glucopyranosyl)-(1→4)-2,3,6-tri-*O*-benzyl-α-D-galactopyranoside (**46**). The donor **13** (420 mg, 345 μmol, 1.5 eq.) and TBS-protected acceptor **11** (280 mg, 230 μmol, 1.0 eq.) were combined, co-evaporated with dry toluene (5 mL) and dry CH_2_Cl_2_ (5 mL) and dried under high vacuo for 1 h. The starting materials were then dissolved in dry CH_2_Cl_2_ (10 mL), and freshly dried 4 Å molecular sieves were added to the reaction solution. The mixture was stirred for 1 h at ambient temperature and cooled to −20 °C before TMSOTf (4.00 μL, 23.0 μmol, 0.1 eq.) was added. The reaction was allowed to slowly warm to room temperature before another portion of TMSOTf (4.00 μL, 23.0 μmol, 0.1 eq.) was added after 0.5 h. The reaction was stirred for further 1.5 h before being stopped by addition of NEt_3_ (100 μL). The mixture was diluted with CH_2_Cl_2_ and filtered through a short plug of Celite^®^ Hyflo Supercel. The organic phase was washed with 1 M HCl (10 mL), sat. aq. NaHCO_3_ (10 mL) and brine (10 mL) and was dried over MgSO_4_. The crude product was subjected to column chromatography (*^c^*Hex/EtOAc *v*/*v* = 4:1) to obtain the TBS-protected tetrasaccharide intermediate **46** as an inseparable mixture, together with a decomposition product of the cellobiose donor **13**. The product was dissolved in CH_2_Cl_2_/MeOH (20 mL, *v*/*v* = 1:1), and *p-*TsOH (44.0 mg, 231 μmol, 1.0 eq.) was added. The reaction was warmed to 50 °C and stirred 6 h before being neutralized by the addition of NEt_3_ (200 μL). The solvents were removed under reduced pressure, and the crude product was subjected to column chromatography (*^c^*Hex/EtOAc *v*/*v* = 3:1) to obtain **47** (150 μmol, 65% over 2 steps) as an amorphous solid. *Rf* = 0.30 (*^c^*Hex/EtOAc *v*/*v* = 2:1). RP HPLC (Luna, 0.1% TFA; 0 min 50% B → 10 min 100% B, flow: 1 mL/min): *t_R_* = 22.72 min, λ = 230 nm. ${\left[\alpha \right]}_{D}^{24}$ = +30.1° (*c* = 0.5, CHCl_3_). ^1^H NMR (800 MHz, CDCl3) δ = 8.00 –7.97 (m, 4H, Ar-H), 7.96–7.92 (m, 4H, Ar-H), 7.84 (d, *J*_CH,CH_ = 7.7 Hz, 2H, Ar-H), 7.74 (t, *J*_CH,CH_ = 7.6 Hz, 4H, Ar-H), 7.55–7.50 (m, 2H, Ar-H), 7.47 (t, *J*_CH,CH_ = 7.3 Hz, 1H, Ar-H), 7.45–7.11 (m, 50H, Ar-H), 6.99 (t, *J*_CH,CH_ = 7.6 Hz, 2H, Ar-H), 6.90 (t, *J*_CH,CH_ = 7.3 Hz, 1H, Ar-H), 5.72 (t, *J*_H3″,H4″_ = *J*_H3″,H2″_ = 9.4 Hz, 1H, H-3″), 5.63 (t, *J*_H3‴,H4‴_ = *J*_H3‴,H2‴_ = 9.6 Hz, 1H, H-3‴), 5.50 (dd, *J*_H2″,H3″_ = 9.8 Hz, *J*_H2″,H1″_ = 8.1 Hz, 1H, H-2″), 5.45 (dd, *J*_H2‴,H3‴_ = 9.6 Hz, *J*_H2‴,H1‴_ = 7.9 Hz, 1H, H-2‴), 5.29 (t, *J*_H4‴,H3‴_ = *J*_H4‴,H5‴_ = 9.6 Hz, 1H, H-4‴), 5.16 (d, *J*_CH,CH_ = 18.7 Hz, 2H, CH_Cbz_), 5.07 (d, *J*_CH,CH_ = 11.4 Hz, 1H, CH_Bn_), 5.01 (d, *J*_H1″,H2″_ = 8.1 Hz, 1H, H-1″), 4.82–4.77 (m, 3H, H-1‴, H-1′, CH_Bn_), 4.63–4.55 (m, 3H, H-1, 2 × CH_Bn_), 4.53–4.48 (m, 2H, 2 × CH_Bn_), 4.48–4.41 (m, 3H, NCH_Bn_, CH_Bn_), 4.34–4.28 (m, 2H, CH_Bn_, H-6″/H-6‴), 4.26–4.16 (m, 4H, H-4″, H-6″/H-6‴, 2 × CH_Bn_), 4.02–3.96 (m, 2H, H-5′, H-6″/H-6‴), 3.91 (t, *J*_H3′,H4′_ = *J*_H3′,H2′_ = 9.2 Hz, 1H, H-3′), 3.88 (s, 1H, H-4), 3.82–3.62 (m, 7H, H-5‴, H-6a, H-6″/H-6‴, H-4′, H-2, H-3, H-5), 3.61–3.56 (m, 1H, H-5″), 3.50–3.44 (m, 2H, H-6a′, CH_Linker_), 3.41 (dd, *J*_H6b,H6a_ = 9.4 Hz, *J*_H6b,H5_ = 6.1 Hz, 1H, H-6b), 3.36 (dd, *J*_H2′,H3′_ = 9.8 Hz, *J*_H2′,H1′_ = 3.5 Hz, 1H, H-2′), 3.34–3.25 (m, 2H, H-6b′, CH_Linker_), 3.23–3.19 (m, 1H, CH_Linker_), 3.16–3.10 (m, 1H, CH_Linker_), 1.57–1.40 (m, 4H, 4 × CH_Linker_), 1.26–1.11 (m, 2H, 2 × CH_Linker_).^13^C NMR (200 MHz, CDCl3) δ = 165.8 (C=O), 165.7 (2C, 2 × C=O), 165.5 (C=O), 165.2 (C=O), 165.1 (C=O), 164.7 (C=O), 156.8/156.3 (C=O_Cbz_), 139.4, 138.6, 138.5, 138.3, 138.1, 138.0, 137.0/136.9 (7 × Cq), 133.5 (2C), 133.3, 133.2, 130.1, 129.9, 129.8 (3C), 129.7, 129.6, 129.1, 128.8, 128.7, 128.6 (3C), 128.5, 128.4 (2C), 128.3 (2C), 128.1, 128.0, 127.9 (3C), 127.7, 127.6 (2C), 127.5, 127.4, 127.3, 126.9, 126.5 (35 × C-Ar), 101.4 (C-1″), 100.8 (C-1‴), 99.5 (C-1′), 97.7 (C-1), 80.4 (C-3′), 79.9 (C-2′), 78.3 (C-4′), 77.8 (C-4), 77.4 (C-5)*, 76.0 (C-4″), 75.3 (C-2), 74.8 (CH_Bn_), 74.0 (CH_Bn_), 73.5 (CH_Bn_), 73.2 (2C, C-3″, C-5″), 73.0 (2C, CH_Bn_, C-3‴), 72.6 (2C, CH_Bn_, C-2″),72.4 (C-5‴), 71.9 (C-2‴), 71.0 (C-5′), 69.6 (C-3), 69.5 (C-4‴), 68.5 (C-6), 68.1/68.0 (CH_Linker_), 67.3/67.2 (CH_Cbz_), 62.7 (C-6″/C-6‴), 62.5 (C-6″/C-6‴), 60.5 (C-6′), 50.6/50.3 (NCH_Bn_), 47.2/46.3 (CH_Linker_), 29.2/29.1 (CH_Linker_), 28.0/27.6 (CH_Linker_), 23.5 (CH_Linker_). Due to signal overlap, 86 out of 128 carbon atoms were assigned. *Assigned by HSQC due to signal superimposition with solvent peak. ^1^H-^13^C-coupled HSQC (CDCl_3_) *J*_C1,H1_ = 169 Hz, *J*_C1′H1′_ = 168 Hz, *J*_C1″,H1″_ = 160 Hz, *J*_C1‴,H1‴_ = 162 Hz. MALDI-TOF calculated for C_129_H_128_FNO_31_^+^ [M + H + MeOH]^+^: 2187.85; found: 2188.76.

*N*-(Benzyl)-benzyloxycarbonyl-5-aminopentyl-(2,3,4,6-tetra-*O*-benzoyl-β-D-glucopyranosyl)-(1→4)-(2,3,6-tri-*O*-benzoyl-β-D-glucopyranosyl)-(1→4)-(2,3-di-*O*-benzyl-6-deoxy-6-fluoro-α-D-glucopyranosyl)-(1→4)-2,3,6-tri-*O*-benzyl-α-D-galactopyranoside (**48**). In a microwave vessel equipped with a stirring bar, **47** (140 mg, 69.6 μmol, 1.0 eq.) was dissolved in dry CH_2_Cl_2_ (2 mL). Subsequently, 2,4,6-collidine (28.0 μL, 0.21 mmol, 3.0 eq.) and DAST (14.0 μL, 0.10 mmol, 1.5 eq.) were added. The reaction solution was heated for 1 h in a microwave oven (80 °C, 100 W). Since the reaction was not deemed complete by TLC, another portion of 2,4,6-collidine (28.0 μL, 0.21 mmol, 3.0 eq.) and DAST (14.0 μL, 0.10 mmol, 1.5 eq.) were added, and microwave heating was continued for another 1 h. Then, the reaction was cooled to 0 °C and stopped by the addition of MeOH (2 mL). The organic solvents were removed under reduced pressure. The crude residue was dissolved in CH_2_Cl_2_, and the solution was washed with sat. aq. NaHCO_3_ solution (10 mL) and brine (10 mL) and was dried with MgSO_4_. The crude product was subjected to column chromatography (*^c^*Hex/EtOAc *v*/*v* = 4:1) to obtain **48** (112 mg, 51.9 μmol, 75%) as a colorless foam. *Rf* = 0.43 (*^c^*Hex/EtOAc *v*/*v* = 2:1). RP HPLC (Luna, 0.1% TFA; 0 min 50% B → 10 min 100% B, flow: 1 mL/min): *t_R_* = 24.07 min, λ = 230 nm. ${\left[\alpha \right]}_{D}^{24}$ = +30.6° (*c* = 0.3, CHCl_3_). ^1^H NMR (800 MHz, CDCl3) δ = 7.97–7.94 (m, 4H, Ar-H), 7.93–7.90 (m, 4H, Ar-H), 7.81–7.79 (m, 2H, Ar-H), 7.75–7.71 (m, 4H, Ar-H), 7.53–7.49 (m, 2H, Ar-H), 7.49–7.09 (m, 51H, Ar-H), 6.98 (t, *J*_CH,CH_ = 7.6 Hz, 2H, Ar-H), 6.87 (t, *J*_CH,CH_ = 7.3 Hz, 1H, Ar-H), 5.69 (t, *J*_H3″,H4″_ = *J*_H3″,H2″_ = 9.5 Hz, 1H, H-3″), 5.64–5.58 (m, 1H, H-3‴), 5.51–5.46 (m, 1H, H-2″), 5.45–5.42 (m, 1H, H-2‴), 5.28 (t, *J*_H4‴,H3‴_ = *J*_H4‴,H5‴_ = 9.1 Hz, 1H, H-4‴), 5.15 (d, *J*_CH,CH_ = 19.1 Hz, 2H, CH_Cbz_), 5.05 (d, *J*_CH,CH_ = 11.5 Hz, 1H, CH_Bn_), 4.95 (d, *J*_H1″,H2″_ = 8.0 Hz, 1H, H-1″), 4.88 (d, *J*_H1′,H2′_ = 3.6 Hz, 1H, H-1′), 4.83 (d, *J*_CH,CH_ = 11.6 Hz, 1H, CH_Bn_), 4.76 (d, *J*_H1‴,H2‴_ = 7.9 Hz, 1H, H-1‴), 4.64 (d, *J*_CH,CH_ = 12.3 Hz, 1H, CH_Bn_), 4.61–4.58 (m, 2H, H-1, CH_Bn_), 4.53–4.49 (m, 2H, 2 × CH_Bn_), 4.45 (d, *J*_CH,CH_ = 25.2 Hz, 2H, NCH_Bn_), 4.41 (d, *J*_CH,CH_ = 12.0 Hz, 1H, CH_Bn_), 4.39–4.36 (m, 1H, CH_Bn_), 4.28–4.16 (m, 5H, 2 × CH_Bn_, H-6a′, H-4″, H-6a″), 4.13 (dd, *J*_H6b″,H6a″_ = 12.0 Hz, *J*_H6b″,H5″_ = 4.0 Hz, 1H, H-6b″), 4.07 (dd, *J*_H5′,F_ = 34.1 Hz, *J*_H5′,H4_ = 10.3 Hz, 1H, H-5′), 3.97–3.94 (m, 2H, H-6a‴, H-4), 3.90 (t, *J*_H3′,H4′_ = *J*_H3′,H2′_ = 9.3 Hz, 1H, H-3′), 3.85–3.68 (m, 6H, H-6b′, H-4′, H-2, H-3, H-5, H-6a), 3.66–3.62 (m, 2H, H-5‴, H-6b‴), 3.53 (ddd, *J*_H5″,H4″_ = 9.9, *J*_H5″,H6b″_ = 3.9, *J*_H5″,H6a″_ = 1.8 Hz, 1H, H-5″), 3.50–3.41 (m, 1H, CH_Linker_), 3.41–3.35 (m, 2H, H-2′, H-6b), 3.35–3.26 (m, 1H, CH_Linker_), 3.23–3.16 (m, 1H, CH_Linker_), 3.15–3.10 (m, 1H, CH_Linker_), 1.60–1.40 (m, 4H, 4 × CH_Linker_), 1.31–1.11 (m, 2H, 2 × CH_Linker_). ^13^C NMR (200 MHz, CDCl3) δ = 165.8 (2C, 2 × C=O), 165.7 (C=O), 165.5 (C=O), 165.3 (C=O), 165.1 (C=O), 164.7 (C=O), 156.8/156.3 (C=O_Cbz_), 139.3, 138.6, 138.3 (2C), 138.2 (2C), 138.1, 138.03, 137.0/136.9 (9 × Cq), 133.5 (2C), 133.3, 133.2, 130.1, 129.9, 129.8 (2C), 129.7, 129.6, 129.1, 128.8, 128.7 (2C), 128.6 (2C), 128.5, 128.4 (3C), 128.3, 128.1, 128.0 (2C), 127.7 (2C), 127.6, 127.5, 127.4, 127.3, 126.9, 126.2 (32 × C-Ar), 101.6 (C-1″), 100.9 (C-1‴), 99.5 (C-1′), 97.7 (C-1), 81.0 (d, *J*_C6′,F_ = 170.9 Hz, C-6′), 80.4 (C-3′), 79.7 (C-2′), 77.7 (d, *J*_C4′,F_ = 3.8 Hz, C-4′), 77.2 (C-3*), 77.1 (C-4*), 76.0 (C-4″), 75.1 (C-2), 74.6 (CH_Bn_), 74.1 (CH_Bn_), 73.3 (2C, C-5″, CH_Bn_), 73.1 (2C, 2 × CH_Bn_),73.0 (C-3″), 72.8 (C-3‴), 72.6 (C-2″), 72.4 (C-5‴), 71.9 (C-2‴), 69.9 (d, *J*_C5′,F_ = 16.3 Hz, C-5′), 69.6 (C-4‴), 69.3 (C-5), 68.1/68.0 (CH_Linker_), 67.9 (C-6), 67.3 (CH_Cbz_), 62.7 (C-6‴), 62.4 (C-6″), 50.6/50.3 (NCH_Bn_), 47.3/46.3 (CH_Linker_), 29.2/29.1 (CH_Linker_), 28.1/27.6 (CH_Linker_), 23.5 (CH_Linker_). Due to signal overlap, 85 out of 128 carbon atoms were assigned. *Assigned by HSQC-Spectrum due to signal superimposition with solvent peak. ^19^F NMR (377 MHz, CDCl3) δ = −234.9 – −235.4 (m). ^1^H, ^13^C-coupled HSQC (CDCl_3_) *J*_C1,H1_ = 169 Hz, *J*_C1′H1′_ = 170 Hz, *J*_C1″,H1″_ = 160 Hz, *J*_C1‴,H1‴_ = 158 Hz. MALDI-TOF calculated for C_128_H_122_FNO_29_Na^+^ [M + Na]^+^: 2179.80; found: 2179.60.

5-Aminopentyl-(β-D-glucopyranosyl)-(1→4)-(β-D-glucopyranosyl)-(1→4)-(6-deoxy-6-fluoro-α-D-gluco-pryranosyl)-(1→4)-α-D-galactopyranoside (**8**). To a stirred solution of **48** (46 mg, 21.3 μmol, 1.0 eq.) in MeOH/THF (*v*/*v* = 1:1, 8 mL), NaOMe (640 μL, 0.5 M in MeOH) was added. The reaction solution was stirred for 18 h at ambient temperature before being neutralized by the addition of Amberlite^®^ IR120. The reaction mixture was filtered, and the solvents were removed under reduced pressure. The crude residue was dried for 17 h under high vacuo before it was dissolved in a mixture of MeOH/THF/H_2_O/AcOH (*v*/*v* = 19:5:4:1, 20 mL), and Pd/C (40 mg) was added under Ar atmosphere. The reaction was purged three times with H_2_ and stirred for 48 h at ambient temperature. The catalyst was filtered off by Celite^®^ Hyflo Supercel, and the solvents were removed under reduced pressure. The crude product was dissolved in H_2_O/MeOH (*v*/*v* = 4:1) and subjected to RP column chromatography (H_2_O/MeOH, *v*/*v* = 4:1) to obtain **8** (15.0 mg, 19.9 μmol, 93% over two steps) after lyophilization. ^1^H NMR (800 MHz, DMSO-d_6_) δ = 4.88–4.78 (m, 2H, H-1′, H-6a′), 4.64 (d, *J*_H1,H2_ = 3.6 Hz, 1H, H-1), 4.52–4.40 (m, 2H, H-6b′, H-5′), 4.25 (d, *J*_H1‴,H2‴_ = 7.9 Hz, 1H, H-1‴), 4.22 (d, *J*_H1″,H2″_ = 7.9 Hz, 1H, H-1″), 3.86 (d, *J*_H4,H3_ = 3.2 Hz, 1H, H-4), 3.78 (d, *J*_H6a″,H6b″_ = 11.3 Hz, 1H, H-6a″), 3.75 (t, *J*_H6a,H6b_ = *J*_H6a,H5_ = 9.4 Hz, 1H, H-6a), 3.70–3.64 (m, 2H, H-3, H-6a‴), 3.62 (t, *J*_H5,H6a_ = *J*_H5,H6b_ = 7.1 Hz, 1H, H-5), 3.60–3.54 (m, 4H, H-6b″, CH_Linker_, H-2, H-3′), 3.46–3.39 (m, 2H, H-6b, H-6b‴), 3.38–3.28 (m, 5H, H-3″, CH_Linker_, H-4′, H-4″, H-5″), 3.27 (dd, *J*_H2′,H3′_ = 9.6 Hz, *J*_H1′,H2′_ = 3.6 Hz, 1H, H-2′), 3.19–3.14 (m, 2H, H-3‴, H-5‴), 3.08–3.03 (m, 2H, H-4‴, H-2″), 2.98 (t, *J*_H2″,H1″_ = *J*_H2″,H3″_ = 8.5 Hz, 1H, H-2″), 2.62 (s, 2H, 2 × CH_Linker_), 1.56–1.42 (m, 4H, 4 × CH_Linker_), 1.35 (q, *J*_CH,CH_ = 7.5 Hz, 2H, 2 × CH_Linker_). ^13^C NMR (200 MHz, DMSO-d_6_) δ =103.2 (C-1‴), 103.1 (C-1″), 99.1 (C-1′), 99.0 (C-1), 81.8 (d, *J*_C6′,F_ = 167.7 Hz, C-6′), 79.5 (C-4″), 79.4 (d, *J*_C4′,F_ = 5.2 Hz, C-4′), 76.9 (C-5‴), 76.4 (C-4), 76.3 (C-3‴), 74.9 (C-3″/C-5″), 74.8 (C-3″/C-5″), 73.3 (C-2‴), 73.0 (C-2″), 71.9 (C-2′), 71.3 (C-3′), 71.0 (C-5), 70.0 (C-4‴), 68.5 − 68.3 (3C, C-2, C-3, C-5′), 67.0 (CH_Linker_), 61.0 (C-6‴), 60.3 (C-6″), 58.7 (C-6), 40.0 (CH_Linker_), 29.8/28.8 (CH_Linker_), 24.1/24.0 (CH_Linker_), 23.0 (CH_Linker_). ^19^F-NMR (377 MHz, DMSO-d_6_) δ = -235.0 (td, *J*_F,H6a′_ = *J*_F,H6b′_ = 47.6 Hz, *J*_F,H5′_ = 33.3 Hz). ^1^H-^13^C-coupled HSQC (DMSO-d_6_) *J*_C1,H1_ = 167 Hz, *J*_C1′H1′_ = 169 Hz, *J*_C1″,H1″_ = 160 Hz, *J*_C1‴,H1‴_ = 161 Hz. HRMS (ESI^+^) calculated for C_29_H_53_FNO_20_^+^ [M + H]^+^: 754.3139; found: 754.3142.

## 4. Conclusions

In summary, practical synthetic routes were developed for the assembly of a panel of seven novel C6-fluorinated mimic epitopes from serotype 8 CPS fragments of *S. pneumoniae*. Fluorinated trisaccharide compounds **3**–**5** were designed based on a known native minimal protective epitope of ST 8, whereas the fluorinated tetrasaccharides **6**–**9** were based on a chemically modified CPS structure in which the terminal glucuronic acid unit was exchanged for a glucose moiety. Recent years have seen the fluorination of epitopes as a promising approach to overcome two major disadvantages of carbohydrate antigens—their low immunogenicity and fast degradation in vivo. Therefore, we envision that the compounds presented herein will be useful tools for evaluating the impact of F-modification on epitope binding by glycan-specific monoclonal antibodies at the cellular level (e.g., via SPR or glycan microarray) as well as for identifying most promising glycotope mimetics for efficient vaccine development. The synthetic routes towards the fluorinated linker-functionalized target compounds are robust and, in some cases (**4** and **8**), involve late fluorination steps of oligosaccharide precursors, which have only been rarely described in the literature. Furthermore, the desired compounds were obtained in sufficient quantities (15–30 mg) for subsequent conjugation steps.

## Data Availability

All data to support the conclusions of this manuscript is contained within this article and the Supporting Information (SI).

## Supplementary Materials

The following supporting information (experimental procedures, NMR spectra of compounds) can be downloaded at: https://www.mdpi.com/article/10.3390/ijms26041535/s1.
