# Supplementary material for: Synthesis of Fluorinated Glycotope Mimetics Derived from Streptococcus pneumoniae Serotype 8 CPS
Source: Int J Mol Sci. 2025 Feb 12;26(4):1535. doi: 10.3390/ijms26041535 (PMC11855009; doi:10.3390/ijms26041535)
Supplement: Supplementary file 1 [file ijms-26-01535-s001.zip › Supporting Information S1_IJMolSci_SP8_AHR_revised.pdf]

Supporting Information S1

Synthesis of fluorinated glycotope mimetics derived from *Streptococcus pneumoniae* serotype 8 CPS

Daniel Gast, Sebastian Neidig, Maximilian Reindl and Anja Hoffmann-Röder*

Department of Chemistry, Ludwig-Maximilians-Universität München, Butenandtstrasse 5-13, Haus F, 81377 Munich, Germany; anja.hoffmann-roeder@cup.lmu.de

Experimental Data

Table of contents

Material and Methods 2

Synthesis of galactosyl acceptor 18 4

Synthesis of fluorinated galactosyl acceptor 19 7

Synthesis of disaccharide acceptors 10, 11 and 12 12

Synthesis of cellobiosyl donor 13 18

Synthesis of glucosyl acceptor 20 and fluorinated glucosyl building block 17 22

Synthesis of fluorinated glucosyl building block 14........ 27

Synthesis of fluorinated cellobiosyl donor 15 32

Material and Methods

If not otherwise noted, all reactions were magnetically stirred and conducted in oven-dried glassware. Moisture sensitive reactions were performed in an argon atmosphere using standard Schlenk techniques. Solvents for moisture sensitive reactions (diethyl ether, dichloromethane, tetrahydrofuran) were dried according to standard procedures and distilled prior to use or purchased from Acros Organics (Geel, Belgium) as "extra dry" reagents (*N*,*N*-dimethylformamide). Commercially available reagents were purchased from Acros Organics (Geel, Belgium) Sigma-Aldrich and Merck KGaA (both Darmstadt, Germany) as well as from TCI Deutschland GmbH (Eschborn, Germany). Reagents were used without further purification. Analytical thin-layer chromatography (TLC) was used for monitoring reactions. TLC was performed on pre-coated silica gel 60 F_{254} aluminum plates (Merck KGaA, Darmstadt, Germany) and visualized by exposure to ultraviolet light (UV, 254 nm) and/or staining with a 1:1 mixture of 1 M H_2SO_4 in EtOH and 3 % 4-methoxyphenol solution in EtOH. Alternatively, staining was performed with Seebach reagent [Cerium phosphomolybdic acid (5.0 g), conc. H_2SO_4 (16mL), water (200 mL) and Cerium(IV) sulfate (2.0 g)]. If not stated otherwise, purification of substances was achieved by standard flash column chromatography on silica (35-70 µm particle size) from Acros Organics (Geel, Belgium). Amberlite® IR120 and Celite® Hyflo Supercel were purchased from Merck KGaA (Darmstadt, Germany).

Microwave-assisted syntheses were carried out in a Discover microwave from CEM GmbH (Kamp-Lintfort, Germany). The corresponding reaction conditions were noted in the respective experiments. Analytical RP-HPLC was performed using a JASCO system (PU-2080 Plus, LG-2080-02-S, DG-2080-53 and MD-2010 Plus, JASCO Deutschland GmbH, Pfungstadt, Germany) on a *Phenomenex Luna* column (C18, 5 µm, 250 mm × 4.6 mm;). As eluent. a gradient of water (A) and acetonitrile (B) containing 0.1 % TFA with a flow rate of 1 ml/min was applied. NMR spectra (^{1}H, ^{13}C, ^{19}F, 2D NMR) were recorded on *Varian* 400 MHz and 600 MHz spectrometer or on a *Bruker Avance III* 800 MHz spectrometer equipped with a *CyroProbe™* (Bruker Corporation, Billerica, MA, USA). The chemical shifts are indicated in parts per million (ppm) and relative to the signal of the deuterated solvent. As abbreviations to denote the multiplicities are applied: s (singlet), d (doublet), t (triplett), q (quartett) and m (multiplet). The assignment of proton and carbon signals was accomplished by additional COSY, HSQC and HMBC experiments. Anomeric configurations were verified with proton-coupled HSQC

experiments when required. High resolution (HR-ESI) mass spectra were recorded on a *Thermo Finnigan LTQ FT* spectrometer (Thermo Fisher Scientific, Waltham, MA, USA) either in positive or negative ionization mode. MALDI-TOF spectra were recorded on a *Bruker Daltonics Autoflex II* Time-of-flight spectrometer (Bruker Corporation, Billerica, MA, USA) equipped with a N_2-laser (λ = 337 nm). Optical rotations were measured on a *Perkin-Elmer* polarimeter 241 (PerkinElmer, Inc., Waltham, MA, USA) at the Sodium-D-line (589 nm) and at the given temperature in °C. Concentrations c are given in g/100 ml and the solvents used are stated in brackets ($CHCl_3$).

Synthesis of galactosyl acceptor 18

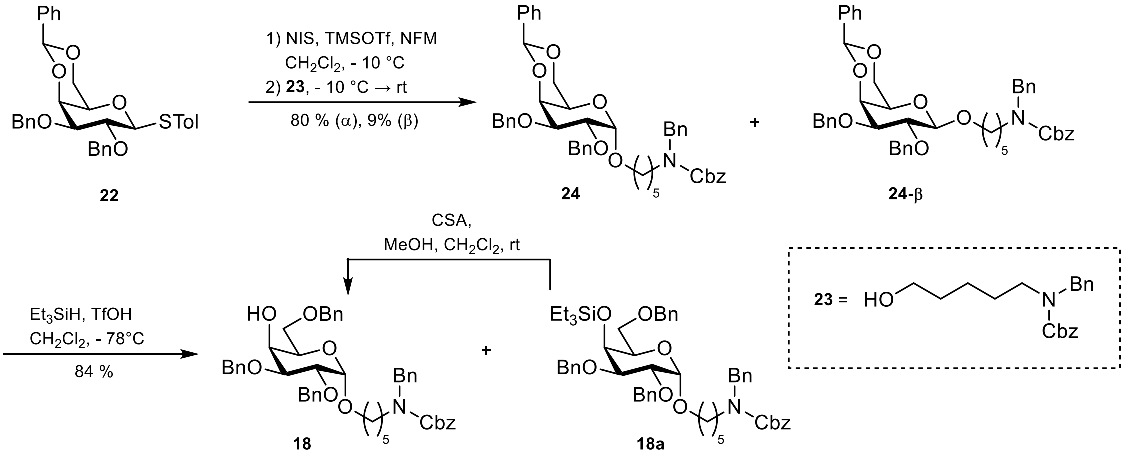

Figure S1: Synthesis of galactosyl acceptor **18** from precursor **24**.

N-(Benzyl)-benzyloxycarbonyl-5-aminopentyl-2,3-di-*O*-benzyl-4,6-*O*-benzylidene-*α*-D-galactopyranoside (24)

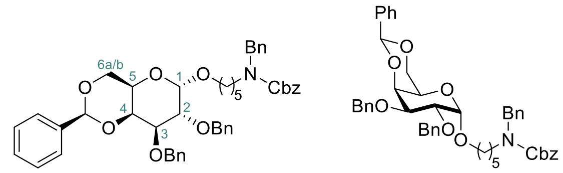

Diethyl ether directed glycosylation:

Thioglycoside donor **22** (1.00 g, 1.80 mmol, 1.0 eq.) and linker **23** (766 mg, 2.34 mmol, 1.3 eq.) were combined and co-evaporated with toluene (2 × 20 ml) and dried under high vacuo for 1 h. Subsequently, the starting materials were dissolved in a mixture of Et_2O/CH_2Cl_2 (v/v = 3:1, 60 ml) and freshly activated MS 4 Å were added. The mixture was stirred for 1 h at ambient temperature and cooled to -10 °C before NIS (511 mg, 2.34 mmol, 1.3 eq.) and TMSOTf (414 µl, 2.34 mmol, 1.3 eq.) were added. After TLC monitoring indicated complete conversion of galactosyl donor **22**, the reaction was neutralized by addition of NEt_3 (1.5 ml) and filtered through a pad of Celite® Hyflo Supercel. The organic layer was washed with sat. aq. $Na_2S_2O_3$ (2 × 25 ml), sat. aq. $NaHCO_3$ (25 ml) and brine (15 ml) and was dried with $MgSO_4$. Organic solvents were removed under reduced pressure and the crude residue was subjected to column flash chromatography (^{c}Hex/EtOAc *v*/*v* = 5:1 → 3:1) to obtain **24** (150 mg, 0.20 mmol, 11 %) and the corresponding β-anomer **24-β** (1.00 g, 1.31 mmol, 73 %) as colorless oils.

NFM-modulated glycosylation:

Donor **22** (2.00 g, 3.60 mmol, 1.0 eq.) was co-evaporated with toluene (2 ×25 ml) and dried for 1 h under high vacuo before being dissolved in dry CH_2Cl_2 (50 ml). *N*-formyl morpholine (5.78 mL, 57.6 mmol, 16 eq.; stored 17 h over freshly activated MS 4 Å prior to use) and freshly activated 4 Å MS were added and the resulting mixture was stirred for 1 h at ambient temperature. *N*-Iodosuccinimide (1.21 g, 5.40 mmol, 1.5 eq.) was added and the reaction mixture was cooled to -20 °C. Subsequently, TMSOTf (976 µL, 5.40 mmol, 1.5 eq.) was added dropwise over 10 min and the reaction mixture was allowed to warm to -10 °C. After the complete conversion of the starting material to the NFM-imidinium adduct was observed, the amino pentanol linker **23** (2.12 g, 6.49 mmol, 1.8 eq.; co-evaporated with toluene and dried under high vacuo prior to use) was dissolved in dry CH_2Cl_2 and slowly added over 0.5 h. The reaction mixture was stirred at -10 °C until the TLC monitoring indicated complete conversion of the imidinium adduct. The reaction was stopped by the addition of NEt_3 (3 ml), diluted with CH_2Cl_2 and filtered through a pad of Celite® Hyflo Supercel. The filtrate was washed with sat. aq. $Na_2S_2O_3$ (2 × 25 ml) and brine (15 ml). The combined organic layers were dried with $MgSO_4$ and concentrated under reduced pressure. The crude product was subjected to column flash chromatography (^{c}Hex/EtOAc *v*/*v* = 1:0 → 6:1) to obtain **24** (2.19 g, 2.89 mmol, 80 %) and the corresponding **24-β** (261 mg, 0.34 mmol, 9 %) as colorless oils.

24: R*f* = 0.43 (^{c}Hex/EtOAc *v*/*v* = 3:1); **RP HPLC** (Luna, 0.1 % TFA; 0 min 50 % B → 10 min 100 % B, flow: 1 ml/min): t_R = 14.72, λ = 230 nm; $[\alpha]_D^{22}$ = + 60.8 ° (c = 0.33; $CHCl_3$); **^{1}H NMR** (800 MHz, $CDCl_3$): δ [ppm] = 7.54 – 7.52 (m, 2H, Ar-H), 7.40 (d, $J_{CH,CH}$ = 7.5 Hz, 2H, Ar-H), 7.38 – 7.23 (m, 20H, Ar-H), 7.17 (d, $J_{CH,CH}$ = 7.4 Hz, 1H, Ar-H), 5.47 (s, 1H, CH-Ar), 5.18 (d, $J_{CH,CH}$ = 25.5 Hz, 2H, CH_{Cbz}), 4.88 – 4.84 (m, 2H, H-1, CH_{Bn}), 4.81 (d, $J_{CH,CH}$ = 12.1 Hz, 1H, CH_{Bn}), 4.73 (d, $J_{CH,CH}$ = 12.2 Hz, 1H, CH_{Bn}), 4.66 – 4.62 (m, 1H, CH_{Bn}), 4.52 – 4.46 (m, 2H, NCH_{Bn}), 4.21 – 4.15 (m, 2H, H-6a, H-4), 4.05 (dd, $J_{H2,H3}$ = 10.1 Hz, $J_{H2,H1}$ = 3.6 Hz, 1H, H-2), 4.02 – 3.95 (m, 2H, H-3, H-6b), 3.64 – 3.52 (m, 2H, H-5, CH_{Linker}), 3.44 – 3.35 (m, 1H, CH_{Linker}), 3.30 – 3.17 (m, 2H, 2 × CH_{Linker}), 1.66 – 1.47 (m, 4H, 4 × CH_{Linker}), 1.35 – 1.21 (m, 2H, 2 × CH_{Linker}); **^{13}C NMR** (200 MHz, $CDCl_3$): δ [ppm] = 156.9/156.3 (C=O-Cbz), 139.0, 138.9, 138.0 (2C),137.0/ 136.9 (5 × Cq), 129.0, 128.7, 128.6 (2C), 128.4, 128.2, 128.1, 128.0 (2C), 127.7, 127.6, 127.5, 127.4, 127.3, 126.5 (15 × C-Ar), 101.2 (CH-Ar), 98.3 (C-1), 76.3 (C-3), 75.8 (C-2), 74.9 (C-4), 73.7 (CH_{Bn}), 72.2 (CH_{Bn}), 69.6 (C-6), 68.3 (CH_{Linker}), 67.3 (CH_{Cbz}), 62.7 (C-5), 50.6/50.3 (N-CH_{Bn}), 47.3/46.3 (CH_{Linker}), 29.3 (CH_{Linker}), 28.1/27.7 (CH_{Linker}), 23.6 (CH_{Linker}); Due to signal overlap, 37 out of 47 C atoms were assigned;

^{1}H-^{13}C-coupled HSQC ($CDCl_3$): $J_{H1,C1}$ = 169 Hz; **HRMS** (ESI$^+$): Calculated for $C_{47}H_{51}O_8NNa^+$ $[M+Na]^+$: 780.3507; found: 780.3493.

24-β: R$_f$ = 0.18 (^{c}Hex/EtOAc *v*/*v* = 3:1); **RP HPLC** (Luna, 0.1 % TFA; 0 min 50 % B → 10 min 100 % B, flow: 1 ml/min): t_R = 14.60, λ = 230 nm; **^{1}H NMR** (600 MHz, $CDCl_3$): δ [ppm] = 7.57 – 7.54 (m, 2H, Ar-H), 7.40 – 7.12 (m, 23H), 5.49 (s, 1H, CH-Ar), 5.16 (d, $J_{CH,CH}$ = 17.7 Hz, 2H, CH_{Cbz}), 4.92 – 4.73 (m, 4H, 4 × CH_{Bn}), 4.47 (d, $J_{CH,CH}$ = 16.5 Hz, 2H, NCH_{Bn}), 4.37 – 4.31 (m, 1H, H-1), 4.28 (d, $J_{H6a,H6b}$ = 12.3 Hz, 1H, H-6a), 4.10 (d, $J_{H4,H3}$ = 3.7 Hz, 1H, H-4), 4.00 (dd, $J_{H6b,H6a}$ = 12.3 Hz, $J_{H6b,H5}$ = 1.8 Hz, 1H, H-6b), 3.97 – 3.88 (m, 1H, CH_{Linker}), 3.82 (dd, $J_{H2,H3}$ = 9.7 Hz, $J_{H2,H1}$ = 7.8 Hz, 1H, H-2), 3.54 (dd, $J_{H3,H2}$ = 9.7 Hz, $J_{H3,H4}$ = 3.7 Hz, 1H, H-3), 3.49 – 3.39 (m, 1H, CH_{Linker}), 3.28 (s, 1H, H-5), 3.25 – 3.13 (m, 2H, 2 × CH_{Linker}), 1.72 – 1.46 (m, 4H, 4 × CH_{Linker}), 1.41 – 1.26 (m, 2H, 2 × CH_{Linker}); **^{13}C NMR** (150 MHz, $CDCl_3$): δ [ppm] = 156.8/156.3 (C=O-Cbz), 139.0, 138.6, 138.0 (2C), 137.0/136.9 (5 × Cq), 129.0, 128.6 (2C), 128.5, 128.4 (2C), 128.2, 128.0, 127.9, 127.8 (3C), 127.4, 127.3, 126.6 (15 × C_{Ar}), 103.8 (C-1), 101.4 (CH-Ar), 79.3 (C-3), 78.6 (C-2), 75.3 (CH_{Bn}), 74.1 (C-4), 72.1 (CH_{Bn}), 69.8/69.7 (CH_{Linker}), 69.4 (C-6), 67.2 (CH_{Cbz}), 66.5 (C-5), 50.7/50.3 (NCH_{Bn}), 47.3/46.3 (CH_{Linker}), 29.5 (CH_{Linker}), 28.1/27.7 (CH_{Linker}), 23.5 (CH_{Linker}); Due to signal overlap 37 out of 47 C atoms were assigned; **^{1}H-^{13}C-coupled HSQC** ($CDCl_3$): $J_{H1,C1}$ = 159 Hz; **HRMS** (ESI$^+$): Calculated for $C_{47}H_{55}O_8N_2^+$ $[M+NH_4]^+$: 775.3953; found: 775.3962.

N-(Benzyl)-benzyloxycarbonyl-5-aminopentyl-2,3,6-tri-*O*-benzyl-α-D-galactopyranoside (18)

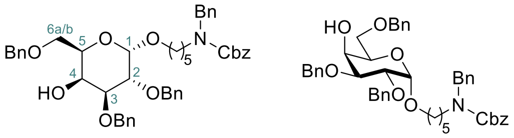

To a stirred solution of **24-β** (1.75 g, 2.31 mmol, 1.0 eq.) in dry CH_2Cl_2 (20 ml) were added freshly activated MS 4 Å and triethyl silane (2.58 ml, 16.2 mmol, 7.0 eq.). The reaction mixture was stirred 0.5 h at room temperature, before being cooled to -78 °C. Subsequently trifluoromethanesulfonic acid (1.23 ml, 13.9 mmol, 6.0 eq.) was added slowly, and the reaction mixture was stirred for 4.5 h. After complete conversion of the starting material was observed by TLC monitoring, NEt_3 (3.0 ml) was added. The mixture was filtered through a pad of Celite® Hyflo Supercel and was concentrated under reduced pressure. The crude product was subjected to column chromatography (^{c}Hex/EtOAc *v*/*v* = 4:1) to afford **18** as well as silyl ether **18a** as side-product. To cleave the silyl ether, compound **18a** was dissolved in MeOH/CH_2Cl_2

(v/v = 1:1, 20 ml) and camphorsulfonic acid (500 mg) was added. The reaction mixture was stirred for 45 min at ambient temperature, before it was neutralized by the addition of NEt_3. The solvents were removed under reduced pressure, and the crude product was subjected to flash chromatography (^{c}Hex/EtOAc *v/v* = 4:1). Products were combined to obtain **18** (1.47 g, 1.93 mmol, 84 %) as a colorless oil.

R*f* = 0.42 (^{c}Hex/EtOAc *v/v* = 2:1); **RP HPLC** (Luna, 0.1 % TFA; 0 min 50 % B → 10 min 100 % B, flow: 1ml/min): t_R = 14.52 min, λ = 230 nm; $[\alpha]_D^{22}$ = + 34.1 ° (c = 0.66; $CHCl_3$); **^{1}H NMR** (600 MHz, $CDCl_3$): δ [ppm] = 7.43 – 7.14 (m, 25H, Ar-H), 5.19 (d, $J_{CH,CH}$ = 16.3 Hz, 2H, CH_{Cbz}), 4.83 – 4.77 (m, 3H, H-1, CH_{Bn}), 4.72 (d, $J_{CH,CH}$ = 11.5 Hz, 1H, CH_{Bn}), 4.65 (d, $J_{CH,CH}$ = 12.2 Hz, 1H, CH_{Bn}), 4.62 – 4.54 (m, 2H, CH_{Bn}), 4.50 (d, $J_{CH,CH}$ = 17.4 Hz, 2H, CH_{Bn}), 4.09 (s, 1H, H-4), 3.93 (bs, 1H, H-5), 3.90 – 3.85 (m, 2H, H-2, H-3), 3.74 (dd, $J_{H6a,H6b}$ = 10.0 Hz, $J_{H6a,H5}$ = 5.5 Hz, 1H, H-6a), 3.67 (dd, $J_{H6b,H6a}$ = 10.0 Hz, $J_{H6b,H5}$ = 6.3 Hz, 1H, H-6b), 3.66 – 3.57 (m, 1H, CH_{Linker}), 3.44 – 3.33 (m, 1H, CH_{Linker}), 3.30-3.17 (m, 2H, CH_{Linker}), 1.69 – 1.48 (m, 4H, CH_{Linker}), 1.40-1.22 (m, 2H, CH_{Linker}); **^{13}C NMR** (150 MHz, $CDCl_3$): δ [ppm] = 156.8/156.3 (C=O-Cbz), 138.7, 138.4, 138.2, 138.1, 137.0/136.9 (5 × Cq), 128.7, 128.6, 128.5 (2C), 128.0 (2C), 127.9 (3C), 127.8 (3C), 127.4 (2C), 127.3 (25 × C_{Ar}), 97.5 (C-1), 77.8 (C-2/C-3), 76.0 (C-2/C-3), 73.7 (CH_{Bn}), 73.4 (CH_{Bn}), 72.8 (CH_{Bn}), 69.7 (C-6), 68.6 (C-5), 68.1 (2C, C-4, CH_{Linker}), 67.3 (CH_{Cbz}), 50.6/50.3 (NCH_{Bn}), 47.3/46.3 (CH_{Linker}), 29.2 (CH_{Linker}), 28.1/27.7 (CH_{Linker}), 23.6 (CH_{Linker}); Due to signal overlap, 37 out of 47 C atoms were assigned; **^{1}H-^{13}C-coupled HSQC** ($CDCl_3$): $J_{H1,C1}$ = 168 Hz; **HRMS** (ESI$^+$): Calculated for $C_{47}H_{57}N_2O_8^+$ $[M+NH_4]^+$: 777.4109; found: 777.4098.

Synthesis of fluorinated galactosyl acceptor 19

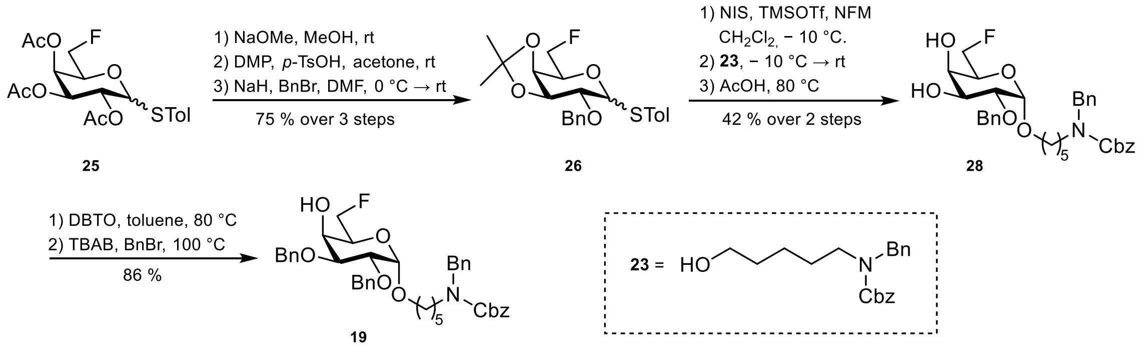

Figure S2: Synthesis of fluorinated galactosyl acceptor **19** from precursor **25**.

4-Methylphenyl-2-*O*-benzyl-3,4-*O*-isopropylidene-6-deoxy-6-fluoro-1-thio-*α*/β-D-galacto-pyranoside (26)

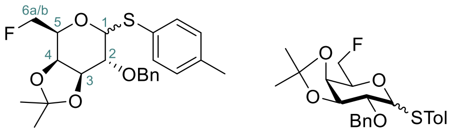

To a stirred solution of **25** (3.10 g, 7.48 mmol, 1.0 eq.) in MeOH (150 ml) were added catalytic amounts of a freshly prepared solution of NaOMe in MeOH. The reaction mixture was stirred for 1 h at ambient temperature, before being neutralized by addition of Amberlite® IR120. The resin was filtered off and organic solvents were removed under reduced pressure to obtain the crude triol.

This triol was subsequently dissolved in acetone (125 ml) and 2,2-dimethoxy propane (9.0 ml, 73.4 mmol, 9.8 eq.) as well as catalytic amounts of *p*-TsOH were added. The reaction mixture was stirred at ambient temperature until the TLC monitoring showed complete conversion of the starting material. The reaction was neutralized by the addition of NEt_3 (1.5 ml) and concentrated under reduced pressure. The crude residue was dissolved in CH_2Cl_2 (100 ml) washed with 1M HCl (50 ml), sat. aq. $NaHCO_3$ (50 ml), and brine before it was dried with $MgSO_4$.

The resulting crude intermediate was dissolved in dry DMF (50 ml) and cooled to 0 °C, before NaH (60 % in mineral oil, 750 mg, 18.7 mmol, 2.5 eq.) was added portion wise. The reaction mixture was warmed to ambient temperature and stirred for 10 min, before benzyl bromide (1.60 ml, 13.5 mmol, 1.8 eq.) was added. The reaction was stirred for another 1 h before sat. aq. NH_4Cl solution (15 ml) was added. The aqueous mixture was concentrated under reduced pressure and the crude residue was dissolved in CH_2Cl_2 and washed with water (20 ml) and brine (25 ml). The organic layer was dried with $MgSO_4$, concentrated and the crude product was subjected to column chromatography (^{c}Hex/EtOAc *v*/*v* = 9:1) to obtain an anomeric mixture of **26** (2.35 g, 5.62 mmol, 75 % over three steps) as a yellow oil.

β-26: R_f = 0.31 (^{c}Hex/EtOAc v/v = 1:1); **RP HPLC** (Luna, 0.1 % TFA; 0 min 50 % B → 10 min 100 % B, flow: 1ml/min): t_R = 12.72 min, λ = 230 nm; **^{1}H NMR** (400 MHz, $CDCl_3$): δ [ppm] = 7.47 – 7.41 (m, 4H, Ar-H), 7.39 – 7.33 (m, 2H, Ar-H), 7.32 – 7.28 (m, 1H, Ar-H), 7.11 (d, $J_{CH,CH}$ = 7.9 Hz, 2H, Ar-H), 4.81 (d, $J_{CH,CH}$ = 11.3 Hz, 1H, CH_{Bn}), 4.75 – 4.65 (m, 2H, CH_{Bn}, H-6a), 4.63 – 4.53 (m, 2H, H-1, H-6b), 4.30 (t, $J_{H3,H2}$ = $J_{H3,H4}$ = 6.0 Hz, 1H, H-3), 4.20 (dd, $J_{H4,H3}$ = 5.8, $J_{H4,H5}$ = 2.1 Hz,

1H, H-4), 4.01 (dddd, $J_{H5,F}$ = 13.7 Hz, $J_{H5,H6a/b}$ = 6.9 Hz, $J_{H5,H6a/b}$ = 4.6 Hz, $J_{H5,H4}$ = 2.1 Hz, 1H, H-5), 3.53 (dd, $J_{H2,H1}$ = 9.2 Hz, $J_{H2,H3}$ = 6.2 Hz, 1H, H-2), 2.33 (s, 3H, Ar-CH_3), 1.41 (s, 3H, CH_3), 1.35 (s, 3H, CH_3); **^{13}C NMR** (100 MHz, $CDCl_3$): δ [ppm] = 137.9 (2C, 2 × Cq), 132.8 (2C), 129.8 (Cq), 129.7 (2C), 128.5 (2C), 128.4 (2C), 127.9 (9 × C-Ar), 110.6 (Cq), 86.7 (C-1), 82.3 (d, $J_{C6,F}$ = 170.1 Hz, C-6), 79.4 (C-3), 78.1 (C-2), 74.7 (d, $J_{C5,F}$ = 22.0 Hz, C-5), 73.6 (CH_{Bn}), 73.1 (d, $J_{C4,F}$ = 6.5 Hz, C-4), 27.7 (CH_3), 26.4 (CH_3), 21.3 (Ar-CH_3); **^{19}F NMR** (377 MHz, $CDCl_3$): δ [ppm] = -228.4 (ddd, $J_{F,H6a}$ = 47.7 Hz $J_{F,H6b}$ = 46.0 Hz, $J_{F,H5}$ = 13.5 Hz).

***α*-26: R*f* =** 0.38 (^{c}Hex/EtOAc v/v = 1:1); **^{1}H NMR** (400 MHz, $CDCl_3$, selected signals): δ [ppm] = 5.58 (d, $J_{H1,H2}$ = 5.2 Hz, 1H, H-1), 4.40 (t, $J_{H3,H2}$ = $J_{H3,H4}$ = 6.2 Hz, 1H, H-3), 3.95 (dd, $J_{H2,H3}$ = 6.3 Hz, $J_{H2,H1}$ = 5.2 Hz, 1H, H-2); **^{13}C NMR** (100 MHz, $CDCl_3$, selected signals): δ [ppm] = 86.5 (C-1), 75.6 (C-2), 74.3 (C-3); **^{19}F NMR** (377 MHz, $CDCl_3$): δ [ppm] = -230.0 (td, $J_{F,H6a}$ = $J_{F,H6b}$ = 47.0 Hz, $J_{F,H5}$ = 13.8 Hz); **HRMS** (ESI$^+$): Calculated for $C_{23}H_{31}O_4NFS^+$ [M+NH_4]$^+$: 436.1953; found: 436.1954.

N-(Benzyl)-benzyloxycarbonyl-5-aminopentyl-2-*O*-benzyl-6-deoxy-6-fluoro-*α*-D-galacto-pyranoside (28)

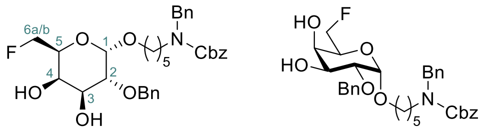

Donor **26** (800 mg, 1.91 mmol, 1.0 eq.) was co-evaporated with dry toluene (20 ml) and dried under high vacuo for 1.5 h. Subsequently, starting material was dissolved in dry CH_2Cl_2 (20 ml) and freshly activated MS 4 Å and NFM (3.10 ml, 30.6 mmol, 16 eq.) were added. The heterogenous mixture was stirred for 1.5 h at ambient temperature before being cooled to −10 °C. After stirring for 0.5 h, NIS (646 mg, 2.87 mmol, 1.5 eq.) and TMSOTf (519 µl, 2.87 mmol, 1.5 eq.) were added. The reaction was stirred at -10 °C for 1 h before another portion of NIS (220 mg, 0.89 mmol, 0.5 eq.) and TMSOTf (150 µl, 0.83 mmol, 0.4 eq.) was added. After the pre-activation of thiodonor **26** was deemed complete, a solution of linker **23** (1.13 g, 3.44 mmol, 1.8 eq.; co-evaporated with toluene and dried under high vacuo prior to use) in dry CH_2Cl_2 (5 ml) was added. The reaction was stepwise warmed to 0 °C and subsequently to ambient temperature. It was stirred for 3 d before it was stopped by the addition of NEt_3 (3 ml). The reaction mixture was diluted with CH_2Cl_2 and filtered through a short pad of Celite® Hyflo Supercel. The organic layer was washed with sat. aq. $Na_2S_2O_3$

(25 ml), 1 M HCl (25 ml) and brine (25 ml) and was dried with $MgSO_4$. The organic solvents were removed under reduced pressure and the crude residue was subjected to flash column chromatography (^{c}Hex/EtOAc *v*/*v* = 5:1). Product containing fractions were pooled and used in the subsequent reaction. The obtained glycosylation product was dissolved in 80 % AcOH (50 ml) and stirred for 2 h at 80 °C. After complete conversion of the starting material, the reaction was concentrated under reduced pressure and co-evaporated with toluene (2 × 50 ml). The crude residue was subjected to column chromatography (^{c}Hex/EtOAc *v*/*v* = 3:1 → 1:1) to obtain pure diol **28** (470 mg, 0.81 mmol, 42 % over two steps) as a colorless oil.

R*f* = 0.13 (^{c}Hex/EtOAc v/v = 3:1); **RP HPLC** (Luna, 0.1 % TFA; 0 min 50 % B → 10 min 100 % B, flow: 1ml/min): t_R = 10.32 min, λ = 230 nm; $[\alpha]_D^{22}$ = + 59.2° (c = 0.5; $CHCl_3$); **^{1}H NMR** (400 MHz, $CDCl_3$): δ [ppm] = 7.38 – 7.12 (m, 15H, Ar-H), 5.17 (d, $J_{CH,CH}$ = 11.5 Hz, 2H, CH_{Cbz}), 4.83 (s, 1H, H-1), 4.65 (d, $J_{H6a,H6b}$ = 6.0 Hz, 1H, H-6a), 4.62 (s, 2H, CH_{Bn}), 4.53 (d, $J_{H6b,H6a}$ = 6.4 Hz, 1H, H-6b), 4.49 (s, $J_{CH,CH}$ = 8.5 Hz, 2H, NCH_{Bn}), 4.01 (bs, 3H, H-3, H-4, H-5), 3.71 (dd, $J_{H2,H3}$ = 9.2 Hz, $J_{H2,H1}$ = 3.5 Hz, 1H, H-2), 3.68 – 3.5 (m, 1H, 1H, CH_{Linker}), 3.38 – 3.10 (m, 3H, 3 × CH_{Linker}), 1.65 – 1.42 (m, 4H, 4 × CH_{Linker}), 1.39 – 1.25 (m, 2H, 2 × CH_{Linker}); **^{13}C NMR** (100 MHz, $CDCl_3$): δ [ppm] = 156.9/156.3 (C=O-Cbz), 138.0 (2C), 137.0/136.8 (3 × Cq), 128.8, 128.7, 128.3, 128.2, 128.1, 128.0, 127.4, 127.3 (8 × C-Ar), 96.6 (C-1), 83.0 (d, $J_{C6,F}$ = 168.3 Hz, C-6), 76.7 (C-2), 72.8 (CH_{Bn}), 68.8 – 68.3 (4C, C3, C4, C5, CH_{Linker}), 67.3 (CH_{Cbz}), 50.6/50.3 (NCH_{Bn}), 47.2/46.3 (CH_{Linker}), 29.3 (CH_{Linker}), 28.0/27.6 (CH_{Linker}), 23.6 (CH_{Linker}). Due to signal overlap, 26 out of 33 C atoms were assigned; **^{19}F NMR** (377 MHz, $CDCl_3$): δ [ppm] = -229.7 – -229.0 (m); **^{1}H-^{13}C-coupled HSQC (**$CDCl_3$): $J_{H1,C1}$ = 171 Hz; **HRMS** (ESI$^+$): Calculated for $C_{33}H_{44}FN_2O_7^+$ $[M+NH_4]^+$: 599.3127; found: 599.3126.

***N*-(Benzyl)-benzyloxycarbonyl-5-aminopentyl-2,3-di-*O*-benzyl-6-deoxy-6-fluoro-*α*-D-galactopyranoside (19)**

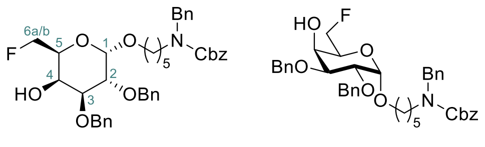

To a stirred solution of compound **28** (400 mg, 0.69 mmol, 1.0 eq.) in dry toluene (25 ml) was added dibutyltin oxide (177 mg, 0.71 mmol, 1.0 eq.) and the reaction was stirred for 5 h at 85 °C. Then, benzyl bromide (178 µl, 1.50 mmol, 2.2 eq.) and TBAB (222 mg, 0.69 mmol, 1.0 eq.) were added and the reaction was heated for further 6 h at 100 °C. After the reaction was

deemed complete by the TLC analysis, solvents were removed under reduced pressure and the crude residue was dissolved in CH_2Cl_2 (50 ml). The organic layer was washed with water (25 ml), sat. aq. $NaHCO_3$ solution (25 ml) and brine and was dried with $MgSO_4$. The solvents were removed under reduced pressure and the crude residue was subjected to flash column chromatography (^{c}Hex/EtOAc *v/v* = 1:0 → 5:1) to afford **24** (397 mg, 0.59 mmol, 86 %) as a colorless oil.

R*f* = 0.66 (^{c}Hex/EtOAc *v/v* = 1:1); **RP HPLC** (Luna**,** 0.1 % TFA; 0 min 50 % B → 10 min 100 % B, flow: 1ml/min): t_R = 13.25 min, λ = 230 nm); $[\alpha]_D^{24}$ = + 41.7° (c = 1.0; $CHCl_3$); **^{1}H NMR** (600 MHz, $CDCl_3$): δ [ppm] = 7.40 – 7.14 (m, 20H, Ar-H), 5.18 (d, $J_{CH,CH}$ = 19.5 Hz, 2H, CH_{Cbz}), 4.83 – 4.76 (m, 3H, H-1, 2 × CH_{Bn}), 4.69 (d, $J_{CH,CH}$ = 11.5 Hz, 1H, CH_{Bn}), 4.65 – 4.60 (m, 2H, H-6a, CH_{Bn}), 4.55 – 4.46 (m, 3H, H-6b, NCH_{Bn}), 4.06 – 3.95 (m, 2H, H-4, H-5), 3.91 – 3.85 (m, 1H, H-3), 3.81 (dd, $J_{H2,H3}$ = 9.7 Hz, $J_{H2,H1}$ = 3.6 Hz, 1H, H-2), 3.67 – 3.56 (m, 1H, CH_{Linker}), 3.43 – 3.33 (m, 1H, CH_{Linker}), 3.29 – 3.16 (m, 2H, 2 × CH_{Linker}), 1.67 – 1.49 (m, 4H, 4 × CH_{Linker}), 1.35 – 1.24 (m, 2H, 2 × CH_{Linker}); **^{13}C NMR** (150 MHz, $CDCl_3$): δ [ppm] = 156.9/156.3 (C=O-Cbz), 138.5, 138.2, 138.1/138.0, 137.1/136.9 (4 × Cq), 128.7, 128.5, 128.1, 128.0, 128.0, 127.9 (6 × C-Ar), 97.4 (C-1), 82.9 (d, $J_{C6,F}$ = 168.3 Hz, C-6), 77.4 (C-3), 75.9 (C-2), 73.4 (CH_{Bn}), 73.1 (CH_{Bn}), 68.4 – 68.3 (2C, CH_{Linker}, C-5,), 67.6 (d, $J_{C4,F}$ = 6.6 Hz, C-4), 67.3 (CH_{Cbz}), 50.6/50.3 (NCH_{Bn}), 47.3/46.3 (CH_{Linker}), 29.2 (CH_{Linker}), 28.1/27.6 (CH_{Linker}), 23.6 (CH_{Linker}); Due to signal overlap, 26 out of 40 C atoms were assigned; **^{19}F NMR** (377 MHz, $CDCl_3$): δ [ppm] = -228.9 (dtd, $J_{F,H6a}$ = 61.3 Hz, $J_{F,H6a}$ = 46.9 Hz, $J_{F,H5}$ = 14.8 Hz); **^{1}H-^{13}C-coupled HSQC (**$CDCl_3$): $J_{H1,C1}$ = 171 Hz; **HRMS** (ESI^+): Calculated for $C_{40}H_{50}FN_2O_7^+$ $[M+NH_4]^+$: 689.3587; found: 689.3593.

Synthesis of disaccharide acceptors 10, 11 and 12

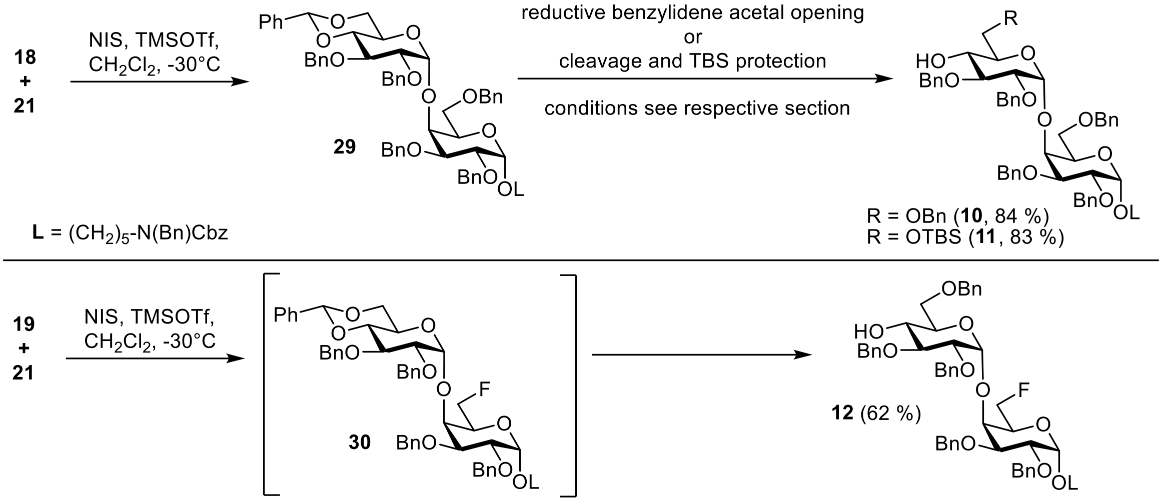

Figure S3: Synthesis of disaccharide acceptor building blocks **10**, **11**, and **12**.

N-(Benzyl)-benzyloxycarbonyl-5-aminopentyl-(2,3-di-*O*-benzyl-4,6-*O*-benzyliden-*α*-D-glucopyranosyl)-(1→4)-2,3,6-tri-*O*-benzyl-*α*-D-galactopyranoside (29)

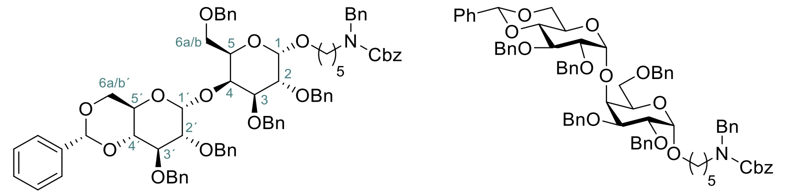

Donor **21** (321 mg, 0.58 mmol, 1.4 eq.) and galactosyl acceptor **18** (310 mg, 0.41 mmol, 1.0 eq.) were combined, co-evaporated with toluene (10 ml) and dried under high vacuo for 1 h. The starting materials were dissolved in dry CH_2Cl_2 (4 ml) and stirred for 1 h with freshly activated MS 4 Å. Subsequently, the reaction mixture was cooled to -30 °C and NIS (191 mg, 0.85 mmol, 1.6 eq.) and TMSOTf (2.30 µl, 12.3 µmol, 0.03 eq.) were added. The reaction was stirred for 1 h at -30 °C before another portion of TMSOTf (2.30 µl, 12.3 µmol, 0.03 eq.) was added. The reaction was stirred for 2 h at -30 °C until a portion of NIS (50 mg, 0.23 mmol, 0.6 eq.) and TMSOTf (2.30 µl, 12.3 µmol, 0.03 eq.) was added, again. After the reaction was deemed complete by the TLC monitoring, it was neutralized by the addition of NEt_3 (100 µl). The mixture was diluted with CH_2Cl_2 and filtered through a pad of Celite® Hyflo Supercel. The organic layer was washed with sat. aq. $Na_2S_2O_3$ (15 ml), 1 M HCl (10 ml), saturated aqueous

$NaHCO_3$ solution (10 ml), and brine (10 ml) and was dried over $MgSO_4$. The crude product was subjected to flash column chromatography (^{c}Hex/EtOAc v/v = 7:1) obtaining **29** (450 mg, 0.38 mmol, 93 %) as a colorless oil.

R*f* = 0.47 (^{c}Hex/EtOAc v/v = 3:1); **RP HPLC** (Luna, 0.1 % TFA; 0 min 50 % B → 10 min 100 % B, flow: 1ml/min): t_R = 20.40 min, λ = 230 nm; $[\boldsymbol{\alpha}]_{\mathbf{D}}^{\mathbf{22}}$ = + 22.7 ° (c = 1.1; $CHCl_3$); **^{1}H NMR** (400 MHz, $CDCl_3$): δ [ppm] = 7.54 – 7.13 (m, 40H, Ar-H), 5.51 (s, 1H, CH-Ar), 5.17 (d, $J_{CH,CH}$ = 9.6 Hz, 2H, CH_{Cbz}), 4.98 – 4.92 (m, 2H, H-1′, CH_{Bn}), 4.90 – 4.84 (m, 2H, 2 × CH_{Bn}), 4.80 – 4.72 (m, 5H, H-1, 4 × CH_{Bn}), 4.70 (d, $J_{CH,CH}$ = 11.9 Hz, 1H, CH_{Bn}), 4.48 (d, $J_{CH,CH}$ = 10.6 Hz, 2H, NCH_{Bn}), 4.31 – 4.19 (m, 3H, H-5′,2 × CH_{Bn}), 4.08 (bs, 1H, H-4), 4.03 (t, $J_{H3',H2'}$ = $J_{H3',H4'}$ = 9.3 Hz, 1H, H-3′), 3.96 – 3.83 (m, 4H, H-2, H-3, H-5, H-6a), 3.73 (dd, $J_{H6a',H6b'}$ = 10.1 Hz, $J_{H6a',H5'}$ = 4.9 Hz, 1H, H-6a′), 3.63 – 3.33 (m, 6H, H-6b′, H-2′, H-4′, H-6b, 2 × CH_{Linker}), 3.29 – 3.14 (m, 2H, 2 × CH_{Linker}), 1.66 – 1.43 (m, 4H, 4 × CH_{Linker}), 1.35 – 1.17 (m, 2H, 2 × CH_{Linker}); **^{13}C NMR** (100 MHz, $CDCl_3$): δ [ppm] = 156.8/156.3 (C=O-Cbz), 139.0, 138.8, 138.6, 138.5, 138.4, 138.1, 137.9, 137.0/136.9 (8 × Cq), 128.9, 128.7, 128.5 (3C), 128.4 (2C), 128.3, 128.2, 128.1, 128.0, 127.8, 127.7 (3C), 127.6, 127.5, 126.2 (18 × C-Ar), 101.2 (CH-Ar), 100.6 (C-1′), 97.9 (C-1), 83.0 (C-4′), 79.7 (C-2′), 79.3 (C-3′), 78.0 (C-2/C-3/C-5), 77.4 (C-4), 75.3 (CH_{Bn}), 74.7 (C-2/C-3/C-5), 74.5 (CH_{Bn}), 73.4 (CH_{Bn}), 73.0 (CH_{Bn}), 73.0 (CH_{Bn}), 69.5 (C-2/C-3/C-5), 69.1 (C-6′), 68.2 (2C, C-6, CH_{Linker}), 67.3 (CH_{Cbz}), 63.1 (C-5′), 50.6/50.3 (NCH_{Bn}), 47.3/46.3 (CH_{Linker}), 29.2 (CH_{Linker}), 28.1/27.7 (CH_{Linker}), 23.6 (CH_{Linker}); Due to signal overlap, 52 out of 74 C atoms were assigned; **^{1}H-^{13}C-coupled HSQC** ($CDCl_3$): $J_{C1,H1}$ = 166 Hz, $J_{C1'H1'}$ = 170 Hz; **HRMS** (ESI$^+$): Calculated for $C_{74}H_{83}O_{13}N_2^+$ $[M+NH_4]^+$:1207.5890; found: 1207.5919.

***N*-(Benzyl)-benzyloxycarbonyl-5-aminopentyl-(2,3,6-tri-*O*-benzyl-*α*-D-glucopyranosyl)-(1→4)-2,3,6-tri-*O*-benzyl-*α*-D-galactopyranoside (10)**

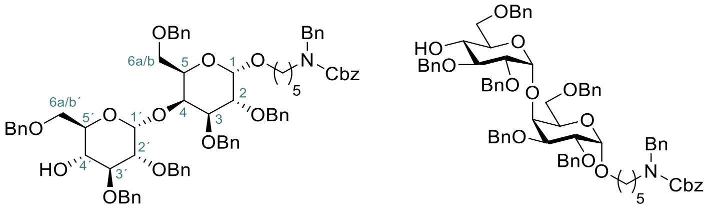

A stirred solution of disaccharide **29** (470 mg, 0.40 mmol, 1.0 eq.) in a mixture of dry MeCN/CH_2Cl_2 (v:v = 3:1, 40 ml) was cooled to -10 °C. Then, borane trimethylamine complex (216 mg, 2.96 mmol, 7.4 eq.) and $BF_3{\cdot}OEt_2$ (375 µl, 2.96 mmol, 7.4 eq.) were added sequentially. The reaction was stirred at -10 °C for 1.5 h before another portion of borane trimethylamine

complex (60 mg, 0.82 mmol, 2.0 eq.) and $BF_3{\cdot}OEt_2$ (104 µl, 0.82 mmol, 2.0 eq.) were added. After the reaction was deemed complete by the TLC monitoring, MeOH (10 ml) and NEt_3 (5 ml) were added, and the mixture was stirred for further 20 min at ambient temperature. The solvents were removed under reduced pressure and the crude product was subjected to column chromatography (^{c}Hex/EtOAc v/v = 7:1 → 5:1 → 3:1) to obtain **10** (376 mg, 0.32 mmol, 80 %) as a colorless oil.

R*f* = 0.35 (^{c}Hex/EtOAc v/v = 3:1); **RP HPLC** (Luna**,** 0.1 % TFA; 0 min 50 % B → 10 min 100 % B, flow: 1ml/min): t_R = 18.50 min, λ = 230 nm; $[\alpha]_D^{24}$ = + 46.7 ° (c = 1.0; $CHCl_3$); **^{1}H NMR** (600 MHz, $CDCl_3$): δ [ppm] = 7.42 – 7.13 (m, 40H, Ar-H), 5.18 (d, $J_{CH,CH}$ = 14.2 Hz, 2H, CH_{Cbz}), 5.01 (d, $J_{H1',H2'}$ = 3.4 Hz, 1H, H-1′), 4.93 (d, $J_{CH,CH}$ = 11.3 Hz, 1H, CH_{Bn}), 4.86 – 4.75 (m, 5H, H-1, 4 × CH_{Bn}), 4.71 – 4.66 (m, 3H, 3 × CH_{Bn}), 4.48 (d, $J_{CH,CH}$ = 15.5 Hz, 2H, NCH_{Bn}), 4.36 (d, $J_{CH,CH}$ = 12.2 Hz, 1H, CH_{Bn}), 4.30 – 4.22 (m, 2H, CH_{Bn}), 4.20 (d, $J_{CH,CH}$ = 12.2 Hz, 1H, CH_{Bn}), 4.17 (dt, $J_{H5',H4'}$ = 9.9 Hz, $J_{H5',H6a'}$ = $J_{H5',H6b'}$ = 3.5 Hz, 1H, H-5′), 4.11 (d, $J_{H4,H3/H5}$ = 2.7 Hz, 1H, H-4), 3.96 – 3.81 (m, 5H, H-2, H-3, H-5, H-3′, H-6a), 3.75 (dd, $J_{H4',H5'}$ = 9.8 Hz, $J_{H4',H3'}$ = 8.9 Hz, 1H, H-4′), 3.62 – 3.48 (m, 3H, H-2′, H-6b, CH_{Linker}), 3.45 – 3.35 (m, 1H, CH_{Linker}), 3.32 (dd, $J_{H6a',H6b'}$ = 10.3 Hz, $J_{H6a',H5'}$ = 3.0 Hz Hz, 1H, H-6a′), 3.28 – 3.15 (m, 2H, 2 × CH_{Linker}), 3.12 (dd, $J_{H6b',H6a'}$ = 10.3 Hz, $J_{H6b',H5'}$ = 4.0 Hz, 1H, H-6b′), 1.68 – 1.46 (m, 4H, 4 × CH_{Linker}), 1.36 – 1.19 (m, 2H, 2 × CH_{Linker}); **^{13}C NMR** (150 MHz, $CDCl_3$): δ [ppm] = 156.8/156.3 (C=O-Cbz), 139.1, 139.0, 138.6, 138.4, 138.3, 138.2, 138.0, 137.0/136.9 (8 × Cq), 128.7, 128.6, 128.5 (2C), 128.4, 128.3, 128.1, 128.0, 127.9, 127.8 (2C), 127.7 (2C), 127.6, 127.5 (2C), 127.3 (17 × C-Ar), 99.8 (C-1′), 97.6 (C-1), 81.8 (C-2/C-3/C-3′/C-5), 80.1 (C-2′), 78.0 (C-2/C-3/C-3′/C-5), 76.6 (C-4), 75.4 (2C, CH_{Bn}, C-2/C-3/C-3′/C-5), 73.9 (CH_{Bn}), 73.4 (2C, 2 × CH_{Bn}) , 73.1 (CH_{Bn}), 72.9 (CH_{Bn}), 71.8 (C-4′), 70.3 (C-5′), 69.5(C-2/C-3/C-3′/C-5), 69.1 (C-6′), 68.1 (2C, C-6, CH_{Linker}), 67.3 (CH_{Cbz}), 50.6/50.3 (NCH_{Bn}), 47.3/46.3 (CH_{Linker}), 29.3 (CH_{Linker}), 28.1/27.7 (CH_{Linker}), 23.6 (CH_{Linker}); Due to signal overlap, 51 out of 74 C atoms were assigned; **^{1}H-^{13}C-coupled HSQC** ($CDCl_3$): $J_{C1,H1}$ = 169 Hz, $J_{C1'H1'}$ = 169 Hz; **HRMS** (ESI$^+$): Calculated for $C_{74}H_{85}O_{13}N_2^+$ $[M+NH_4]^+$: 1209.6046; found: 1209.6078.

N-(Benzyl)-benzyloxycarbonyl-5-aminopentyl-(2,3-di-O-benzyl-6-O-*tert*-butyldimethyl-silyl-α-D-glucopyranosyl)-(1→4)-2,3,6-tri-O-benzyl-α-D-galactopyranoside (11)

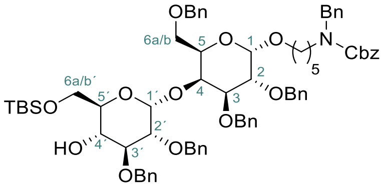

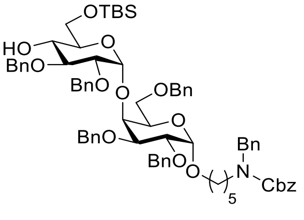

To a solution of disaccharide **29** (350 mg, 0.29 mmol, 1.0 eq.) in CH_2Cl_2 (16 ml) were added ethanethiol (212 µl, 2.94 mmol, 10 eq.) and *p*-TsOH (30.0 mg, 0.15 mmol, 0.5 eq.). The reaction was stirred for 1 h at ambient temperature, before it was neutralized by the addition of NEt_3 (0.5 ml). The solvents were removed under reduced pressure to afford the crude diol, which was subjected to flash chromatography (^{c}Hex/EtOAc v/v = 4:1 → 2:1). The product containing fractions were pooled and organic solvents were removed under reduced pressure.

The obtained diol was dissolved in dry DMF (10 ml) and imidazole (102 mg, 1.50 mmol, 5.0 eq.) and TBSCl (110 mg, 0.73 mmol, 2.5 eq.) were added sequentially. The reaction was stirred at ambient temperature until the TLC monitoring indicated complete conversion of the starting material. The reaction was stopped by the addition of MeOH (2 ml) and was stirred for 10 min at ambient temperature. Subsequently, the organic solvents were removed under reduced pressure and the crude residue was co-evaporated with toluene (2 × 15 ml). The crude product was dissolved in CH_2Cl_2 (20 ml) and washed with 1 M HCl (15 ml) and brine (15 ml) and was dried with $MgSO_4$. The solvents were removed under reduced pressure and the crude silyl ether was subjected to column chromatography (^{c}Hex/EtOAc v/v = 5:1) to obtain **11** (296 mg, 0.24 mmol, 83 % over two steps) as a colorless oil.

R*f* = 0.33 (^{c}Hex/EtOAc v/v = 4:1); **RP HPLC** (Luna**,** 0.1 % TFA; 0 min 50 % B → 10 min 100 % B, flow: 1ml/min): t_R = 25.48 min, λ = 230 nm; $[\alpha]_D^{24}$ = + 45.0 ° (c = 1.0; $CHCl_3$); **^{1}H NMR** (600 MHz, $CDCl_3$): δ [ppm] = 7.44 – 7.11 (m, 35, Ar-H), 5.17 (d, $J_{CH,CH}$ = 13.4 Hz, 2H, CH_{Cbz}), 4.98 (d, $J_{H1',H2'}$ = 3.3 Hz, 1H, H-1′), 4.93 – 4.90 (m, 1H, CH_{Bn}), 4.88 – 4.83 (m, 2H, 2 ×CH_{Bn}), 4.82 – 4.76 (m, 3H, H-1, 2 ×CH_{Bn}), 4.71 – 4.63 (m, 3H, 3 ×CH_{Bn}), 4.47 (d, $J_{CH,CH}$ = 15.0 Hz, 2H, NCH_{Bn}), 4.29 – 4.20 (m, 2H, 2 ×CH_{Bn}), 4.14 – 4.07 (m, 2H, H-5′, H-4), 3.95 (dd, $J_{H2,H3}$ = 10.3 Hz, $J_{H2,H1}$ = 3.6 Hz, 1H, H-2), 3.93 – 3.83 (m, 4H, H-3, H-3′,H-5,H-6a), 3.72 (t, $J_{H4',H3'}$ = $J_{H4',H5'}$ = 9.3 Hz, 1H, H-4′), 3.61 – 3.52 (m, 2H, H-6a′, CH_{Linker}), 3.51 – 3.49 (m, 1H, H-6b) 3.47 (dd, $J_{H2',H3'}$ = 9.9 Hz, $J_{H2',H1'}$ = 3.4 Hz, 1H, H-2′), 3.44 – 3.35 (m, 1H, CH_{Linker}), 3.33 (dd, $J_{H6b',H6a'}$ = 10.7 Hz, $J_{H6b',H5'}$ = 4.7 Hz, 1H, H-6b′), 3.28 –

3.13 (m, 2H, 2 × CH_{Linker}), 2.77 (s, 1H, OH), 1.65 – 1.41 (m, 4H, 4 × CH_{Linker}), 1.35 – 1.18 (m, 2H, 2 × CH_{Linker}), 0.82 (s, 9H, Si-^{t}Bu), -0.05 (s, 3H, Si-CH_3), -0.07 (s, 3H, Si-CH_3); **^{13}C NMR** (150 MHz, $CDCl_3$): δ [ppm] = 156.8/156.3 (C=O-Cbz), 139.2, 139.0, 138.8, 138.5, 138.4, 138.1, 137.0/136.9 (7 × Cq), 128.7, 128.6, 128.5 (3C), 128.4, 128.3, 128.0 (2C), 127.9, 127.8, 127.7 (2C), 127.5 (2C), 127.3 (16 × C-Ar), 99.7 (C-1′), 97.7 (C-1), 81.7 (C-3/C-3′/C-5), 80.2 (C-2′), 78.2 (C-3/C-3′/C-5), 76.6 (C-4), 75.6 (C-2), 75.5 (CH_{Bn}), 73.9 (CH_{Bn}), 73.5 (CH_{Bn}), 73.1 (CH_{Bn}), 72.7 (CH_{Bn}), 72.5 (C-4′), 70.9 (C-5′), 69.6 (C-3/C-3′/C-5), 68.4 (C-6), 68.2 (CH_{Linker}), 67.3 (CH_{Cbz}), 63.7 (C-6′), 50.6/50.3 (NCH_{Bn}), 47.3/46.3 (CH_{Linker}), 29.3 (CH_{Linker}), 28.1/27.7(CH_{Linker}), 26.1 (3C, Si-^{t}Bu), 23.6 (CH_{Linker}), 18.5 (Cq, Si-^{t}Bu), -5.3 (Si-CH_3), -5.4 (Si-CH_3); Due to signal overlap, 54 out of 73 C atoms were assigned; **^{1}H-^{13}C-coupled HSQC** ($CDCl_3$): $J_{C1,H1}$ = 173 Hz, $J_{C1'H1'}$ = 172 Hz; **HRMS** (ESI$^+$): Calculated for $C_{73}H_{93}O_{13}N_2Si^+$ $[M+NH_4]^+$: 1233.6441; found: 1233.6473.

_N_-(Benzyl)-benzyloxycarbonyl-5-aminopentyl-(2,3,6-tri-_O_-benzyl-_α_-D-glucopyranosyl)-(1→4)-2,3-di-_O_-benzyl-6-deoxy-6-fluoro-_α_-D-galactopyranoside (12)

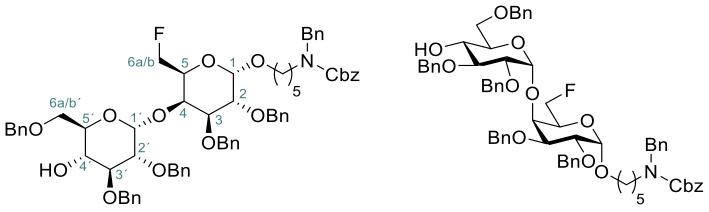

Glycosylation: The thioglycoside **21** (458 mg, 0.83 mmol, 1.5 eq.) and acceptor **19** (370 mg, 0.55 mmol, 1.0 eq.) were combined, co-evaporated with toluene (50 ml) and dried under high vacuo for 1 h. The starting materials were dissolved in dry CH_2Cl_2 (6 ml) and stirred for 1 h with freshly activated MS 4 Å. Subsequently, the reaction mixture was cooled to -30 °C and NIS (198 mg, 0.88 mmol, 1.6 eq.) and TMSOTf (9.90 µl, 60.0 µmol, 0.1 eq.) were added. The reaction was stirred for 2 h at -30 °C before another portion of TMSOTf (9.90 µl, 60.0 µmol, 0.1 eq.) was added. The reaction was slowly warmed to -10 °C and then to 0 °C and it was stirred for another 2 h. After the reaction was deemed complete by the TLC monitoring, it was neutralized by addition of NEt_3 (1 ml), diluted with CH_2Cl_2 and filtered through a pad of Celite® Hyflo Supercel. The organic phase was washed with sat. aq. $Na_2S_2O_3$ (15 ml), 1 M HCl (10 ml), and brine (10 ml) and dried over $MgSO_4$. After evaporation of the solvents the crude product was subjected to flash column chromatography (^{c}Hex/EtOAc v/v = 7:1) to obtain **30**

(480 mg*) as a colorless oil. *NMR analysis revealed an inseparable impurity most likely arising from decomposition of donor **21.**

R$_f$ = 0.48 (^{c}Hex/EtOAc v/v = 3:1); **RP HPLC** (Luna**,** 0.1 % TFA; 0 min 50 % B → 10 min 100 % B, flow: 1ml/min): t_R = 18.13 min, λ = 230 nm; **^{1}H NMR** (400 MHz, $CDCl_3$): δ [ppm]= 7.54 – 7.13 (m, 35H, Ar-H), 5.51 (s, 1H, CH-Ar), 5.18 (d, $J_{CH,CH}$ = 10.6 Hz, 2H, CH_{Cbz}), 4.96 (d, $J_{CH,CH}$ = 11.1 Hz, 1H, CH_{Bn}), 4.91 – 4.68 (m, 10H, H-1, H-1′, H-6a, 7 × CH_{Bn}), 4.50 (d, $J_{CH,CH}$ = 8.6 Hz, 2H, NCH_{Bn}), 4.36 (dt, $J_{H6b,F}$ = 46.4 Hz, $J_{H6b,H6a}$ = 7.4 Hz, 2H, H-6b), 4.20 (td, $J_{H5',H4'}$ = 9.9 Hz, $J_{H5',H6a'}$ = 4.9 Hz, 1H, H-5′), 4.07 – 3.85 (m, 5H, H-2, H-3, H-4, H-5, H-3′), 3.74 (dd, $J_{H6a',H6b'}$ = 10.1 Hz, $J_{H6a',H5'}$ = 4.9 Hz, 1H, H-6a′), 3.64 – 3.50 (m, 3H, H-2′, H-4′, CH_{Linker}), 3.50 – 3.37 (m, 2H, H-6b′, CH_{Linker}), 3.31 – 3.12 (m, 2H, 2 × CH_{Linker}), 1.68 – 1.46 (m, 4H, 4 × CH_{Linker}), 1.37 – 1.22 (m, 2H, 2 × CH_{Linker}); **^{13}C NMR** (100 MHz, $CDCl_3$): δ [ppm] = 156.8/156.3 (C=O-Cbz), 139.0, 138.6, 138.4, 138.0, 137.8 (5 × Cq), 137.0/136.9 (Cq_{Cbz}), 128.9, 128.7, 128.6, 128.5 (3C), 128.4, 128.3, 128.1, 128.0 (2C), 127.9, 127.8, 127.7, 126.2 (15 × C-Ar), 101.2 (CH-Ar), 100.9 (C-1′), 98.0 (C-1), 82.8 (C-4′), 81.0 (d, $J_{C6,F}$ = 162.4 Hz, C-6), 79.6 (C-2′), 79.1 (C-3′), 77.6 (C-2/C-3), 77.2 (C-4), 75.3 (CH_{Bn}), 74.7 (CH_{Bn}), 74.5 (C-2/C-3), 73.5 (CH_{Bn}), 73.0 (CH_{Bn}), 69.0 (C-6′), 68.9 − 68.3 (2C, C-5, CH_{Linker}), 67.3 (CH_{Cbz}), 63.3 (C-5′), (50.6/50.3 (NCH_{Bn}), 47.3/46.3 (CH_{Linker}), 29.2 (CH_{Linker}), 28.1/27.6 (CH_{Linker}), 23.5 (CH_{Linker}); Due to signal overlap, 46 out of 67 C atoms were assigned; **^{19}F NMR** (377 MHz, $CDCl_3$): δ [ppm] = -235.1 – -235.6 (m); **^{1}H-^{13}C-coupled HSQC** ($CDCl_3$): $J_{C1,H1}$ = 170 Hz, $J_{C1'H1'}$ = 171 Hz; **HRMS** (ESI$^+$): Calculated for $C_{67}H_{73}FNO_{12}^+$ [M+H]$^+$: 1102.5112; found: 1102.5139.

Regioselective benzylidene opening: A stirred solution of the glycosylation product **30** (480 mg) in dry MeCN/CH_2Cl_2 (v/v = 3:1, 40 ml) was cooled to -10 °C before $BH_3{\cdot}NMe_3$ (238 mg, 3.26 mmol, 7.4 eq.) and $BF_3{\cdot}OEt_2$ (413 µl, 3.26 mmol, 7.4 eq.) were added. The reaction was stirred at -10 °C for 1.5 h, before another portion of $BH_3{\cdot}NMe_3$ (32 mg, 0.44 mmol, 1.0 eq.) and $BF_3{\cdot}OEt_2$ (62 µl, 0.44 mmol, 1.0 eq.) were added. After the TLC monitoring indicated the complete conversion of the starting material, the reaction was stopped by the addition of a methanolic NEt_3 solution (33 % vol.) and warmed to ambient temperature (over 0.5 h). The organic solvents were removed under reduced pressure and the crude residue was directly subjected to column chromatography (^{c}Hex/EtOAc v/v = 7:1) obtaining **12** (375 mg, 0.34 mmol, 62 % over two steps) as a colorless oil.

R$_f$ = 0.39 (^{c}Hex/EtOAc v/v = 3:1); **RP-HPLC** (Luna, 0.1 % TFA; 0 min 50 % B → 10 min 100 % B, flow: 1ml/min): t_R = 16.73, λ = 230 nm; $[\alpha]_D^{22}$ = + 51.8 ° (c = 1.1; $CHCl_3$); **^{1}H NMR** (600 MHz,

$CDCl_3$): δ [ppm] = 7.44 – 7.13 (m, 35H, Ar-H), 5.18 (d, $J_{CH,CH}$ = 17.1 Hz, 2H, CH_{Cbz}), 4.96 (d, $J_{CH,CH}$ = 11.4 Hz, 1H, CH_{Bn}), 4.91 (d, $J_{H1',H2'}$ = 3.4 Hz, 1H, H-1′), 4.86 – 4.64 (m, 9H, H-1, H-6a, 7 × CH_{Bn}), 4.50 (d, $J_{CH,CH}$ = 16.0 Hz, 2H, NCH_{Bn}), 4.47 – 4.34 (m, 2H, H-6b, CH_{Bn}), 4.21 (d, $J_{CH,CH}$ = 12.2 Hz, 1H, CH_{Bn}), 4.10 (dt, $J_{H5',H4'}$ = 9.6 Hz, $J_{H5',H6a'}$ = $J_{H5',H6b'}$ = 3.6 Hz, 1H, H-5′), 4.08 – 4.04 (m, 1H, H-4), 4.00 – 3.93 (m, 1H, H-5), 3.92 – 3.84 (m, 2H, H-2, H-3), 3.79 (t, $J_{H3',H2'}$ = $J_{H3',H4'}$ = 9.1 Hz, 1H, H-3′), 3.75 (td, $J_{H4',H3'}$ = $J_{H4',H5'}$ = 9.2 Hz, $J_{H4',OH}$ = 2.5 Hz, 1H, H-4′), 3.63 – 3.55 (m, 1H, CH_{Linker}), 3.53 (dd, $J_{H2',H3'}$ = 9.4 Hz, $J_{H2',H1'}$ =3.4 Hz, 1H, H-2′), 3.46 – 3.37 (m, 1H, CH_{Linker}), 3.36 (dd, $J_{H6a',H6b'}$ = 10.3 Hz, $J_{H6a',H5'}$ = 3.0 Hz, 1H, H-6a′), 3.29 – 3.17 (m, 2H, 2 × CH_{Linker}), 3.14 (dd, $J_{H6b',H6a'}$ = 10.3 Hz, $J_{H6b',H5'}$ = 4.0 Hz, 1H, H-6b′), 2.46 (d, $J_{OH,H4'}$ = 2.6 Hz, 1H, OH), 1.66 – 1.47 (m, 4H, 4 × CH_{Linker}), 1.39 – 1.20 (m, 2H, 2 × CH_{Linker}); **^{13}C NMR** (150 MHz, $CDCl_3$): δ [ppm] = 156.9/156.3 (C=O-Cbz), 139.1, 138.8, 138.5, 138.2, 138.0 (2C), 137.0/ 136.9 (7 × Cq), 128.7, 128.6 (2C), 128.5, 128.4 (2C), 128.3, 128.2, 128.0 (3C), 127.9 (2C), 127.8, 127.7 (2C), 127.6 (2C), 127.5, 127.4, 127.3 (21 × C-Ar), 100.1 (C-1′), 97.8 (C-1), 81.6 (C-3′), 80.5 (C-6, d, $J_{C6,F}$ = 166 Hz), 80.1 (C-2′), 77.5 (C-2/C-3), 76.7 (d, $J_{C4,F}$ = 2.9 Hz, C-4), 75.4 (2C, C-2/C-3, CH_{Bn}), 74.1 (CH_{Bn}), 73.5 (2C, 2 × CH_{Bn}), 72.9 (CH_{Bn}), 71.7 (C-4′), 70.5 (C-5′), 69.2 (C-6′), 68.9 (d, $J_{C5,F}$ = 25 Hz, C-5), 68.3 (CH_{Linker}), 67.3 (CH_{Cbz}), 50.6/50.3 (NCH_{Bn}), 47.3/46.2 (CH_{Linker}), 29.2 (CH_{Linker}), 28.1/27.6 (CH_{Linker}), 23.5 (CH_{Linker}); Due to signal overlap, 53 out of 67 C atoms were assigned; **^{19}F NMR** (377 MHz, CDCl3): δ [ppm] = -230.0 – -231.4 (m); **^{1}H-^{13}C-coupled HSQC** ($CDCl_3$): $J_{C1,H1}$ = 174 Hz, $J_{C1'H1'}$ = 170 Hz; **HRMS** (ESI$^+$): Calculated for $C_{67}H_{74}O_{12}NFNa^+$[M+Na]$^+$: 1126.5087; found: 1126.5118.

Synthesis of cellobiosyl donor 13

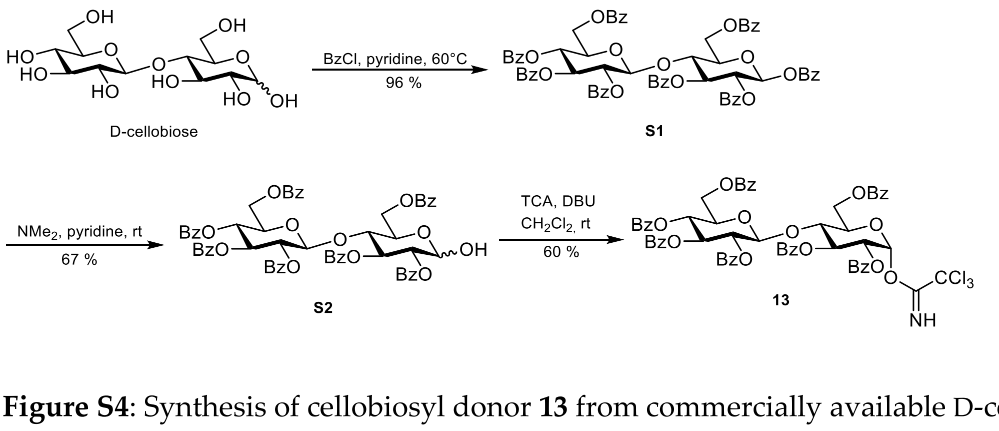

Figure S4: Synthesis of cellobiosyl donor **13** from commercially available D-cellobiose.

2,3,4,6-Tetra-*O*-benzoyl-β-D-glucopyranosyl-(1→4)-1,2,3,6-tetra-*O*-benzoyl-β-D-gluco-pyranoside (S1)

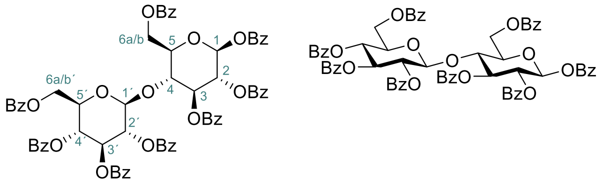

To a stirred solution of D-cellobiose (1.00 g, 2.92 mmol, 1.0 eq.) in pyridine (15 ml), benzoyl chloride (5.75 ml, 49.7 mmol, 17.0 eq.) was slowly added. The reaction mixture was stirred for 16 h at 60 °C and subsequently poured onto ice. The aqueous mixture was extracted with CH_2Cl_2 (2 × 25 ml), and the combined organic layers were washed with 1 M HCl (25 ml), brine (25 ml), and were dried with $MgSO_4$. The organic solvents were removed under reduced pressure. The crude product was purified by crystallization from EtOH, providing compound **S1** as a colorless solid (3.28 g, 2.79 mmol, 96 %).

R*f* = 0.56 (^{c}Hex/EtOAc v/v = 2:1); **^{1}H NMR** (400 MHz, $CDCl_3$): δ [ppm] = 8.04 – 7.93 (m, 10H, Ar-H), 7.92 – 7.86 (m, 2H, Ar-H), 7.76 – 7.73 (m, 4H, Ar-H), 7.59 – 7.49 (m, 2H, Ar-H), 7.48 – 7.35 (m, 12H, Ar-H), 7.34 – 7.20 (m, 10H, Ar-H), 6.12 (d, $J_{H1,H2}$ = 8.0 Hz, 1H, H-1), 5.94 (t, $J_{H3,H2}$ = $J_{H3,H4}$ = 9.2 Hz, 1H, H-3), 5.79 – 5.71 (m, 2H, H-2, H-3′), 5.58 – 5.52 (m, 1H, H-2′), 5.41 (t, $J_{H4',H3'}$ = $J_{H4',H5'}$ = 9.5 Hz, 1H, H-4′), 4.96 (d, $J_{H1',H2'}$ = 7.9 Hz, 1H, H-1′), 4.61 (dd, $J_{H6a,H6b}$ = 12.4 Hz, $J_{H6a,H5}$ = 1.9 Hz, 1H, H-6a), 4.50 (dd, $J_{H6b,H6a}$ = 12.4 Hz, $J_{H6b,H5}$ = 4.0 Hz, 1H, H-6b), 4.38 (t, $J_{H4,H3}$ = $J_{H4,H5}$ = 9.3 Hz, 1H, H-4), 4.10 – 4.01 (m, 2H, H-5, H-6a′), 3.86 – 3.77 (m, 2H, H-5′, H-6b′); **^{13}C-NMR** (100 MHz, CDCl3): δ [ppm] = 165.8 (2C), 165.7, 165.4, 165.3, 165.1, 164.9, 164.6 (8 × C=O), 133.9, 133.6, 133.5 (3C), 133.4 (3C), 130.7, 130.2, 129.9, 129.8 (2C), 129.7, 129.0, 128.7, 128.6 (2C), 128.5, 128.4 (2C, 21 × C-Ar), 101.2 (C-1′), 92.6 (C-1), 76.1 (C-4), 73.9 (C-5), 72.9 (C-3), 72.8 (C-3′), 72.5 (C-5′), 71.9 (C-2′), 70.9 (C-2), 69.5 (C-4′), 62.7 (C-6′), 62.2 (C-6). Due to signal overlap 41 out of 68 C atoms were assigned; **HRMS** (ESI^+): Calculated for $C_{68}H_{58}NO_{19}^+$ $[M+NH_4]^+$: 1192.3598; found: 1192.3625.

2,3,4,6-Tetra-*O*-benzoyl-β-D-glucopyranosyl-(1→4)-2,3,6-tri-*O*-benzoyl-*α*/β-D-gluco-pyranoside (S2)

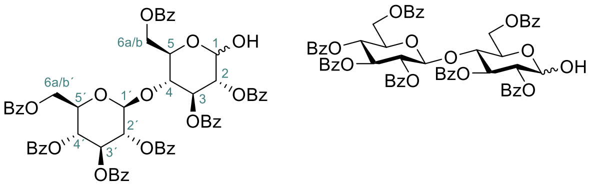

To a stirred solution of benzoyl ester **S1** (1.00 g, 0.86 mmol, 1.0 eq.) in pyridine (10 ml), dimethylamine (2 M in THF, 12 ml) was added. The reaction mixture was stirred for 30 h at ambient temperature before being diluted with CH_2Cl_2. The organic layer was washed with 1M HCl (15 ml) and brine (15 ml), before it was dried with $MgSO_4$. The crude product was subjected to column chromatography (^{c}Hex/EtOAc v/v = 3:1 → 2:1) to obtain **S2** (614 mg, 0.58 mmol, 67 %) as a mixture of anomers (*α*/β ~ 2:1).

R*f* = 0.33 (^{c}Hex/EtOAc v/v = 2:1);**: RP-HPLC** (Luna, 0.1 % TFA; 0 min 50 % B → 10 min 100 % B, flow: 1ml/min, λ = 230 nm): t_R = 13.64 min (*α*-anomer), t_R = 13.30 min (β-anomer); **^{1}H NMR** (600 MHz, $CDCl_3$) *α*-anomer: δ [ppm] = 8.03 – 7.19 (m, 35H, Ar-H), 6.14 (dd, $J_{H3,H2}$ = 10.1 Hz, $J_{H3,H4}$ = 9.2 Hz, 1H, H-3), 5.76 (t, $J_{H3',H2'}$ = $J_{H3',H4'}$ = 9.7 Hz, 1H, H-3′), 5.60 (d, $J_{H1,H2}$ = 3.5 Hz, 1H, H-1), 5.54 (dd, $J_{H2',H3'}$ = 9.9 Hz, $J_{H2',H1'}$ = 7.9 Hz, 1H, H-2′), 5.42 (t, $J_{H4',H3'}$ = $J_{H4',H5'}$ = 9.6 Hz, 1H, H-4′), 5.19 (dd, $J_{H2,H3}$ = 10.2 Hz, $J_{H2,H1}$ = 3.6 Hz, 1H, H-2), 5.02 (d, $J_{H1',H2'}$ = 8.0 Hz, 1H, H-1′), 4.61 (dd, $J_{H6a,H6b}$ = 12.2 Hz, $J_{H6a,H5}$ = 2.0 Hz, 1H, H-6a), 4.47 (dd, $J_{H6b,H6a}$ = 12.1, $J_{H6b,H5}$ = 3.7 Hz, 1H, H-6b), 4.37 (ddd, $J_{H5,H4}$ = 10.1 Hz, $J_{H5,H6b}$ = 3.7 Hz, $J_{H5,H6a}$ = 1.9 Hz, 1H, H-5), 4.24 (t, $J_{H4,H3}$ = $J_{H4,H5}$ = 9.8 Hz, 1H, H-4), 4.14 – 4.09 (m, 1H, H-6a′), 3.89 (dd, $J_{H6a',H6b'}$ = 11.9 Hz, $J_{H6a',H5'}$ = 5.4 Hz, 1H, H-6b′), 3.86 – 3.82 (m, 1H, H-5′); β-anomer (selected signals): δ [ppm] = 5.85 (t, $J_{H3,H2}$ = $J_{H3,H4}$ = 9.5 Hz, 1H, H-3), 5. 47 (t, $J_{H3',H2'}$ = $J_{H3',H4'}$ = 9.7 Hz, 1H, H-3′), 5.22 (dd, $J_{H2,H3}$ = 10.0 Hz, $J_{H2,H1}$ = 8.0 Hz, 1H, H-2), 4.96 (d, $J_{H1',H2'}$ = 7.9 Hz, 1H, H-1′), 4.89 (d, $J_{H1,H2}$ = 8.0 Hz, H-1); **^{13}C NMR** (150 MHz, $CDCl_3$) *α*-anomer: δ [ppm] = 166.1, 166.0, 165.9, 165.8, 165.5, 165.1, 164.9 (7 × C=O), 133.5 (3C), 133.4, 133.3 (3C), 130.3, 130.0, 129.9 (2C), 129.8 (2C), 129.7, 129.6, 129.1, 128.6 (3C), 128.5 (2C), 128.4 (2C, 23 × C-Ar), 100.9 (C-1′), 90.4 (C-1), 76.5 (C-4), 73.0 (C-3′), 72.5 (C-5′), 72.4 (C-2), 72.1 (C-2′), 69.9 (C-3), 69.6 (C-4′), 68.7 (C-5), 62.8 (C-6′), 62.4 (C-6). Due to signal overlap, 42 out of 61 C atoms were assigned; β-anomer (selected signals): δ [ppm] = 167.1, 166.0, 165.8 (2C), 165.5, 165.1 (2C, 7 × C=O), 133.8, 133.7, 133.4, 130.1, 129.7, 129.6, 129.1, 128.8 (2C), 128.7 (10 × C-Ar), 101.1 (C-1′), 95.9 (C-1), 76.5 (C-4), 74.5 (C-2), 73.4 (C-5′), 72.9 (C-2′), 72.5 (C-3′/C-5′), 72.2 (C-3),

72.0 (C-3′/C-5′), 69.5 (C-4′), 62.8 (C-6′), 62.5 (C-6) **HRMS** (ESI+): Calculated for $C_{61}H_{54}O_{18}N^+$ $[M+NH_4]^+$: 1088.3336; found: 1088.3364.

2,3,4,6-Tetra-*O*-benzoyl-β-D-glucopyranosyl-(1→4)-2,3,6-tri-*O*-benzoyl-*α*-D-gluco-pyranosyl trichloroacetimidate (13)

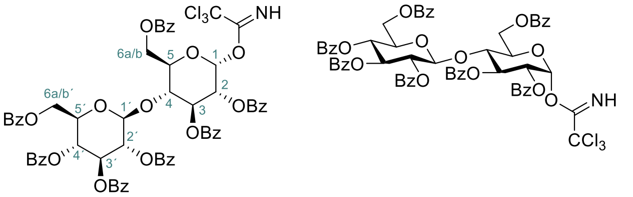

To a stirred solution of **S2** (730 mg, 0.68 mmol, 1.0 eq.) in dry CH_2Cl_2 (20 ml) trichloroacetonitrile (682 µl, 6.80 mmol, 10.0 eq.) and DBU (102 µl, 0.68 mmol, 1.0 eq.) were added at 0 °C. The reaction mixture was slowly warmed to ambient temperature and stirred for 24 h. Then, the reaction mixture was concentrated to dryness, and the crude residue was dissolved in CH_2Cl_2 (500 µl) and directly subjected to column chromatography (cHex/EtOAc v/v = 3:1 + 1 % NEt_3) to obtain **13** (500 mg, 0.41 mmol, 60 %) as a colorless oil.

R*f* = 0.39 (cHex/EtOAc v/v = 2:1 + 1 % NEt_3); **^{1}H NMR** (600 MHz, $CDCl_3$): δ [ppm] = 8.55 (s, 1H, N=H), 8.02 – 8.00 (m, 2H, Ar-H), 7.97 (dd, $J_{CH,CH}$ = 8.3 Hz, 1.4 Hz, 2H, Ar-H), 7.94 – 7.91 (m, 6H, Ar-H), 7.77 (dd, $J_{CH,CH}$ = 8.3 Hz, $J_{CH,CH}$ = 1.4 Hz, 2H, Ar-H), 7.75 – 7.73 (m, 2H, Ar-H), 7.58 – 7.21 (m, 21H, Ar-H), 6.69 (d, $J_{H1,H2}$ = 3.8 Hz, 1H, H-1), 6.16 – 6.09 (m, 1H, H-3), 5.75 (t, $J_{H3',H2'}$ = $J_{H3',H4'}$ = 9.6 Hz, 1H, H-3′), 5.53 (dd, $J_{H3',H2'}$ = 9.8 Hz, $J_{H2',H1'}$ = 7.9 Hz, 1H, H-2′), 5.48 (dd, $J_{H2,H3}$ = 10.1 Hz, $J_{H2,H1}$ = 3.8 Hz, 1H, H-2), 5.42 (t, $J_{H4',H3'}$ = $J_{H4',H5'}$ = 9.7 Hz, 1H, H-4′), 5.02 (d, $J_{H1',H2'}$ = 7.9 Hz, 1H, H-1′), 4.59 (dd, $J_{H6a,H6b}$ = 12.4 Hz, $J_{H6a,H5}$ = 1.8 Hz, 1H, H-6a), 4.50 (dd, $J_{H6b,H6a}$ = 12.2 Hz, $J_{H6b,H5}$ = 3.4 Hz, 1H, H-6b), 4.36 – 4.31 (m, 2H, H-4, H-5), 4.07 (dd, $J_{H6a', H6b'}$ = 12.0 Hz, $J_{H6a', H5'}$ =3.3 Hz, 1H, H-6a′), 3.88 (dd, $J_{H6b', H6a'}$ = 12.0 Hz, $J_{H6b', H5'}$ = 5.1 Hz, 1H, H-6b′), 3.84 – 3.80 (m, 1H, H-5′); **^{13}C NMR** (150 MHz, $CDCl_3$): δ [ppm] = 165.8 (3C), 165.6, 165.3, 165.1, 164.9 (7 × C=O), 160.8 (C=NH), 133.7, 133.5 (2C), 133.4 (2C), 133.3, 130.0, 129.9 (2C), 129.8 (4C), 129.7 (2C), 129.6, 129.5, 128.6 (2C), 128.5 (3C), 128.4 (23 × C_{Ar}), 101.3 (C-1′), 93.1 (C-1), 90.8 (CCl_3), 76.1 (C-4), 73.0 (C-3′), 72.6 (C-5′), 72.2 (C-2′), 71.4 (C-5), 70.8 (C-2), 70.2 (C-3), 69.5 (C-4′), 62.8 (C-6′), 62.0 (C-6). Due to signal overlap, only 44 out of 63 C atoms were assigned; **HRMS** (ESI+): Calculated for $C_{63}H_{54}Cl_3N_2O_{18}^+$ $[M+NH_4]^+$: 1231.2432; found: 1231.2478.

Synthesis of glucosyl acceptor 20 and fluorinated glucosyl building block 17

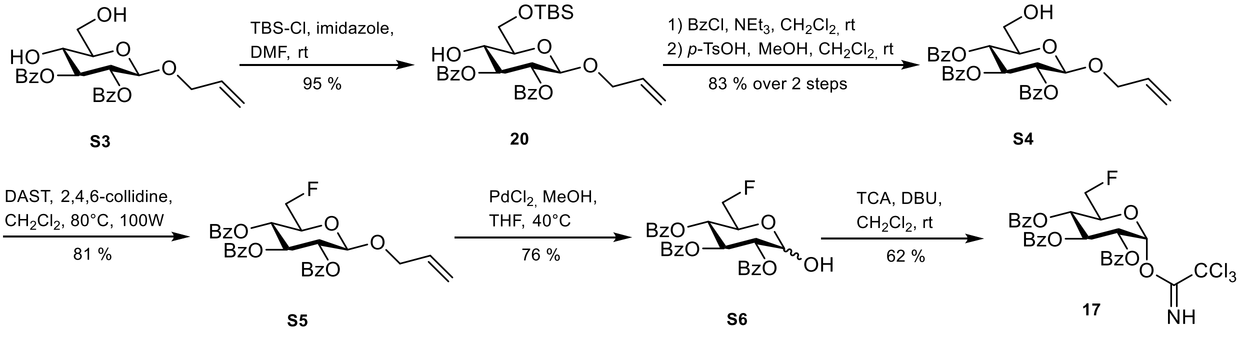

Figure S5: Synthesis of fluorinated donor building block **17** via key building block **20**.

Allyl-2,3-di-*O*-benzoyl-6-*O*-*tert*-butyldimethylsilyl-β-D-glucopyranoside (20)

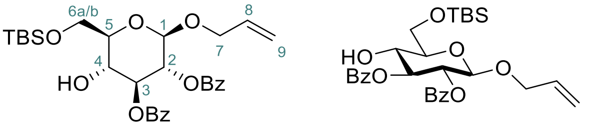

Imidazole (636 mg, 9.34 mmol, 5.0 eq.) and *tert*-butyldimethylsilyl chloride (704 mg, 4.67 mmol, 2.5 eq.) were added to a solution of **S3** (800 mg, 1.87 mmol, 1.0 eq.) in dry DMF (40 ml), and the reaction was stirred for 45 min at ambient temperature. Upon completion, MeOH (2.5 ml) was added, and the reaction was evaporated to dryness. The crude product was dissolved in CH_2Cl_2 (100 ml) and washed with 1 M HCl (50 ml), 10 % aq. LiCl (50 ml) and brine (25 ml) and dried over $MgSO_4$. The crude product was subjected to flash column chromatography (^{c}Hex/EtOAc v/v = 7:1) to obtain **20** (961 mg, 1.77 mmol, 95 %) as a colorless oil.

R_f= 0.19 (^{c}Hex/EtOAc v/v = 7:1); **RP HPLC** (Luna, 0.1 % TFA; 0 min 50 % B → 10 min 100 % B, flow: 1ml/min): t_R = 13.85 min, λ = 230 nm; $[\alpha]_D^{24}$ = + 16.8 ° (c = 0.5, $CHCl_3$); **^{1}H NMR** (400 MHz, $CDCl_3$): δ [ppm] = 7.97 (m, 4H, H-Ar), 7.56 – 7.44 (m, 2H, H-Ar), 7.37 (m, 4H, H-Ar), 5.78 (dddd, $J_{H8,H9trans}$ =16.9 Hz, $J_{H8,H9cis}$ =10.7 Hz, $J_{H8,H7b}$ = 6.2 Hz, $J_{H8,H7a}$ = 4.8 Hz, 1H, H-8), 5.50 (dd, $J_{H3,H2}$ = 9.8 Hz, $J_{H3,H4}$ = 9.0 Hz, 1H, H-3), 5.41 (dd, $J_{H2,H3}$ = 9.9 Hz, $J_{H2,H1}$ = 7.8 Hz, 1H, H-2), 5.22 (dd, $J_{H9trans,H8}$ = 17.2 Hz, $J_{H9trans,H9cis}$ = 1.7 Hz, 1H, H-9$_{trans}$), 5.14 – 5.10 (m, 1H, H-9$_{cis}$), 4.74 (d, $J_{H1,H2}$ = 7.8 Hz, 1H, H-1), 4.33 (ddt, $J_{H7a,H7b}$ = 13.3 Hz, $J_{H7a,H8}$ = 4.9 Hz, $J_{H7a,H9cis}$ = $J_{H7a,H9trans}$ = 1.6 Hz, 1H, H-7a), 4.12 (ddt, $J_{H7b,H7a}$ = 13.3 Hz, $J_{H7b,H8}$ = 6.2 Hz, $J_{H7b,H9cis}$ = $J_{H7b,H9trans}$ =1.4 Hz, 1H, H-7b), 4.04 – 3.91

(m, 3H, H-4, H-6a, H-6b), 3.58 (dt, $J_{H5,H4}$ = 9.3 Hz, $J_{H5,H6a}$ = $J_{H5,H6b}$ = 5.3 Hz, 1H, H-5), 3.52 (d, $J_{OH,H4}$ = 2.5 Hz, 1H, OH), 0.92 (s, 9H, Si-^{t}Bu), 0.13 (s, 3H, Si-CH_3), 0.12 (s, 3H, Si-CH_3); **^{13}C NMR** (100 MHz, $CDCl_3$): δ [ppm] = 167.1 (C=O), 165.5 (C=O), 133.6 (C-8), 133.5, 133.3, 130.1 (2C), 129.9 (2C), 129.6 (C_q), 129.3 (C_q), 128.5 (2C, 10 × C-Ar), 117.7 (C-9), 99.7 (C-1), 76.4 (C-3), 74.8 (C-5), 72.2 (C-4), 71.6 (C-2), 69.9 (C-7), 64.6 (C-6), 26.0 (3C, Si-^{t}Bu), 18.4 (C_q, Si-^{t}Bu) , − 5.30 (2C, 2× CH_3); Due to signal overlap, 27 out of 29 C atoms were assigned; **HRMS** (ESI^+): Calculated for $C_{29}H_{42}O_8NSi^+$ $[M+NH_4]^+$: 560.2674; found: 560.2683.

Allyl-2,3,4-tri-*O*-benzoyl-β-D-glucopyranoside (S4)

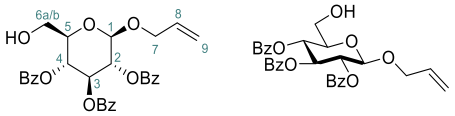

To a solution of **20** (920 mg, 1.70 mmol, 1.0 eq.) in dry CH_2Cl_2 (50 ml), NEt_3 (1.18 ml, 8.50 mmol, 5.0 eq.) and benzoyl chloride (588 µl, 5.10 mmol, 3.0 eq.) were added. The reaction was stirred at ambient temperature for 17 h before *N,N*-dimethylaminopropylamine (1.06 ml, 8.50 mmol, 5.0 eq.) was added. The resulting mixture was diluted with CH_2Cl_2 (100 ml) and washed with 1 M HCl (2 × 50 ml) and brine (25 ml). The organic layer was dried with $MgSO_4$, and the solvents were evaporated.

The crude benzoyl ester was dissolved in a mixture of CH_2Cl_2/MeOH (v/v = 1:1, 40 ml) and *p*-TsOH·H_2O (755 mg, 3.97 mmol, 2.3 eq.) was added. The reaction was stirred at ambient temperature until the TLC monitoring indicated the complete conversion of the starting material. The reaction was neutralized by the addition of NEt_3 (4 ml) and the organic solvents were removed under reduced pressure. The crude product was subjected to flash column chromatography (^{c}Hex/EtOAc v/v = 4:1) to obtain **S4** (752 mg, 1.41mmol, 83 % over two steps) as a colorless oil.

R$_f$ = 0.12 (^{c}Hex/EtOAc v/v = 4:1); **^{1}H NMR** (400 MHz, $CDCl_3$): δ [ppm] = 7.95 (m, 4H, H-Ar), 7.87 – 7.81 (m, 2H, H-Ar), 7.56 – 7.48 (m, 2H, H-Ar), 7.45 – 7.34 (m, 5H, H-Ar), 7.31 – 7.24 (m, 2H, H-Ar), 5.94 (t, $J_{H3,H2}$ = $J_{H3,H4}$ = 9.7 Hz, 1H, H-3), 5.81 (dddd, $J_{H8,H9trans}$ = 16.8 Hz, $J_{H8,H9cis}$ = 10.8 Hz, $J_{H8,H7b}$ = 6.1 Hz, $J_{H8,H7a}$ = 4.9 Hz, 1H, H-8), 5.57 – 5.47 (m, 2H, H-2, H-4), 5.26 (dq, $J_{H9trans,H8}$ = 17.2 Hz, $J_{H9trans,H9cis}$ = $J_{H9trans,H7a}$ = $J_{H9trans,H7b}$ = 1.6 Hz, 1H, H-9_{trans}), 5.15 (dq, $J_{H9cis,H8}$ = 10.5 Hz, $J_{H9cis,H9trans}$ = $J_{H9cis,H7a}$ = $J_{H9cis,H7b}$ = 1.4 Hz, 1H, H-9_{cis}), 4.89 (d, $J_{H1,H2}$ = 8.0 Hz, 1H, H-1), 4.39 (ddt,

$J_{H7a,H7b}$ = 13.3 Hz, $J_{H7a,H8}$ = 5.0 Hz, $J_{H7a, H9trans}$ = $J_{H7a, H9cis}$ = 1.6 Hz, 1H, H-7a), 4.19 (ddt, $J_{H7b,H7a}$ = 13.3 Hz, $J_{H7b,H8}$ = 6.1 Hz, $J_{H7b, H9trans}$ = $J_{H7b, H9cis}$ = 1.4 Hz, 1H, H-7b), 3.87 (ddd, $J_{H6a,H6b}$ = 12.2 Hz, $J_{H6a,OH}$ = 8.5 Hz, $J_{H6a,H5}$ = 1.8 Hz, 1H, H-6a), 3.82 – 3.72 (m, 2H, H-5, H-6b), 2.57 (dd, $J_{OH,H6a}$ = 8.7 Hz, $J_{OH,H6b}$ = 5.2 Hz, 1H, OH); **^{13}C NMR** (100 MHz, $CDCl_3$): δ [ppm] = 166.2, 166.0, 165.2 (3 × C=O), 133.8 (C-Ar), 133.6 (C-8), 133.4, 133.3, 130.1, 129.9 (2C), 129.5, 129.0, 128.7 (2C), 128.5, 128.4 (11 × C-Ar), 117.9 (C-9), 100.1 (C-1), 74.8 (C-5), 72.9 (C-3), 72.0 (C-2/C-4), 70.4 (C-7), 69.7(C-2/C-4), 61.5 (C-6); Due to signal overlap, 24 out of 30 C atoms were assigned; **HRMS** (ESI$^+$): Calculated for $C_{30}H_{33}O_9N^+$ $[M+NH_4]^+$: 550.2072; found: 550.2074.

Allyl-2,3,4-tri-*O*-benzoyl-6-deoxy-6-fluoro-β-D-glucopyranoside (S5)

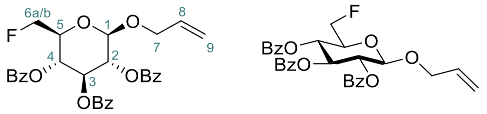

Compound **S4** (600, mg 1.13 mmol, 1.0 eq.) was dissolved in a flame-dried microwave vessel in dry CH_2Cl_2 (4 ml). Subsequently, 2,4,6-collidine (452 µl, 3.39 mmol, 3.0 eq.) and DAST (225 µl,1.70 mmol, 1.5 eq.) were added, and the reaction was stirred for 2 min at ambient temperature before it was heated in a microwave oven (80 °C, 100 W, 60 min). After the complete conversion of the starting material was observed, the reaction was stopped by the addition of MeOH (500 µl) and the mixture was diluted with CH_2Cl_2.* The organic layer was washed with sat. aq. $NaHCO_3$ (2 × 45 ml) and brine (30 ml) and was dried with $MgSO_4$. The organic solvents were removed under reduced pressure and the crude product was subjected to column chromatography (^{c}Hex/EtOAc v/v = 5:1) to obtain **S5** (1.46 g, 2.73 mmol, 81 %) as a colorless foam.

*Reaction replicates were combined for the aqueous work-up and purification.

R_f= 0.33 (^{c}Hex/EtOAc v/v = 5:1); **^{1}H NMR** (400 MHz, $CDCl_3$): δ [ppm] = 7.98 – 7.91 (m, 4H, Ar-H), 7.85 – 7.80 (m, 2H, Ar-H), 7.56 – 7.48 (m, 2H, Ar-H), 7.46 – 7.35 (m, 5H, Ar-H), 7.32 – 7.24 (m, 2H, Ar-H), 5.90 (t, $J_{H3,H2}$ = $J_{H3,H4}$ = 9.6 Hz, 1H, H-3), 5.81 (dddd, $J_{H8,H9trans}$ = 17.0 Hz, $J_{H8,H9cis}$ =10.4 Hz, $J_{H8,H7b}$ = 6.4 Hz, $J_{H8,H7a}$ = 4.8 Hz, 1H, H-8), 5.56 – 5.46 (m, 2H, H-2, H-4), 5.27 (dq, $J_{H9trans,H8}$ = 17.2 Hz, $J_{H9trans,H9cis}$ = $J_{H9trans,H7a}$ = 1.6 Hz, 1H, H-9$_{trans}$), 5.16 (dq, $J_{H9cis,H8}$ = 10.5 Hz, $J_{H9cis,H9trans}$ = 1.4 Hz, 1H, H-9$_{cis}$), 4.90 (d, $J_{H1,H2}$ = 7.8 Hz, 1H, H-1), 4.68 – 4.53 (m, 2H, H-6a, H-6b), 4.41 (ddt, $J_{H7a,H7b}$ = 13.2 Hz, $J_{H7a,H8}$ = 4.8 Hz, $J_{H7a,H9trans}$ = $J_{H7a,H9cis}$ = 1.6 Hz, 1H, H-7a), 4.19 (ddt,

$J_{H7b,H7a}$ = 13.2 Hz, $J_{H7b,H8}$ = 6.3 Hz, $J_{H7b,H9trans}$ = $J_{H7b,H9cis}$ = 1.4 Hz, 1H, H-7b), 4.04 (dddd, $J_{H5,F}$ = 19.8 Hz, $J_{H5,H4}$ = 10.0 Hz, $J_{H5,6a/b}$ = 4.8 Hz, $J_{H5,6a/b}$ = 3.2 Hz, 1H, H-5); **^{13}C NMR** (100 MHz, $CDCl_3$): δ [ppm] = 165.9, 165.4, 165.2 (3 × C=O), 133.7 (C-Ar), 133.4 (3C, 2 ×C-Ar, C-8), 130.0, 129.9 (2C), 129.4, 128.9, 128.8, 128.6, 128.5 (2C, 9 × C-Ar), 118.1 (C-9), 99.8 (C-1), 81.8 (d, $J_{C6,F}$ = 176 Hz, C-6), 73.4 (d, $J_{C5,F}$ = 19.6 Hz, C-5), 73.0 (C-3), 71.9 (C-2), 70.2 (C-7), 69.0 (d, $J_{C4,F}$ = 6.9 Hz, C-4). Due to signal overlap 24 out of 30 C atoms were assigned; **^{19}F-NMR** (377 MHz, $CDCl_3$): δ [ppm] = -230.2 (td, $J_{F,H6a}$ = $J_{F,H6b}$ = 47.0 Hz, $J_{F,H5}$ = 19.7 Hz); **HRMS** (ESI$^+$): Calculated for $C_{30}H_{31}O_8NF^+$ [M+NH_4]$^+$: 552.2028; found: 552.2031.

2,3,4-Tri-*O*-benzoyl-6-deoxy-6-fluoro-*α*/β-D-glucopyranoside (S6)

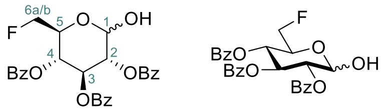

To a stirred solution of **S5** (1.20 g, 2.25 mmol, 1.0 eq.) in MeOH/THF (v/v = 4:1, 50 ml), palladium(II) chloride (199 mg, 1.12 mmol, 0.6 eq.) was added. The reaction mixture was stirred at 40 °C until the complete conversion of the starting material was observed. The reaction was diluted with THF and filtered through a pad of Celite® Hyflo Supercel. The organic solvents were removed under reduced pressure and the crude residue was dissolved in CH_2Cl_2 (50 ml), washed with water (15 ml) and brine (15 ml) and dried with $MgSO_4$. The solvents were removed under reduced pressure and the crude product was subjected to flash column chromatography (^{c}Hex/EtOAc v/v = 3:1) to obtain **S6** (850 mg, 1.72 mmol, 76 %, *α*/β = 9:1) as an amorphous solid.

***α*-S4: R*f* =** 0.19 (^{c}Hex/EtOAc v/v = 4:1); **RP-HPLC** (Luna, 0.1 % TFA; 0 min 50 % B → 10 min 100 % B, flow: 1ml/min): t_R = 10.24 min, λ = 230 nm; **^{1}H NMR** (400 MHz, $CDCl_3$): δ [ppm] = 7.98 (td, $J_{CH,CH}$ = 8.2 Hz, $J_{CH,CH}$ = 1.3 Hz, 4H, Ar-H), 7.91 – 7.85 (m, 2H, Ar-H), 7.57 – 7.47 (m, 2H, Ar-H), 7.47 – 7.36 (m, 5H, Ar-H), 7.29 (dd, $J_{CH,CH}$ = 8.4 Hz, $J_{CH,CH}$ = 7.2 Hz, 2H, Ar-H), 6.25 (t, $J_{H3,H2}$ = $J_{H3,H4}$ = 9.9 Hz, 1H, H-3), 5.79 (t, $J_{H1,H2}$ = $J_{H1,OH}$ = 3.7 Hz, 1H, H-1), 5.59 (t, $J_{H4,H3}$ = $J_{H4,H5}$ = 9.9 Hz, 1H, H-4), 5.31 (ddd, $J_{H2,H3}$ = 10.2 Hz, $J_{H2,H1}$ = 3.6 Hz, $J_{H2,OH}$ = 1.1 Hz, 1H, H-2), 4.69 – 4.49 (m, 3H, H-5, H-6a, H-6b), 3.39 (dd, $J_{OH,H1}$ = 3.9 Hz, $J_{OH,H2}$ = 1.3 Hz, 1H, OH); **^{13}C NMR** (100 MHz, $CDCl_3$): δ [ppm] = 166.0, 165.9, 165.5 (3 × C=O), 133.7, 133.6, 133.3, 130.1, 130.0, 129.8, 129.2, 129.0, 128.9, 128.6, 128.5 (11 × C-Ar), 90.6 (C-1), 81.7 (d, $J_{C6,F}$ = 175 Hz, C-6), 72.2 (C-2), 70.1 (C-3), 68.8 (d,

$J_{C5,F}$ = 18.8 Hz, C-5), 68.60 (d, $J_{C4,F}$ = 6.9 Hz, C-4). Due to signal overlap, 20 out of 27 C atoms were assigned; **^{19}F NMR** (377 MHz, $CDCl_3$): δ [ppm] = -232.0 (td, $J_{F,H6a}$ = $J_{F,H6b}$ = 47.2 Hz, $J_{F,H5}$ = 23.2 Hz).

β-S4: R$_f$ = 0.19 (^{c}Hex/EtOAc v/v = 4:1); **RP-HPLC** (Luna, 0.1 % TFA; 0 min 50 % B → 10 min 100 % B, flow: 1ml/min): t_R = 9.55 min (β), λ = 230 nm; **^{1}H NMR** (400 MHz, $CDCl_3$, selected signals): δ [ppm] = 5.96 (t, $J_{H3,H2}$ = $J_{H3,H4}$ = 9.7 Hz, 1H, H-3), 5.37 – 5.33 (m, 1H, H-2), 5.06 (t, $J_{H1,H2}$ = $J_{H1,OH}$ = 8.3 Hz, 1H, H-1); **^{13}C NMR** (100 MHz, $CDCl_3$, selected signals): δ [ppm] = 96.2 (C-1), 74.3 (C-2), 72.3 (C-3); **^{19}F NMR** (377 MHz, $CDCl_3$): δ [ppm] = -231.3 (td, $J_{F,H6a}$ = $J_{F,H6b}$ = 46.9 Hz, $J_{F,H5}$ = 20.9 Hz); **HRMS** (ESI$^+$): Calculated for $C_{27}H_{27}O_8NF^+$ $[M+NH_4]^+$: 512.1715; found: 512.1716.

2,3,4-Tri-*O*-benzoyl-6-deoxy-6-fluoro-*α*-D-glucopyranosyl trichloroacetimidate (17)

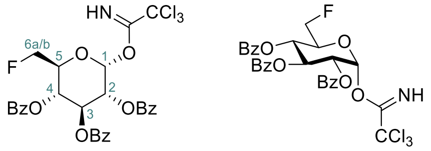

To an ice-cooled solution of lactol **S6** (500 mg, 1.01 mmol, 1.0 eq.) in dry CH_2Cl_2 (20 ml), trichloroacetonitril (1.00 ml, 10.1 mmol, 10 eq.) and DBU (165 µl, 1.11 mmol, 1.1 eq.) were added. The reaction mixture was allowed to warm to ambient temperature and was stirred for 18 h. The reaction mixture was concentrated under reduced pressure and the crude residue was subjected to column chromatography (^{c}Hex/EtOAc v/v = 5:1 + 1 % NEt_3) to obtain **17** (400 mg, 0.63 mmol, 62 %) as a colorless oil.

R*f* = 0.41 ^{c}Hex/EtOAc v/v = 4:1 + 1% NEt_3); $[\alpha]_D^{22}$ = + 46.1 ° (c = 0.5; $CHCl_3$); **^{1}H NMR** (600 MHz, $CDCl_3$): δ [ppm] = 8.68 (s, 1H, C=NH), 7.97 (ddd, $J_{CH,CH}$ = 11.9 Hz, $J_{CH,CH}$ = 8.3 Hz, $J_{CH,CH}$ = 1.4 Hz, 4H, Ar-H), 7.90 – 7.85 (m, 2H, Ar-H), 7.57 – 7.27 (m, 9H, Ar-H), 6.87 (d, $J_{H1,H2}$ = 3.6 Hz, 1H, H-1), 6.29 (t, $J_{H3,H2}$ = $J_{H3,H4}$ = 10.0 Hz, 1H, H-3), 5.75 (t, $J_{H4,H3}$ = $J_{H4,H5}$ = 10.0 Hz, 1H, H-4), 5.60 (dd, $J_{H2,H3}$ = 10.2 Hz, $J_{H2,H1}$ = 3.6 Hz, 1H, H-2), 4.70 – 4.54 (m, 2H, H-6a, H-6b), 4.49 (dddd, $J_{H5,F}$ = 23.5 Hz, $J_{H5,H4}$ = 10.5 Hz, $J_{H5,6a/b}$ = 4.1 Hz, $J_{H5,6a/b}$ = 2.3 Hz, 1H, H-5); **^{13}C NMR** (150 MHz, $CDCl_3$): δ [ppm] = 165.8, 165.5, 165.2 (3 × C=O), 160.6 (C=NH), 133.8, 133.7, 133.4, 130.0 (2C), 129.8, 129.0, 128.7 (2C), 128.6, 128.5 (2C, 12 × C-Ar), 93.3 (C-1), 90.8 (CCl_3), 81.0 (d, $J_{C6,F}$ = 177 Hz, C-6), 71.6 (d, $J_{C5,F}$ = 19.3 Hz, C-5), 70.7 (C-2), 70.2 (C-3), 67.9 (d, $J_{C4,F}$ = 6.6 Hz, C-4). Due to signal overlap, 23 out of 29 C atoms were assigned; **^{19}F NMR** (377 MHz, $CDCl_3$): δ [ppm] = -232.6 (td, $J_{F,H6a}$ =

$J_{F,H6b}$ = 47.0 Hz, $J_{F,H5}$ = 23.5 Hz); **HRMS** (ESI+): Calculated for $C_{27}H_{22}FO_7^+$ [M-OC(NH)CCl_3]+: 477.1345; found: 477.1343.

Synthesis of fluorinated glucosyl building block 14

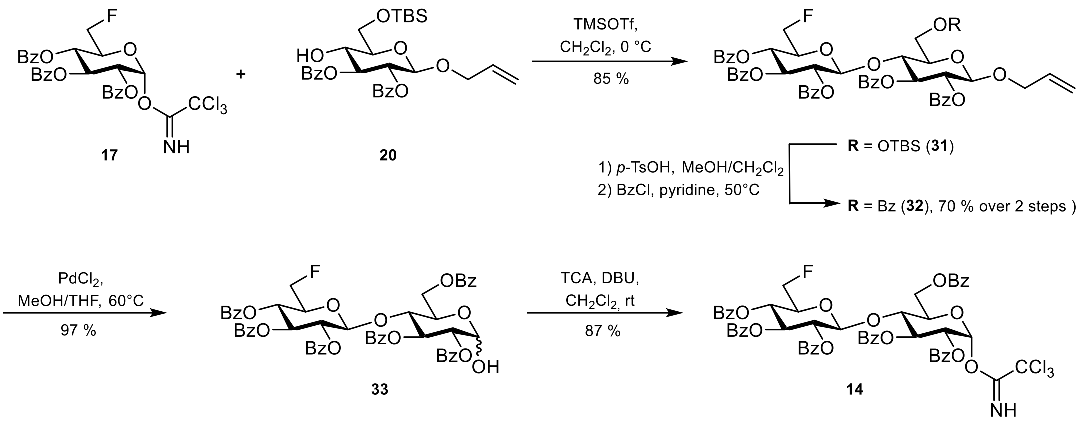

Figure S6: Synthesis of fluorinated disaccharide donor **14**.

Allyl-(2,3,4-tri-*O*-benzoyl-6-deoxy-6-fluoro-β-D-glucopyranosyl)-(1→4)-2,3-di-*O*-benzoyl-6-*O*-*tert*-butyldimethylsilyl-β-D-glucopyranoside (31)

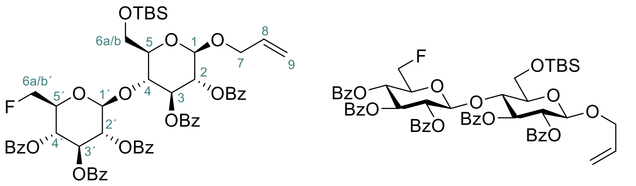

Glucosyl donor **17** (450 mg, 0.70 mmol, 1.6 eq.) and acceptor **20** (239 mmol, 0.44 mmol, 1.0 eq.) were combined, co-evaporated with toluene (12 ml) and dried under high vacuo for 1 h. The starting materials were dissolved in dry CH_2Cl_2 (12 ml) and stirred over freshly activated MS 4 Å. The reaction mixture was cooled to 0 °C and TMSOTf (7.20 µl, 0.04 mmol, 0.1 eq.) was added. After stirring for 1 h at 0 °C, another portion of TMSOTf (7.20 µl, 0.04 mmol, 0.1 eq.) was added and the reaction was slowly warmed to ambient temperature. After stirring for 3.5 h at ambiance the reaction mixture was neutralized by the addition of NEt_3. The reaction mixture was diluted with CH_2Cl_2 and filtered through a pad of Celite® Hyflo Supercel. The filtrate was washed with 1 M HCl (15 ml) and brine (10 ml) and was dried with $MgSO_4$. The crude product was subjected to flash column chromatography (^{c}Hex/EtOAc v/v = 6:1) to obtain **31** (330 mg, 0.32 mmol, 74 %) as an amorphous, colorless solid.

R*f* = 0.43 (^{c}Hex/EtOAc v/v = 3:1); **RP HPLC** (Luna, 0.1 % TFA; 0 min 50 % B → 10 min 100 % B, flow: 1ml/min): t_R = 16.61 min, λ = 230 nm; $[\alpha]_D^{22}$ = -49.1 ° (c = 0.5; $CHCl_3$); **^{1}H NMR** (400 MHz, $CDCl_3$): δ [ppm] = 8.10 – 8.06 (m, 2H, Ar-H), 7.98 – 7.93 (m, 4H, Ar-H), 7.89 – 7.84 (m, 2H, Ar-H), 7.79 – 7.73 (m, 2H, Ar-H), 7.57 – 7.45 (m, 4H, Ar-H), 7.43 – 7.31 (m, 9H, Ar-H), 7.25 (t, $J_{CH,CH}$ = 7.7 Hz, 2H, Ar-H), 5.80 – 5.65 (m, 3H, H-8, H-3, H-3′), 5.44 (dd, $J_{H2',H3'}$ = 9.8 Hz, $J_{H2',H1'}$ =8.0 Hz, 1H, H-2′), 5.37 (dd, $J_{H2,H3}$ = 9.8 Hz, $J_{H2,H1}$ = 7.9 Hz, 1H, H-2), 5.22 (t, $J_{H4',H3'}$ = $J_{H4',H5'}$ = 9.7 Hz, 1H, H-4′), 5.20 – 5.13 (m, 1H, H-9_{trans}), 5.10 – 5.04 (m, 2H, H-1′, H-9_{cis}), 4.65 (d, $J_{H1,H2}$ = 7.9 Hz, 1H, H-1), 4.31 – 3.72 (m, 8H, H-7a, H-7b, H-6a′, H-6b′, H-6a, H-6b, H-4, H-5′), 3.37 (ddd, $J_{H5, H4}$ = 9.6 Hz, $J_{H5, H6a/b}$ = 2.9 Hz, $J_{H5, H6a/b}$ = 1.6 Hz, 1H, H-5), 1.02 (s, 9H, Si-^{t}Bu), 0.16 (s, 6H, 2 × Si-CH_3). **^{13}C NMR** (100 MHz, $CDCl_3$): δ [ppm] = 165.8, 165.7, 165.4, 165.2, 164.6 (5 × C=O), 133.7 (2C, C-Ar, C-8), 133.5, 133.4, 133.2, 133.1 (2C), 130.0, 129.9 (2C), 129.9, 129.8, 129.6, 129.1, 128.7, 128.6 (2C), 128.5, 128.4, 128.3 (19 × C-Ar) , 117.4 (C-9), 100.5 (C-1′), 99.6 (C-1), 81.0 (d, $J_{C6',F}$ = 175.7 Hz, C-6′), 75.3 (C-5), 75.2 (C-4), 73.2 (2C, C-5′ (d, $J_{C5',F}$ = 20.7 Hz), C-3′), 73.1 (C-3), 72.1 (C-2), 72.0 (C-2′), 69.5 (C-7), 68.8 (d, $J_{C4',F}$ = 6.9 Hz, C-4′), 61.0 (C-6), 26.1 (3C, Si-^{t}Bu), 18.5 (C_q, Si-^{t}Bu), -4.8 (Si-CH_3), -5.1 (Si-CH_3). Due to signal overlap, 45 out of 56 C atoms were assigned. **^{19}F-NMR** (377 MHz, $CDCl_3$): δ [ppm] = -228.6 (td, $J_{F,H6a}$ = $J_{F,H6b}$ = 46.4 Hz, $J_{F,H5}$ = 19.0 Hz). **HRMS** (ESI$^+$): Calculated for $C_{56}H_{63}O_{15}FNSi^+$ $[M+NH_4]^+$: 1036.3946; found: 1036.3968.

Allyl-(2,3,4-tri-*O*-benzoyl-6-deoxy-6-fluoro-β-D-glucopyranosyl)-(1→4)-2,3,6-tri-*O*-benzoyl-β-D-glucopyranoside (32)

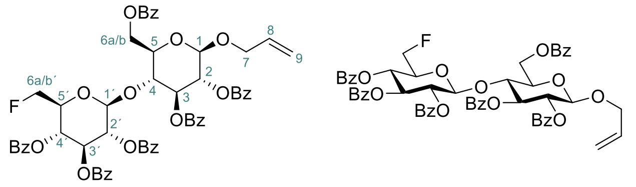

To a stirred solution of disaccharide **31** (480 mg, 0.47 mmol, 1.0 eq.) in MeOH/CH_2Cl_2 (30 ml, v/v = 1:1), *p*-TsOH (90.0 mg, 0.47 mmol, 1.0 eq.) was added. The reaction was stirred at 50 °C until the complete conversion of the starting material was observed by the TLC monitoring. The reaction was neutralized by the addition of NEt_3 (200 µl) and concentrated to dryness under reduced pressure. The crude residue was dissolved in CH_2Cl_2 (30 ml), washed with 1 M HCl (15 ml), sat. aq. $NaHCO_3$ (15 ml), and brine (15 ml), before it was dried with $MgSO_4$. The crude residue was subjected to flash column chromatography (^{c}Hex/EtOAc v/v = 2:1) and fractions containing the primary alcohol were combined for the subsequent reaction. This

material was dissolved in dry pyridine (15 ml) and benzoyl chloride (128 µl, 1.06 mmol, 3.0 eq.) was added dropwise. The reaction was stirred at 50 °C until the TCL monitoring indicated the complete conversion of the starting material. Then, dimethylaminopropyl amine (DMAPA, 140 µl, 1.12 mmol, 2.4 eq.) was added and the reaction was stirred for 20 min at ambient temperature. The organic solvents were removed under reduced pressure and the crude residue was dissolved in CH_2Cl_2 (50 ml) and washed with 1 M HCl (25 ml) and brine (25 ml). The organic phase was dried with $MgSO_4$ and the crude product was subjected to flash column chromatography (^{c}Hex/EtOAc v/v = 3:1) to obtain **32** (330 mg, 0.33 mmol, 70 % over two steps) as a colorless foam.

R*f* = 0.40 (^{c}Hex/EtOAc v/v = 2:1); **RP-HPLC** (Luna**,** 0.1 % TFA; 0 min 50 % B → 10 min 100 % B, flow: 1ml/min): t_R = 13.95 min, λ = 230 nm; $[\boldsymbol{\alpha}]_D^{22}$ = + 28.3 ° (c = 0.66; $CHCl_3$); **^{1}H NMR** (400 MHz, $CDCl_3$): δ [ppm] = 8.10 – 7.99 (m, 4H, Ar-H), 7.93 (ddd, $J_{CH,CH}$ = 8.0 Hz, $J_{CH,CH}$ = 6.4 Hz, $J_{CH,CH}$ = 1.4 Hz, 4H, Ar-H), 7.86 – 7.78 (m, 2H, H-Ar), 7.77 – 7.70 (m, 2H, H-Ar), 7.67 – 7.57 (m, 1H, H-Ar), 7.56 – 7.44 (m, 5H, H-Ar), 7.44 – 7.20 (m, 12H, H-Ar), 5.80 – 5.65 (m, 3H, H-8, H-3′, H-3), 5.49 – 5.39 (m, 2H, H-2, H-2′), 5.20 (t, $J_{H4',H3'}$ = $J_{H4',H5'}$ = 9.6 Hz, 1H, H-4′), 5.19 – 5.10 (m, 1H, H-9_{trans}), 5.06 (dq, $J_{H9cis,H8}$ = 10.4 Hz, $J_{H9cis,H9trans}$ = $J_{H9cis,H7}$ = 1.4 Hz, 1H, H-9_{cis}), 4.90 (d, $J_{H1',H2'}$ = 7.9 Hz, 1H, H-1′), 4.73 (d, $J_{H1,H2}$ = 7.8 Hz, 1H, H-1), 4.60 (dd, $J_{H6a, H6b}$ = 12.1 Hz, $J_{H6a, H5}$ = 1.9 Hz, 1H, H-6a), 4.44 (dd, $J_{H6b, H6a}$ = 12.1 Hz, $J_{H6b, H5}$ = 4.6 Hz 1H, H-6b), 4.33 – 4.19 (m, 2H, H-4, H-7a), 4.10 – 3.61 (m, 5H, H-5, H-5′, H6a′, H-6b′, H-7b); **^{13}C NMR** (100 MHz, CDCl3): δ [ppm] = 166.0, 165.7, 165.6, 165.4, 165.2, 164.8 (6 × C=O), 133.7 (2C, C-Ar, C-8), 133.5, 133.4 (2C), 133.3 (2C), 130.1, 130.0, 129.9 (4C), 129.8, 129.5, 128.8, 128.7 (3C), 128.6 (2C), 128.5 (2C), 128.4 (23 × C-Ar), 117.8 (C-9), 100.9 (C-1′), 99.7 (C-1), 80.9 (d, $J_{C6',F}$ = 175.8 Hz, C-6′), 76.5 (C-4), 73.5 (d, $J_{C5',F}$ = 20.3 Hz, C-5′), 73.1 (2C, C-3/C-3′, C-5), 72.9 (C-3/C-3′), 72.0 (2C, C-2, C-2′), 70.1 (C-7), 68.7 (d, $J_{C4',F}$ = 7.0 Hz, C-4′), 62.6 (C-6). Due to signal overlap, 44 out of 57 C atoms were assigned; **^{19}F NMR** (377 MHz, $CDCl_3$): δ [ppm] = -228.7 (td, $J_{F,H6a'}$ = $J_{F,H6b'}$ = 46.6 Hz, $J_{F,H5'}$ = 19.2 Hz); **HRMS** (ESI$^+$): Calculated for $C_{57}H_{57}O_{16}FN^+$ $[M+NH_4]^+$: 1026.3343; found: 1026.3354.

2,3,4-Tri-*O*-benzoyl-6-deoxy-6-fluoro-β-D-glucopyranosyl-(1→4)-2,3,6-tri-*O*-benzoyl-*α*/β-D-glucopyranoside (33)

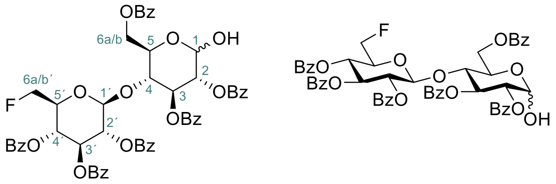

To a stirred solution of **32** (330 mg, 0.33 mmol, 1.0 eq.) in MeOH/THF (25 ml, v/v = 4:1), palladium(II) chloride (59.0 mg, 0.33 mmol, 1.0 eq.) was added. The reaction was stirred for 3 h at 60 °C, before the organic solvents were removed under reduced pressure. The crude residue was dissolved in CH_2Cl_2 (50 ml) and washed with H_2O (25 ml) and brine (20 ml), before it was dried with $MgSO_4$. The crude product was subjected to flash column chromatography (^{c}Hex/EtOAc v/v = 5:2) to obtain **33** (305 mg, 320 µmol, 97 %, *α*/β ~ 3:1) as an anomeric mixture.

***α*-32**: **R*f* =** 0.11 (^{c}Hex/EtOAc v/v = 3:1 + 1 % NEt_3); **RP-HPLC** (Luna**,** 0.1 % TFA; 0 min 50 % B → 10 min 100 % B, flow: 1 ml/min): t_R = 12.91 min, *λ* = 230 nm; **^{1}H NMR** (800 MHz, $CDCl_3$): δ [ppm] = 8.11 – 8.08 (m, 2H, Ar-H), 8.03 – 8.00 (m, 2H, Ar-H), 7.99 – 7.97 (m, 2H, Ar-H), 7.94 – 7.90 (m, 2H, Ar-H), 7.84 – 7.80 (m, 2H, Ar-H), 7.76 – 7.71 (m, 2H, Ar-H), 7.54 – 7.21 (m, 18H, Ar-H), 6.11 (dd, $J_{H3,H4}$ = 10.2 Hz, $J_{H3,H2}$ = 9.1 Hz, 1H, H-3), 5.74 (t, $J_{H3',H4'}$ = $J_{H3',H2'}$ = 9.7 Hz, 2H, H-3′), 5.60 (d, $J_{H1,H2}$ = 3.7 Hz, 1H, H-1), 5.47 (dd, $J_{H2',H3'}$ = 9.7 Hz, $J_{H2',H1'}$ = 7.9 Hz, 1H, H-2′), 5.26 – 5.18 (m, 2H, H-2, H-4′), 4.96 (d, $J_{H1',H2'}$ = 7.8 Hz, 1H, H-1′), 4.59 (dd, $J_{H6a,H6b}$ = 12.1 Hz, $J_{H6a,H5}$ = 1.9 Hz, 1H, H-6a), 4.44 (dd, $J_{H6b,H6a}$ = 12.1 Hz, $J_{H6b,H5}$ = 3.8 Hz, 1H, H-6b), 4.35 – 4.31 (m, 1H, H-5), 4.24 (t, $J_{H4,H3}$ = $J_{H4,H5}$ = 9.6 Hz, 1H, H-4), 4.12 – 4.04 (m, 1H, H-6a′), 3.87 (ddd, $J_{H6b',F}$ = 47.2 Hz, $J_{H6b',H6a'}$ = 10.3 Hz, $J_{H6b',H5'}$ = 5.8 Hz, 1H, H-6b′), 3.74 – 3.68 (m, 1H, H-5′), 3.30 (s, 1H, OH); **^{13}C NMR** (200 MHz, $CDCl_3$): δ [ppm] = 166.1, 166.0, 165.8, 165.5, 165.2, 164.8 (6 × C=O), 133.7, 133.5 (3C), 133.4, 133.2, 130.1, 130.0, 129.9 (2C), 129.8 (2C), 129.1, 128.7 (2C), 128.6 (3C), 128.4 (2C, 20 × C-Ar), 100.8 (C-1′), 90.4 (C-1), 81.0 (d, $J_{C6',F}$ = 175.5 Hz, C-6′), 76.3 (C-4) , 73.5 (d, $J_{C5',F}$ = 20.2 Hz, C-5′), 72.9 (C-3′), 72.3 (C-2), 72.0 (C-2′), 70.1 (C-3), 68.7 (d, $J_{C4',F}$ = 7.0 Hz, C-4′), 68.6 (C-5), 62.4 (C-6). Due to signal overlap, 38 out of 54 C atoms were assigned; **^{19}F NMR** (377 MHz, $CDCl_3$): δ [ppm] = -228.6 (td, $J_{F,H6a}$ = $J_{F,H6b}$ = 46.7 Hz, $J_{F,H5}$ = 18.9 Hz); **HRMS** (ESI$^+$): Calculated for $C_{54}H_{49}O_{16}NF$ $[M+NH_4]^+$: 986.3030; found: 986.3039.

β-33: **R*f* =** 0.11 (^{c}Hex/EtOAc v/v = 3:1 + 1 % NEt_3); **RP-HPLC** (Luna, 0.1 % TFA; 0 min 50 % B → 10 min 100 % B, flow: 1 ml/min): t_R = 12.66 min, *λ* = 230 nm; **^{1}H-NMR** (800 MHz, anomeric

signals, $CDCl_3$): δ [ppm] = 4.93 – 4.89 (m, 2H, H-1, H-1′); **^{13}C-NMR** (200 MHz, anomeric signals, $CDCl_3$): δ [ppm] = 100.8 (C-1′), 95.9 (C-1); **^{19}F-NMR** (377 MHz, $CDCl_3$): δ [ppm] = -228.3 – -228.5 (m).

2,3,4-Tri-*O*-benzoyl-6-deoxy-6-fluoro-β-D-glucopyranosyl-(1→4)-2,3,6-tri-*O*-benzoyl-*α*-D-glucopyranosyl trichloroacetimidate (14)

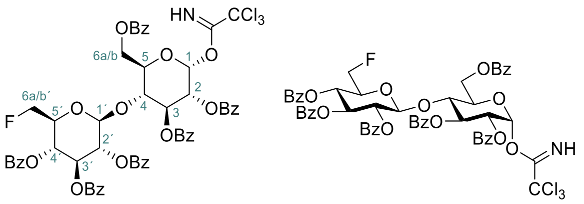

To an ice-cooled stirred solution of **33** (220 mg, 0.23 mmol, 1.0 eq.) in dry CH_2Cl_2 (24 ml), trichloroacetonitrile (231 µl, 2.30 mmol, 10.0 eq.) and DBU (35.0 µl, 0.23 mmol, 1.0 eq.) were added. The reaction mixture was stirred for 10 min at 0 °C, before it was slowly warmed to ambient temperature. The mixture was stirred for further 17 h. The organic solvents were concentrated under reduced pressure and the crude residue was dissolved CH_2Cl_2 (500 µl). Flash column chromatography (^{c}Hex/EtOAc v/v = 3:1 + 1 % NEt_3) provided **14** (220 mg, 0.20 mmol, 87 %) as a slightly yellow oil.

R*f* = 0.27 (^{c}Hex/EtOAc v/v = 3:1 + 1 % NEt_3); $[\alpha]_D^{23}$ = + 48.7 ° (c = 1.2; $CHCl_3$); **^{1}H NMR** (400 MHz, CD_2Cl_2): δ [ppm] = 8.61 (s, 1H, N=H), 8.12 – 8.07 (m, 2H, Ar-H), 8.02 – 7.97 (m, 2H, Ar-H), 7.95 – 7.88 (m, 4H, Ar-H), 7.85 – 7.80 (m, 2H, Ar-H), 7.75 – 7.70 (m, 2H, Ar-H), 7.67 – 7.60 (m, 1H, Ar-H), 7.60 – 7.32 (m, 13H, Ar-H), 7.32 – 7.23 (m, 4H, Ar-H), 6.66 (d, $J_{H1,H2}$ = 3.8 Hz, 1H, H-1), 6.09 (dd, $J_{H3,H2}$ =10.2 Hz, $J_{H3,H4}$ = 9.0 Hz, 1H, H-3), 5.76 (t, $J_{H3',H2'}$ = $J_{H3',H4'}$ = 9.6 Hz, 1H, H-3′), 5.49 (dd, $J_{H2,H3}$ = 10.2 Hz, $J_{H2,H1}$ = 3.8 Hz, 1H, H-2), 5.43 (dd, $J_{H2',H3'}$ = 9.8 Hz, $J_{H2',H1'}$ = 7.9 Hz, 1H, H-2′), 5.28 (t, $J_{H4',H3'}$ = $J_{H4',H5'}$ = 9.8 Hz, 1H, H-4′), 5.03 (d, $J_{H1',H2'}$ = 7.9 Hz, 1H, H-1′), 4.63 (dd, $J_{H6a,H6b}$ = 12.3 Hz, $J_{H6a,H5}$ = 1.9 Hz, 1H, H-6a), 4.44 (dd, $J_{H6b,H6a}$ = 12.3 Hz, $J_{H6b,H5}$ = 4.0 Hz, 1H, H-6b), 4.37 (dd, $J_{H4,H5}$ = 10.2 Hz, $J_{H4,H3}$ = 9.0 Hz, 1H, H-4), 4.33 – 4.27 (m, 1H, H-5), 4.09 (ddd, $J_{H6a',F}$ = 46.8 Hz, $J_{H6a',H6b'}$ = 10.5 Hz, $J_{H6a',H5'}$ = 2.3 Hz, 1H, H-6a′), 3.91 (ddd, $J_{H6b',F}$ = 47.1 Hz, $J_{H6b',H6a'}$ = 10.5 Hz, $J_{H6b',H5'}$ = 5.1 Hz, 1H, H-6b′), 3.75 (dddd, $J_{H5',F}$ = 20.7 Hz, $J_{H5',H4'}$ = 9.9 Hz, $J_{H5',H6b'}$ = 5.1 Hz, $J_{H5',H6a'}$ = 2.2 Hz, 1H, H-5′); **^{13}C NMR** (200 MHz, CD_2Cl_2): δ [ppm] = 166.1, 166.0, 165.9, 165.8, 165.5, 165.4 (6 × C=O), 161.1 (C=NH), 134.1 (2C), 134.0, 133.9, 130.4, 130.3, 130.2 (2C), 130.1, 129.3, 129.2 (2C), 129.1, 129.0 (2C), 128.9 (16 × C_{Ar}), 101.4 (C-1′), 93.5 (C-1), 91.2 (CCl_3), 81.3 (d, $J_{C6',F}$ =

175.7 Hz, C-6′), 76.4 (C-4), 73.8 (d, $J_{C5′,F}$ = 19.9 Hz, C-5′), 73.4 (C-3′), 72.5 (C-2′), 71.9 (C-5), 71.2 (C-2), 70.8 (C-3), 68.7 ($J_{C4′,F}$ = 6.9 Hz, C-4′), 62.3 (C-6). Due to signal overlap, 36 out of 56 C atoms were assigned; **^{19}F NMR** (377 MHz, CD_2Cl_2): δ [ppm] = – 230.4 (td, $J_{F,H6a′}$ = $J_{F,H6b′}$ = 46.9 Hz, $J_{F,H5′}$ = 20.9 Hz); **HRMS** (ESI$^+$): Calculated for $C_{56}H_{49}Cl_3FN_2O_{16}^+$ [M+NH$_4$]$^+$: 1129.2127; found: 1129.2144.

Synthesis of fluorinated cellobiosyl donor 15

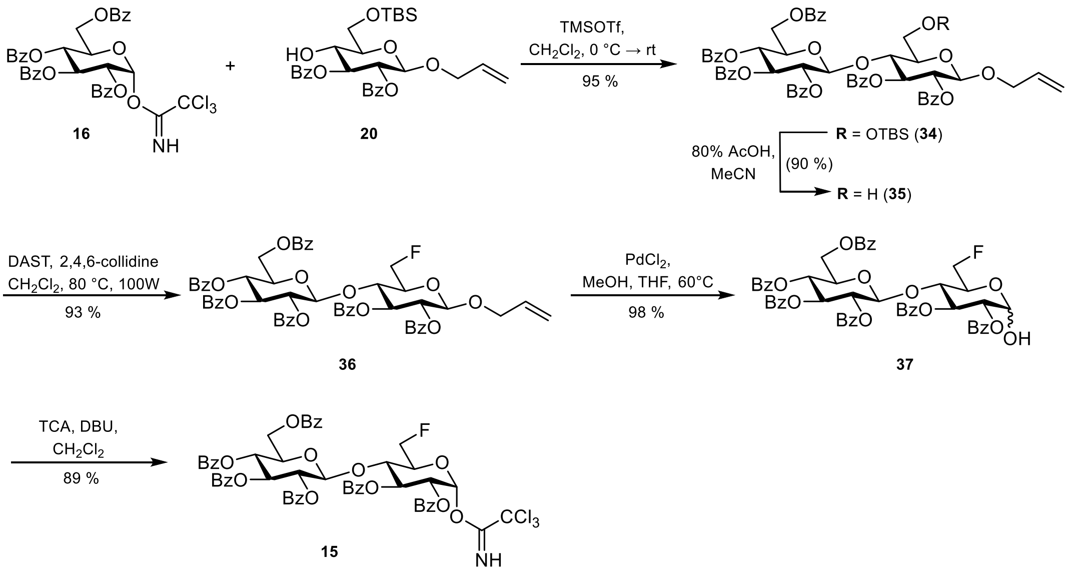

Figure S7: Synthesis of fluorinated disaccharide donor **15**.

Allyl-(2,3,4,6-tetra-*O*-benzoyl-β-D-glucopyranosyl)-(1→4)-2,3-di-*O*-benzoyl-6-*O*-*tert*-butyl-dimethylsilyl-β-D-glucopyranoside (34)

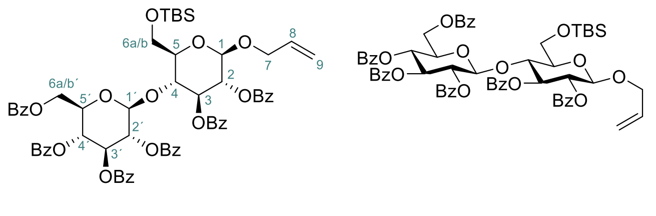

Donor **16** (1.39 g, 1.88 mmol, 1.7 eq.) and the acceptor **20** (600 mg, 1.10 mmol, 1.0 eq.) were combined, co-evaporated with toluene (2 × 10 ml) and dried under high vacuo for 2 h. The starting materials were dissolved in dry CH_2Cl_2 (15 ml) and stirred for 1 h with freshly activated MS 4 Å. Then, TMSOTf (18.0 µl, 0.11 mmol, 0.1 eq.) was added at 0°C and the reaction was allowed to slowly warm to ambient temperature. After complete conversion of

the donor was indicated by the TLC monitoring, the reaction was stopped by the addition of NEt_3 (1 ml). The mixture was diluted with CH_2Cl_2 and filtered through a pad of Celite® Hyflo Supercel. The organic phase was washed with 1 M HCl (20 ml), sat. aq. $NaHCO_3$ (20 ml) and brine (15 ml), before it was dried with $MgSO_4$. The crude product was subjected to column chromatography (^{c}Hex/EtOAc v/v = 6:1) to obtain **34** (1.18 g, 1.05 mmol, 95 %) as a colorless oil.

R*f* = 0.39 (^{c}Hex/EtOAc v/v = 3:1 + 1 % NEt_3); **RP HPLC** (Luna, 0.1 % TFA; 0 min 50 % B → 10 min 100 % B, flow: 1 ml/min): t_R = 17.91 min, λ = 230 nm; $[\boldsymbol{\alpha}]_{\mathbf{D}}^{\mathbf{24}}$ = -45.2 ° (c = 0.5, $CHCl_3$); **^{1}H NMR** (600 MHz, $CDCl_3$): δ [ppm] = 8.01 – 7.97(m, 4H, Ar-H), 7.98 – 7.94 (m, 2H, Ar-H), 7.94 – 7.90 (m, 2H, Ar-H), 7.84 – 7.81 (m, 2H, Ar-H), 7.77 – 7.75 (m, 2H, Ar-H), 7.61 – 7.56 (m, 1H, Ar-H), 7.56 – 7.51 (m, 1H, Ar-H), 7.51 – 7.44 (m, 4H, Ar-H), 7.43 – 7.37 (m, 3H, Ar-H), 7.37 – 7.33 (m, 3H, Ar-H), 7.32 – 7.28 (m, 2H, Ar-H), 7.27 – 7.23 (m, 2H, Ar-H), 7.21 – 7.18 (m, 2H, Ar-H), 5.78 – 5.67 (m, 3H, H-3, H-3′,H-8), 5.47 (dd, $J_{H2',H3'}$ = 9.7 Hz, $J_{H2',H1'}$ = 8.0 Hz, 1H, H-2′), 5.35 (dd, $J_{H2,H3}$ = 9.8 Hz, $J_{H2,H1}$ = 7.9 Hz, 1H, H-2), 5.30 (t, $J_{H4',H3'}$ = $J_{H4',H5'}$ = 9.7 Hz, 1H, H-4′), 5.16 (dq, $J_{H9trans,H8}$ = 17.3 Hz, $J_{H9trans,H9cis}$ = $J_{H9trans,H7}$ = 1.7 Hz, 1H, H-9_{trans}), 5.11 (d, $J_{H1',H2'}$ = 8.0 Hz, 1H, H-1′), 5.07 (dq, $J_{H9cis,H8}$ = 10.5 Hz, $J_{H9cis,H9trans}$ = $J_{H9cis,H7}$ = 1.4 Hz, 1H, H-9_{cis}), 4.64 (d, $J_{H1,H2}$ = 8.0 Hz, 1H, H-1), 4.27 – 4.22 (m, 2H, H-4, H-7a), 4.20 (dd, $J_{H6a', H6b'}$ = 11.9 Hz, $J_{H6a', H5'}$ = 2.8 Hz, 1H, H-6a′), 4.04 (ddt, $J_{H7b,H7a}$ = 13.4 Hz, $J_{H7b,H8}$ = 6.1 Hz, $J_{H7b,H9cis}$ = $J_{H7b,H9trans}$ = 1.4 Hz, 1H, H-7b), 3.90 (ddd, $J_{H5', H4'}$ = 9.8 Hz, $J_{H5', H6b'}$ = 6.8 Hz, $J_{H5', H6a'}$ =2.8 Hz, 1H, H-5′), 3.84 (dd, $J_{H6a,H6b}$ = 11.9 Hz, $J_{H6a,H5}$ = 2.8 Hz, 1H, H-6a), 3.75 (dd, $J_{H6b,H6a}$ = 11.9 Hz, $J_{H6b,H5}$ = 1.5 Hz, 1H, H-6b), 3.61 (dd, $J_{H6b',H6a'}$ = 12.0 Hz, $J_{H6b',H5'}$ = 6.8 Hz, 1H, H-6b′), 3.36 (ddd, $J_{H5,H4}$ = 9.6 Hz, $J_{H5,H6a}$ = 2.8 Hz, $J_{H5,H6b}$ = 1.5 Hz, 1H, H-5), 0.94 (s, 9H, Si-^{t}Bu), 0.13 (s, 3H, Si-CH_3), 0.09 (s, 3H, Si-CH_3); **^{13}C NMR** (150 MHz, $CDCl_3$): δ [ppm] = 165.9 (2C), 165.8, 165.4, 165.3, 164.7 (6 × C=O), 133.8 (C-Ar), 133.5 (2C, C-8, C-Ar), 133.4, 133.3, 133.2 133.1, 130.0 (2C), 129.9 (3C), 129.8 (2C), 129.7 (2C), 129.2, 128.9, 128.8, 128.7, 128.6, 128.5, 128.4 (2C), 128.3 (22 × C-Ar), 117.4 (C-9), 100.6 (C-1′), 99.7 (C-1), 75.4 (C-5), 75.1 (C-4), 73.4 (C3/C-3′), 73.1 (C3/C-3′), 72.6 (C-5′), 72.2 (2C, C-2, C-2′), 69.7 (C-4′), 69.6 (C-7), 63.1 (C-6′), 60.9 (C-6), 26.0 (3C, Si-^{t}Bu), 18.4 (C_q, Si-^{t}Bu), -4.9 (Si-CH_3), -5.1 (Si-CH_3). Due to signal overlap, 51 out of 63 C atoms were assigned; **HRMS** (ESI^+): Calculated for $C_{63}H_{68}O_{17}NSi^+$ $[M+NH_4]^+$: 1138.4251; found: 1138.4263.

Allyl-(2,3,4,6-tetra-*O*-benzoyl-β-D-glucopyranosyl)-(1→4)-2,3-di-*O*-benzoyl-β-D-gluco-pyranoside (35):

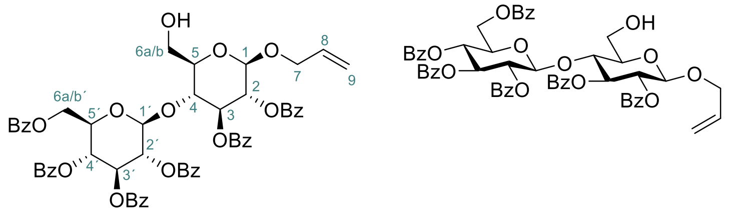

Compound **34** (1.00 g, 0.89 mmol, 1.0 eq.) was dissolved in acetonitrile (60 ml) and 80 % aqueous acetic acid (60 ml). The mixture was stirred for 20 h at 60 °C until the complete conversion of the starting material was observed by the TLC monitoring. The solvents were removed under reduced pressure and the crude product was co-evaporated with toluene (2× 50 ml), before it was subjected to flash column chromatography (^{c}Hex/EtOAc v/v = 4:1 → 1:1) to obtain **35** (806 mg, 0.80 mmol, 90 %) as a colorless oil.

R*f* = 0.12 (^{c}Hex/EtOAc v/v = 2:1); **RP-HPLC** (Luna, 0.1 % TFA; 0 min 50 % B → 10 min 100 % B, flow: 1ml/min): t_R = 13.40 min, λ = 230 nm; $[\alpha]_D^{22}$ = -8.1 ° (c = 1.1, $CHCl_3$); **^{1}H NMR** (600 MHz, $CDCl_3$): δ [ppm] = 7.96 (ddd, $J_{CH,CH}$ = 10.8 Hz, $J_{CH,CH}$ = 8.3 Hz, $J_{CH,CH}$ = 1.4 Hz, 4H, Ar-H), 7.91 (dt, $J_{CH,CH}$ = 8.4, $J_{CH,CH}$ = 1.3 Hz, 4H, Ar-H), 7.76 (ddd, $J_{CH,CH}$ = 16.5, $J_{CH,CH}$ = 8.3 Hz, $J_{CH,CH}$ = 1.4 Hz, 4H, Ar-H), 7.57 – 7.51 (m, 2H, Ar-H), 7.51 – 7.46 (m, 1H, Ar-H), 7.46 – 7.31 (m, 9H, Ar-H), 7.30 – 7.26 (m, 2H, Ar-H), 7.26 – 7.20 (m, 2H, Ar-H), 7.20 – 7.10 (m, 2H, Ar-H), 5.81 (t, $J_{H3',H2'}$ = $J_{H3',H4'}$ = 9.7 Hz, 1H, H-3′), 5.76 – 5.68 (m, 2H, H-3, H-8), 5.50 (dd, $J_{H2',H3'}$ = 9.9 Hz, $J_{H2',H1'}$ = 8.0 Hz, 1H, H-2′), 5.43 – 5.36 (m, 2H, H-2, H-4′), 5.18 (dq, $J_{H9trans,H8}$ = 17.3 Hz, $J_{H9trans,H9cis}$ = $J_{H9trans,H7}$ = 1.7 Hz, 1H, H-9_{trans}), 5.09 (dq, $J_{H9cis,H8}$ = 10.4 Hz, $J_{H9cis,H9trans}$ = $J_{H9cis,H7}$ = 1.4 Hz, 1H, H-9_{cis}), 5.02 (d, $J_{H1',H2'}$ =8.0 Hz, 1H, H-1′), 4.70 (d, $J_{H1,H2}$ = 8.0 Hz, 1H, H-1), 4.30 – 4.22 (m, 2H, H-4, H-7a), 4.07 – 4.01 (m, 2H, H-6a′, H-7b), 3.98 (ddd, $J_{H5',H4'}$ = 9.9 Hz, $J_{H5',H6b'}$ = 5.3 Hz, $J_{H5',H6a'}$ = 3.2 Hz, 1H, H-5′), 3.83 (dd, $J_{H6b',H6a'}$ = 11.9 Hz, $J_{H6b',H5'}$ = 5.3 Hz, 1H, H-6b′), 3.80 – 3.77 (m, 2H, H-6a, H-6b), 3.45 (dt, $J_{H5,H4}$ = 9.8 Hz, $J_{H5,H6a}$ = $J_{H5,H6b}$ = 2.4 Hz, 1H, H-5); **^{13}C NMR** (150 MHz, $CDCl_3$): δ [ppm] = 165.9, 165.8, 165.6, 165.3, 165.1, 164.8 (6 × C=O), 133.5 (2C, 2 × C-Ar), 133.4 (C-8), 133.3, 133.2 (2C), 129.9 (4C), 129.8, 129.7 (2C), 129.6, 129.5, 129.1, 128.9, 128.8, 128.7, 128.5 (3C), 128.4, 128.3 (21 × C-Ar), 117.8 (C-9), 101.1 (C-1′), 100.0 (C-1), 75.4 (C-4), 75.2 (C-5), 73.1 (C-3′), 72.9 (C-3), 72.3 (C-5′), 72.1 (2C, C-2, C-2′), 70.4 (C-7), 69.6 (C-4′), 62.9 (C-6′), 60.4 (C-6). Due to signal overlap, 44 out of 57 C atoms were assigned; **HRMS** (ESI$^+$): Calculated for $C_{57}H_{54}O_{17}N^+$ $[M+NH_4]^+$: 1024.3386; found: 1024.3405.

Allyl-(2,3,4,6-tetra-*O*-benzoyl-β-D-glucopyranosyl)-(1→4)-2,3-di-*O*-benzoyl-6-deoxy-6-fluoro-β-D-gluco pyranoside (36)

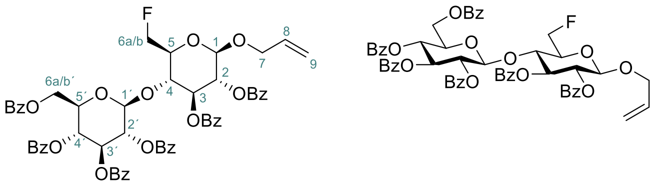

To a stirred solution of **35** (620 mg, 617 µmol, 1.0 eq.) in dry CH_2Cl_2 (6 ml), 2,4,6-collidine (206 µl, 1.55 mmol, 2.5 eq.) and DAST (204 µl, 1.54 mmol, 2.5 eq.) were added slowly. The reaction solution was heated in a microwave oven (100 W, 80 °C) for 1 h until complete conversion of the starting material was observed by the TLC monitoring. The reaction mixture was poured into MeOH (25 ml) and was stirred for 0.5 h at ambient temperature. The organic solvents were evaporated, and the residue was dissolved in CH_2Cl_2 (20 ml), washed with sat. aq. $NaHCO_3$ (15 ml), 1 M HCl (15 ml) and brine (10 ml) and dried with $MgSO_4$. The crude product was subjected to flash column chromatography (^{c}Hex/EtOAc v/v = 3:1) to obtain **36** (580 mg, 575 µmol, 93 %) as a colorless foam.

R*f* = 0.31 (^{c}Hex/EtOAc v/v = 3:1); **RP-HPLC** (Luna, 0.1 % TFA; 0 min 50 % B → 10 min 100 % B, flow: 1ml/min): t_R = 13.95 min, λ = 230 nm; $[\boldsymbol{\alpha}]_{\mathbf{D}}^{\mathbf{22}}$ = − 45.3 ° (c = 0.5; $CHCl_3$); **^{1}H NMR** (400 MHz, $CDCl_3$): δ [ppm] = 8.01 – 7.85 (m, 8H, Ar-H), 7.75 (td, $J_{CH,CH}$ = 8.6 Hz, $J_{CH,CH}$ = 1.4 Hz, 4H, Ar-H), 7.59 – 7.13 (m, 18H, Ar-H), 5.82 – 5.64 (m, 3H, H-3, H-3′, H-8), 5.51 (dd, $J_{H2',H3'}$ = 9.8 Hz, $J_{H2',H1'}$ = 7.9 Hz, 1H, H-2′), 5.44 – 5.36 (m, 2H, H-2, H-4′), 5.16 (dt, $J_{H9trans,H8}$ = 17.3 Hz, $J_{H9trans,H7}$ = 1.7 Hz, 1H, H-9$_{trans}$), 5.07 (dq, $J_{H9cis,H8}$ = 10.5 Hz, $J_{H9cis,H7}$ = 1.5 Hz, 1H, H-9$_{cis}$), 4.94 (d, $J_{H1',H2'}$ = 8.0 Hz, 1H, H-1′), 4.67 (d, $J_{H1,H2}$ = 7.9 Hz, 1H, H-1), 4.65 – 4.45 (m, 2H, H-6a, H-6b), 4.28 (ddt, $J_{H7a,H7b}$ = 13.3 Hz, $J_{H7a,H8}$ = 4.8 Hz, $J_{H7a,H9trans}$ = $J_{H7a,H9cis}$ = 1.6 Hz, 1H, H-7a), 4.20 (t, $J_{H4,H5}$ = $J_{H4,H3}$ = 9.6 Hz, 1H, H-4), 4.08 – 3.96 (m, 2H, H-7b, H-6a′), 3.92 (ddd, $J_{H5',H4'}$ = 9.9 Hz, $J_{H5',H6b'}$ = 5.1, $J_{H5',H6a'}$ = 3.1 Hz, 1H, H-5′), 3.77 (dd, $J_{H6b',H6a'}$ = 11.9 Hz, $J_{H6b',H5'}$ = 5.1 Hz, 1H, H-6b′), 3.55 (dd, $J_{H5,F}$ = 29.3 Hz, $J_{H5,H4}$ = 9.8 Hz, 1H, H-5); **^{13}C NMR** (100 MHz, $CDCl_3$): δ [ppm] = 165.9, 165.8, 165.5, 165.2, 165.1, 164.8 (6 × C=O), 133.7, 133.5, 133.4, 133.3 (2C, C-8, C-Ar), 129.9 (2C), 129.8, 129.7, 129.6, 129.5, 129.4, 128.9, 128.8, 128.7 (2C), 128.7, 128.6, 128.5 (2C), 128.4 (20 × C-Ar), 117.9 (C-9), 101.2 (C-1′), 99.8 (C-1), 80.8 (d, $J_{C6,F}$ = 174 Hz, C-6), 75.6 (d, $J_{C4,F}$ = 5.6 Hz, C-4), 73.9 (d, $J_{C5,F}$ = 18.9 Hz, C-5), 73.1 (C-3′), 72.8 (C-3), 72.4 (C-5′), 72.0 (C-2′), 71.9 (C-2), 70.1 (C-7), 69.4 (C-4′), 62.7 (C-6′). Due to signal overlap, 41 out of 57 C atoms were assigned; **^{19}F NMR** (377 MHz, $CDCl_3$): δ [ppm]

= -230.2 (td, $J_{F,H6a}$ = $J_{F,H6b}$ = 47.4 Hz, $J_{F,H5}$ = 29.3 Hz); **HRMS** (ESI+): Calculated for $C_{57}H_{49}O_{16}FNa^+$ $[M+Na]^+$: 1031.2897; found: 1031.2926.

2,3,4,6-tetra-*O*-benzoyl-β-D-glucopyranosyl-(1→4)-2,3-di-*O*-benzoyl-6-deoxy-6-fluoro-*α*/β-D-glucopyranoside (37)

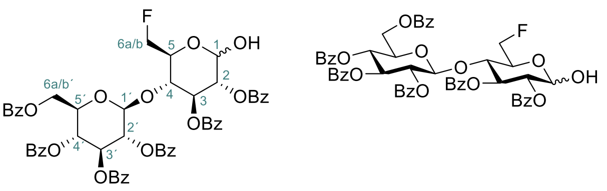

To a stirred solution of **36** (500 mg, 0.50 mmol, 1.0 eq.) in a mixture of THF/MeOH (90 ml, v/v = 1:4), palladium(II) chloride (89.0 mg, 0.50 mmol, 1.0 eq.) was added. The reaction was stirred for 3 h at 60 °C, before the organic solvents were removed under reduced pressure. The crude residue was dissolved in CH_2Cl_2 (50 ml) and washed with H_2O (15 ml) and brine (15 ml), before it was dried with $MgSO_4$. The crude product was subjected to column chromatography (^{c}Hex/EtOAc v/v = 3:1) to obtain **37** (470 mg, 490 µmol, 98 %, *α*/β = 5:1) as a colorless foam.
R*f* = 0.14 (^{c}Hex/EtOAc v/v = 3:1); **RP-HPLC** (Luna**,** 0.1 % TFA; 0 min 50 % B → 10 min 100 % B, flow: 1ml/min, *λ* = 230 nm): t_R = 12.97 min (***α*-37**), t_R = 12.65 min (**β-37**); **^{1}H NMR** (600 MHz, $CDCl_3$, *α*-anomer): δ [ppm] = 8.00 – 7.97 (m, 2H, Ar-H), 7.95 – 7.92 (m, 2H, Ar-H), 7.92 – 7.90 (m, 2H, Ar-H), 7.81 – 7.78 (m, 2H, Ar-H), 7.77 – 7.73 (m, 2H, Ar-H), 7.58 – 7.15 (m, 20H, Ar-H), 6.09 (t, $J_{H3,H2}$ = $J_{H3,H4}$ = 9.7 Hz, 1H, H-3), 5.81 (t, $J_{H3',H2'}$ = $J_{H3',H4'}$ = 9.7 Hz, 1H, H-3′), 5.63 (t, $J_{H1,H2}$ = $J_{H1,OH}$ = 3.5 Hz, 1H, H-1), 5.54 (dd, $J_{H2',H3'}$ = 9.8 Hz, $J_{H2',H1'}$ = 8.0 Hz, 1H, H-2′), 5.46 (t, $J_{H4',H3'}$ = $J_{H4',H5'}$ = 9.4 Hz, 1H, H-4′), 5.16 (dd, $J_{H2,H3}$ = 10.0 Hz, $J_{H2,H1}$ = 3.8 Hz, 1H, H-2), 5.01 (d, $J_{H1',H2'}$ = 7.9 Hz, 1H, H-1′), 4.68 (ddd, $J_{H6a,F}$ = 47.0 Hz, $J_{H6a,H6b}$ = 10.8 Hz, $J_{H6a,H5}$ = 2.3 Hz, 1H, H-6a), 4.46 (dd, $J_{H6b,F}$ = 48.8 Hz, $J_{H6b,H6a}$ = 10.4 Hz, 1H, H-6b), 4.24 – 4.19 (m, 1H, H-4), 4.18 – 4.11 (m, 1H, H-5), 4.07 (dd, $J_{H6a',H6b'}$ = 9.7 Hz, $J_{H6a',H5'}$ = 3.9 Hz, 1H, H-6a′), 3.98 – 3.93 (m, 2H, H-6b′, H-5′), 2.92 (d, $J_{OH,H1}$ = 3.8 Hz, 1H, OH); β-anomer (600 MHz, anomeric signals, $CDCl_3$): δ [ppm] = 4.96 (d, $J_{H1',H2'}$ = 8.0 Hz, H-1′), 4.70 (d, $J_{H1,H2}$ = 8.0 Hz, H-1); **^{13}C NMR** (150 MHz, $CDCl_3$, *α*-anomer): δ [ppm] = 165.9 (3C), 165.5, 165.1, 164.8 (6 × C=O), 133.6 (2C), 133.5, 133.4, 133.3 (2C), 130.0, 129.9 (2C), 129.8, 129.7, 128.6 (2C), 128.5 (2C), 128.4 (2C, 17 × C-Ar), 101.0 (C-1′), 90.5 (C-1), 81.2 (d, $J_{C6,F}$ = 173.2 Hz, C-6), 75.5 (d, $J_{C4,F}$ = 6.0 Hz, C-4), 73.1 (C-3′), 72.4 (2C, C-2, C-5′), 72.1 (C-2′), 69.9 (C-3), 69.5 (d, $J_{C5,F}$ = 18.1 Hz, C-5), 69.4 (C-4′), 62.8 (C-6′). Due to signal overlap, 35 out of 54 C atoms were assigned; β-anomer (150 MHz, anomeric signals, $CDCl_3$): δ [ppm] = 100.1 (C-1′),

100.9 (C-1); **^{19}F NMR** (377 MHz, $CDCl_3$, α-anomer): δ [ppm] = -234.6 (td, $J_{F,H6a}$ = $J_{F,H6b}$ = 47.7 Hz, $J_{F,H5}$ = 21.4 Hz); β-anomer: δ [ppm] = -233.9 − -233.7 (m); **HRMS** (ESI$^+$): Calculated for $C_{54}H_{45}FKO_{16}^+$ [M+K]$^+$: 1007.2324; found: 1007.2345.

2,3,4,6-Tetra-*O*-benzoyl-β-D-glucopyranosyl-(1→4)-2,3-di-*O*-benzoyl-6-deoxy-6-fluoro-*α*-D-glucopyranosyl trichloroacetimidate (15)

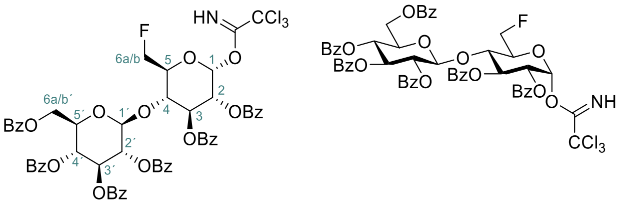

To an ice-cooled solution of **37** (430 mg, 0.44 mmol, 1.0 eq.) in dry CH_2Cl_2 (20 ml), trichloroacetonitrile (441 µl, 4.40 mmol, 10 eq.) and DBU (60.0 µl, 0.44 mmol, 1.0 eq.) were added. The reaction mixture was allowed to warm to ambient temperature and was stirred for 17 h. After the reaction was deemed complete, it was concentrated under reduced pressure (v ~ 1 ml) and the residue was subjected to column chromatography (^{c}Hex/EtOAc v/v = 1:0 + 1 % NEt_3 → 7:1 + 1 % NEt_3) to obtain **15** (430 mg, 0.39 mmol, 89 %) as a colorless oil.

R*f* = 0.33 (^{c}Hex/EtOAc v/v = 2:1 + 1 % NEt_3); $[\boldsymbol{\alpha}]_{\mathbf{D}}^{\mathbf{24}}$ = + 28.5 ° (c = 1.9; $CHCl_3$); **^{1}H NMR** (800 MHz, CD_2Cl_2): δ [ppm] = 8.62 (s, 1H, C=NH), 8.01 – 7.98 (m, 2H, Ar-H), 7.97 – 7.95 (m, 2H, Ar-H), 7.92 – 7.86 (m, 4H, Ar-H), 7.81 – 7.77 (m, 2H, Ar-H), 7.76 – 7.73 (m, 2H, Ar-H), 7.61 – 7.19 (m, 18H, Ar-H), 6.70 (d, $J_{H1,H2}$ = 3.8 Hz, 1H, H-1), 6.09 (t, $J_{H3,H2}$ = $J_{H3,H4}$ = 9.8 Hz, 1H, H-3), 5.84 (t, $J_{H3′,H2′}$ = $J_{H3′,H4′}$ = 9.6 Hz, 1H, H-3′), 5.53 – 5.44 (m, 3H, H-2, H-2′, H-4′), 5.07 (d, $J_{H1′,H2′}$ = 7.9 Hz, 1H, H-1′), 4.68 (ddd, $J_{H6a,F}$ = 46.5 Hz, $J_{H6a,H6b}$ = 10.8 Hz, $J_{H6a,H5}$ = 2.1 Hz, 1H, H-6a), 4.51 (dd, $J_{H6b,F}$ = 48.8 Hz, $J_{H6b,H6a}$ = 10.5 Hz, 1H, H-6b), 4.36 (t, $J_{H4,H3}$ = $J_{H4,H5}$ = 9.8 Hz, 1H, H-4), 4.07 (dd, $J_{H5,F}$ = 30.9 Hz, $J_{H5,H4}$ = 10.1 Hz, 1H, H-5), 4.02 (dd, $J_{H6a′,H6b′}$ = 11.7 Hz, $J_{H6a′,H5′}$ = 3.0 Hz, 1H, H-6a′), 3.98 (ddd, $J_{H5′,H4′}$ = 9.8 Hz, $J_{H5′,H6b′}$ = 4.5 Hz, $J_{H5′,H6a′}$ = 3.0 Hz, 1H, H-5′), 3.94 (dd, $J_{H6b′,H6a′}$ = 11.7 Hz, $J_{H6b′,H5′}$ = 4.5 Hz, 1H, H-6b′); **^{13}C NMR** (200 MHz, CD_2Cl_2): δ [ppm] = 166.1 (2C), 165.9, 165.7, 165.4 (2C, 6 × C=O), 161.1 (N=H), 134.1 (2C), 134.0, 133.8, 133.7, 130.5, 130.3, 130.2, 130.1, 129.4, 129.3, 129.1, 129.0, 128.9 (2C, 15 × C_{Ar}), 101.8 (C-1′), 93.6 (C-1), 91.1 (CCl_3) 81.1 (d, $J_{C6,F}$ = 173.9 Hz, C-6), 75.5 (d, $J_{C4,F}$ = 5.8 Hz, C-4), 73.6 (C-3′), 72.9 (C-5′), 72.7 (d, $J_{C5,F}$ = 18.3 Hz, C-5), 72.6 (C-2′), 71.2 (C-2), 70.6 (C-3), 69.7 (C-4′), 63.0 (C-6′). Due to signal overlap, 35 out of 56 C atoms were assigned; **^{19}F NMR** (377 MHz, CD_2Cl_2): δ [ppm] = -234.9 (td, $J_{F,H6a}$ = $J_{F,H6b}$ = 47.6 Hz,

$J_{F,H5}$ = 30.7 Hz); **HRMS** (ESI$^+$): Calculated for $C_{56}H_{49}Cl_3FN_2O_{16}^+$ [M+NH$_4$]$^+$: 1129.2127; found: 1129.2160.
