# Supplementary material for: Synthesis of Fluorinated Glycotope Mimetics Derived from Streptococcus pneumoniae Serotype 8 CPS
Source: Int J Mol Sci. 2025 Feb 12;26(4):1535. doi: 10.3390/ijms26041535 (PMC11855009; doi:10.3390/ijms26041535)
Supplement: Supplementary file 1 [file ijms-26-01535-s001.zip › Supporting Information S2_IJMolSci_SP8_spectra.pdf]

Supporting Information S2

Synthesis of fluorinated glycotope mimetics derived from *Streptococcus pneumoniae* serotype 8 CPS

Daniel Gast, Sebastian Neidig, Maximilian Reindl and Anja Hoffmann-Röder*

Department of Chemistry, Ludwig-Maximilians-Universität München, Butenandtstrasse 5-13, Haus F, 81377 Munich, Germany; anja.hoffmann-roeder@cup.lmu.de

Spectroscopic Data of selected compounds

Table of contents

N-(Benzyl)-benzyloxycarbonyl-5-aminopentyl 2,3-di-*O*-benzyl-4,6-*O*-benzylidene-*α*-D-galactopyranoside (24) 3

N-(Benzyl)-benzyloxycarbonyl-5-aminopentyl 2,3,6-tri-*O*-benzyl-*α*-D-galacto-pyranoside (18) 4

4-Methylphenyl-2-*O*-benzyl-3,4-*O*-isopropylidene-6-deoxy-6-fluoro-1-thio-*α*/β-D-galactopyranoside (26) 6

N-(Benzyl)-benzyloxycarbonyl-5-aminopentyl-2-*O*-benzyl-6-deoxy-6-fluoro-*α*-D-galacto-pyranoside (28) 8

N-(Benzyl)-benzyloxycarbonyl-5-aminopentyl-2,3-di-*O*-benzyl-6-deoxy-6-fluoro-*α*-D-galactopyranoside (19) 10

N-(Benzyl)-benzyloxycarbonyl-5-aminopentyl-(2,3-di-*O*-benzyl-4,6-*O*-benzyliden-*α*-D-glucopyranosyl)-(1→4)-2,3,6-tri-*O*-benzyl-*α*-D-galactopyranoside (29) 12

N-(Benzyl)-benzyloxycarbonyl-5-aminopentyl-(2,3,6-tri-*O*-benzyl-*α*-D-glucopyra-nosyl)-(1→4)-2,3,6-tri-*O*-benzyl-*α*-D-galactopyranoside (10) 13

N-(Benzyl)-benzyloxycarbonyl-5-aminopentyl-(2,3-di-*O*-benzyl-6-*O*-*tert*-butyldi-methylsilyl-*α*-D-glucopyranosyl)-(1→4)-2,3,6-tri-*O*-benzyl-*α*-D-galactopyranoside (11) 15

N-(Benzyl)-benzyloxycarbonyl-5-aminopentyl-(2,3,6-tri-*O*-benzyl-*α*-D-glucopyra-nosyl)-(1→4)-2,3-di-*O*-benzyl-6-deoxy-6-fluoro-*α*-D-galactopyranoside (12) 16

Allyl-(2,3,4-tri-*O*-benzoyl-6-deoxy-6-fluoro-β-D-glucopyranosyl)-(1→4)-2,3-di-*O*-benzoyl-6-*O*-*tert*-butyldimethylsilyl-β-D-glucopyranoside (31) 18

Allyl-(2,3,4-tri-*O*-benzoyl 6-deoxy-6-fluoro-β-D-glucopyranosyl)-(1→4)-2,3,6-tri-*O*-benzoyl-β-D-glucopyranoside (32) 20

Allyl-(2,3,4,6-tetra-*O*-benzoyl-β-D-glucopyranosyl)-(1→4)-2,3-di-*O*-benzoyl-6-*O*-tert-butyldimethylsilyl-β-D-glucopyranoside (34) 22

Allyl-(2,3,4,6-tetra-*O*-benzoyl-β-D-glucopyranosyl)-(1→4)-2,3-di-*O*-benzoyl-6-deoxy-6-fluoro-β-D-gluco pyranoside (36) 24

N-(Benzyl)-benzyloxycarbonyl-5-aminopentyl-(2,3,4-tri-*O*-benzoyl-6-deoxy-6-fluoro-β-D-glucopyranosyl)-(1→4)-(2,3,6-tri-*O*-benzyl-*α*-D-glucopyranosyl)-(1→4)-2,3,6-tri-*O*-benzyl-*α*-D-galactopyranoside (38) 26

5-Aminopentyl-(6-deoxy-6-fluoro-β-D-glucopyranosyl)-(1→4)-(*α*-D-glucopyranosyl)-(1→4)-*α*-D-galactopyranoside (3) 28

N-(Benzyl)-benzyloxycarbonyl-5-aminopentyl-(2,3,4,6-tetra-*O*-benzoyl-β-D-gluco-pyranosyl)-(1→4)-(2,3,6-tri-*O*-benzyl-*α*-D-glucopyranosyl)-(1→4)-2,3-di-*O*-benzyl-6-deoxy-6-fluoro-*α*-D-galactopyranoside (39) 30

5-Aminopentyl-(β-D-glucopyranosyl)-(1→4)-(*α*-D-glucopyranosyl)-(1→4)-6-deoxy-6-fluoro-*α*-D-galactopyranoside (5) 32

N-(Benzyl)-benzyloxycarbonyl-5-aminopentyl-(2,3,4,6-tetra-*O*-benzoyl-β-D-glucopyranosyl)-(1→4)-(2,3-di-*O*-benzyl-6-deoxy-6-fluoro-*α*-D-glucopyranosyl)-(1→4)-2,3,6-tri-*O*-benzyl-*α*-D-galactopyranoside (42) 34

5-Aminopentyl-(β-D-glucopyranosyl)-(1→4)-(6-deoxy-6-fluoro-*α*-D-glucopyrano-syl)-(1→4)-*α*-D-galactopyranoside (4) 36

N-(Benzyl)benzyloxycarbonyl-5-aminopentyl-(2,3,4-tri-*O*-benzoyl-6-deoxy-6-fluoro-β-D-glucopyranosyl)-(1→4)-(2,3,6-tri-*O*-benzoyl-β-D-glucopyranosyl)-(1→4)-(2,3,6-tri-*O*-benzyl-*α*-D-glucopyranosyl)-(1→4)-2,3,6-tri-*O*-benzyl-*α*-D-galactopyranoside (43) 38

5-Aminopentyl-(6-deoxy-6-fluoro-β-D-glucopyranosyl)-(1→4)-(β-D-glucopyranosyl)-(1→4)-(*α*-D-glucopyranosyl)-(1→4)-*α*-D-galactopyranoside (6) 40

N-(Benzyl)-benzyloxycarbonyl-5-aminopentyl-(2,3,4,6-tetra-*O*-benzoyl-β-D-gluco-pyranosyl)-(1→4)-(2,3-di-*O*-benzoyl-6-deoxy-6-fluoro-β-D-glucopyranosyl)-(1→4)-(2,3,6-tri-*O*-benzyl-*α*-D-glucopyranosyl)-(1→4)-2,3,6-tri-*O*-benzyl-*α*-D-galactopy-ranoside (44) 42

5-Aminopentyl-(β-D-glucopyranosyl)-(1→4)-(6-deoxy-6-fluoro-β-D-glucopyranosyl)-(1→4)-(*α*-D-glucopyranosyl)-(1→4)-*α*-D-galactopyranoside (7) 44

N-(Benzyl)benzyloxycarbonyl-5-aminopentyl-(2,3,4,6-tetra-*O*-benzoyl-β-D-gluco-pyranosyl)-(1→4)-(2,3,6-tri-*O*-benzoyl-β-D-glucopyranosyl)-(1→4)-(2,3,6-tri-*O*-ben-zyl-*α*-D-glucopyranosyl)-(1→4)-2,3-di-*O*-benzyl-6-deoxy-6-fluoro-*α*-D-galacto-pyranoside (45) 46

5-Aminopentyl-(β-D-glucopyranosyl)-(1→4)-(β-D-glucopyranosyl)-(1→4)-(*α*-D-gluco-pyranosyl)-(1→4)-6-deoxy-6-fluoro-*α*-D-galactopyranoside (9) 48

N-(Benzyl)-benzyloxycarbonyl-5-aminopentyl-(2,3,4,6-tetra-*O*-benzoyl-β-D-gluco-pyranosyl)-(1→4)-(2,3,6-tri-*O*-benzoyl-β-D-glucopyranosyl)-(1→4)-(2,3-di-*O*-ben-zyl-6-deoxy-6-fluoro-*α*-D-glucopyranosyl)-(1→4)-2,3,6-tri-*O*-benzyl-*α*-D-galacto-pyranoside (48) 50

5-Aminopentyl-(β-D-glucopyranosyl)-(1→4)-(β-D-glucopyranosyl)-(1→4)-(6-deoxy-6-fluoro-*α*-D-gluco-pryranosyl)-(1→4)-*α*-D-galactopyranoside (8) 52

N-(Benzyl)-benzyloxycarbonyl-5-aminopentyl 2,3-di-O-benzyl-4,6-O-benzylidene-α-D-galactopyranoside (24)

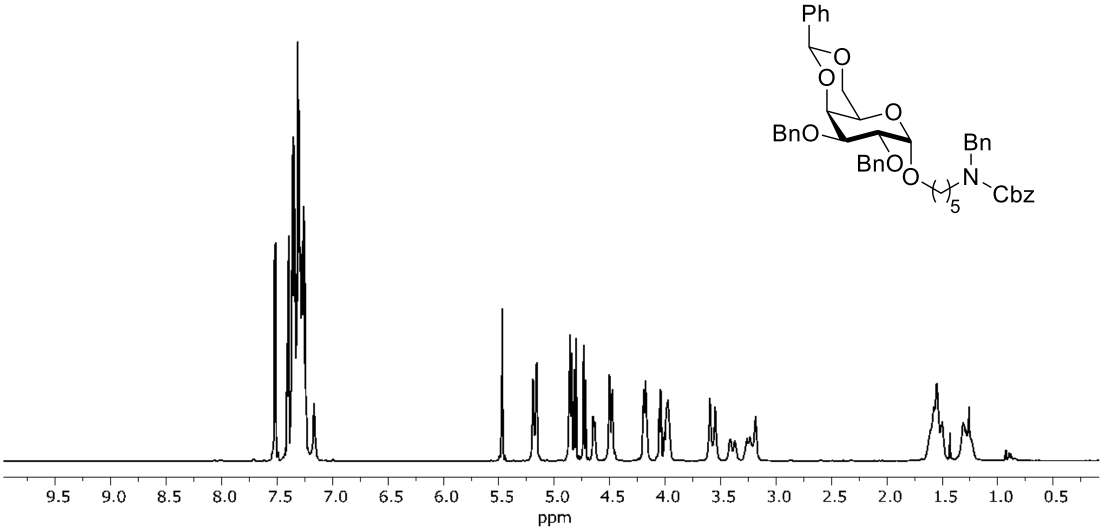

^{1}H NMR (800 MHz, $CDCl_3$) of **24**.

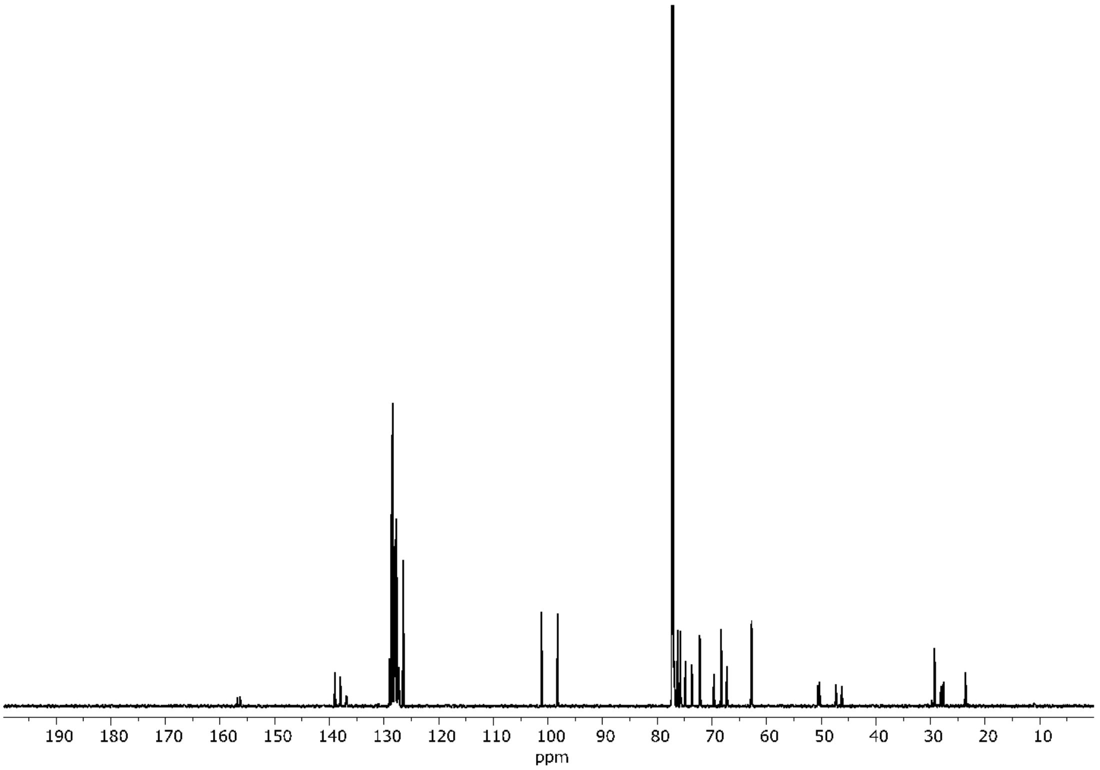

^{13}C NMR (200 MHz, $CDCl_3$) of 24.

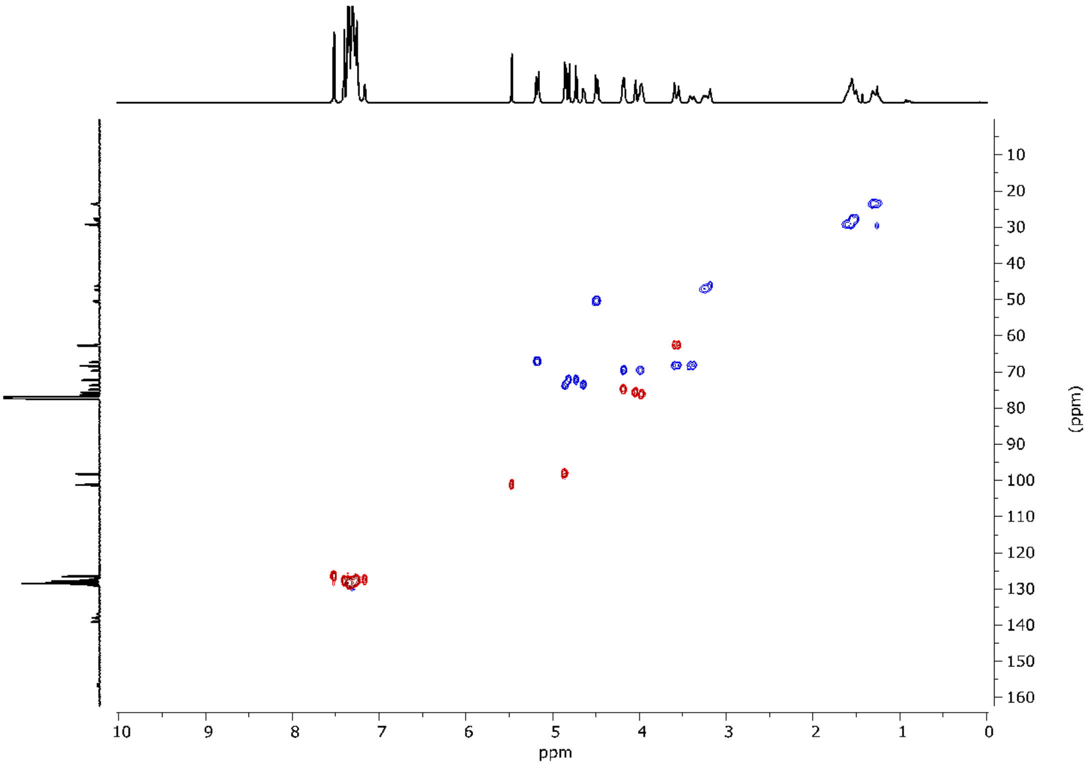

HSQC ($CDCl_3$) of **24**.

N-(Benzyl)-benzyloxycarbonyl-5-aminopentyl 2,3,6-tri-*O*-benzyl-*α*-D-galacto-pyranoside (18)

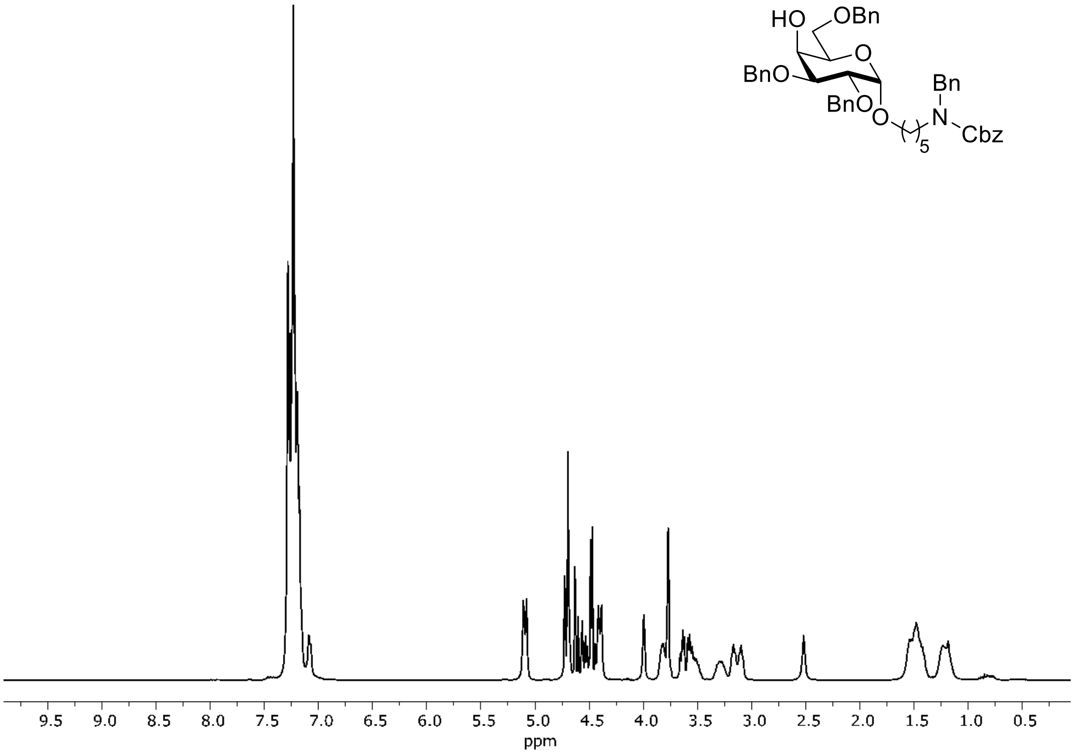

^1H NMR (600 MHz, $CDCl_3$) of **18**.

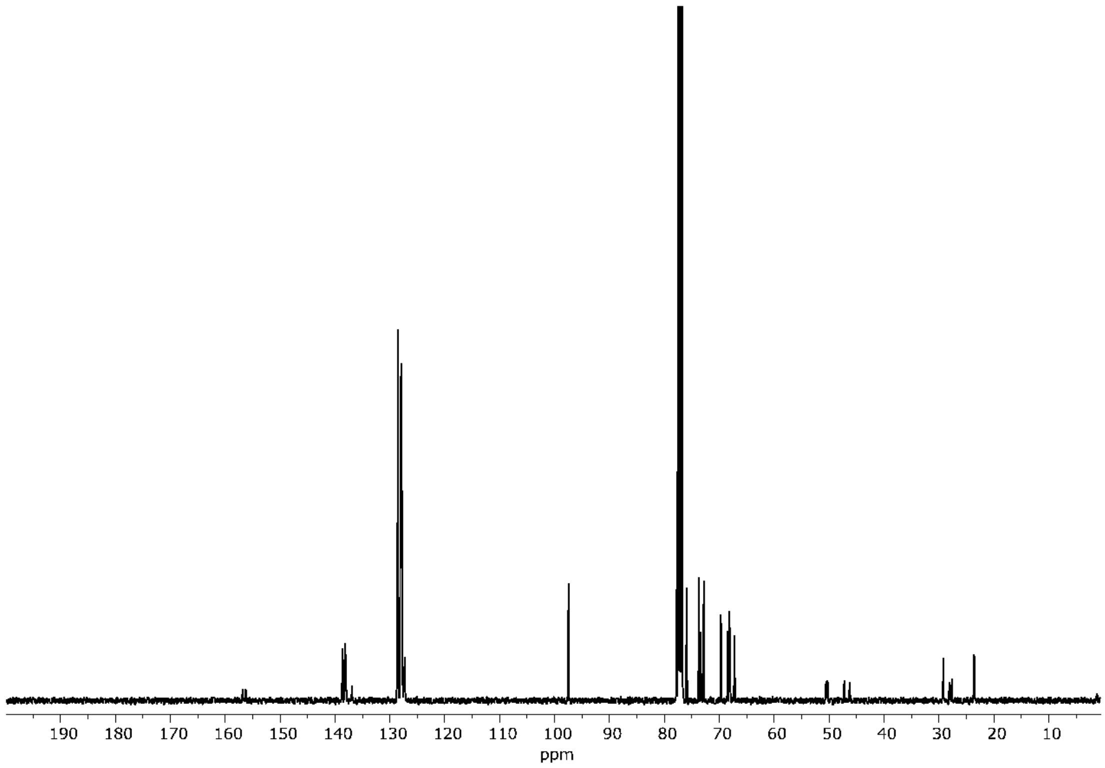

^{13}C NMR (150 MHz, $CDCl_3$) of **18**.

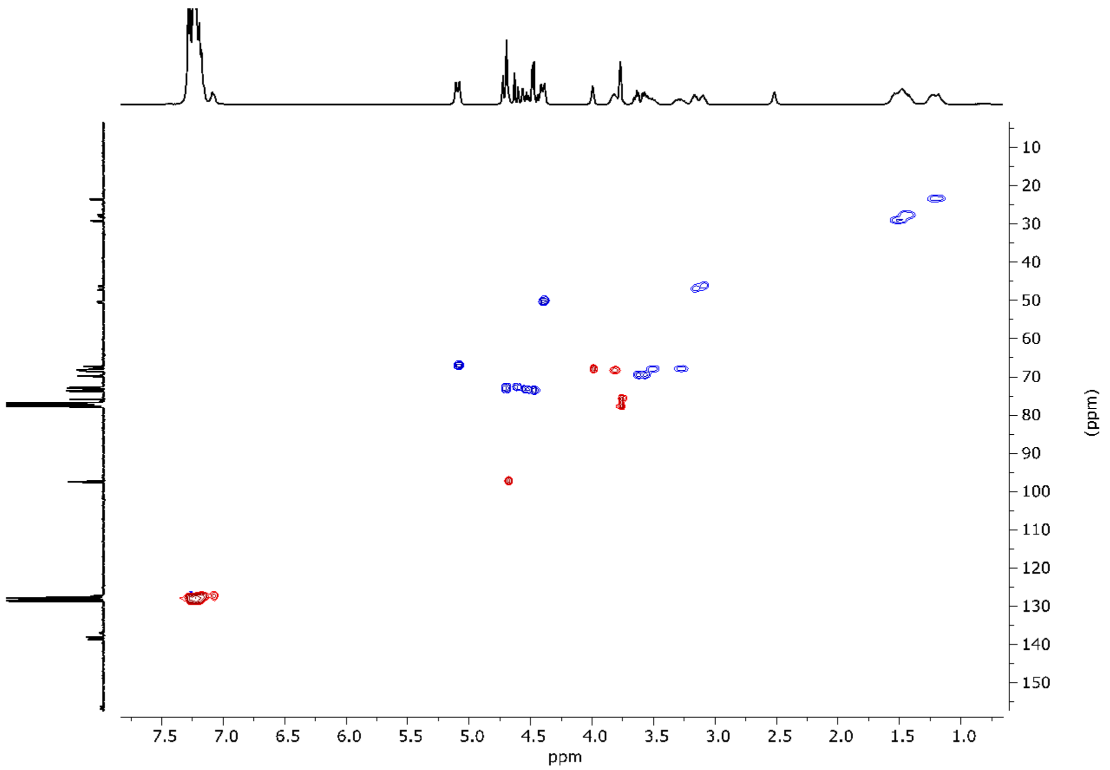

HSQC ($CDCl_3$) of **18**.

4-Methylphenyl-2-*O*-benzyl-3,4-*O*-isopropylidene-6-deoxy-6-fluoro-1-thio-*α*/β-D-galactopyranoside (26)

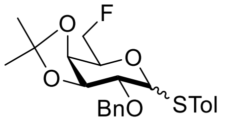

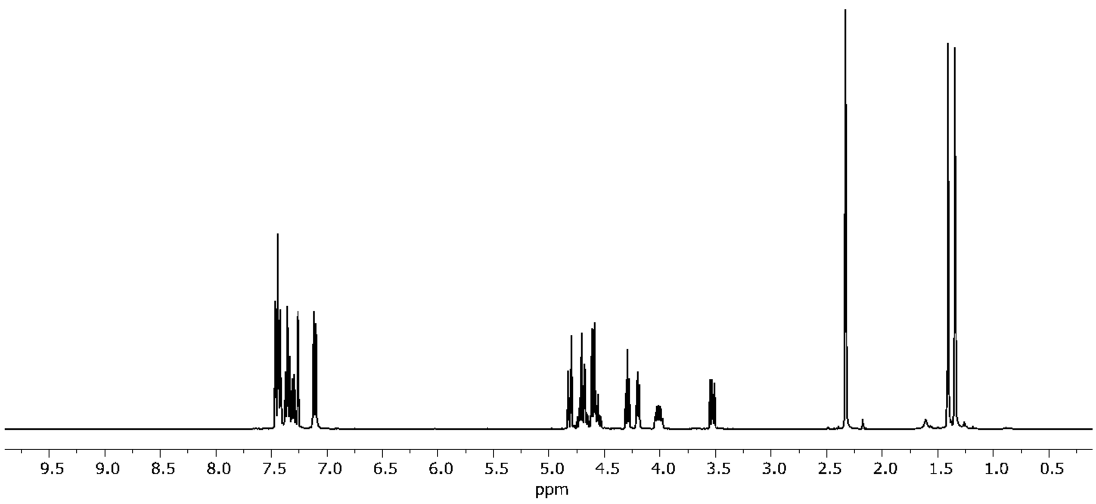

^{1}H NMR (400 MHz, $CDCl_3$) of **26**.

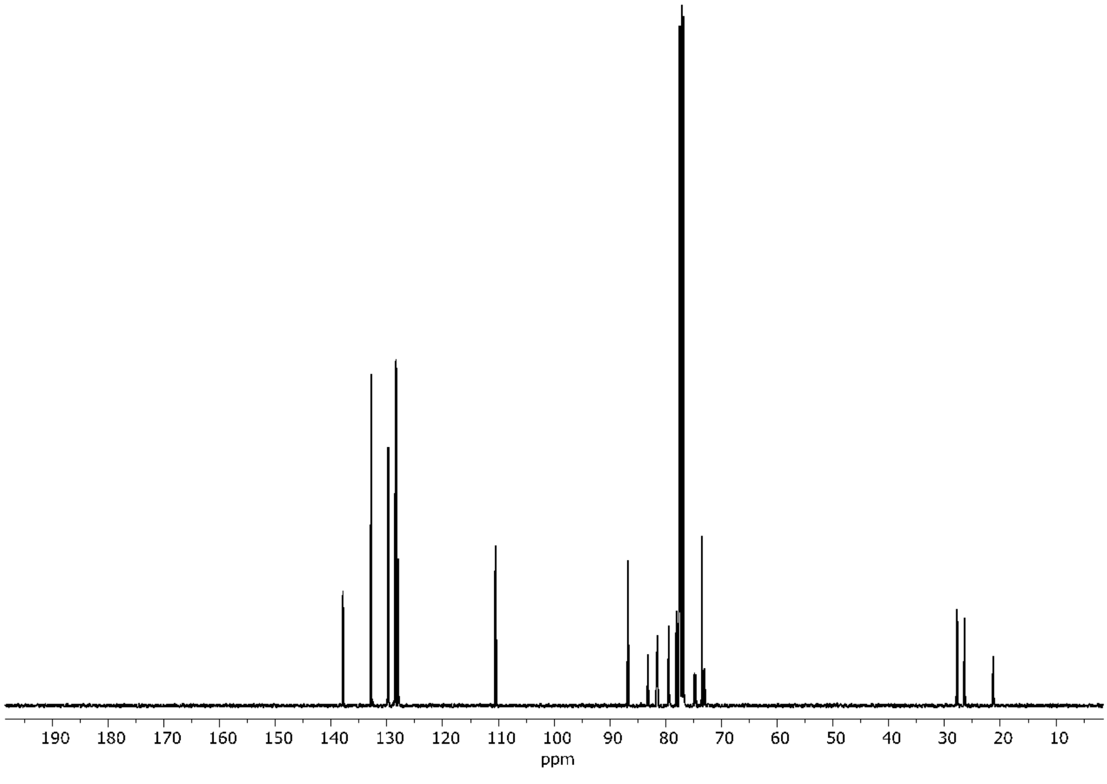

^{13}C NMR (100 MHz, $CDCl_3$) of **26**.

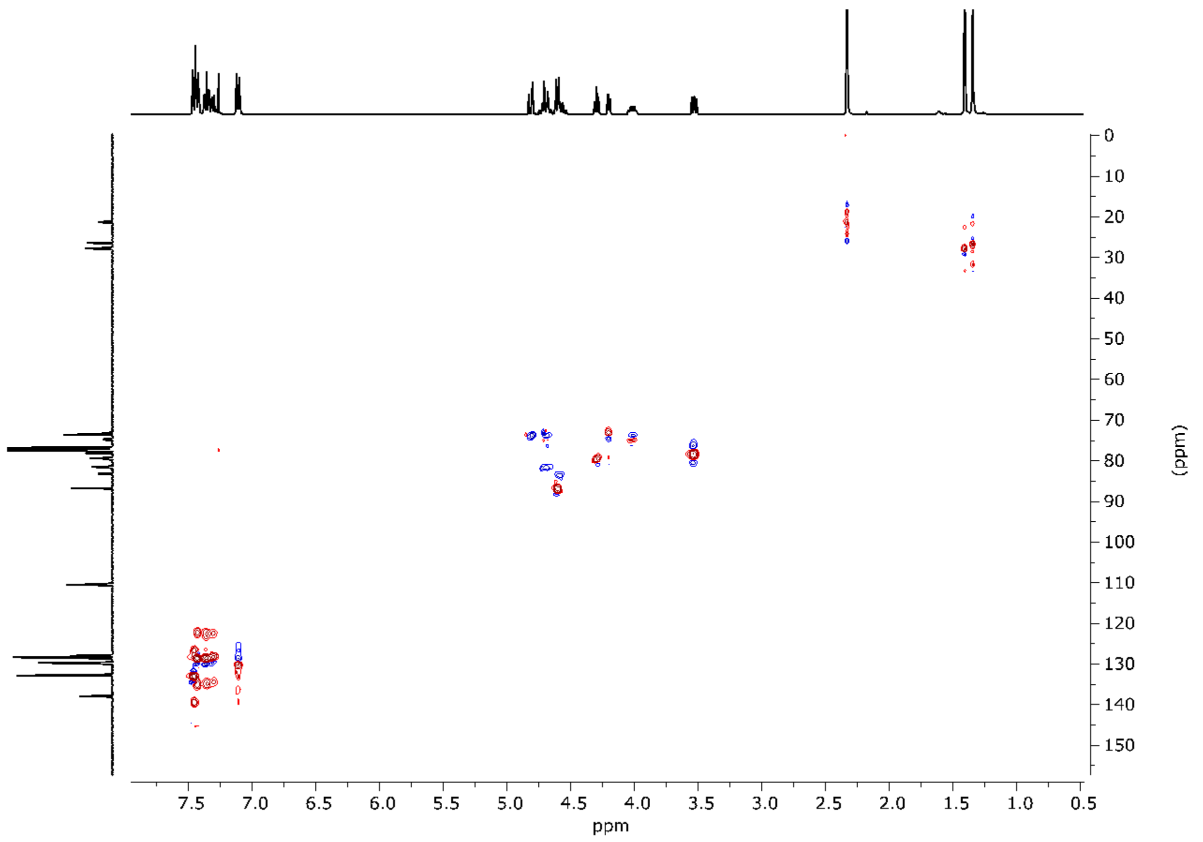

HSQC ($CDCl_3$) of **26**.

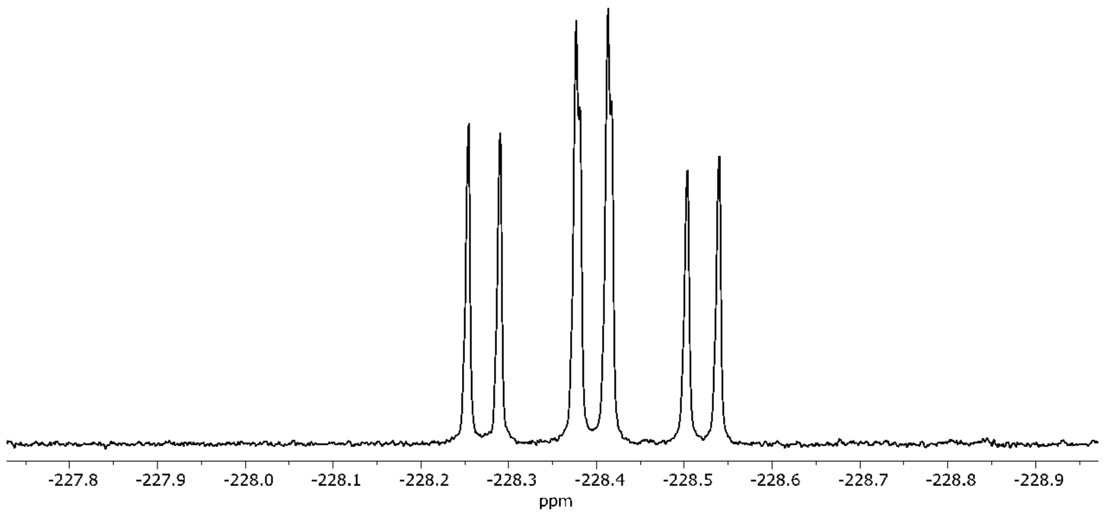

^{19}F NMR (377 MHz, $CDCl_3$) of **26**.

_N_-(Benzyl)-benzyloxycarbonyl-5-aminopentyl-2-_O_-benzyl-6-deoxy-6-fluoro-_α_-D-galacto-pyranoside (28)

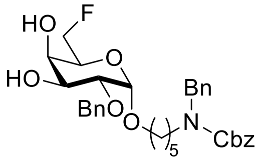

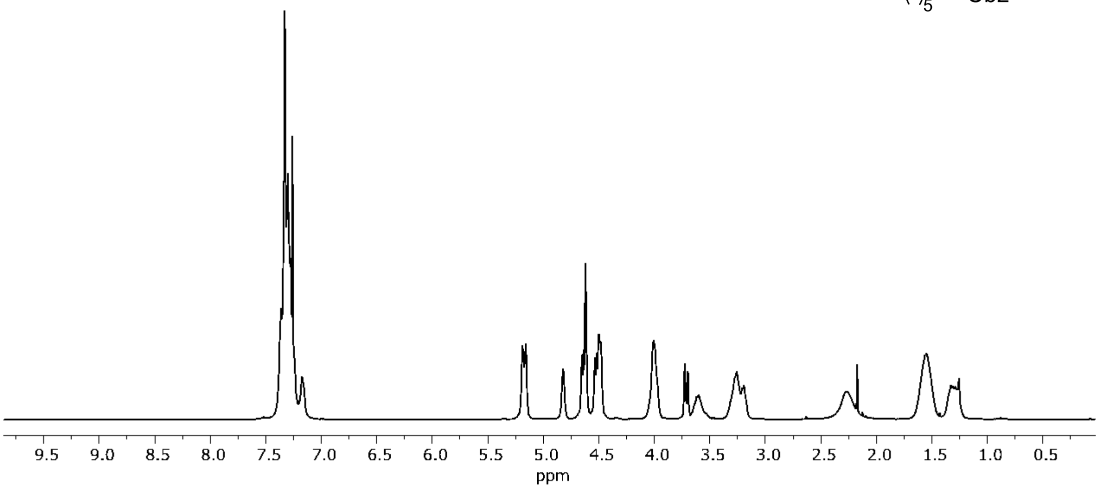

^{1}H NMR (400 MHz, $CDCl_3$) of **28**.

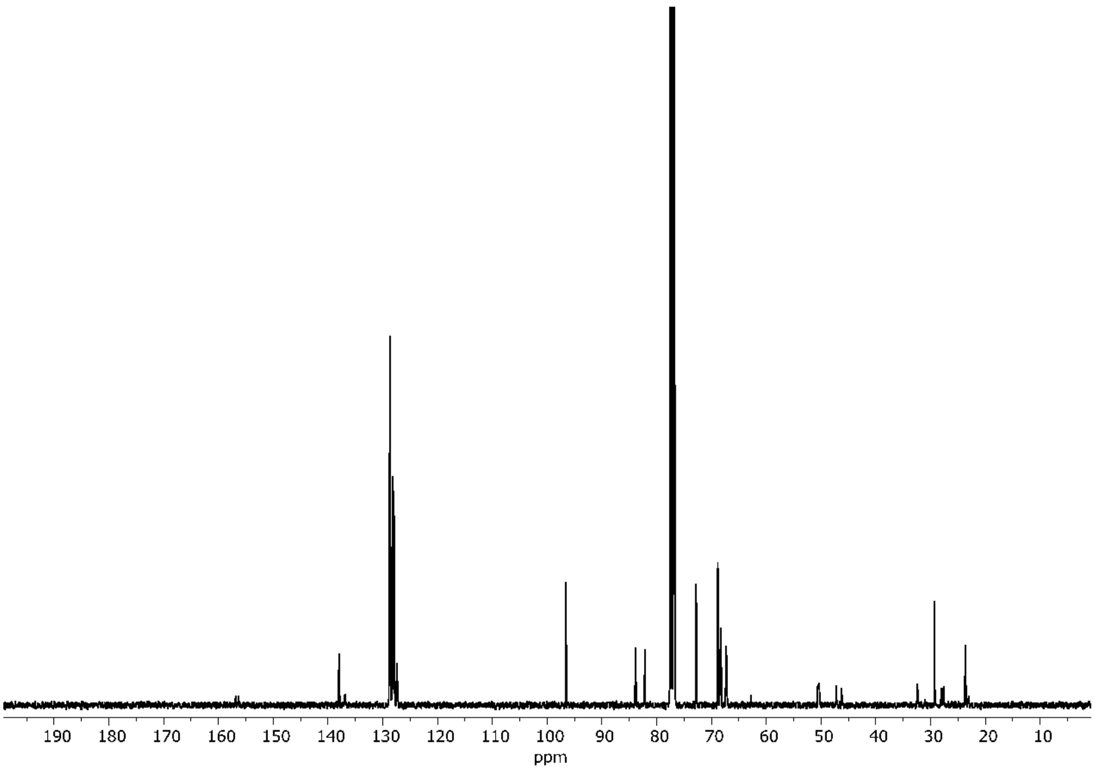

^{13}C NMR (100 MHz, $CDCl_3$) of **28**.

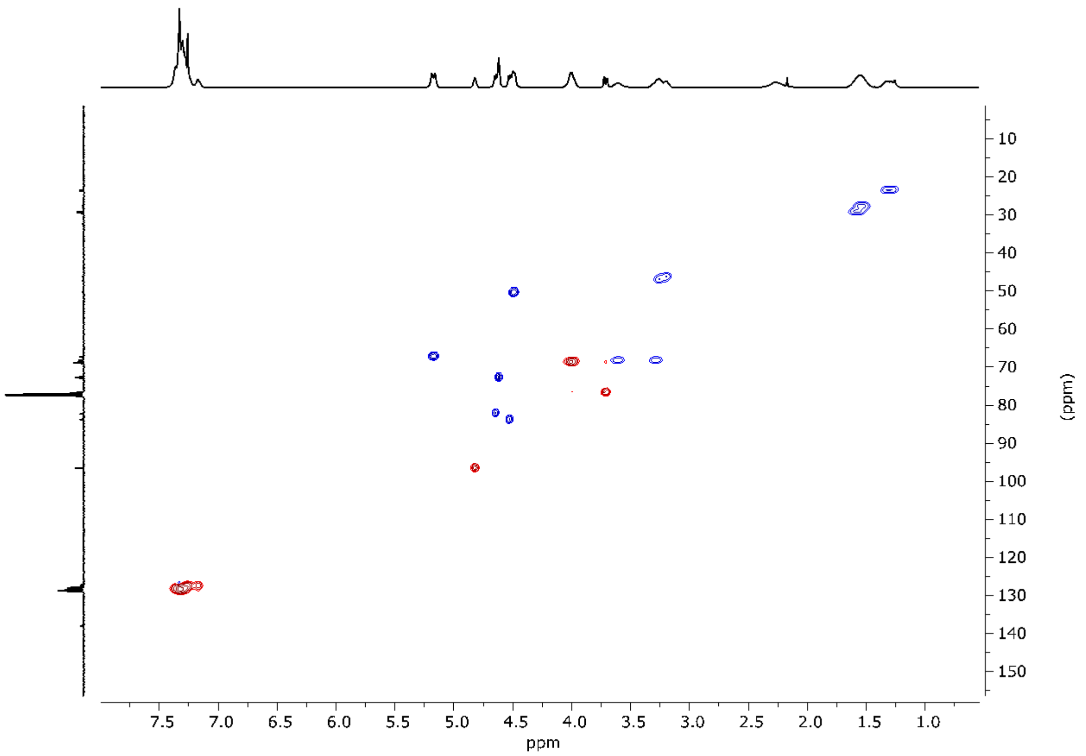

HSQC (CDCl3) of **28**.

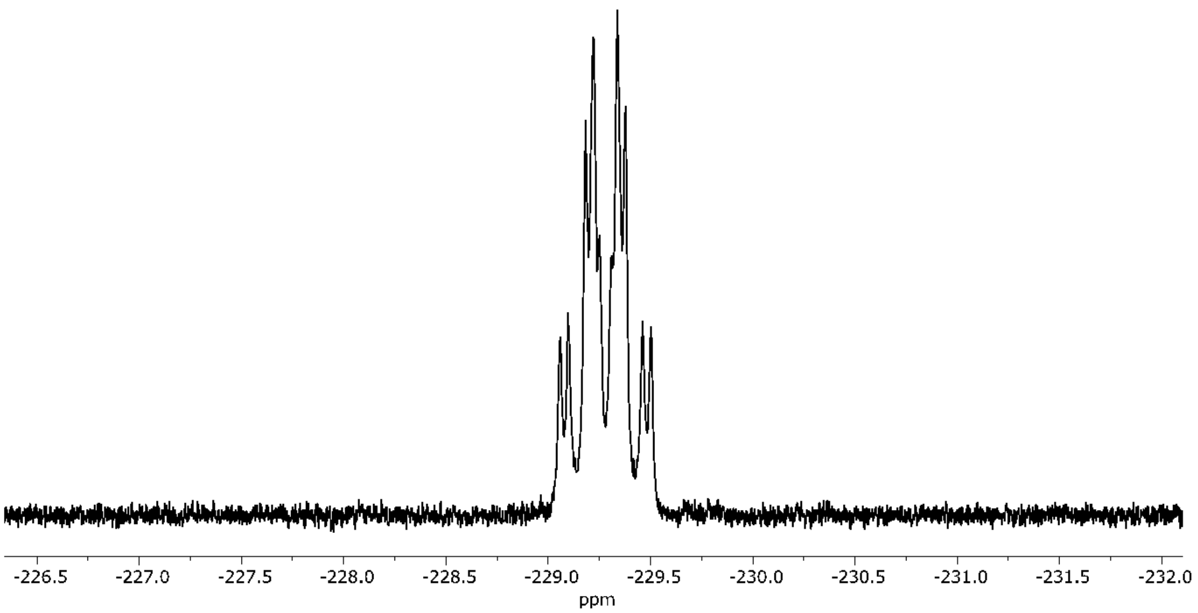

^{19}F NMR (377 MHz, CDCl3) of **28**.

N-(Benzyl)-benzyloxycarbonyl-5-aminopentyl-2,3-di-O-benzyl-6-deoxy-6-fluoro-α-D-galactopyranoside (19)

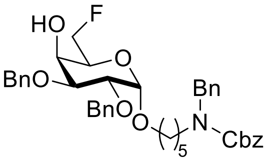

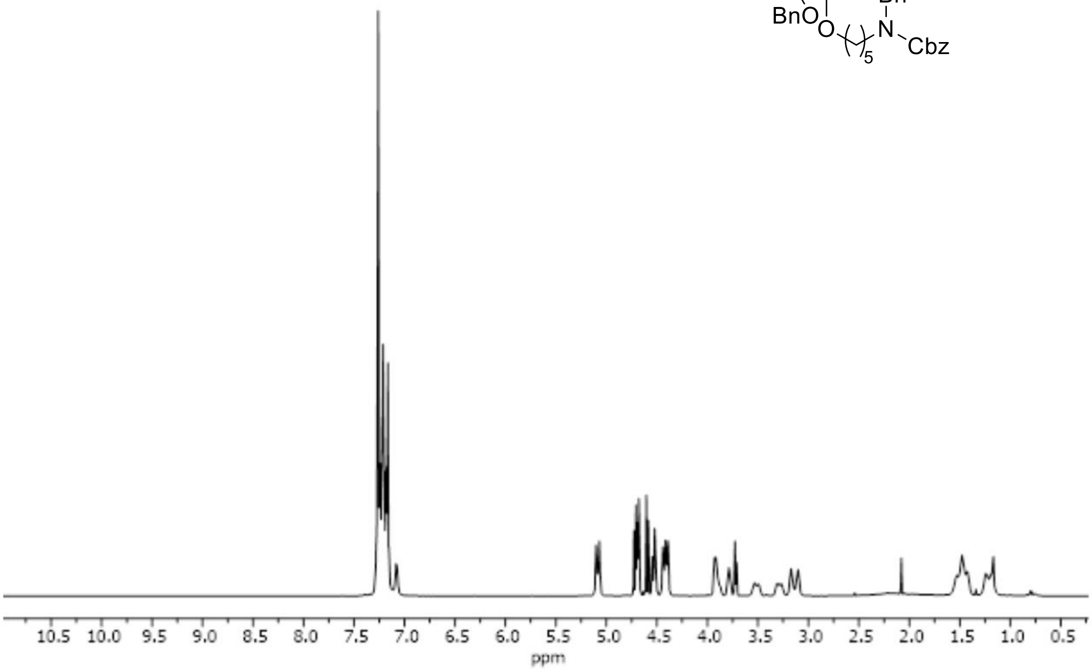

^{1}H NMR (600 MHz, $CDCl_3$) of **19**.

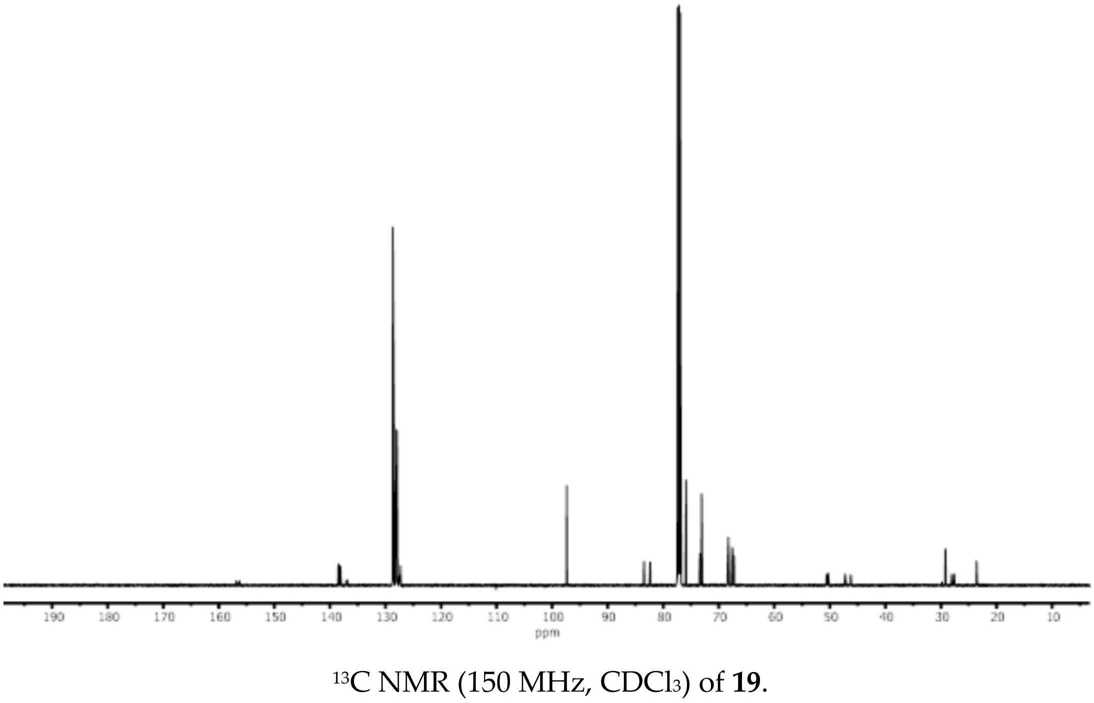

^{13}C NMR (150 MHz, $CDCl_3$) of **19**.

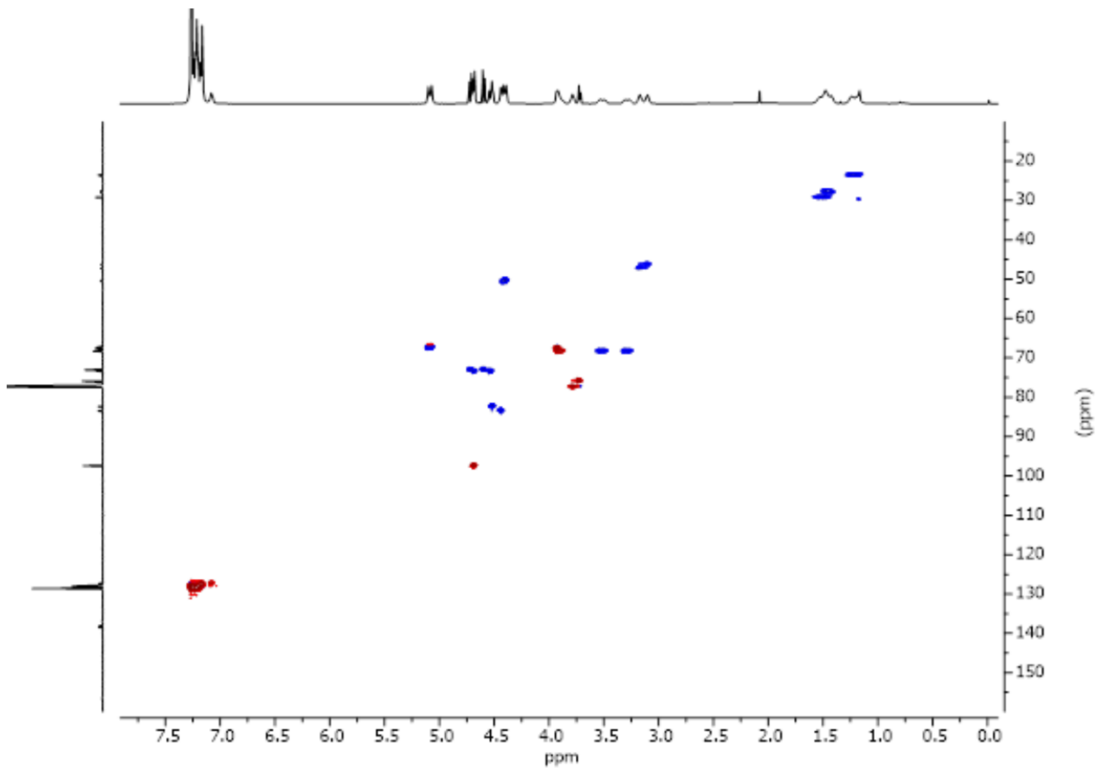

HSQC ($CDCl_3$) of **19**.

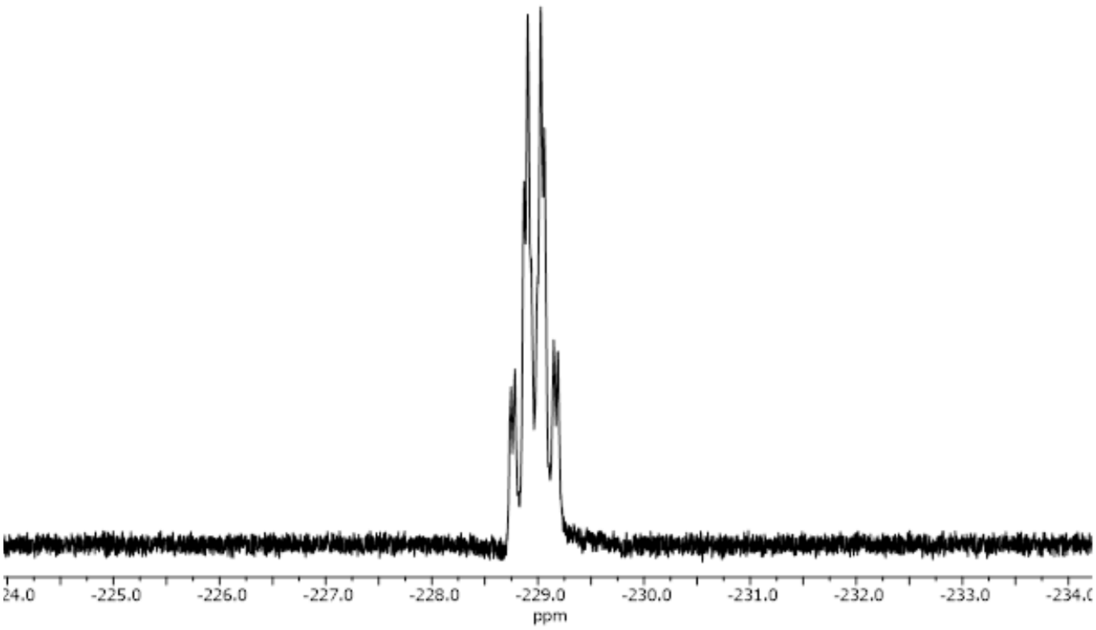

^{19}F NMR (377 MHz, $CDCl_3$) of **19**.

_N_-(Benzyl)-benzyloxycarbonyl-5-aminopentyl-(2,3-di-_O_-benzyl-4,6-_O_-benzyliden-α-D-glucopyranosyl)-(1→4)-2,3,6-tri-_O_-benzyl-α-D-galactopyranoside (29)

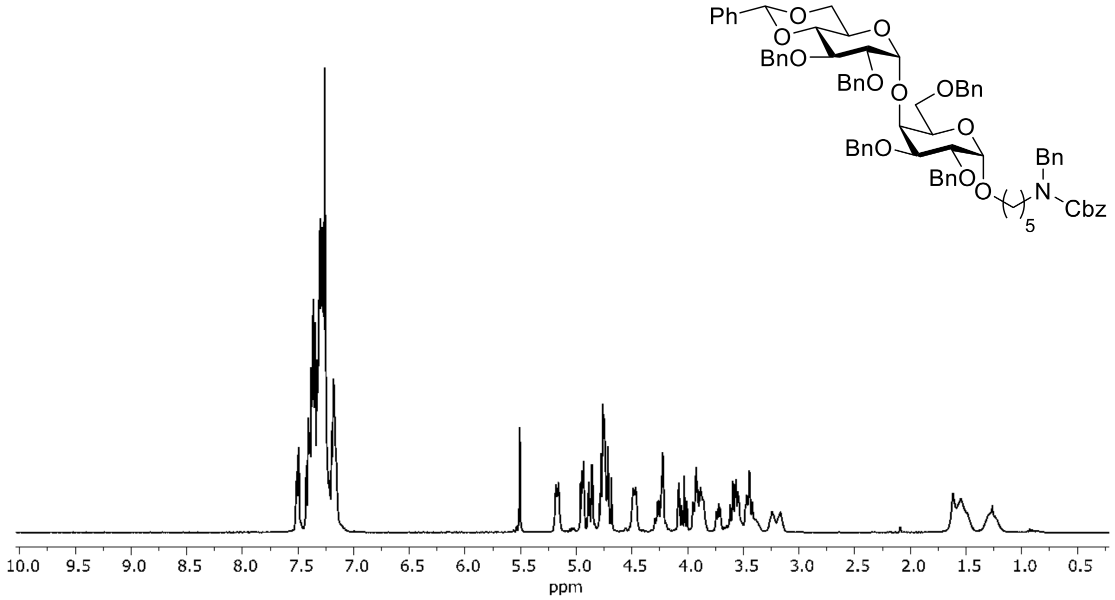

^{1}H NMR (400 MHz, $CDCl_3$) of **29**; Immediate measurement to avoid decomposition.

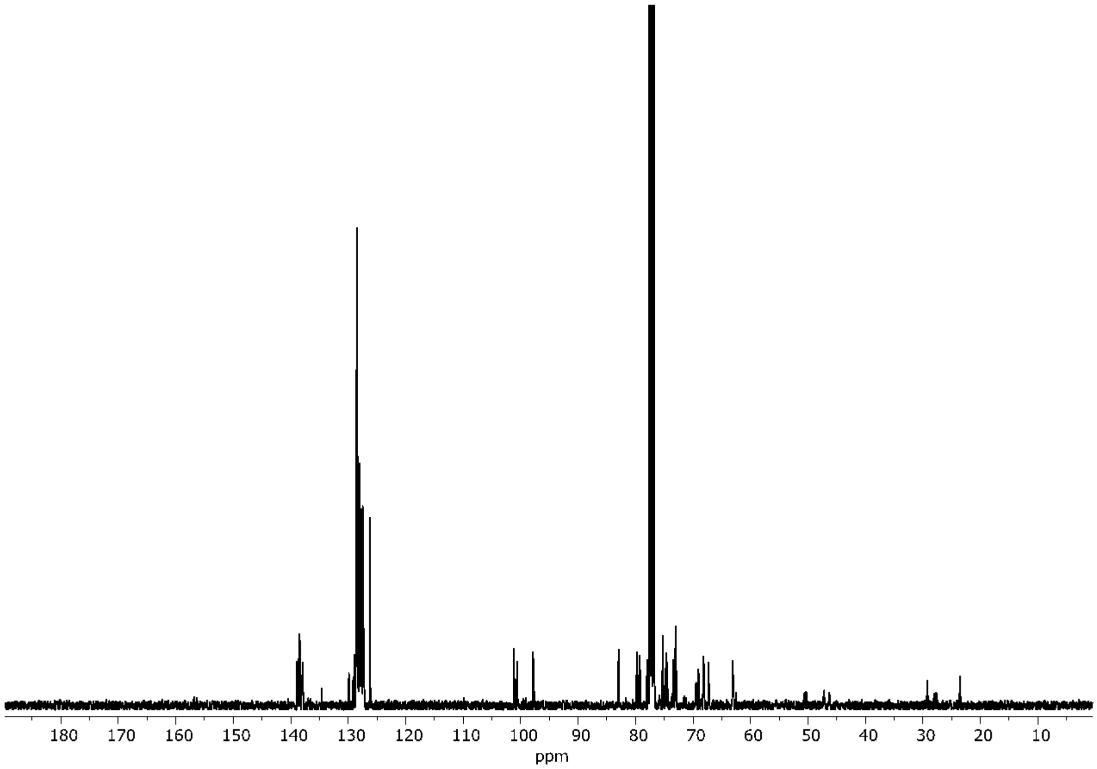

^{13}C NMR (100 MHz, $CDCl_3$) of **29**; Immediate measurement.

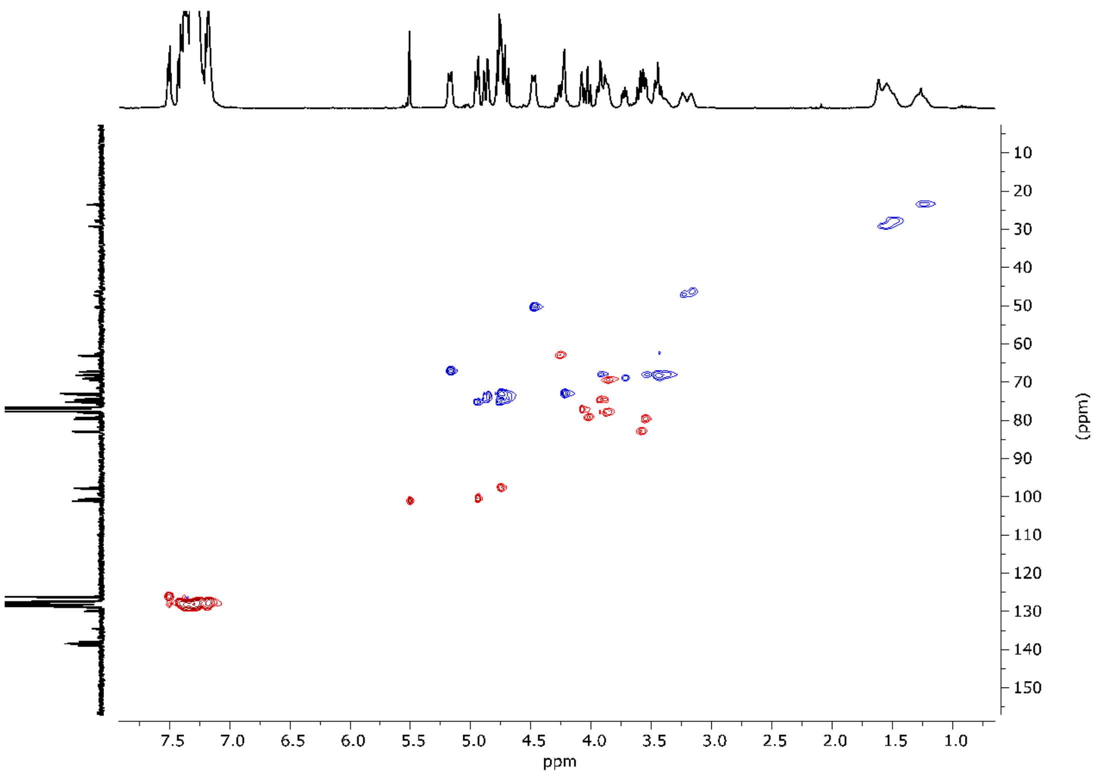

HSQC (CDCl3) of **29**; Immediate measurement.

N-(Benzyl)-benzyloxycarbonyl-5-aminopentyl-(2,3,6-tri-O-benzyl-α-D-glucopyranosyl)-(1→4)-2,3,6-tri-O-benzyl-α-D-galactopyranoside (10)

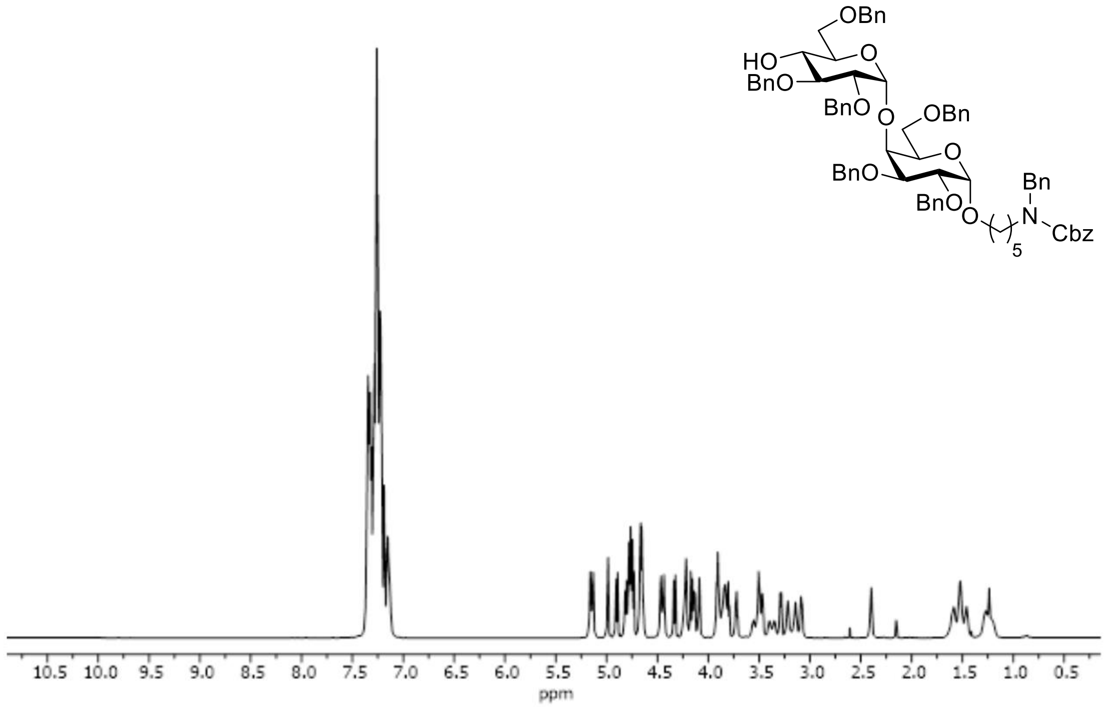

^{1}H NMR (600 MHz, CDCl3) of **10**.

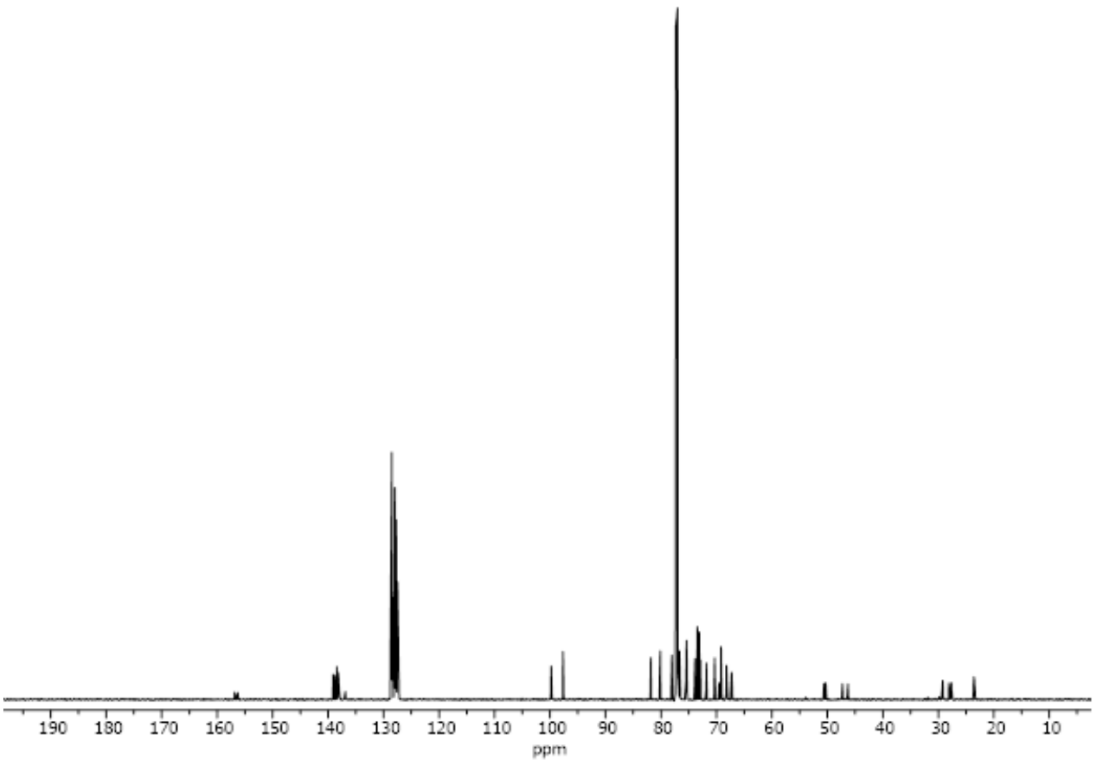

^{13}C NMR (150 MHz, $CDCl_3$) of **10**.

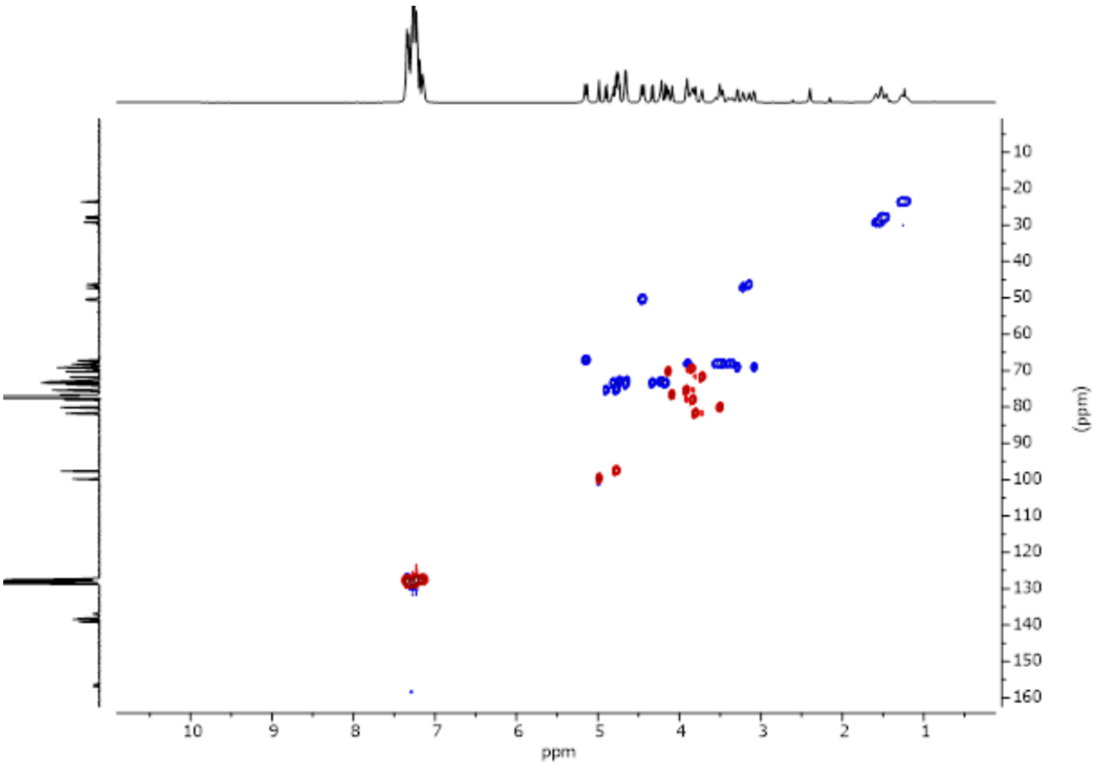

HSQC ($CDCl_3$) of **10**.

***N*-(Benzyl)-benzyloxycarbonyl-5-aminopentyl-(2,3-di-*O*-benzyl-6-*O*-*tert*-butyldimethylsilyl-α-D-glucopyranosyl)-(1→4)-2,3,6-tri-*O*-benzyl-α-D-galactopyranoside (11)**

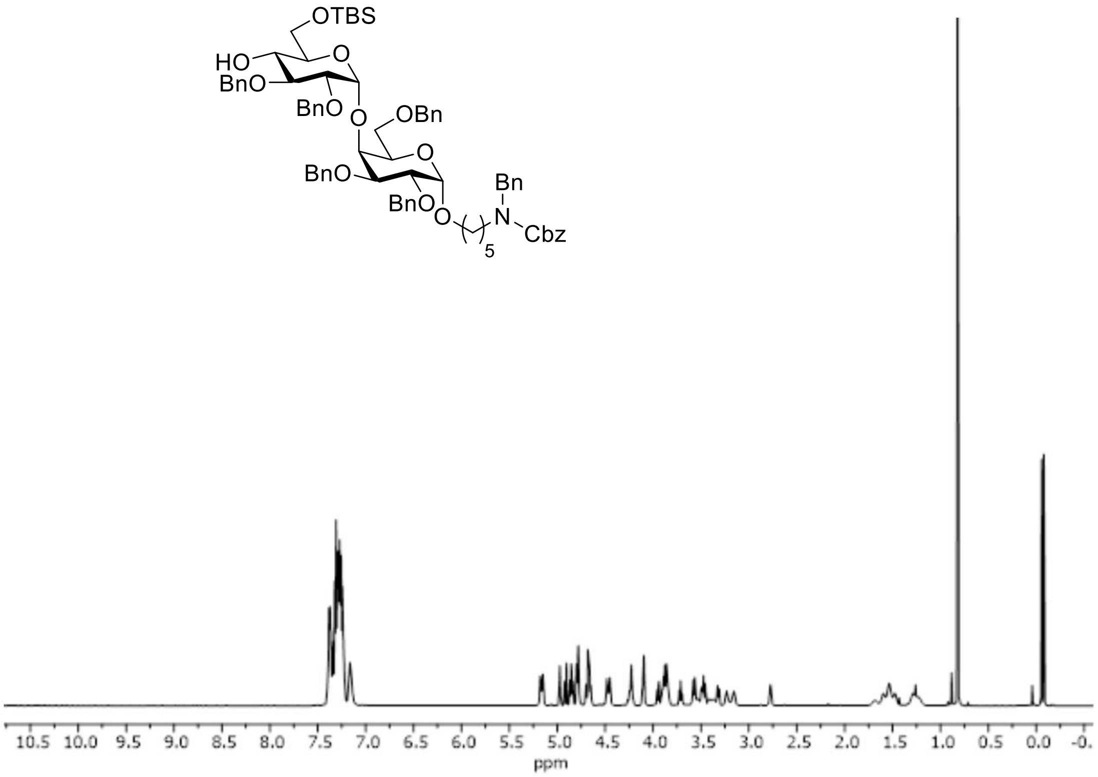

^{1}H NMR (600 MHz, $CDCl_3$) of **11**.

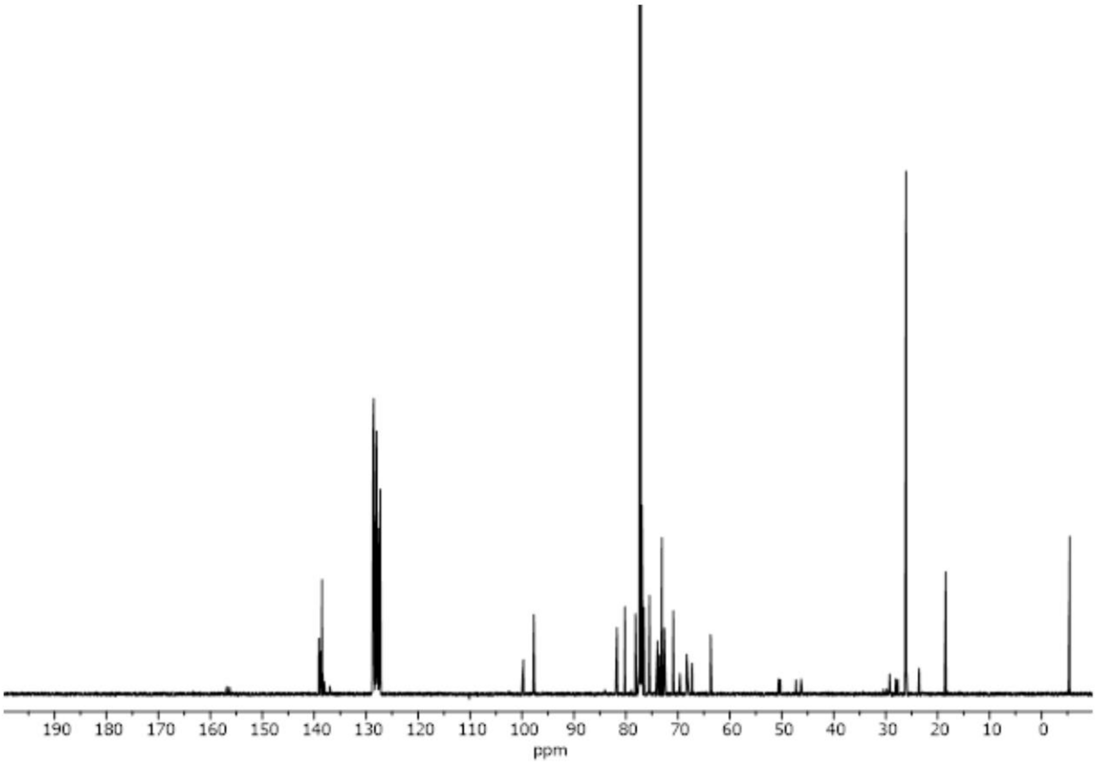

^{13}C NMR (150 MHz, $CDCl_3$) of **11**.

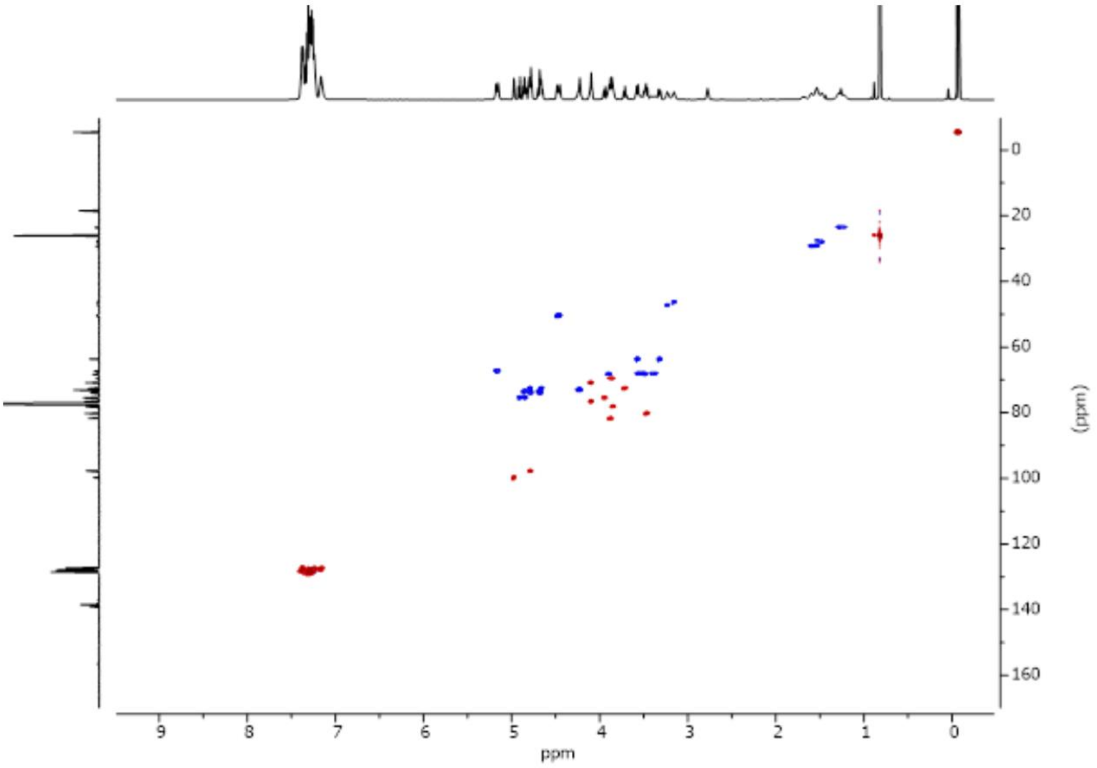

HSQC ($CDCl_3$) of **11**.

***N*-(Benzyl)-benzyloxycarbonyl-5-aminopentyl-(2,3,6-tri-*O*-benzyl-α-D-glucopyranosyl)-(1→4)-2,3-di-*O*-benzyl-6-deoxy-6-fluoro-α-D-galactopyranoside (12)**

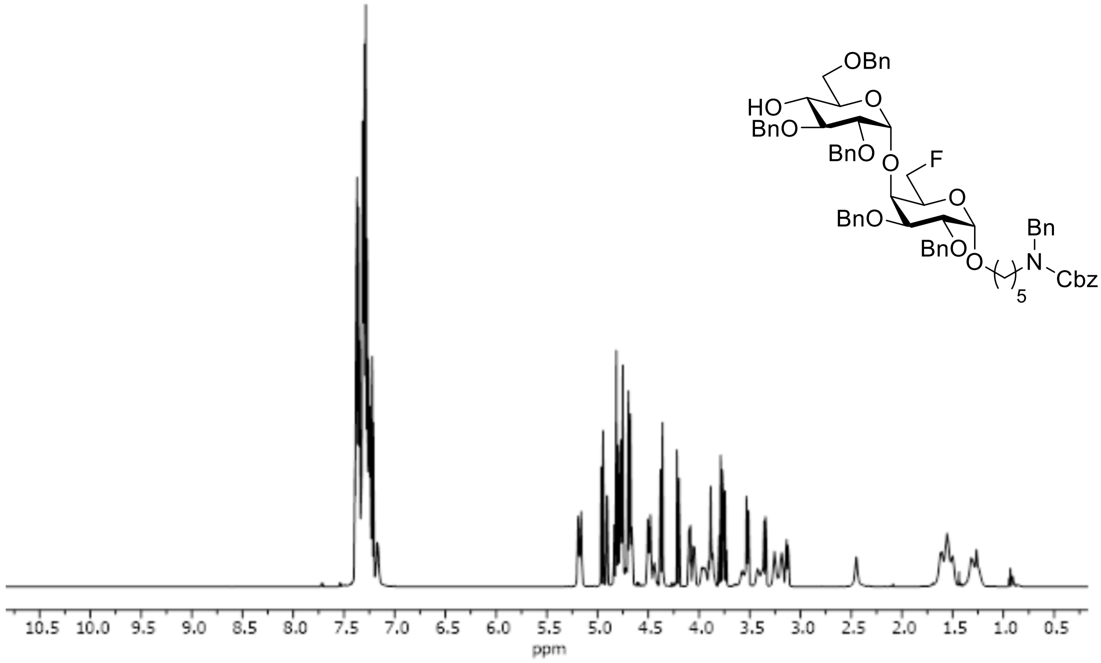

^1H NMR (600 MHz, $CDCl_3$) of **12**.

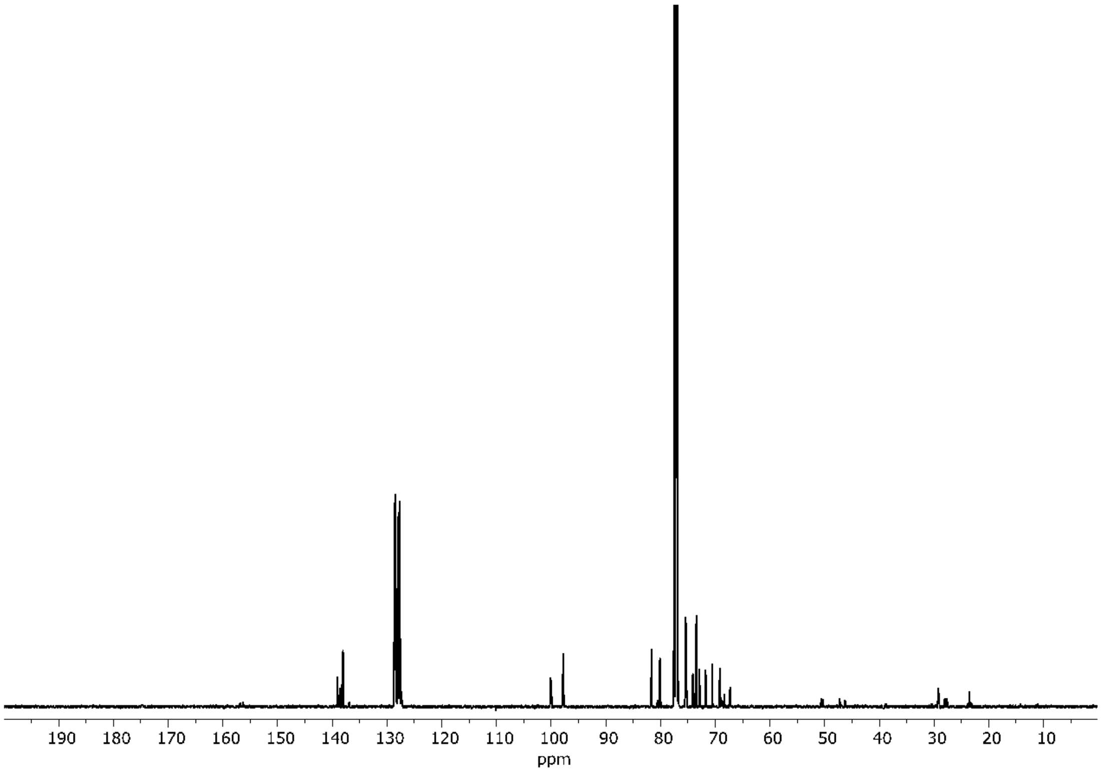

^{13}C NMR (150 MHz, $CDCl_3$) of **12**.

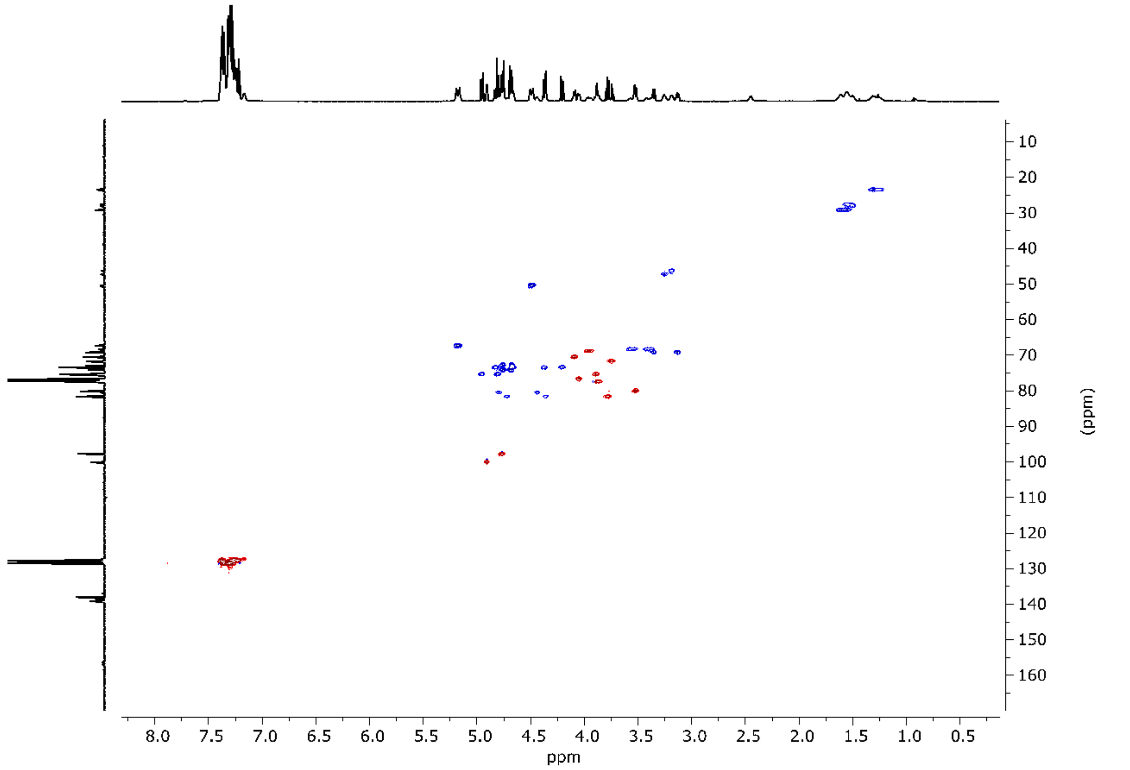

HSQC ($CDCl_3$) of **12**.

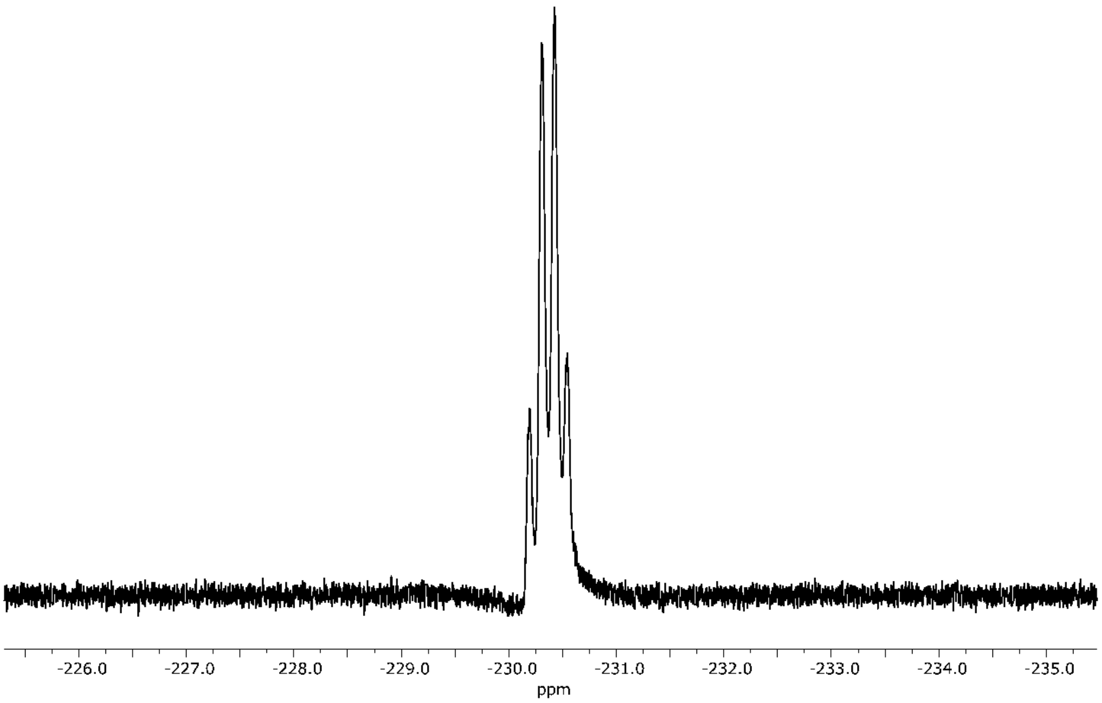

^{19}F NMR (377 MHz, $CDCl_3$) of **12**.

Allyl-(2,3,4-tri-*O*-benzoyl-6-deoxy-6-fluoro-β-D-glucopyranosyl)-(1→4)-2,3-di-*O*-benzoyl-6-*O*-*tert*-butyldimethylsilyl-β-D-glucopyranoside (31)

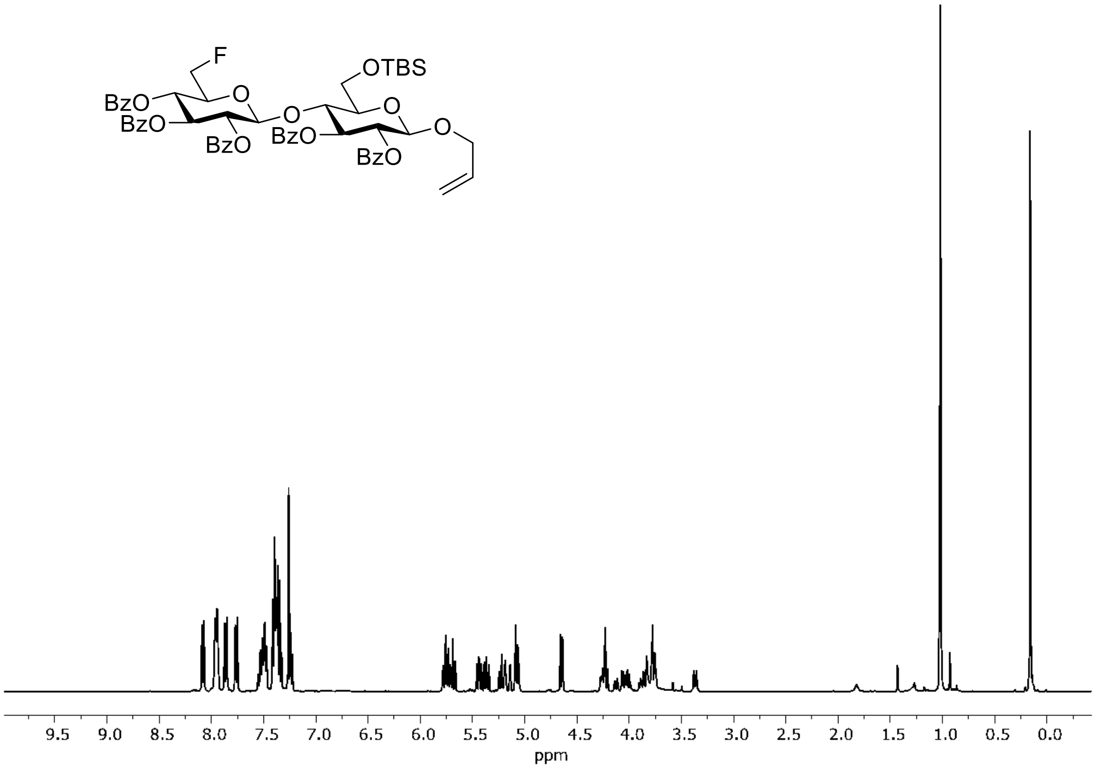

^{1}H NMR (400 MHz, $CDCl_3$) of **31**.

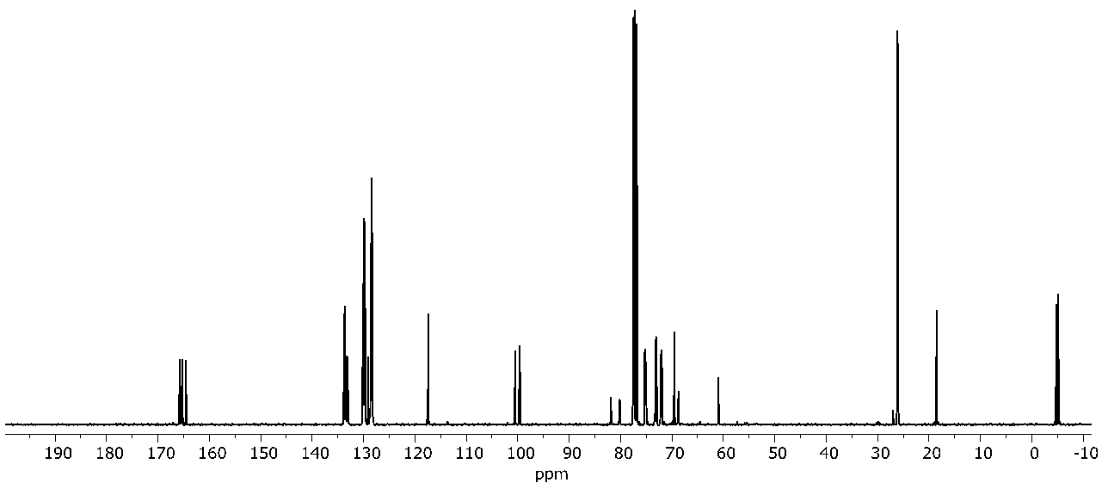

^{13}C NMR (100 MHz, $CDCl_3$) of **31**.

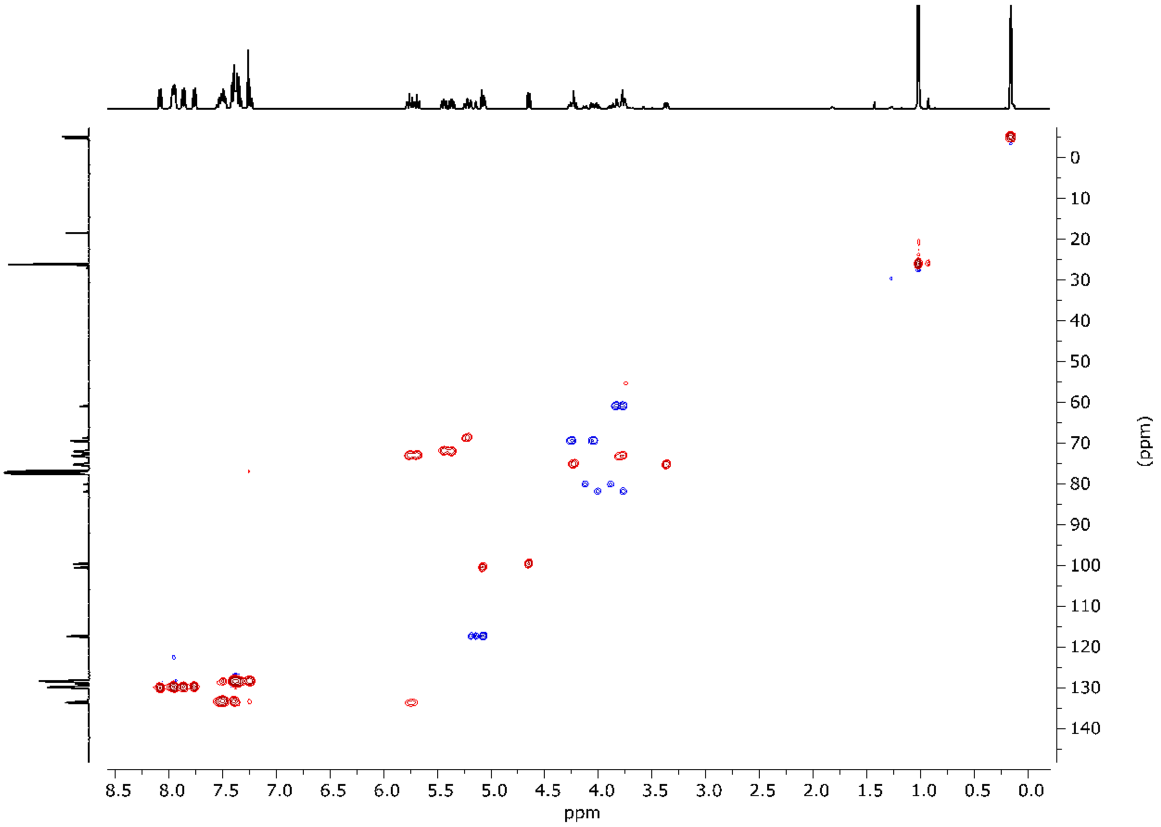

HSQC ($CDCl_3$) of **31**.

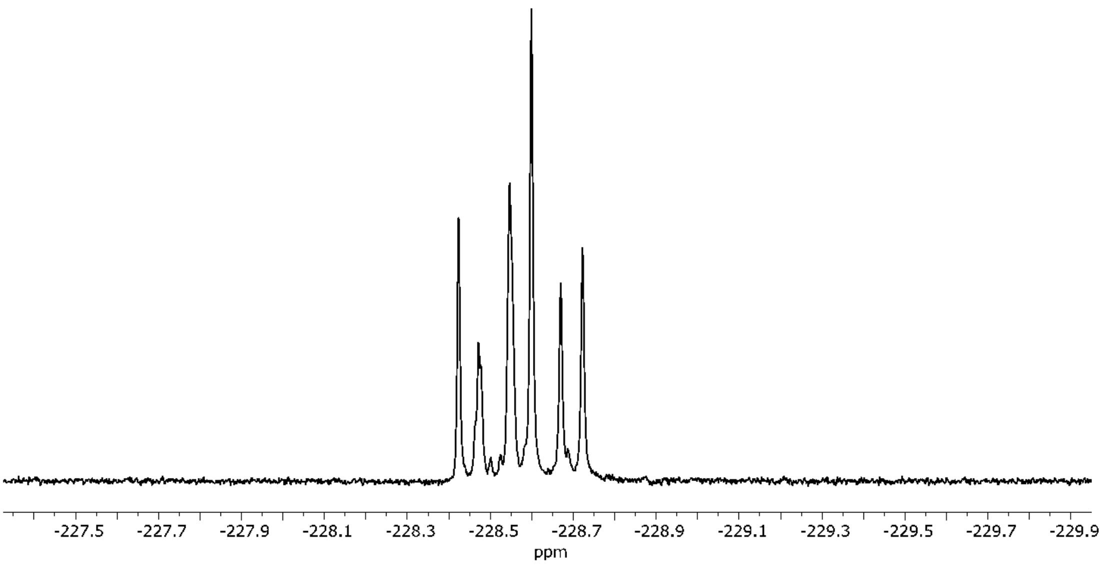

^{19}F NMR (377 MHz, $CDCl_3$) of **31**.

Allyl-(2,3,4-tri-*O*-benzoyl 6-deoxy-6-fluoro-β-D-glucopyranosyl)-(1→4)-2,3,6-tri-*O*-benzoyl-β-D-glucopyranoside (32)

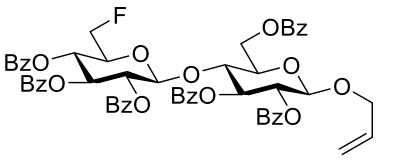

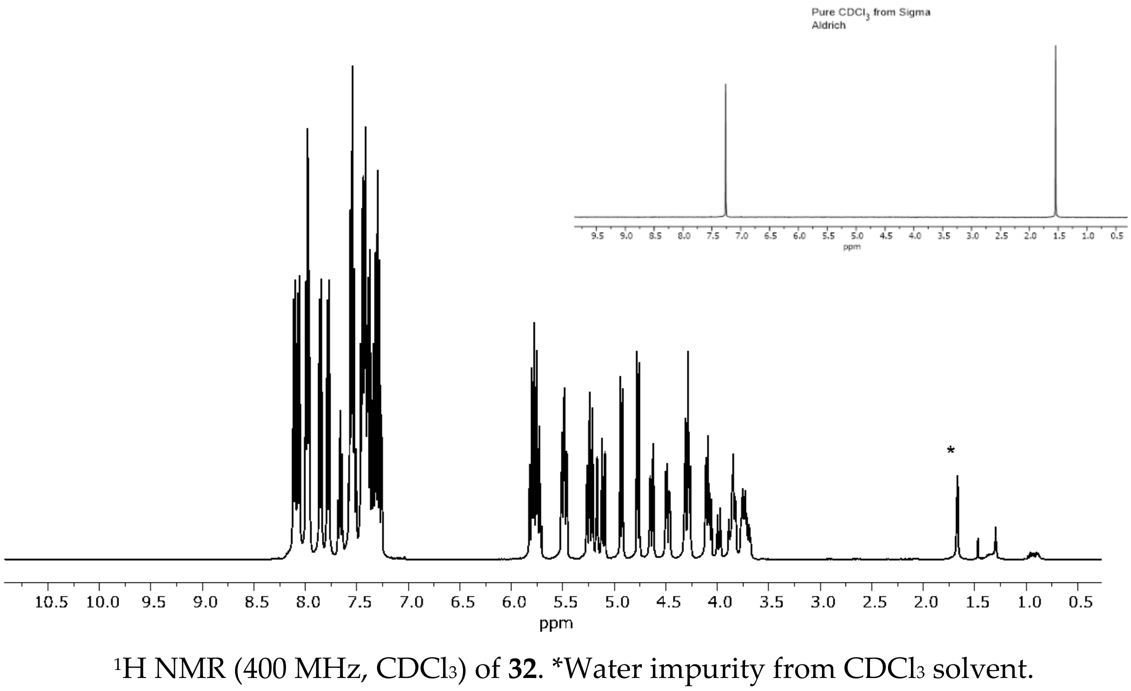

^{1}H NMR (400 MHz, $CDCl_3$) of **32**. *Water impurity from $CDCl_3$ solvent.

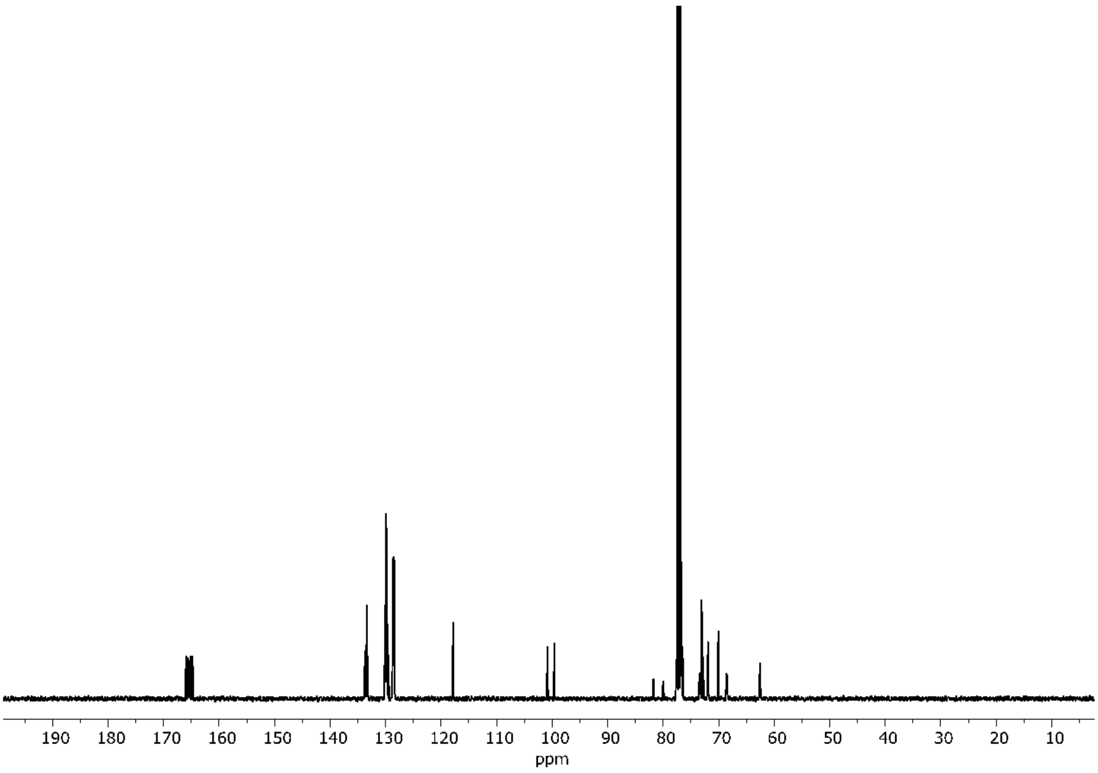

^{13}C NMR (100 MHz, $CDCl_3$) of **32**.

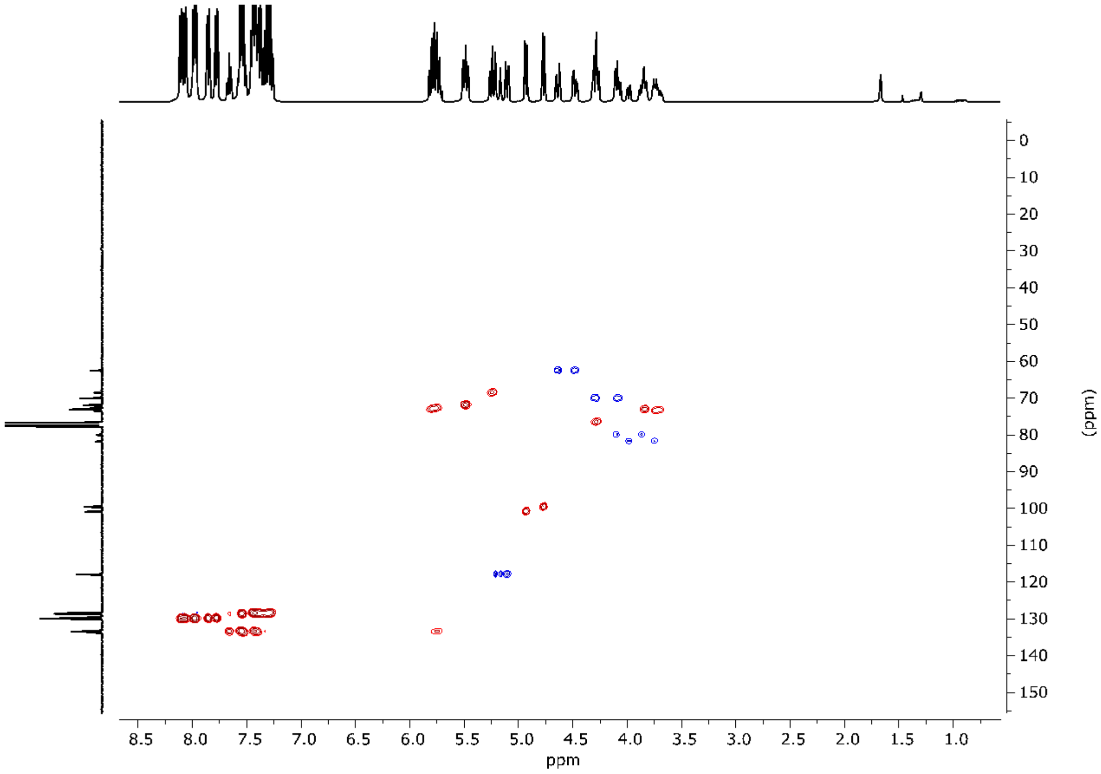

HSQC ($CDCl_3$) of **32**.

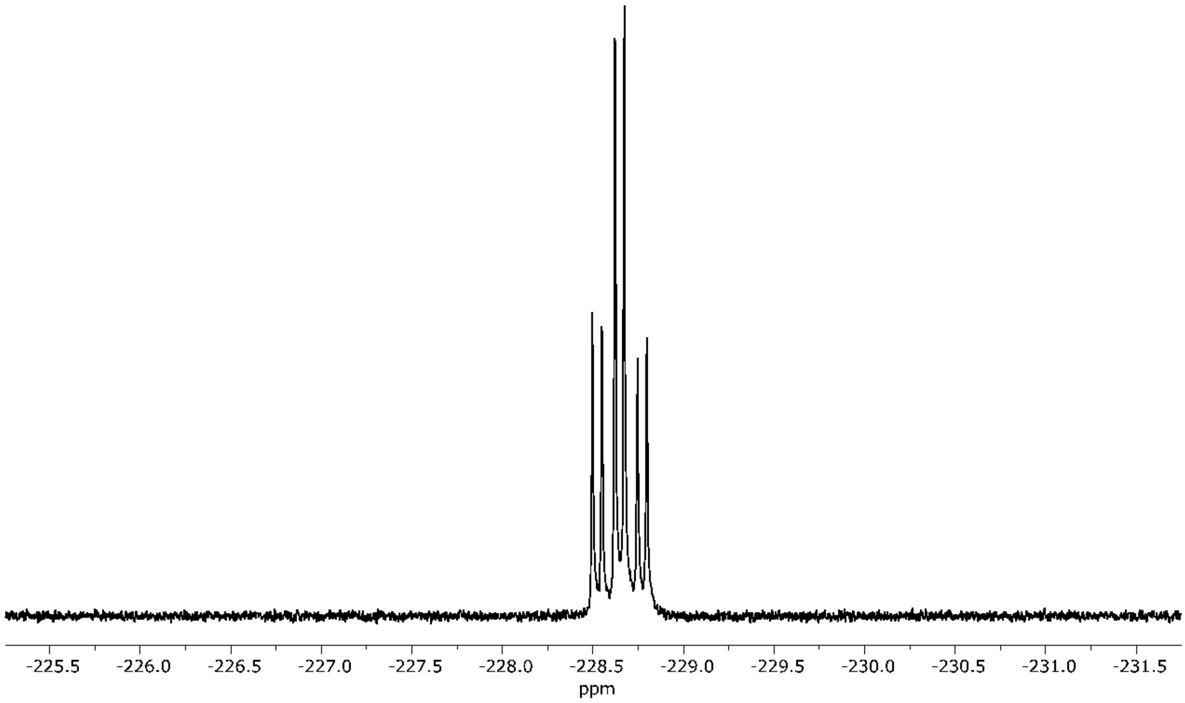

^{19}F NMR (377 MHz, $CDCl_3$) of **32**.

Allyl-(2,3,4,6-tetra-*O*-benzoyl-β-D-glucopyranosyl)-(1→4)-2,3-di-*O*-benzoyl-6-*O*-tert-butyldimethylsilyl-β-D-glucopyranoside (34)

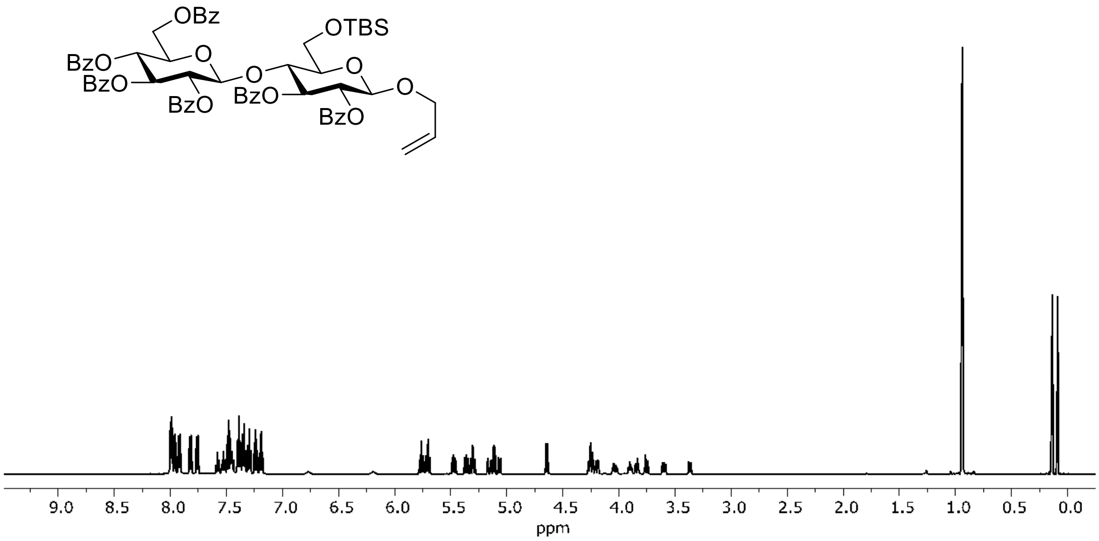

^{1}H NMR (600 MHz, $CDCl_3$) of **34**.

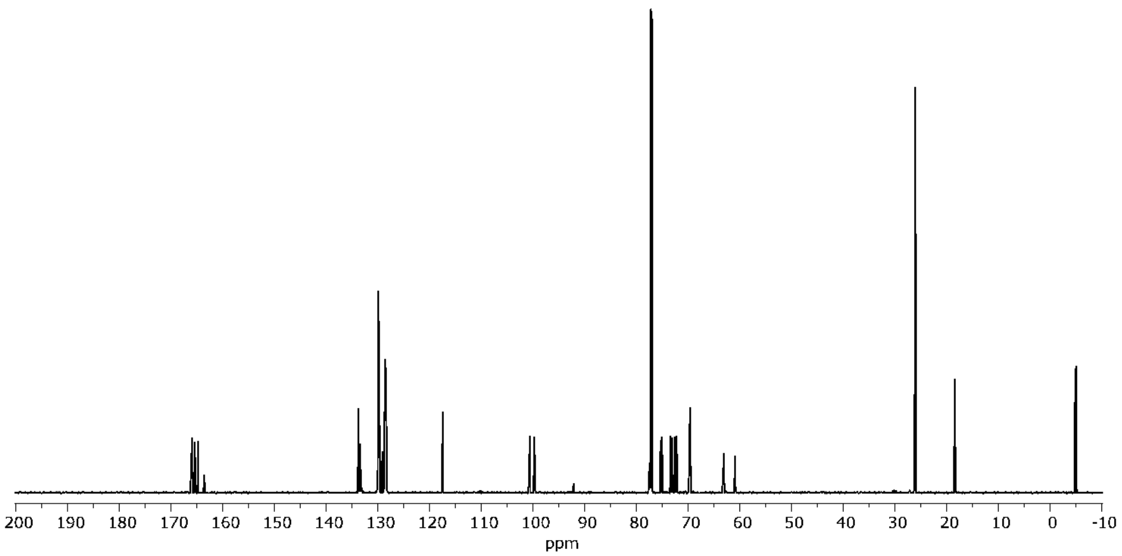

^{13}C NMR (150 MHz, $CDCl_3$) of **34**.

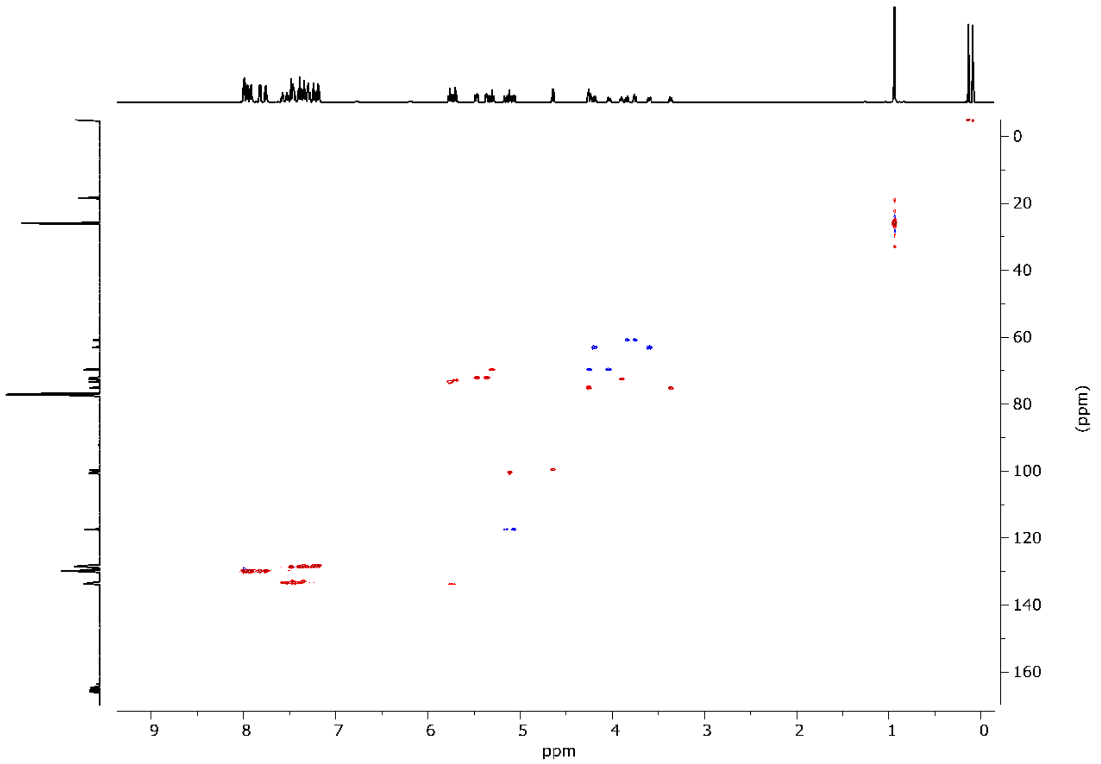

HSQC ($CDCl_3$) of **34**.

Allyl-(2,3,4,6-tetra-*O*-benzoyl-β-D-glucopyranosyl)-(1→4)-2,3-di-*O*-benzoyl-6-deoxy-6-fluoro-β-D-gluco pyranoside (36)

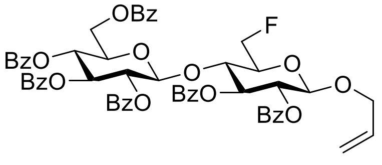

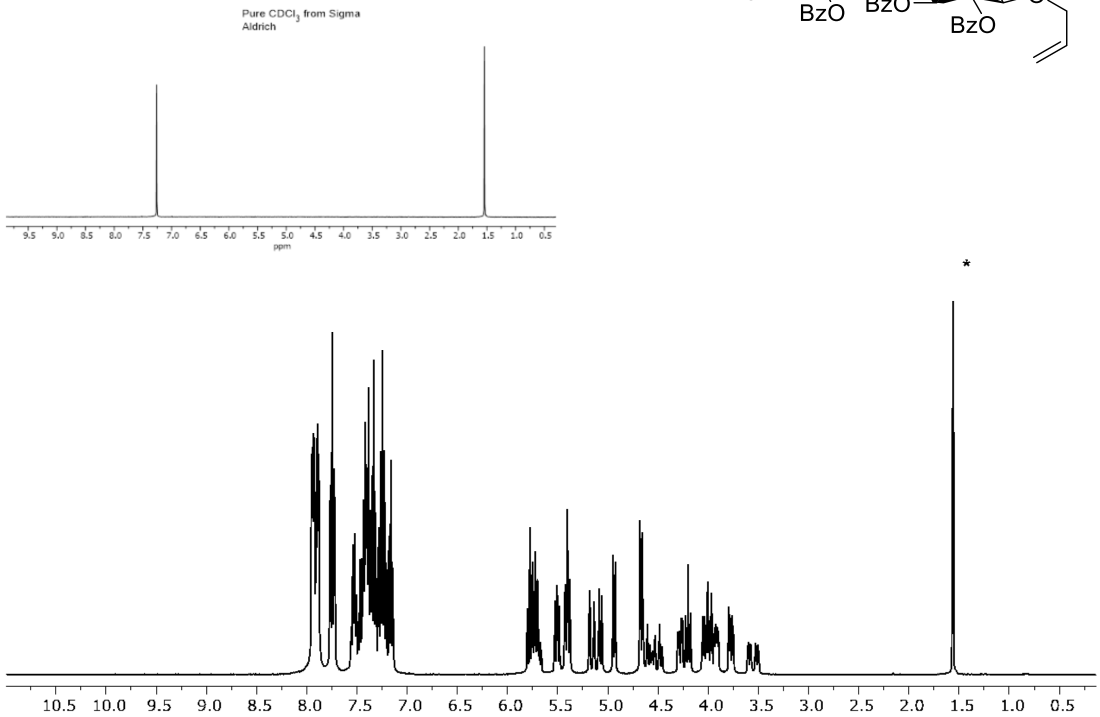

^{1}H NMR (400 MHz, $CDCl_3$) of **36**. *Water impurity from $CDCl_3$ solvent.

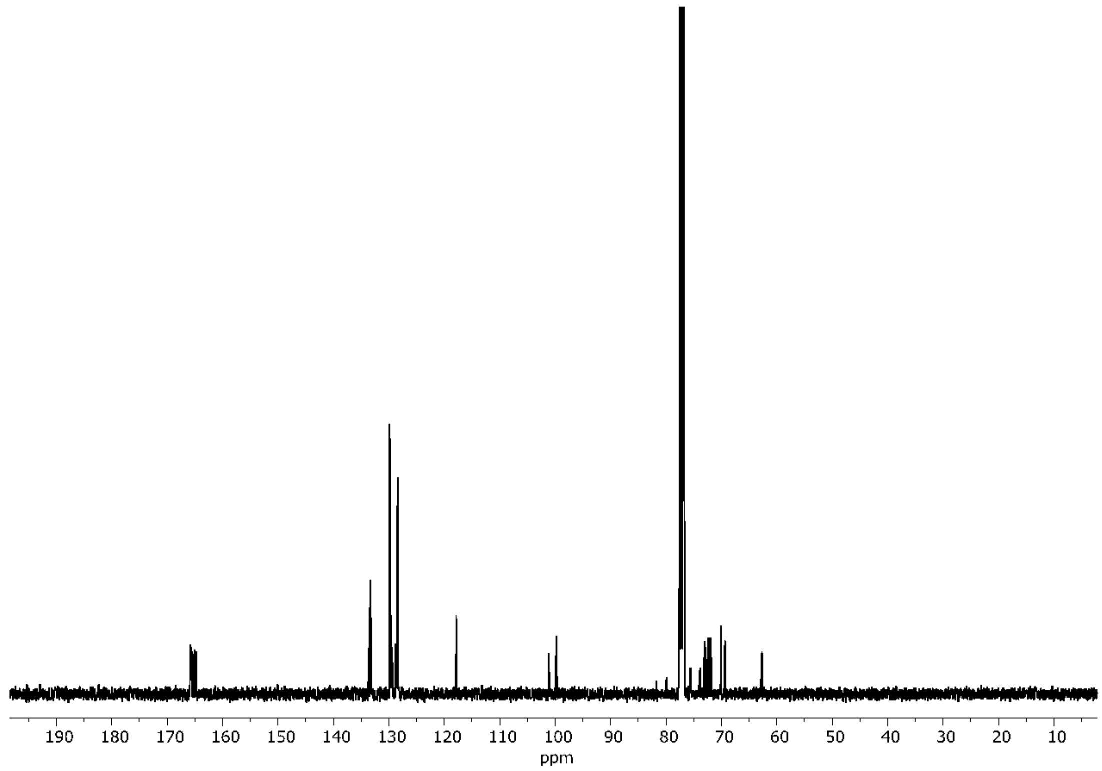

^{13}C NMR (100 MHz, $CDCl_3$) of **36**.

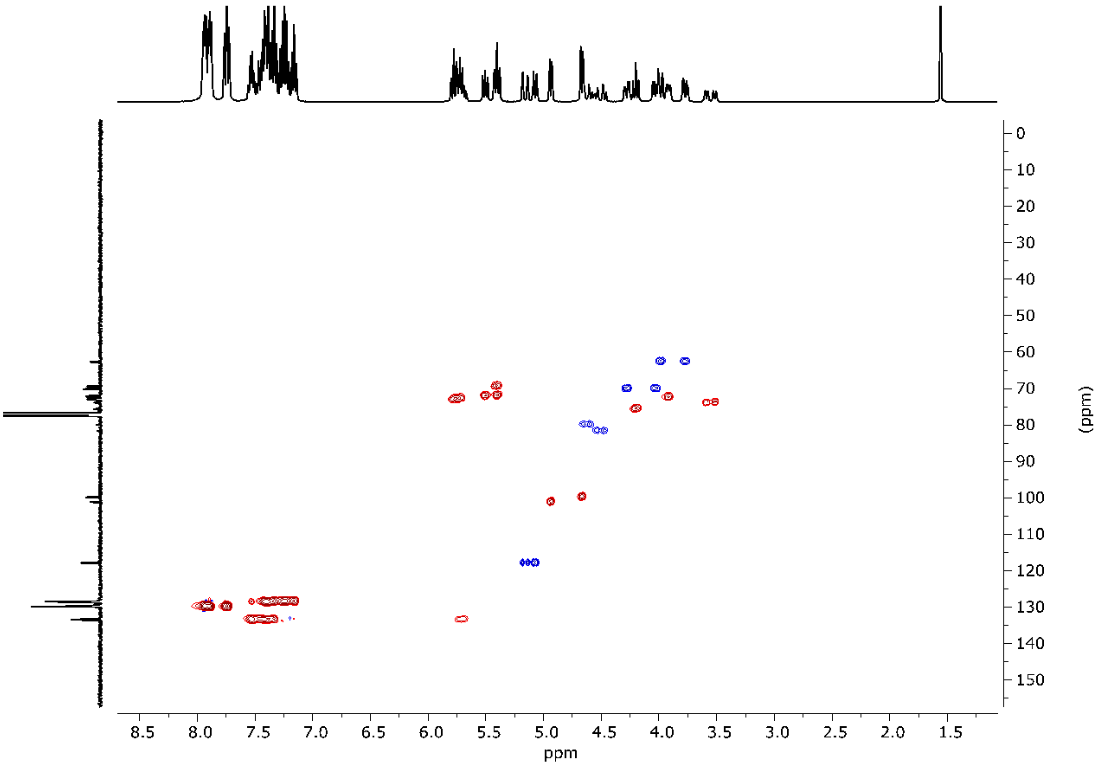

HSQC NMR ($CDCl_3$) of **36**.

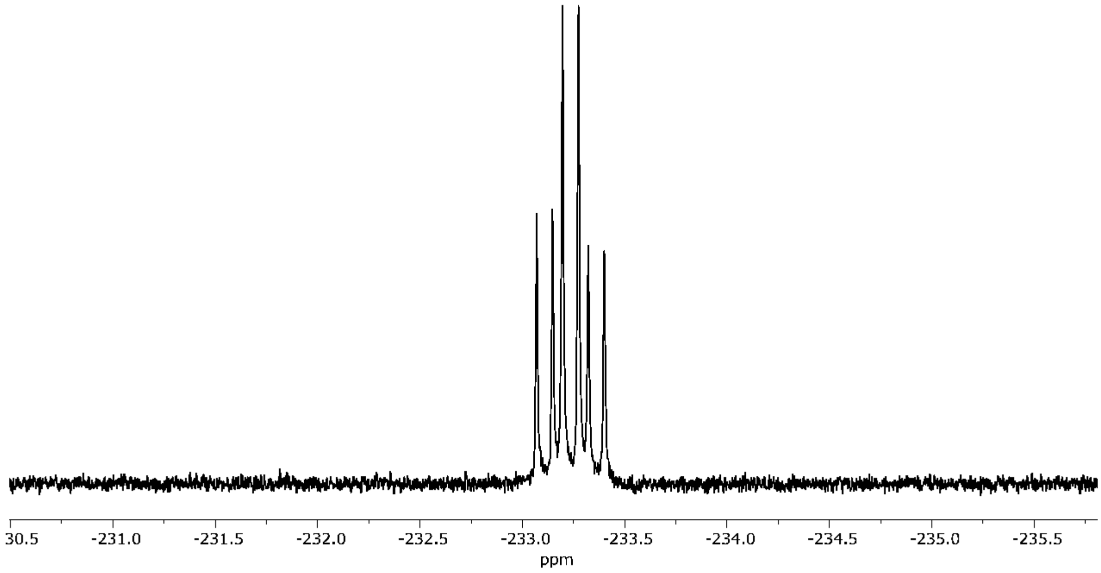

^{19}F NMR (377 MHz, $CDCl_3$) of **36**.

N-(Benzyl)-benzyloxycarbonyl-5-aminopentyl-(2,3,4-tri-O-benzoyl-6-deoxy-6-fluoro-β-D-glucopyranosyl)-(1→4)-(2,3,6-tri-O-benzyl-α-D-glucopyranosyl)-(1→4)-2,3,6-tri-O-benzyl-α-D-galactopyranoside (38)

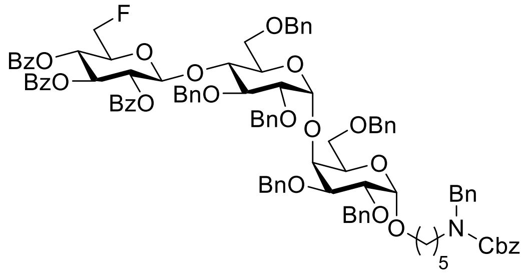

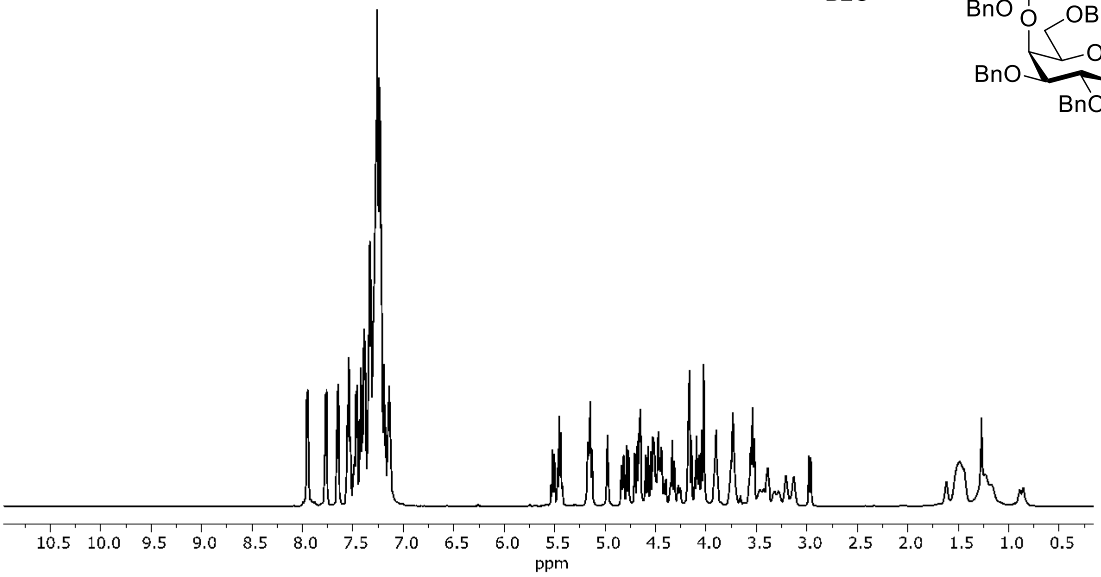

^{1}H NMR (600 MHz, $CDCl_3$) of **38**.

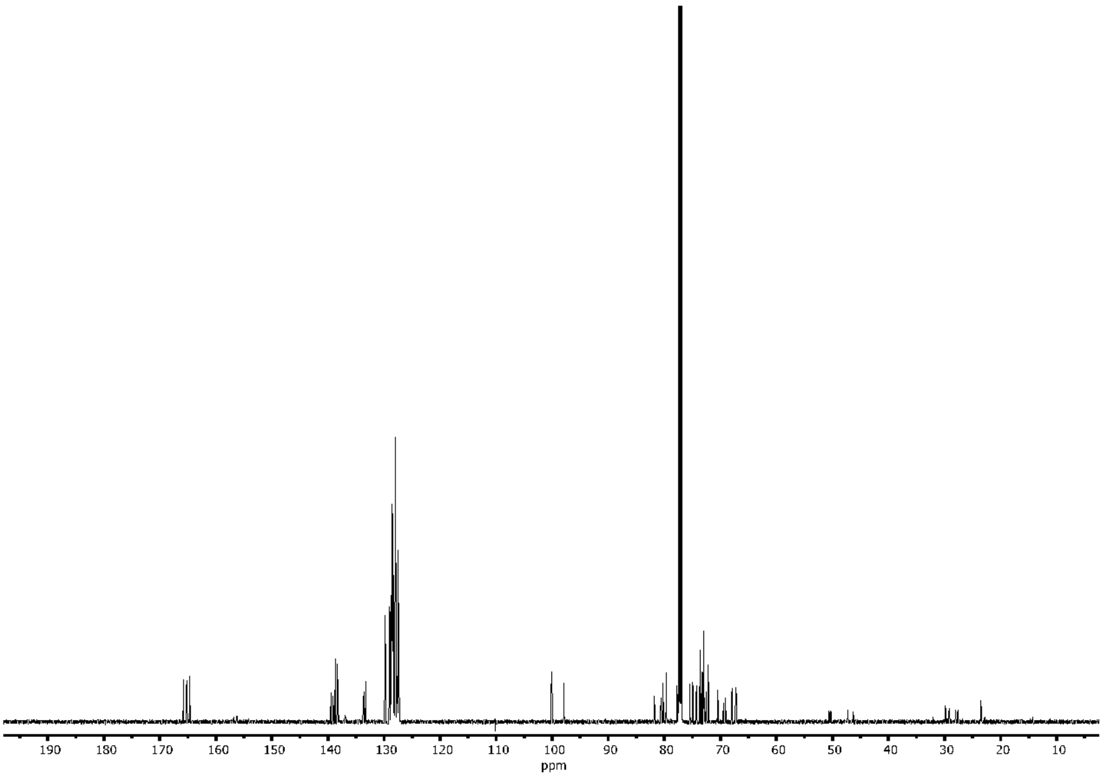

^{13}C NMR (100 MHz, $CDCl_3$) of **38**.

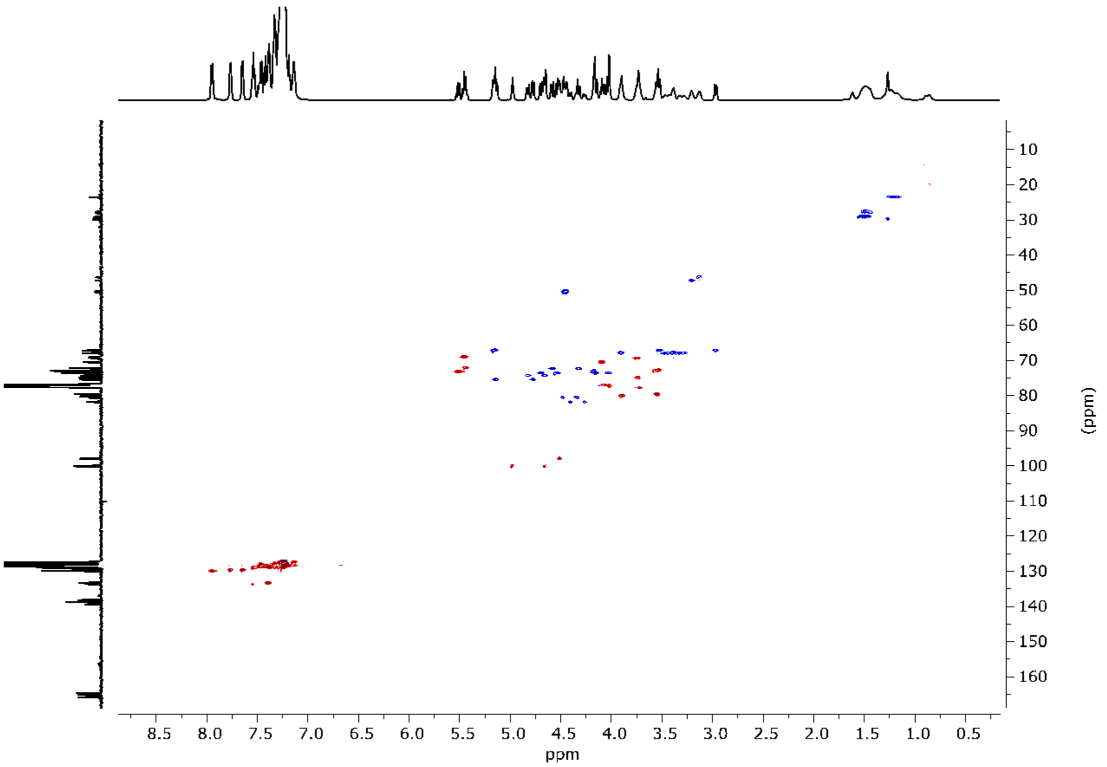

HSQC ($CDCl_3$) of **38**.

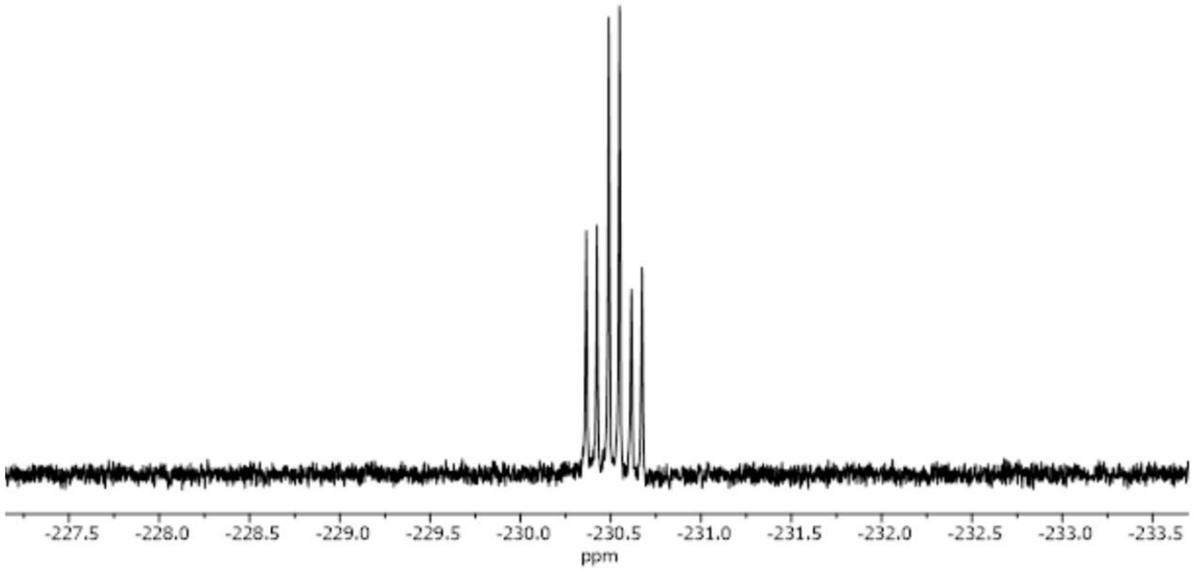

^{19}F NMR (377 MHz, $CDCl_3$) of **38**.

5-Aminopentyl-(6-deoxy-6-fluoro-β-D-glucopyranosyl)-(1→4)-(α-D-glucopyranosyl)-(1→4)-α-D-galactopyranoside (3)

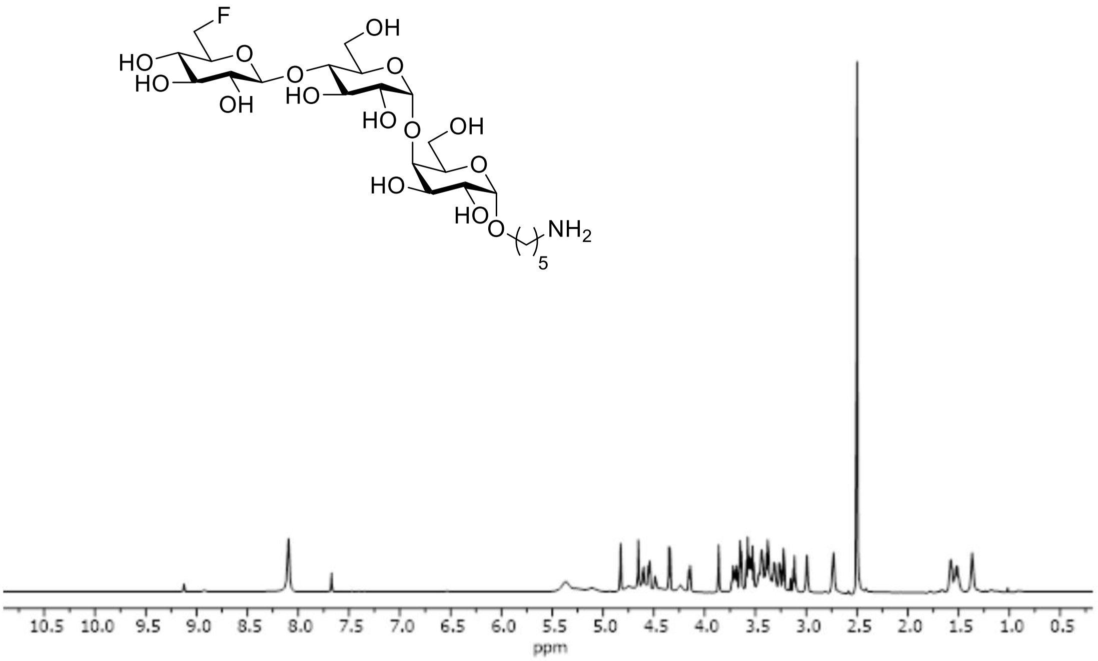

^{1}H NMR (800 MHz, DMSO-d$_6$) of **3**.

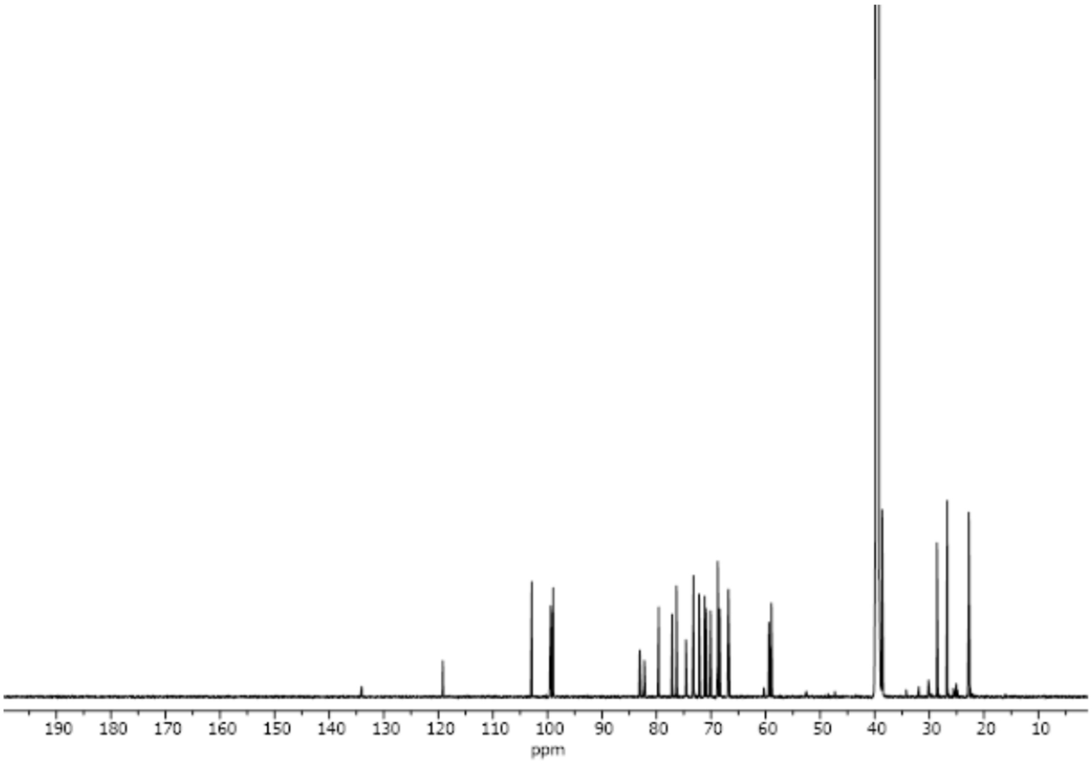

^{13}C NMR (200 MHz, DMSO-d$_6$) of **3**.

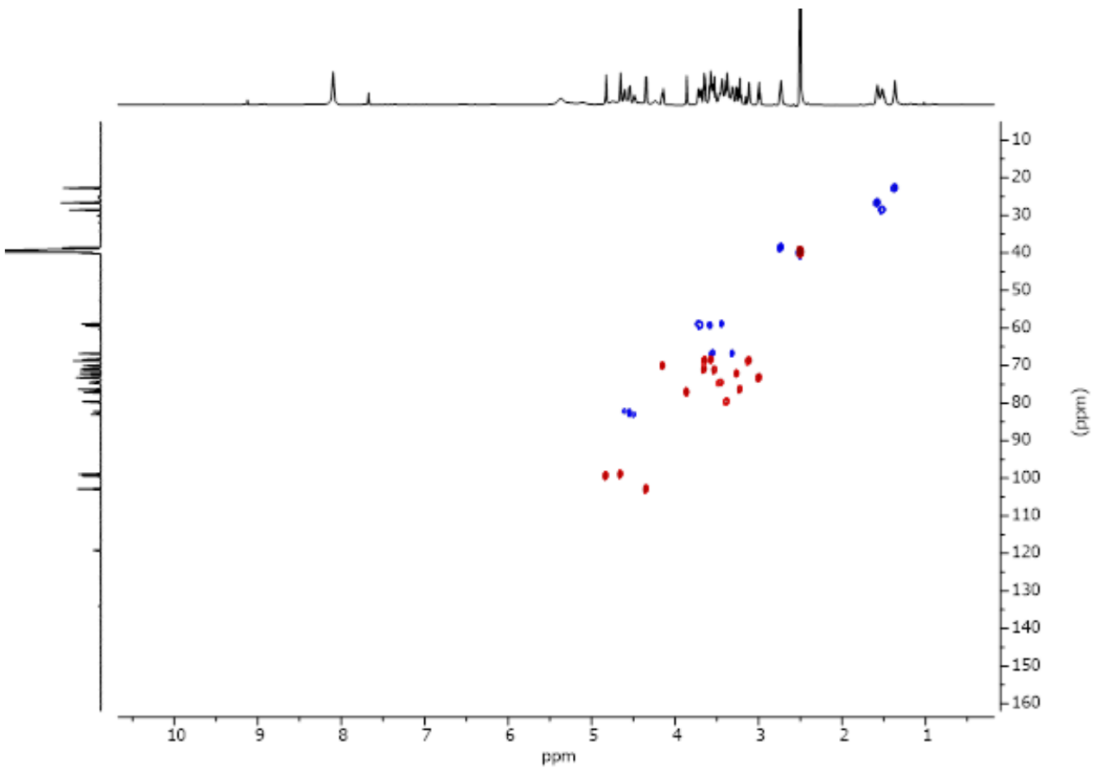

HSQC (DMSO-d_6) of **3**.

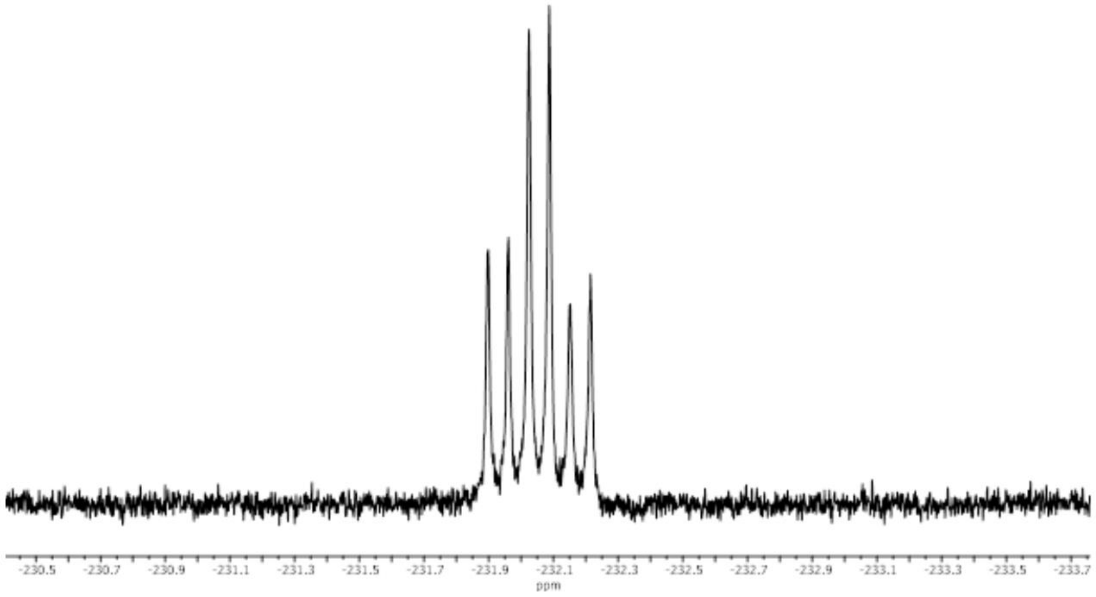

^{19}F NMR (377 MHz, DMSO-d_6) of **3**.

N-(Benzyl)-benzyloxycarbonyl-5-aminopentyl-(2,3,4,6-tetra-O-benzoyl-β-D-gluco-pyranosyl)-(1→4)-(2,3,6-tri-O-benzyl-α-D-glucopyranosyl)-(1→4)-2,3-di-O-benzyl-6-deoxy-6-fluoro-α-D-galactopyranoside (39)

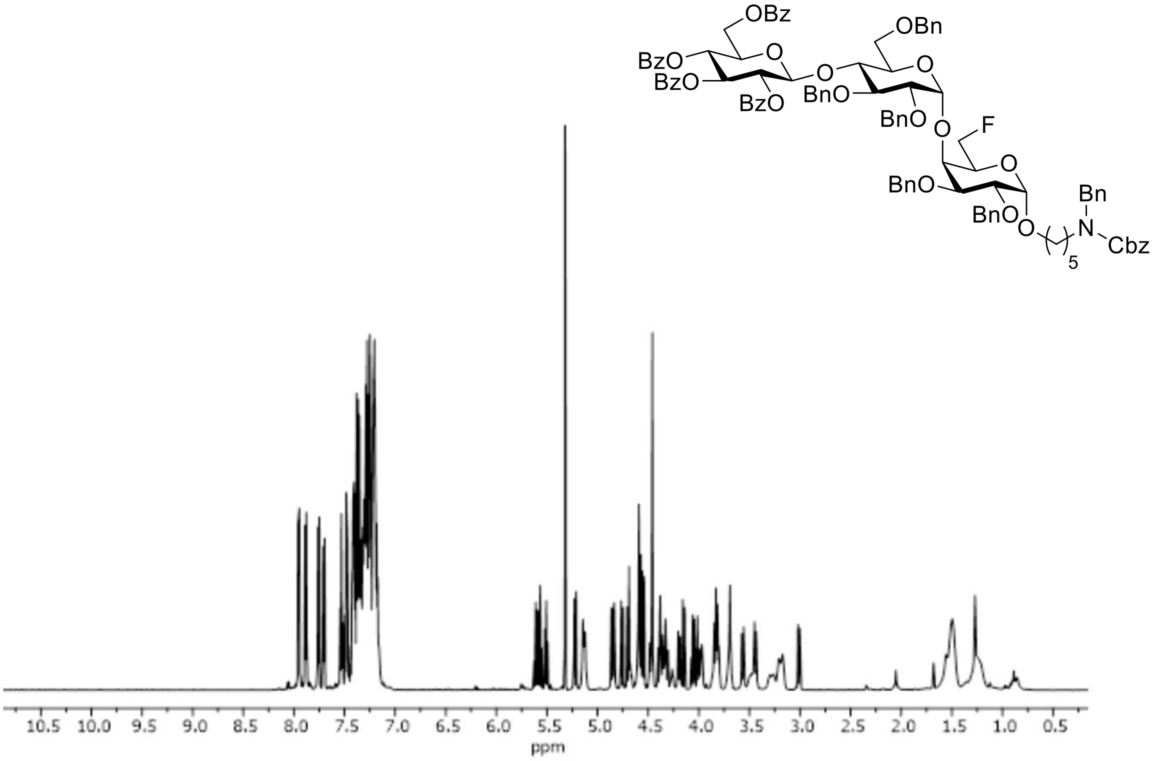

^{1}H NMR (800 MHz, CD_2Cl_2) of **39**.

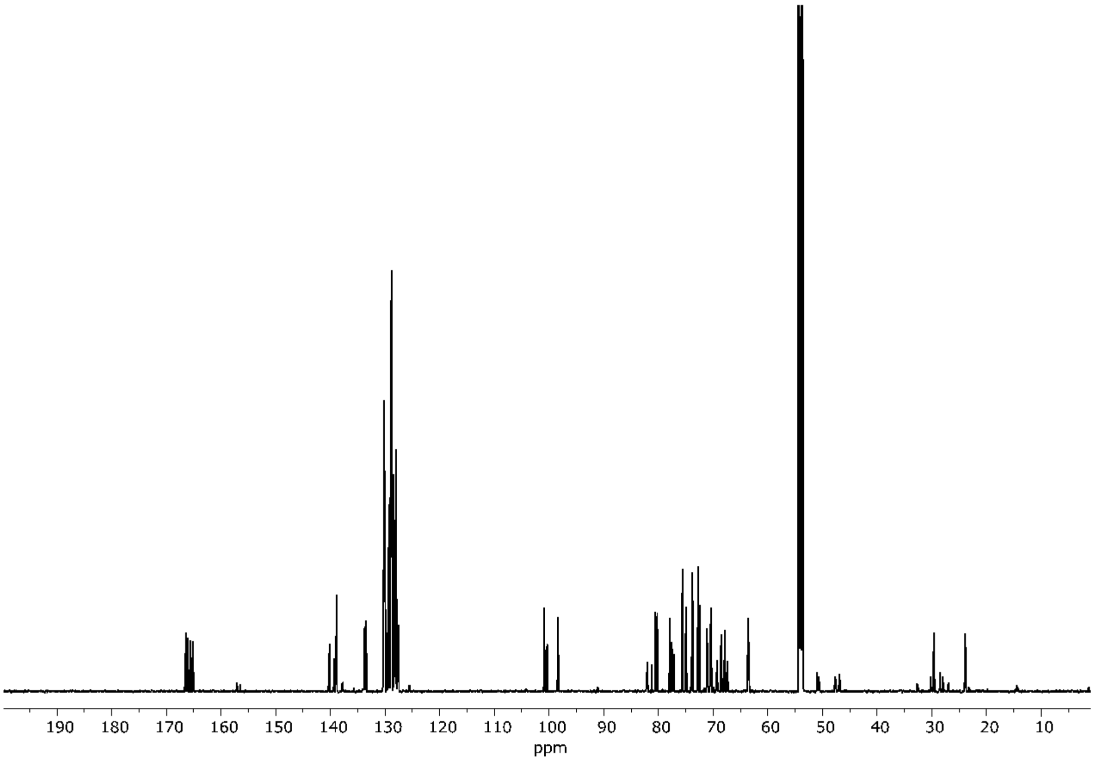

^{13}C NMR (200 MHz, CD_2Cl_2) of **39**.

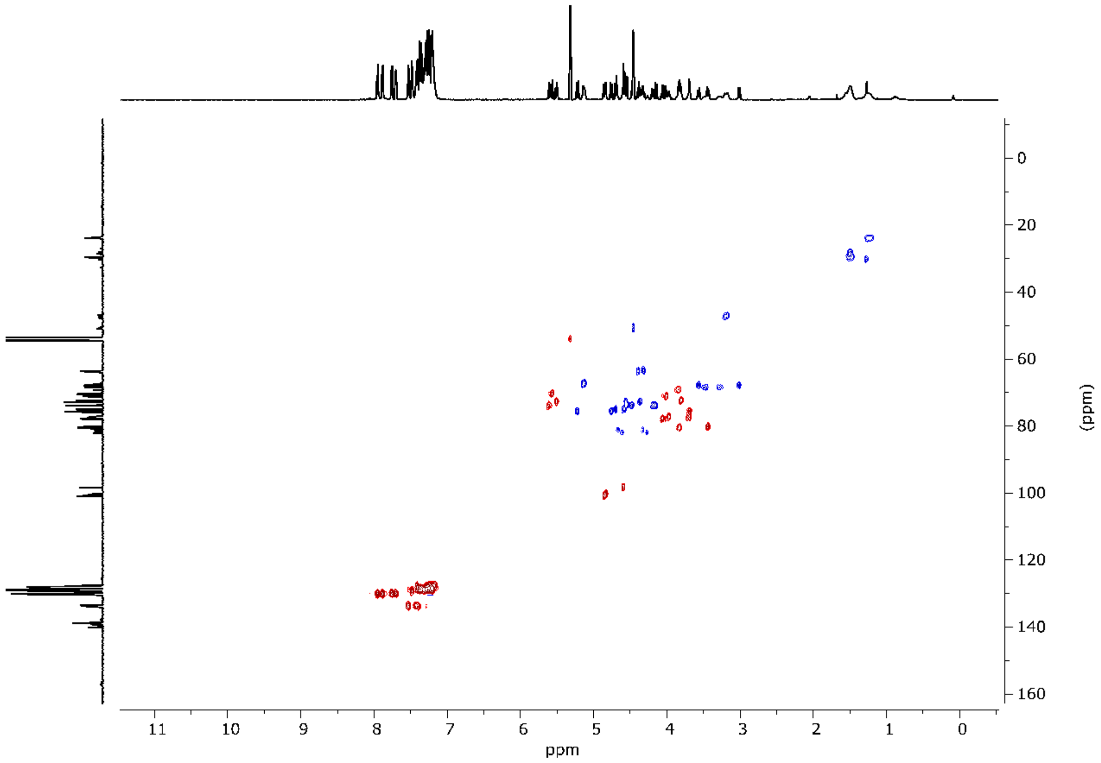

HSQC (CD_2Cl_2) of **39**.

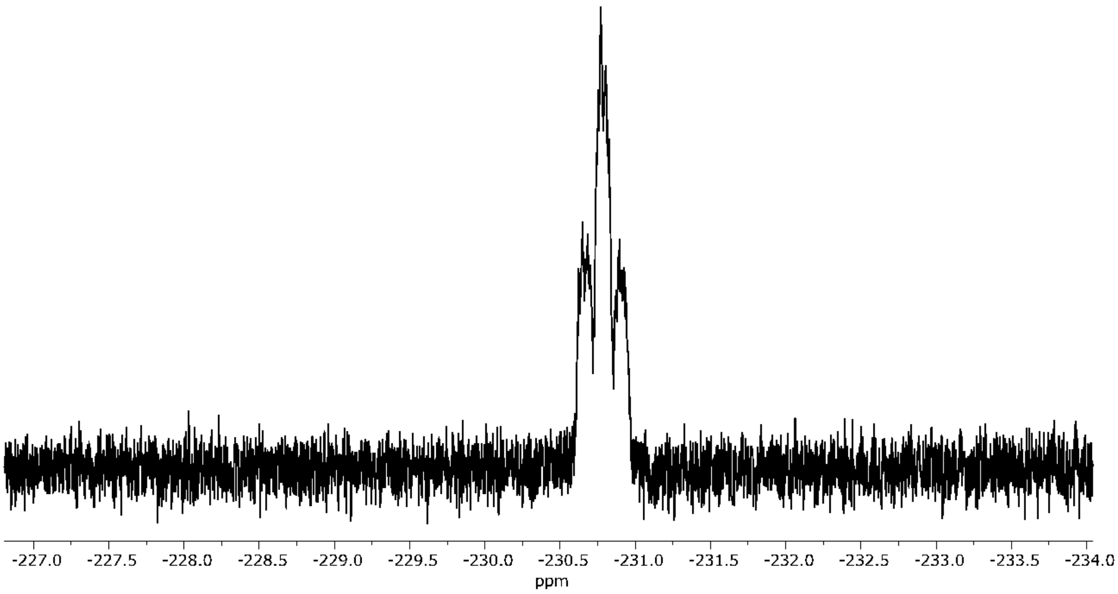

^{19}F NMR (377 MHz, CD_2Cl_2) of **39**.

5-Aminopentyl-(β-D-glucopyranosyl)-(1→4)-(α-D-glucopyranosyl)-(1→4)-6-deoxy-6-fluoro-α-D-galactopyranoside (5)

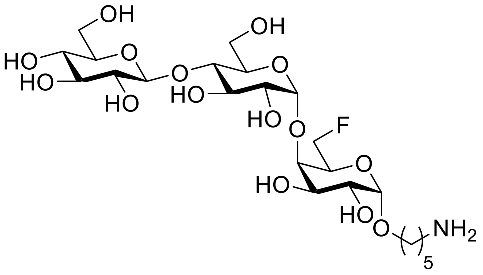

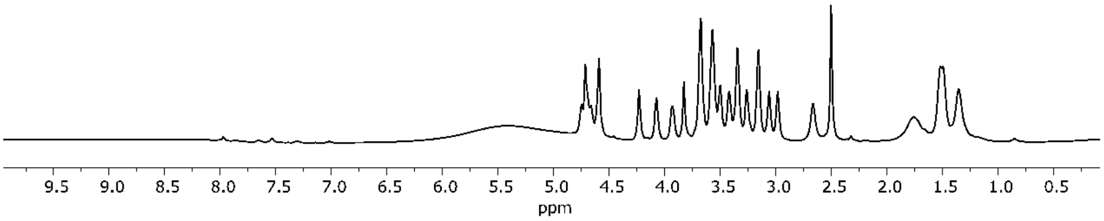

^{1}H NMR (800 MHz, DMSO-d_6) of **5**.

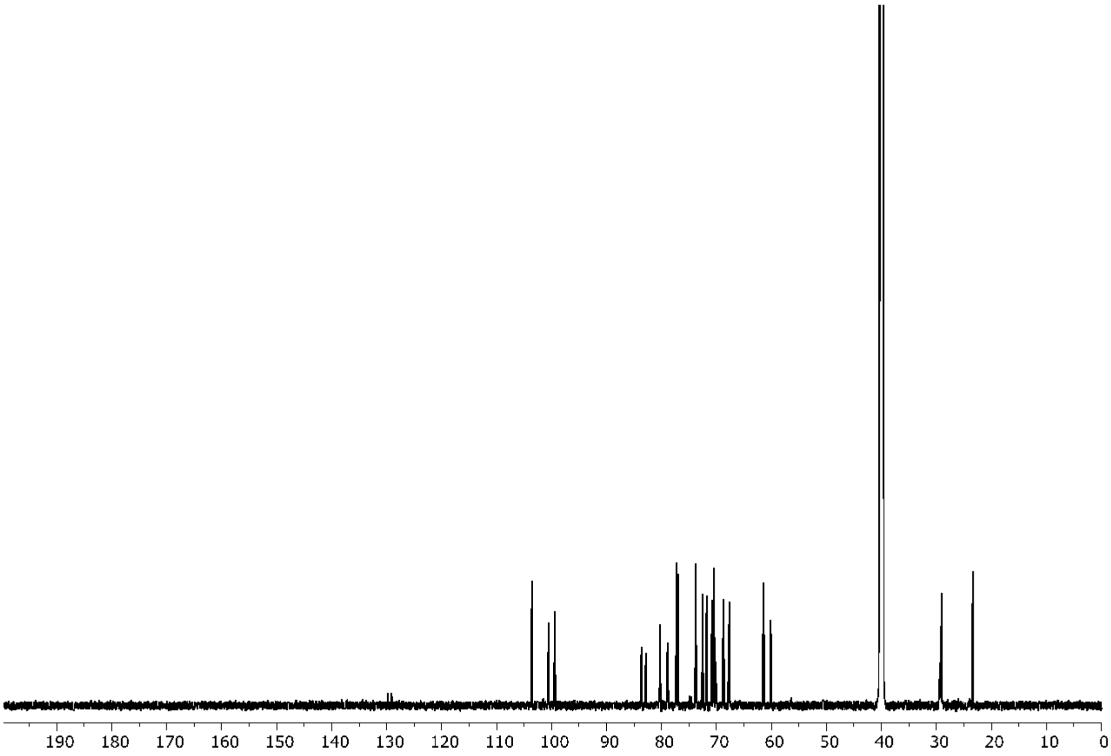

^{13}C NMR (200 MHz, DMSO-d_6) of **5**.

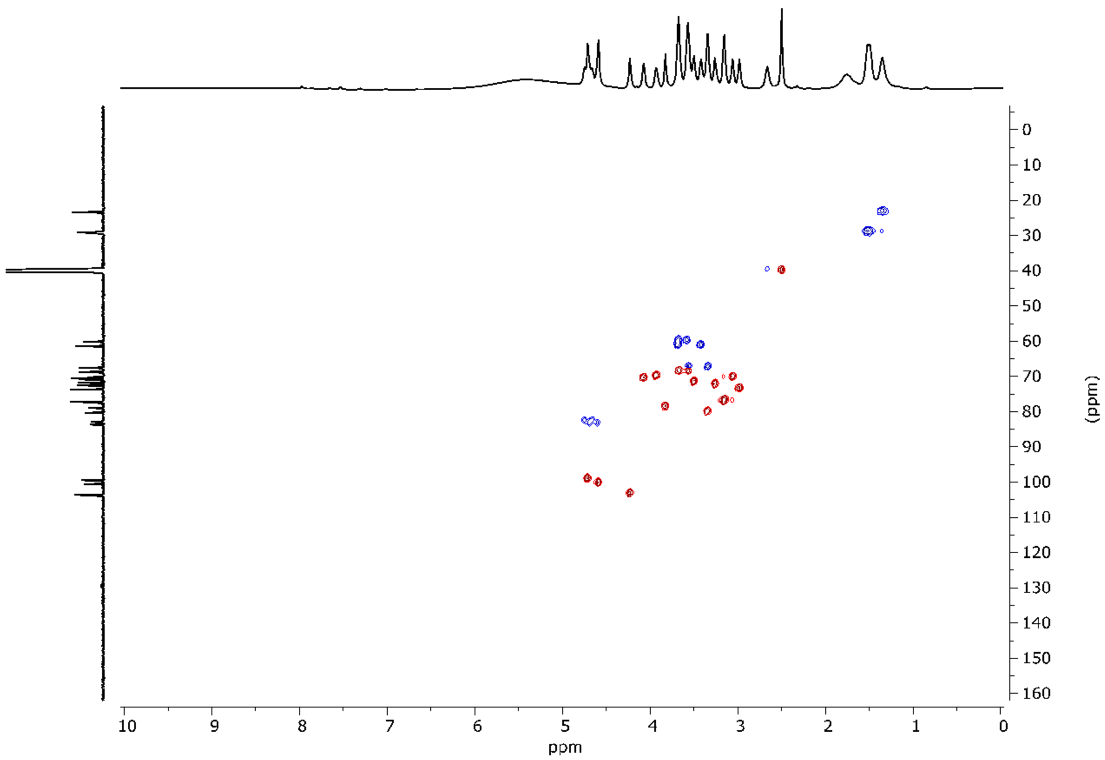

HSQC (DMSO-d$_6$) of **5**.

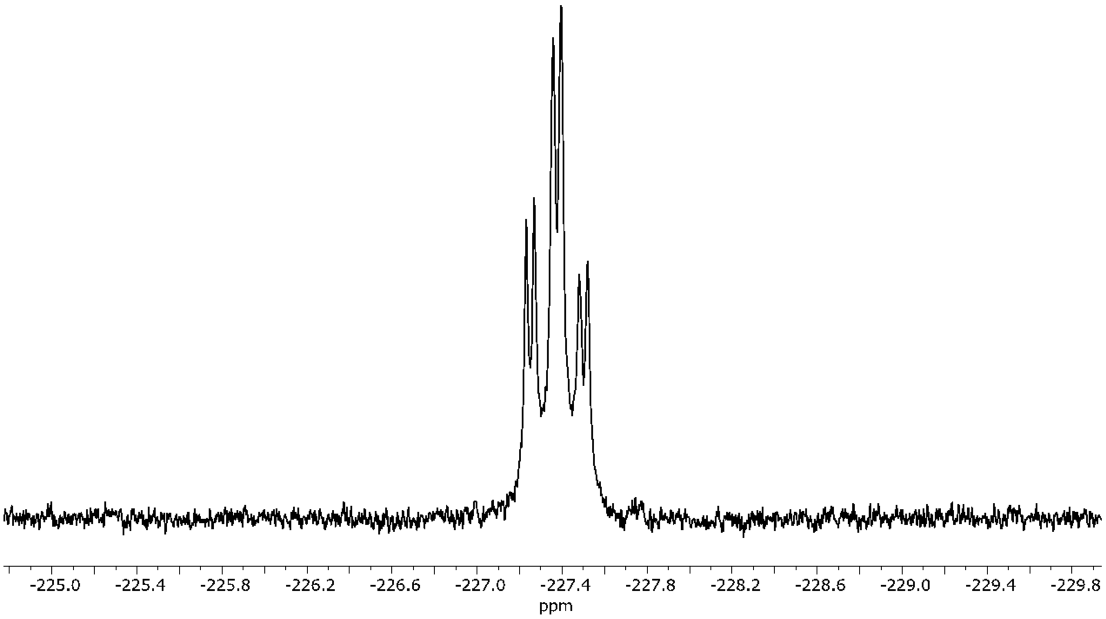

^{19}F NMR (377 MHz, DMSO-d$_6$) of **5**.

N-(Benzyl)-benzyloxycarbonyl-5-aminopentyl-(2,3,4,6-tetra-O-benzoyl-β-D-glucopyranosyl)-(1→4)-(2,3-di-O-benzyl-6-deoxy-6-fluoro-α-D-glucopyranosyl)-(1→4)-2,3,6-tri-O-benzyl-α-D-galactopyranoside (42)

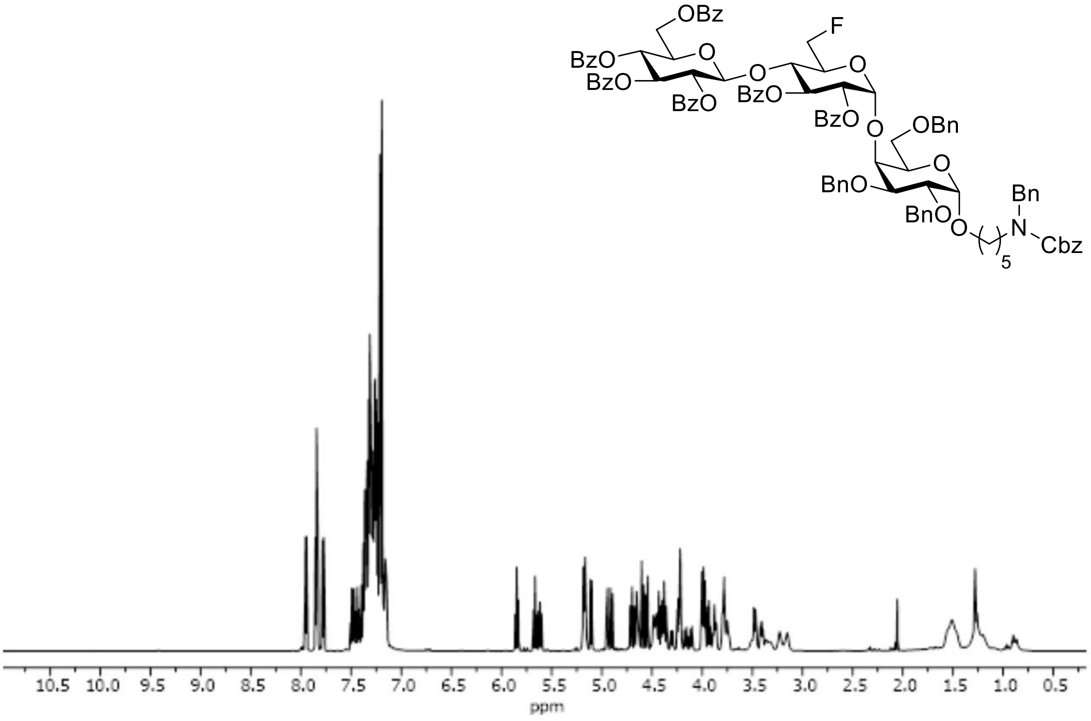

^{1}H NMR (800 MHz, $CDCl_3$) of **42**.

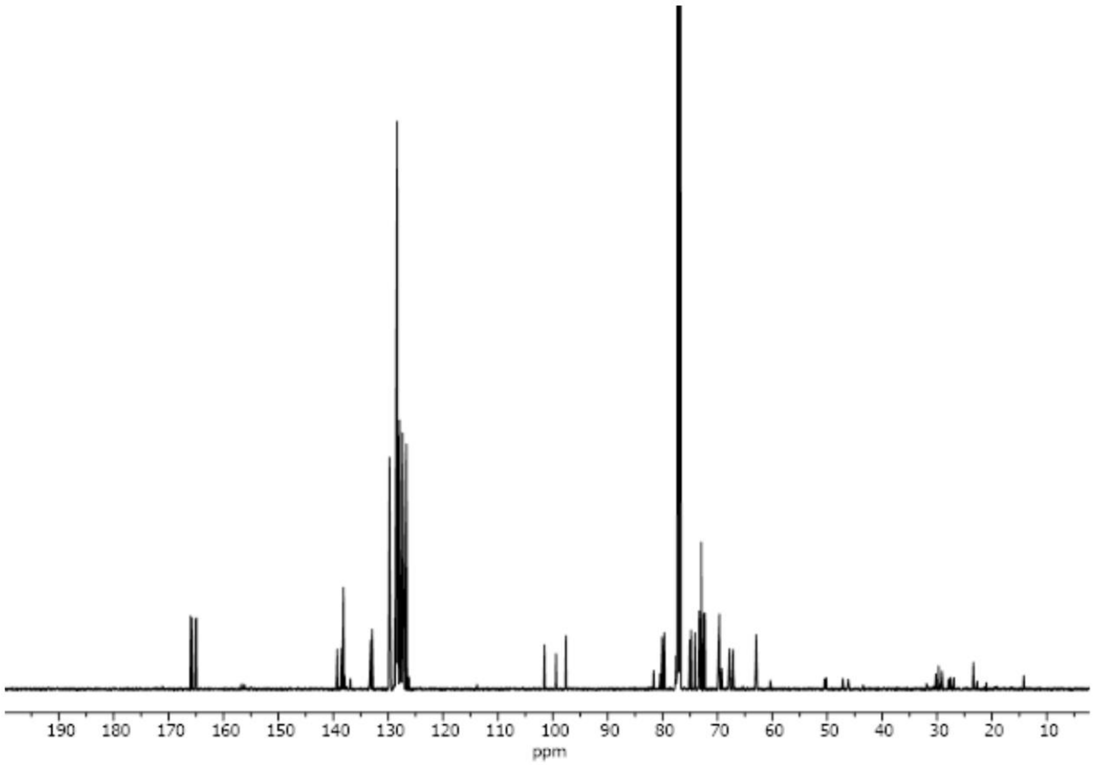

^{13}C NMR (200 MHz, $CDCl_3$) of **42**.

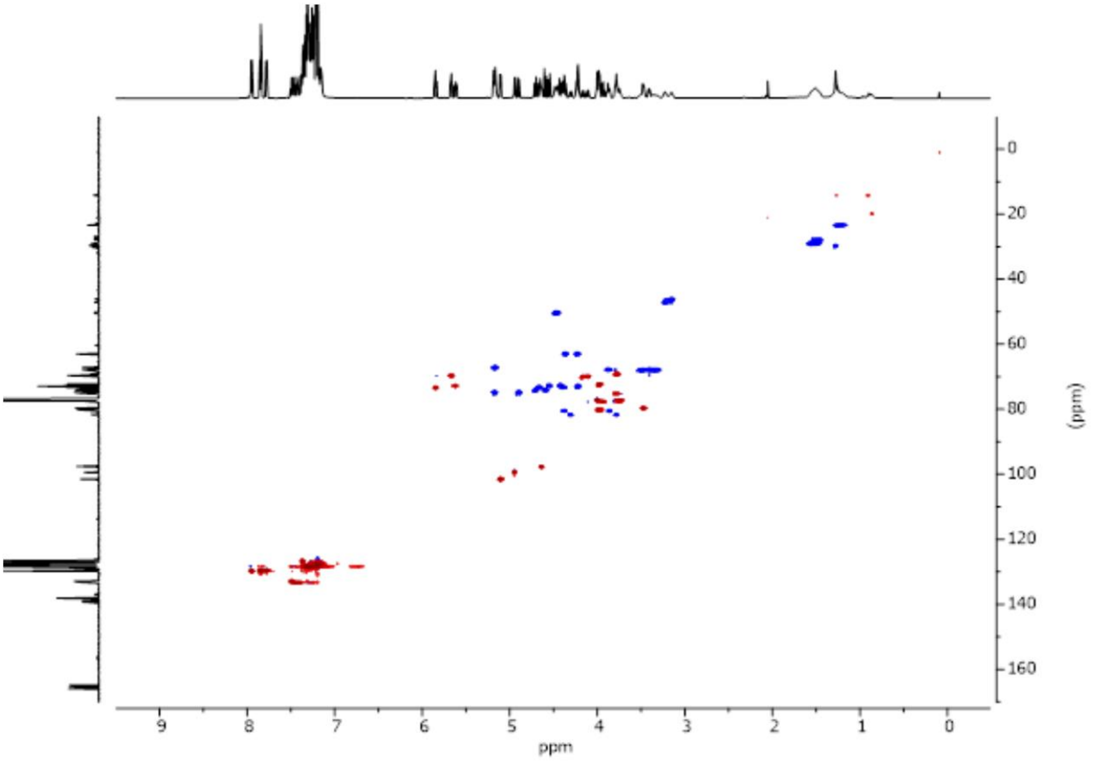

HSQC (CDCl$_3$) of **42**.

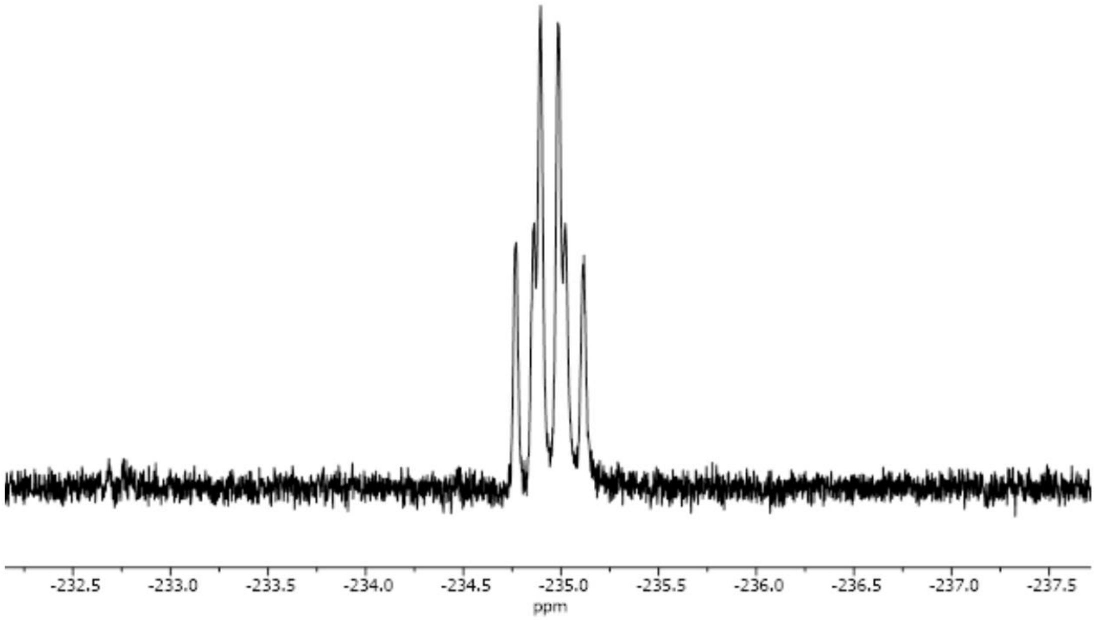

^{19}F NMR (377 MHz, CDCl$_3$) of **42**.

5-Aminopentyl-(β-D-glucopyranosyl)-(1→4)-(6-deoxy-6-fluoro-α-D-glucopyranosyl)-(1→4)-α-D-galactopyranoside (4)

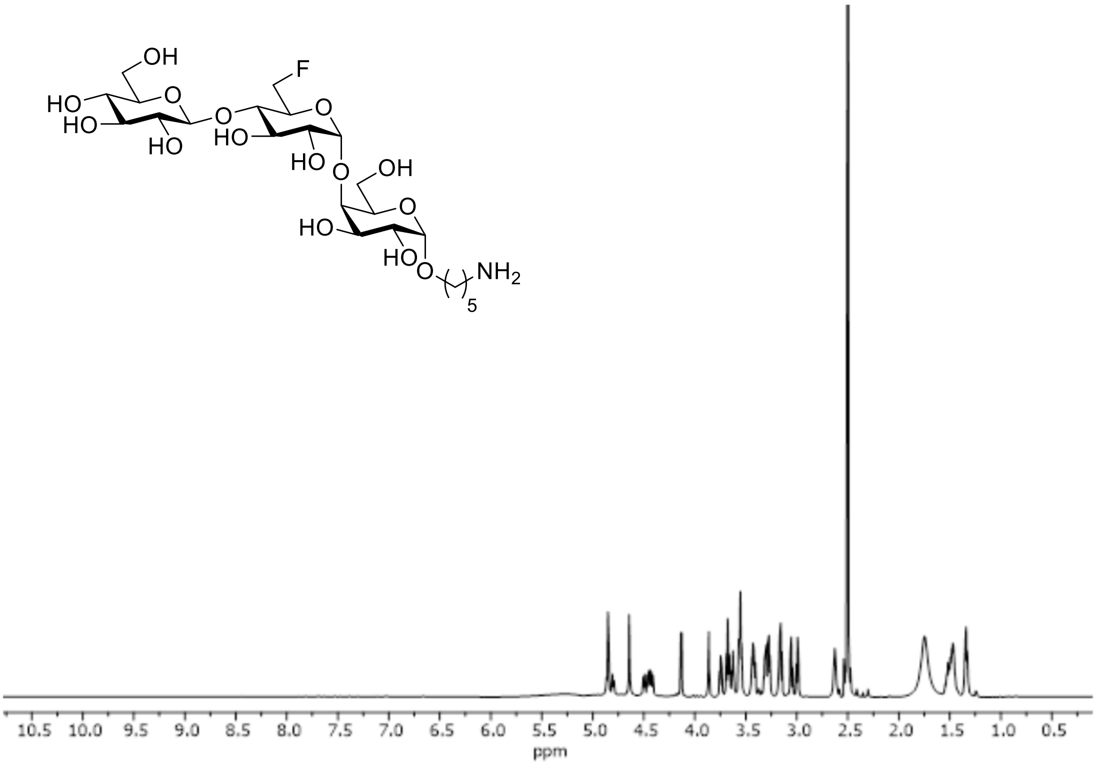

^{1}H NMR (800 MHz, DMSO-d_6) of **4**.

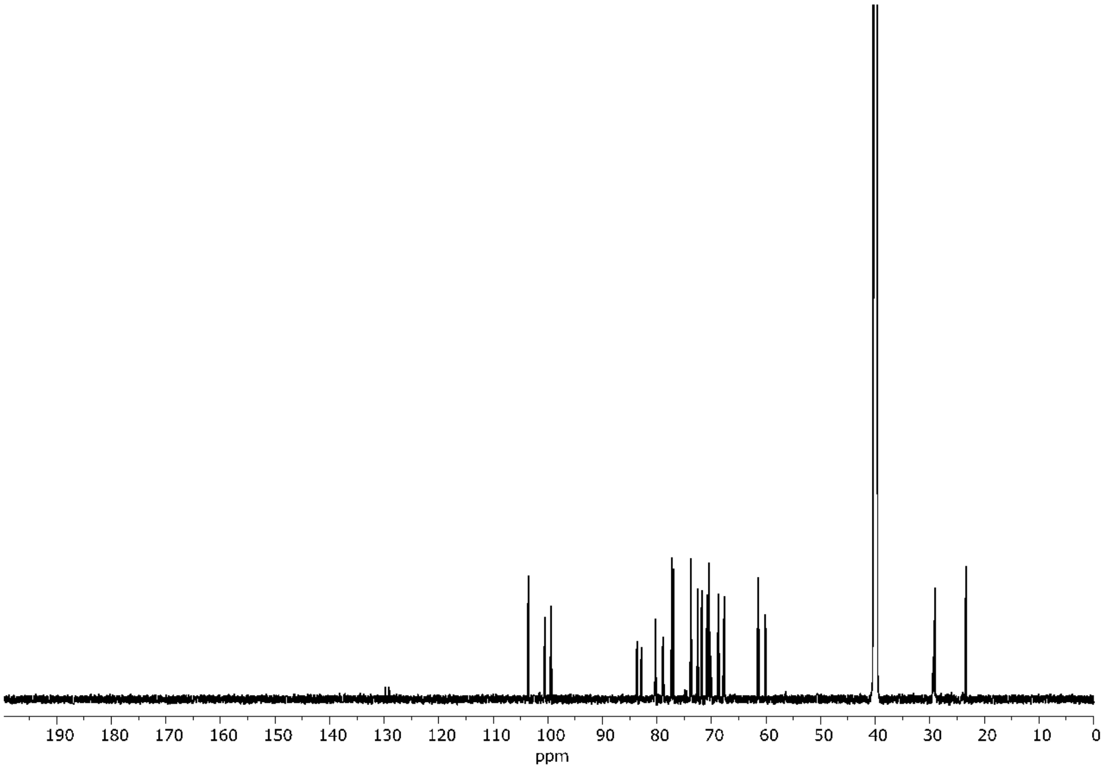

^{13}C NMR (200 MHz, DMSO-d_6) of **4**.

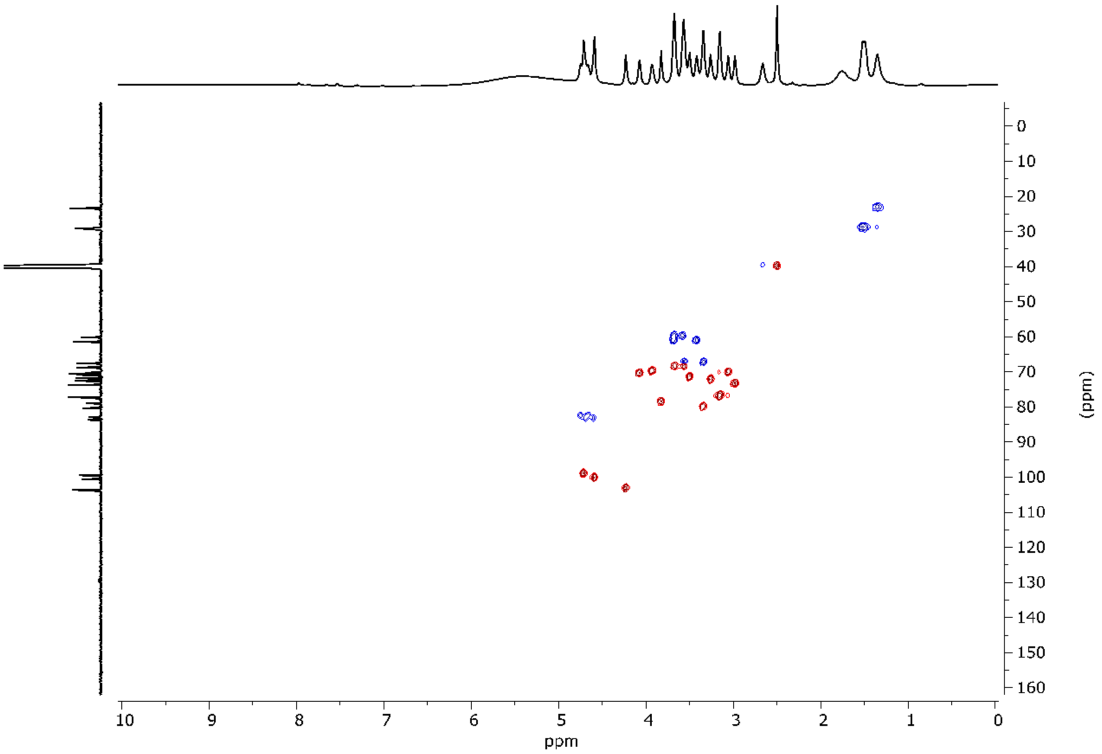

HSQC (DMSO-d$_6$) of **4**.

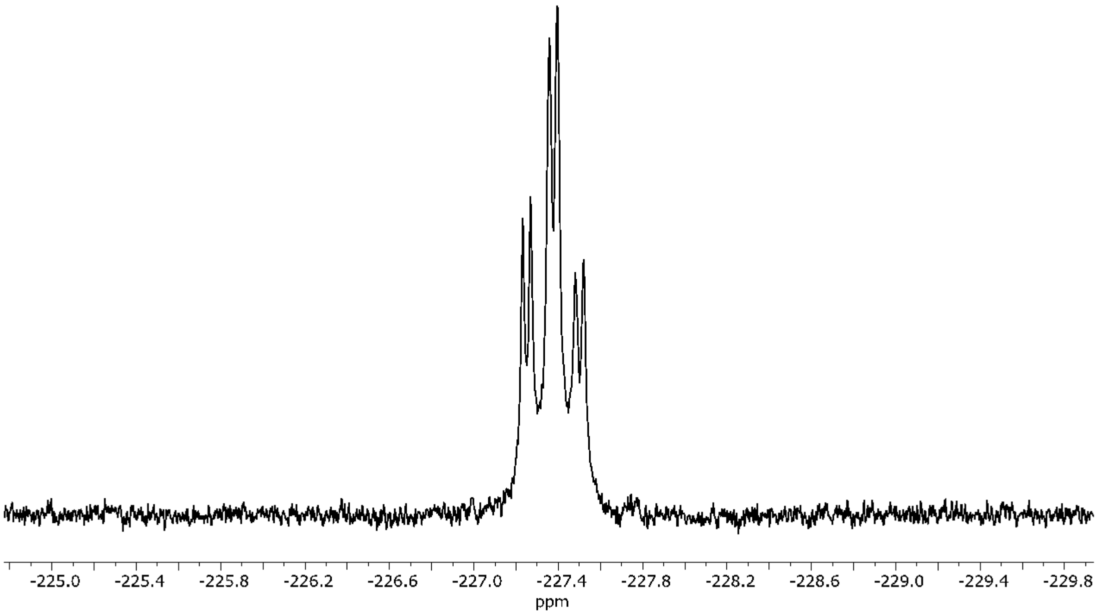

^{19}F NMR (377 MHz, DMSO-d$_6$) of **4**.

N-(Benzyl)benzyloxycarbonyl-5-aminopentyl-(2,3,4-tri-O-benzoyl-6-deoxy-6-fluoro-β-D-glucopyranosyl)-(1→4)-(2,3,6-tri-O-benzoyl-β-D-glucopyranosyl)-(1→4)-(2,3,6-tri-O-benzyl-α-D-glucopyranosyl)-(1→4)-2,3,6-tri-O-benzyl-α-D-galactopyranoside (43)

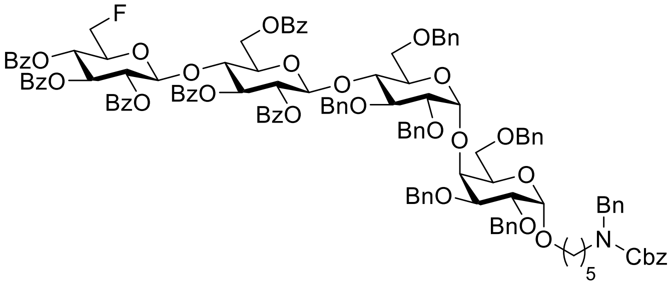

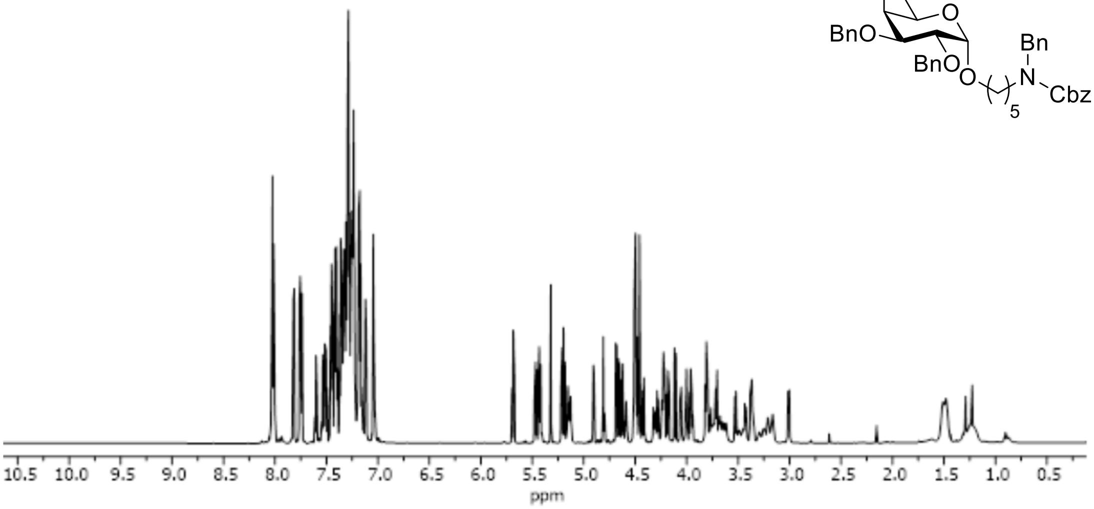

^{1}H NMR (800 MHz, CD_2Cl_2) of **43**.

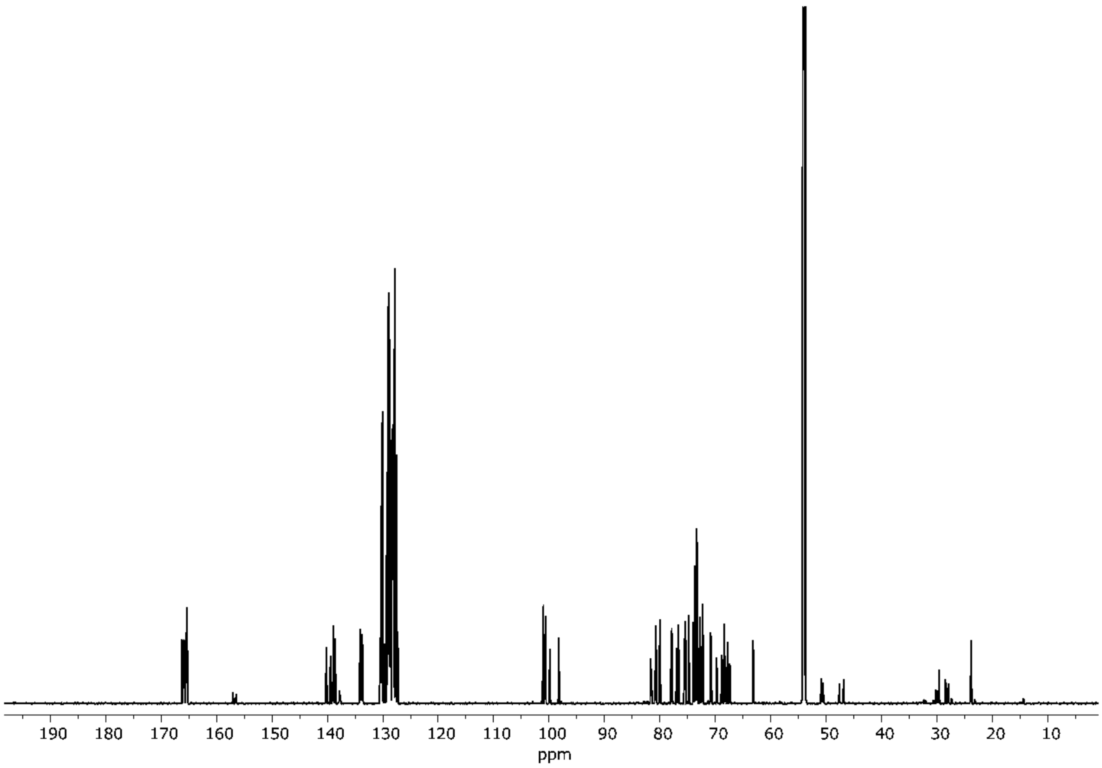

^{13}C NMR (200 MHz, CD_2Cl_2) of **43**.

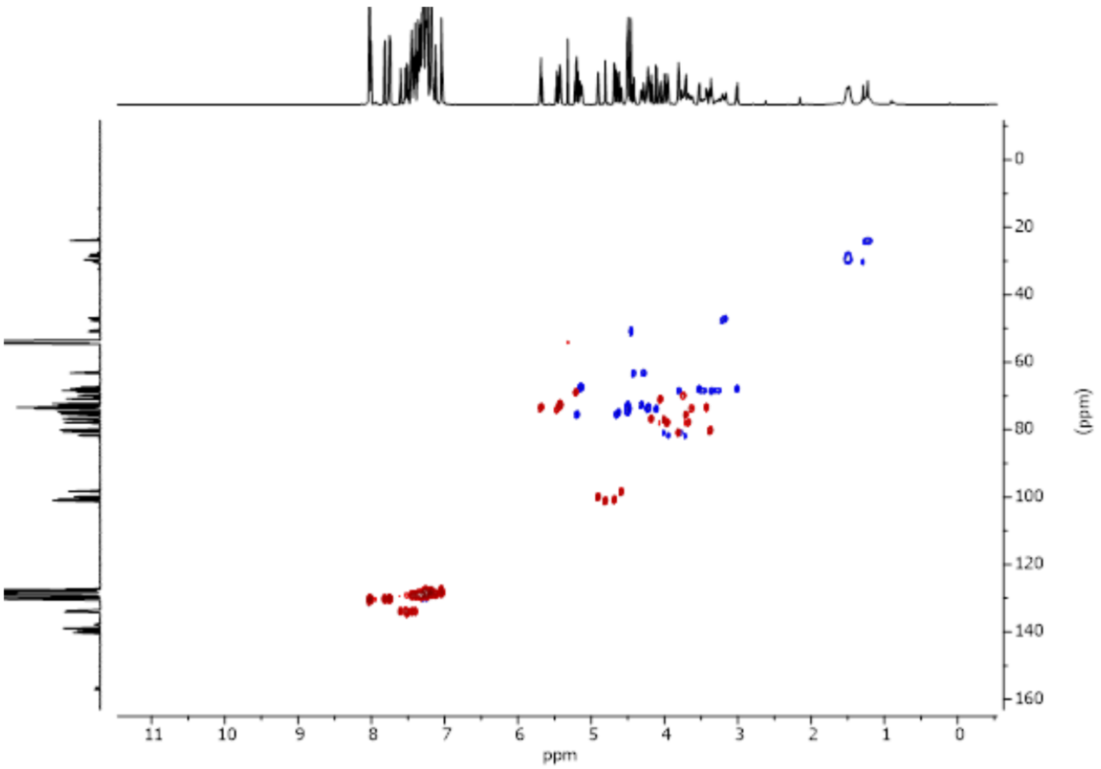

HSQC (CD_2Cl_2) of **43**.

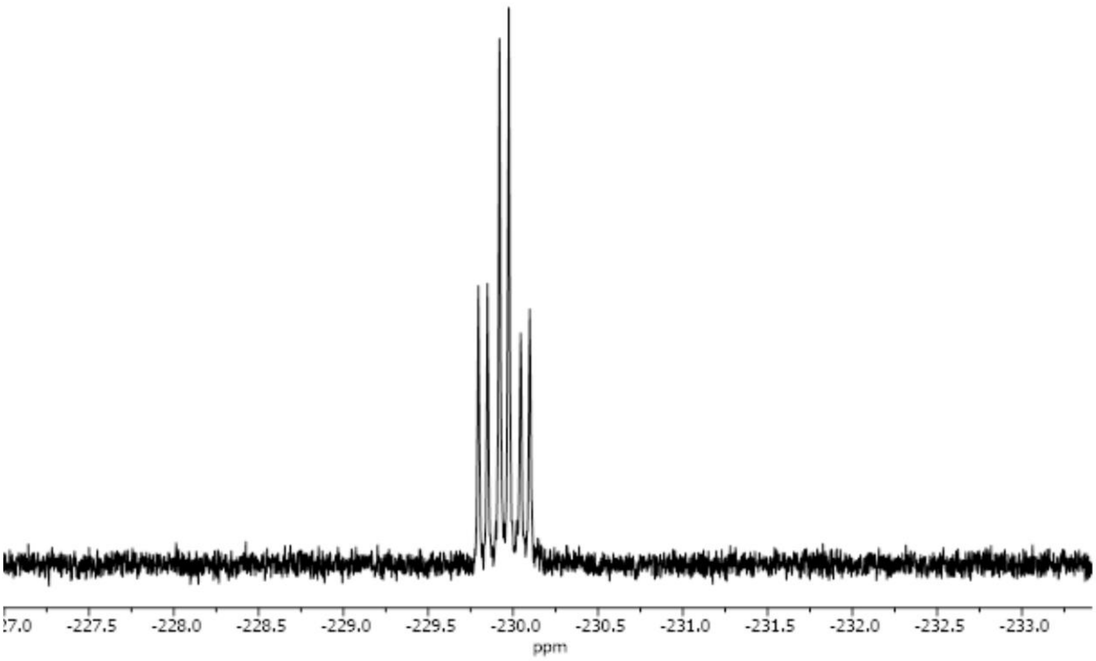

^{19}F NMR (377 MHz, CD_2Cl_2) of **43**.

5-Aminopentyl-(6-deoxy-6-fluoro-β-D-glucopyranosyl)-(1→4)-(β-D-glucopyrano-syl)-(1→4)-(α-D-glucopyranosyl)-(1→4)-α-D-galactopyranoside (6)

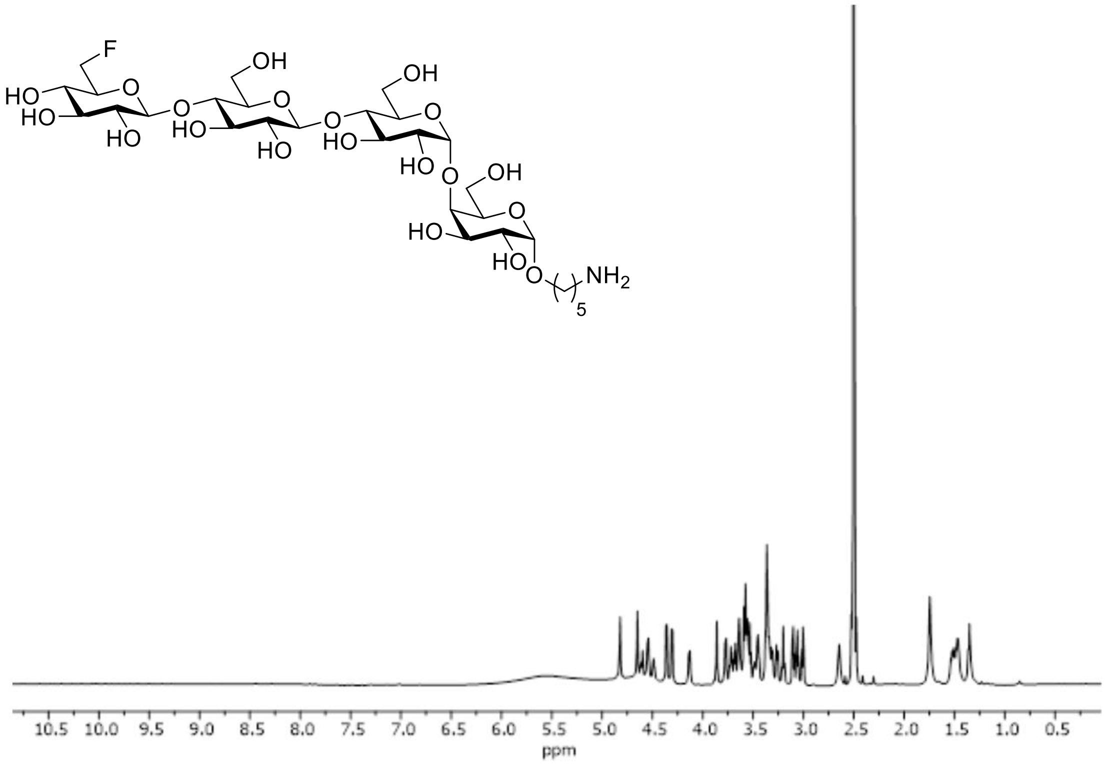

^{1}H NMR (800 MHz, DMSO-d$_6$) of **6**.

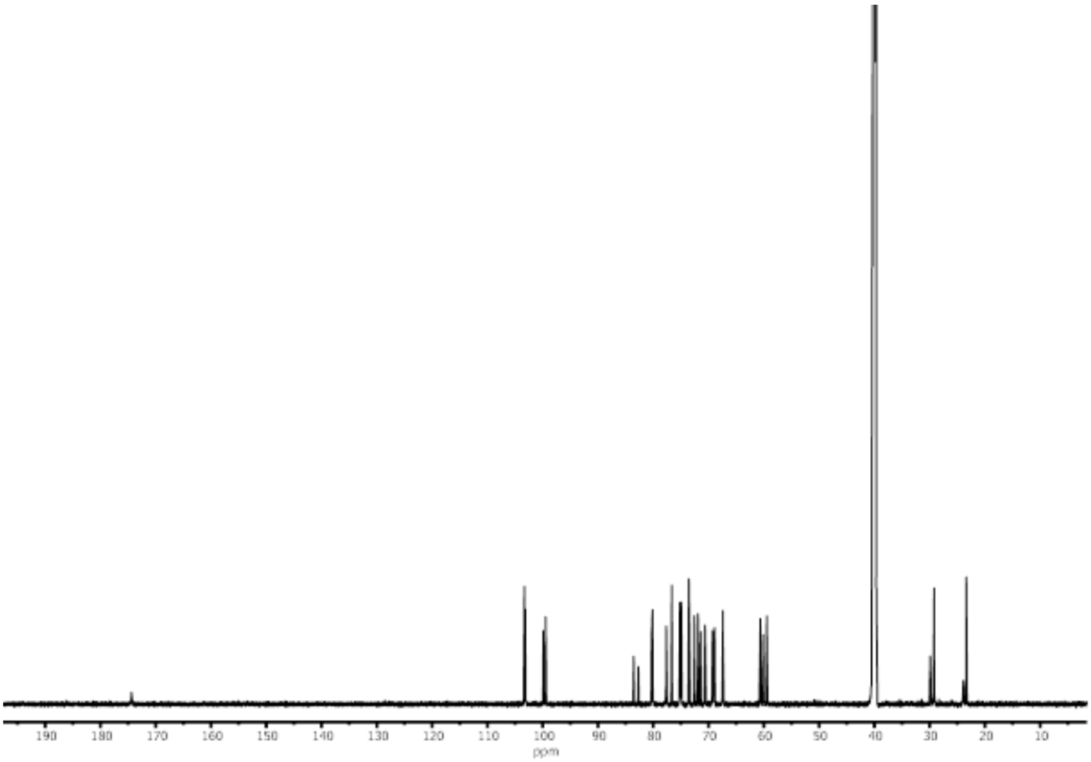

^{13}C NMR (200 MHz, DMSO-d$_6$) of **6**.

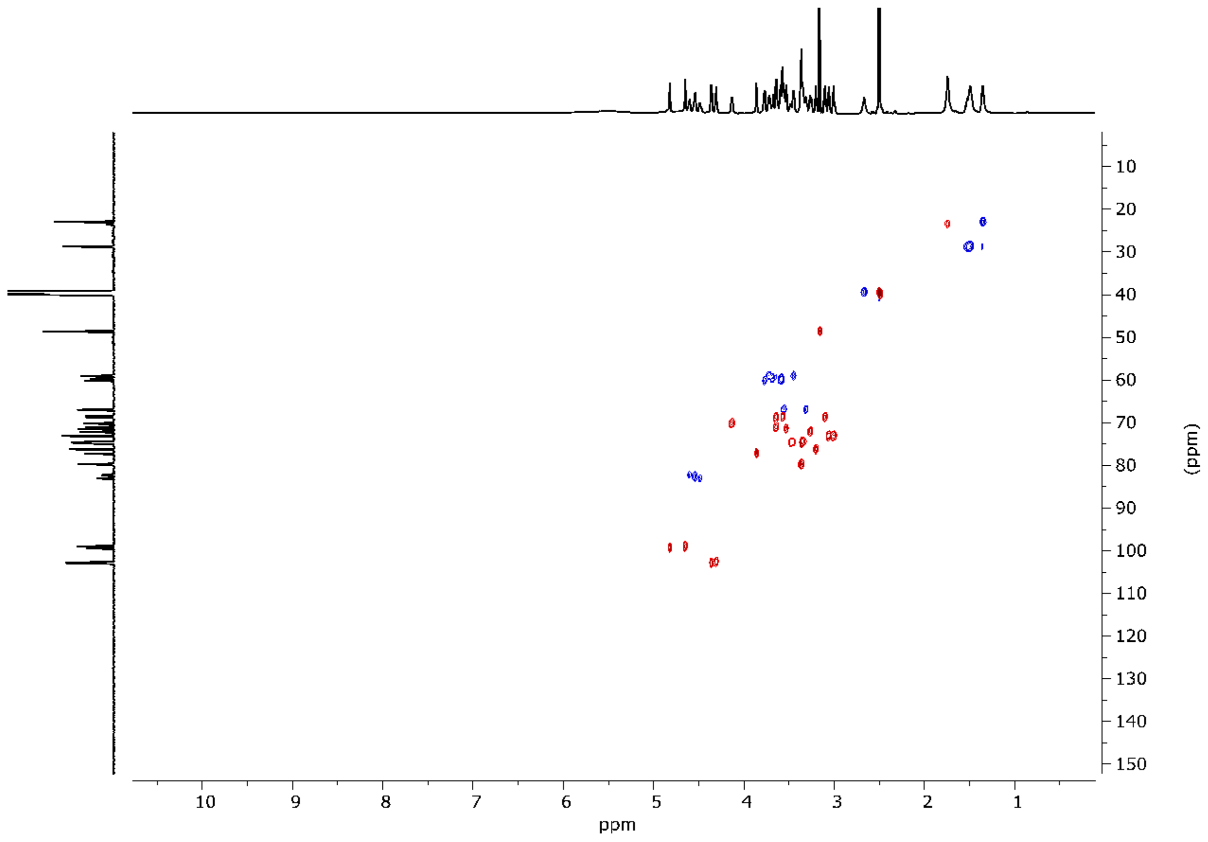

HSQC NMR (DMSO-d6) of **6**.

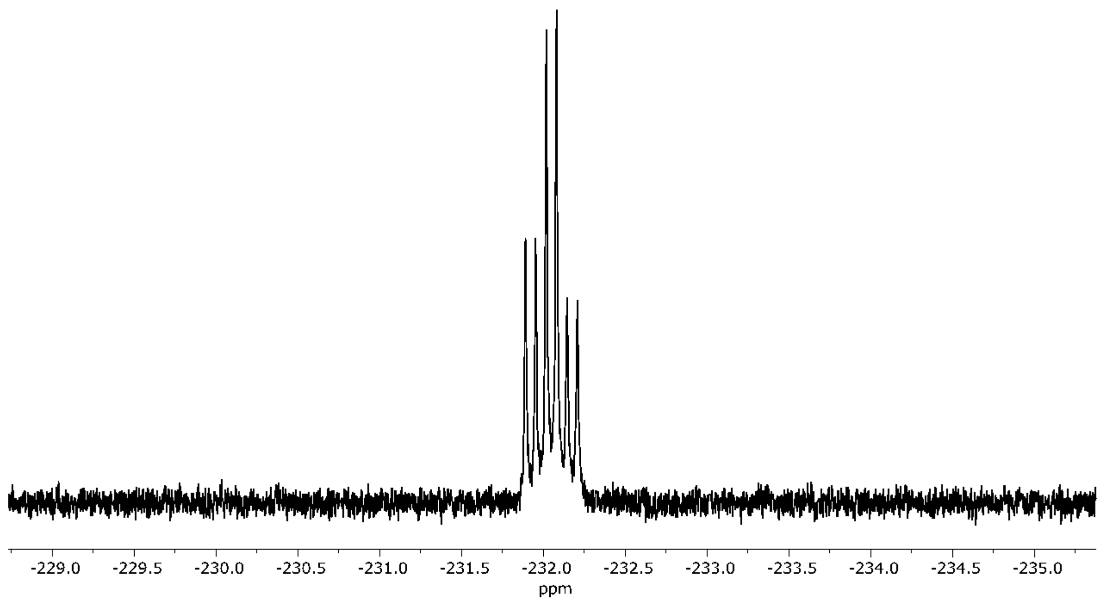

^{19}F NMR (377 MHz, DMSO-d6) of **6**.

***N*-(Benzyl)-benzyloxycarbonyl-5-aminopentyl-(2,3,4,6-tetra-*O*-benzoyl-β-D-glucopyranosyl)-(1→4)-(2,3-di-*O*-benzoyl-6-deoxy-6-fluoro-β-D-glucopyranosyl)-(1→4)-(2,3,6-tri-*O*-benzyl-α-D-glucopyranosyl)-(1→4)-2,3,6-tri-*O*-benzyl-α-D-galactopyranoside (44)**

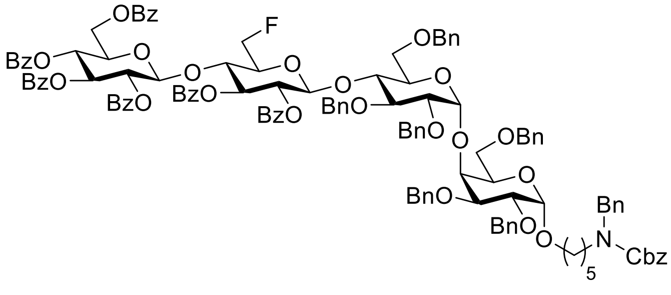

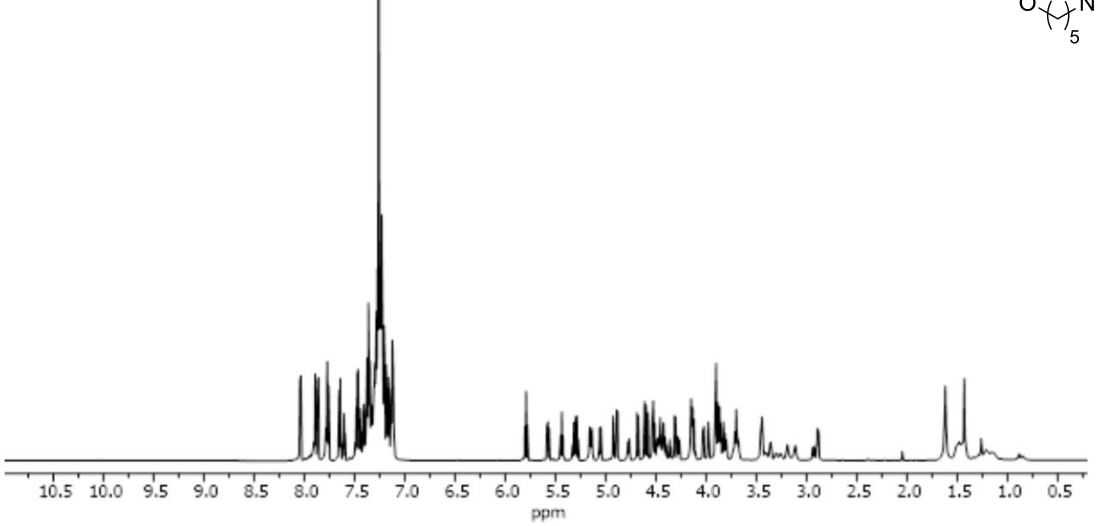

^{1}H NMR (800 MHz, $CDCl_3$) of **44**.

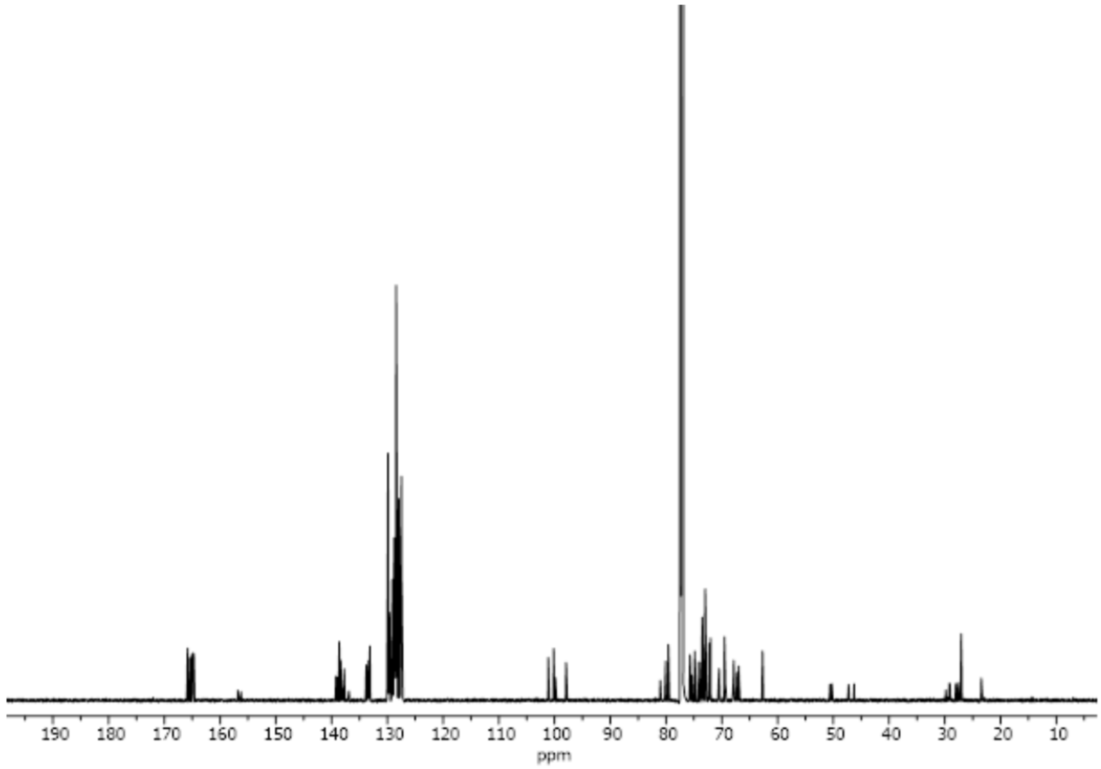

^{13}C NMR (200 MHz, $CDCl_3$) of **44**.

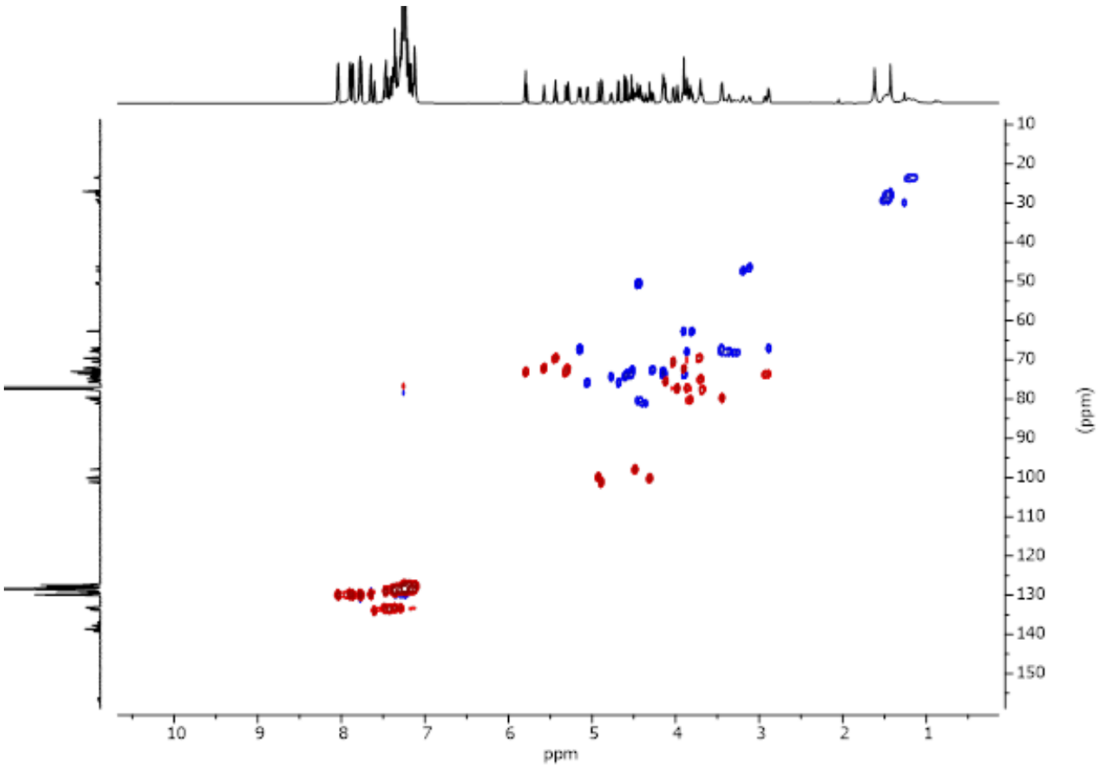

HSQC (CDCl$_3$) of **44**.

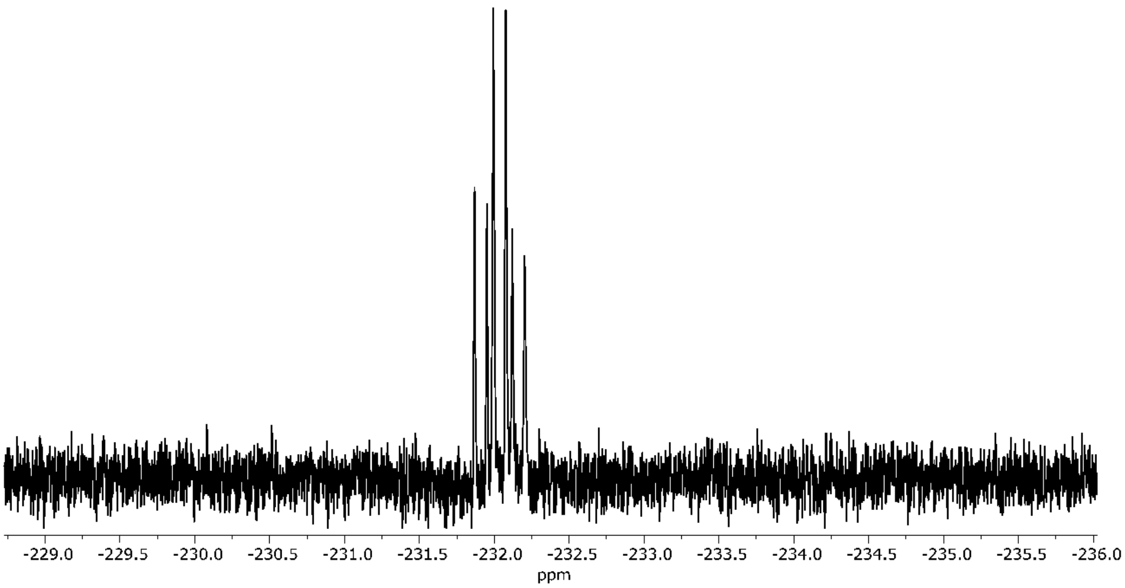

^{19}F NMR (377 MHz, CDCl$_3$) of **44**.

5-Aminopentyl-(β-D-glucopyranosyl)-(1→4)-(6-deoxy-6-fluoro-β-D-glucopyranosyl)-(1→4)-(α-D-glucopyranosyl)-(1→4)-α-D-galactopyranoside (7)

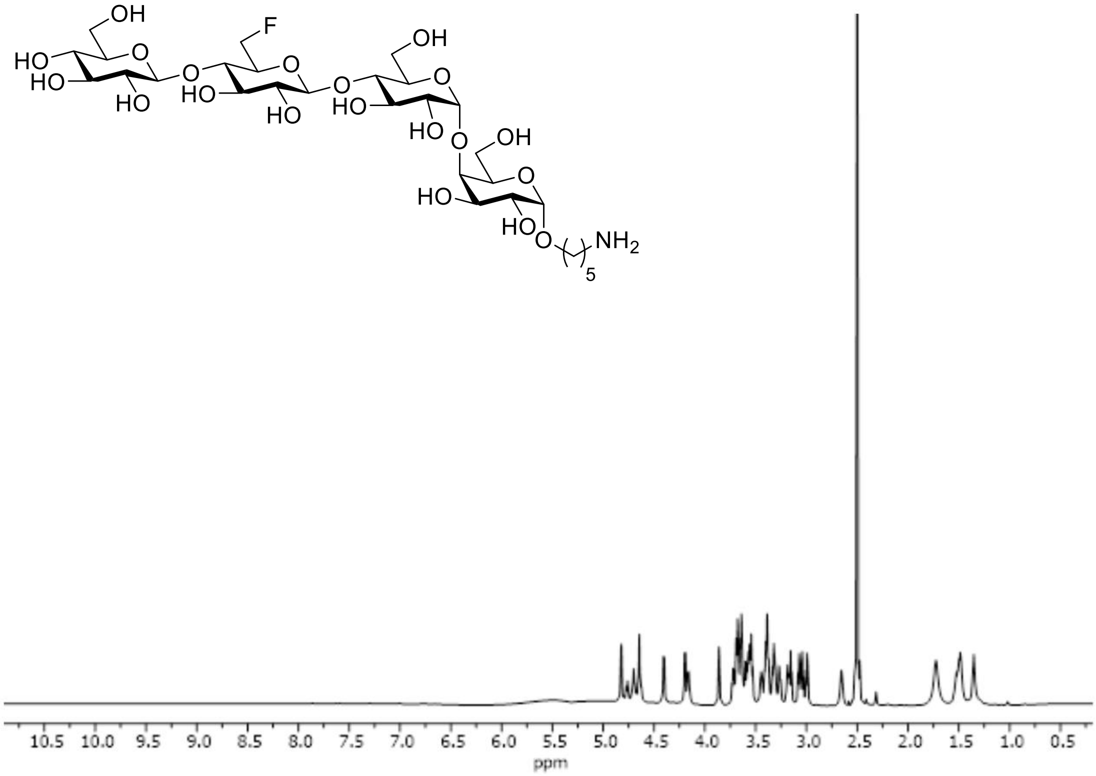

^{1}H NMR (800 MHz, DMSO-d_6) of **7**.

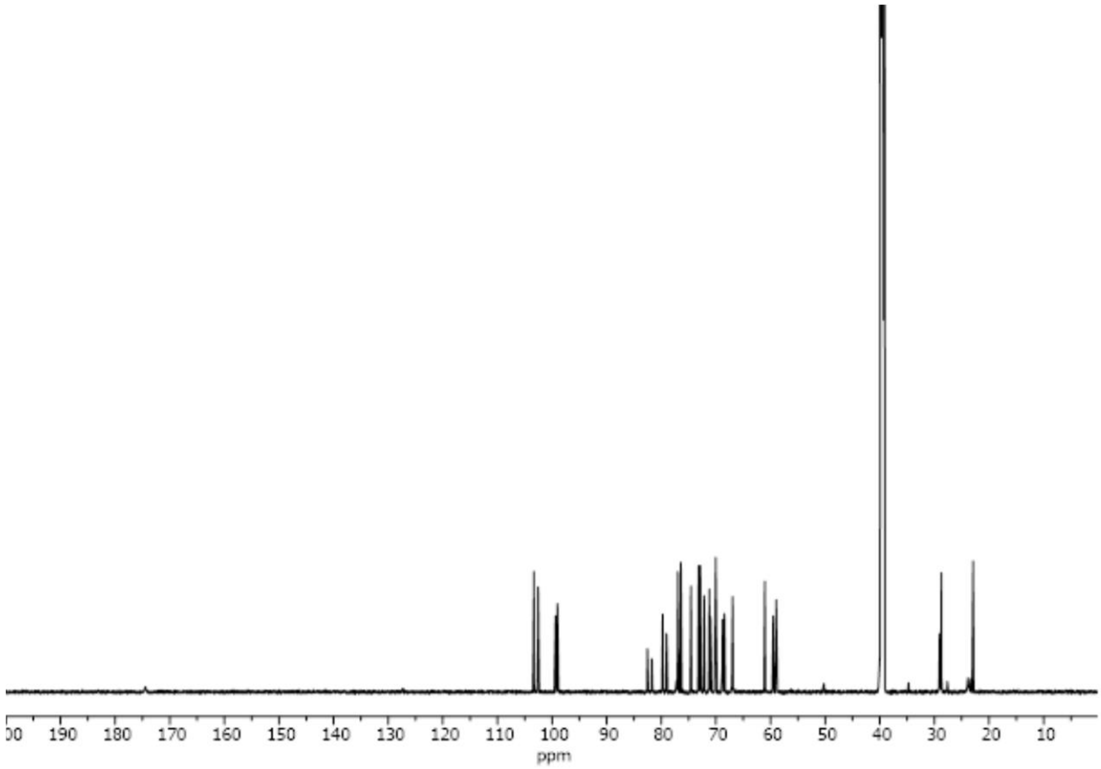

^{13}C NMR (200 MHz, DMSO-d_6) of **6**.

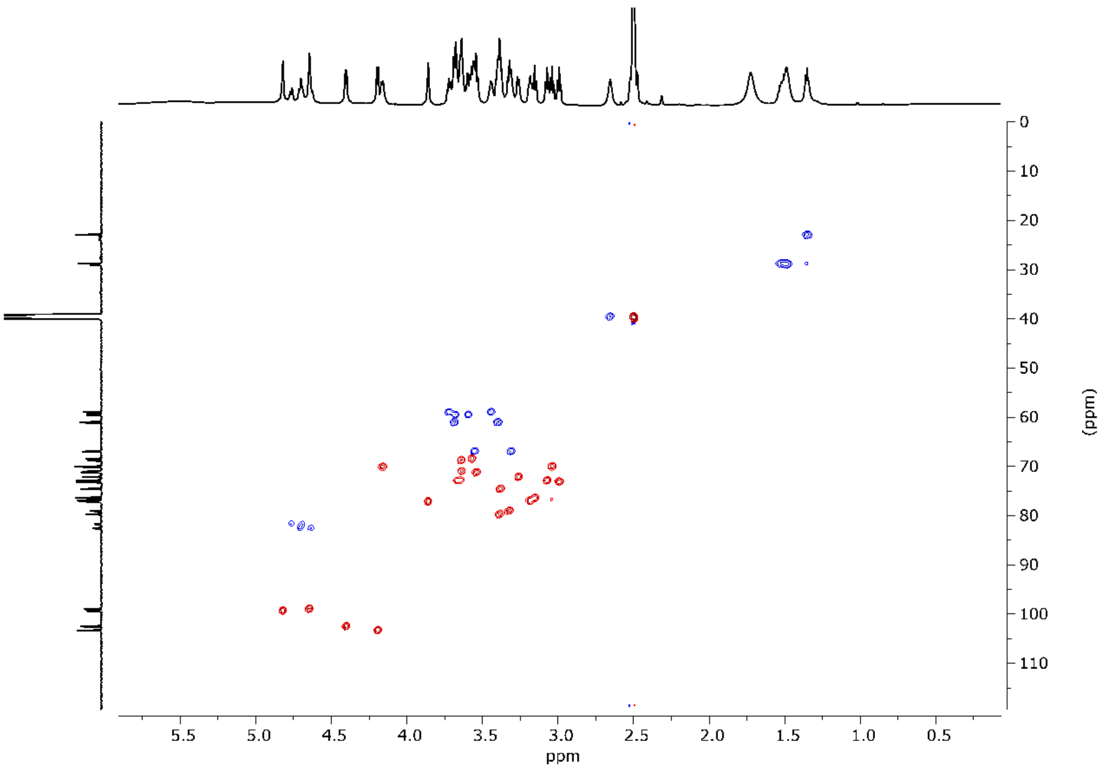

HSQC NMR (DMSO-d$_6$) of **7**.

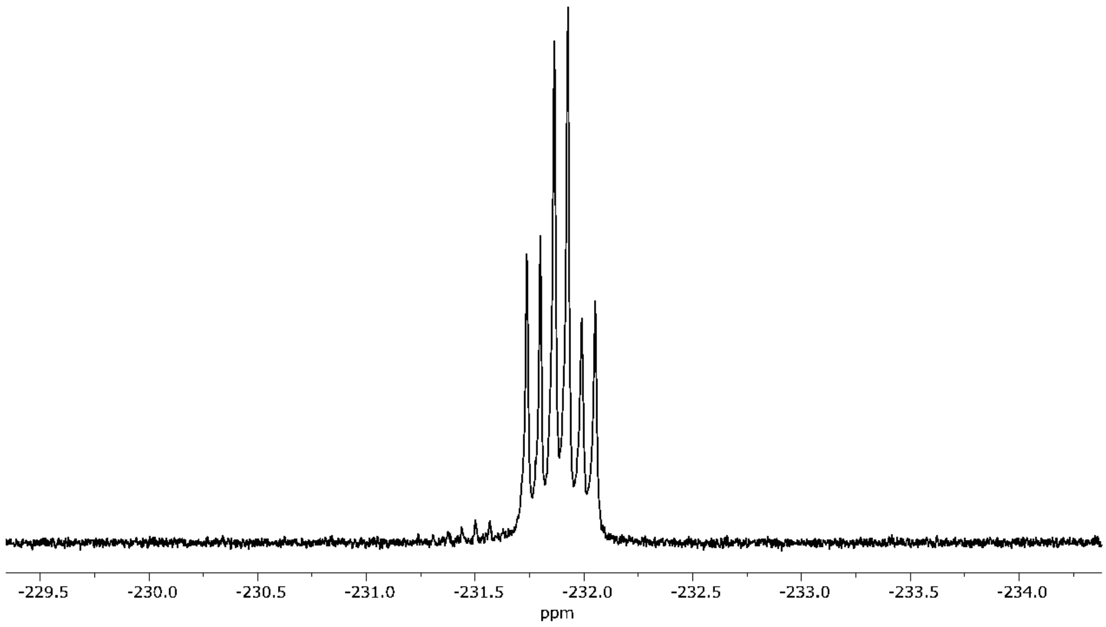

^{19}F NMR (377 MHz, DMSO-d$_6$) of **7**.

N-(Benzyl)benzyloxycarbonyl-5-aminopentyl-(2,3,4,6-tetra-O-benzoyl-β-D-glucopyranosyl)-(1→4)-(2,3,6-tri-O-benzoyl-β-D-glucopyranosyl)-(1→4)-(2,3,6-tri-O-benzyl-α-D-glucopyranosyl)-(1→4)-2,3-di-O-benzyl-6-deoxy-6-fluoro-α-D-galactopyranoside (45)

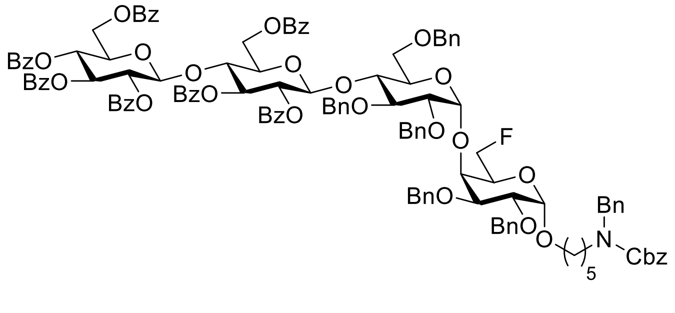

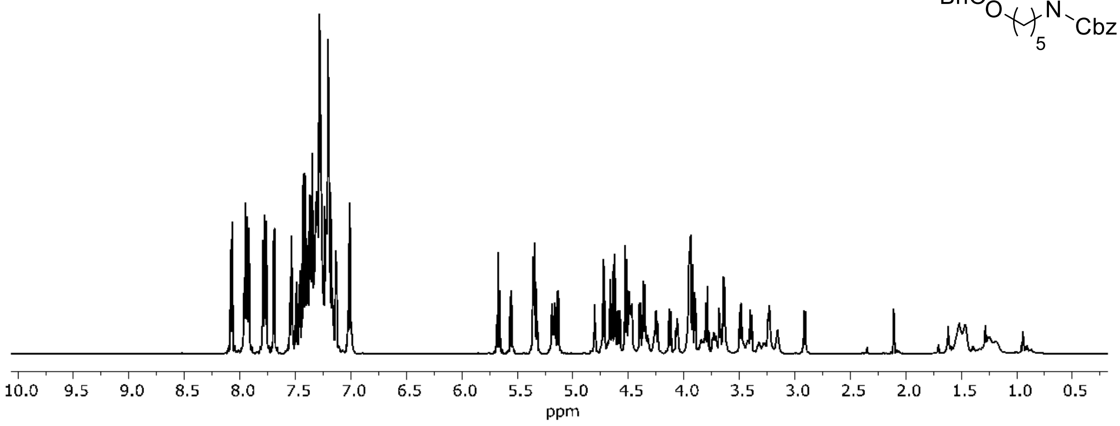

^{1}H NMR (800 MHz, $CDCl_3$) of **45**.

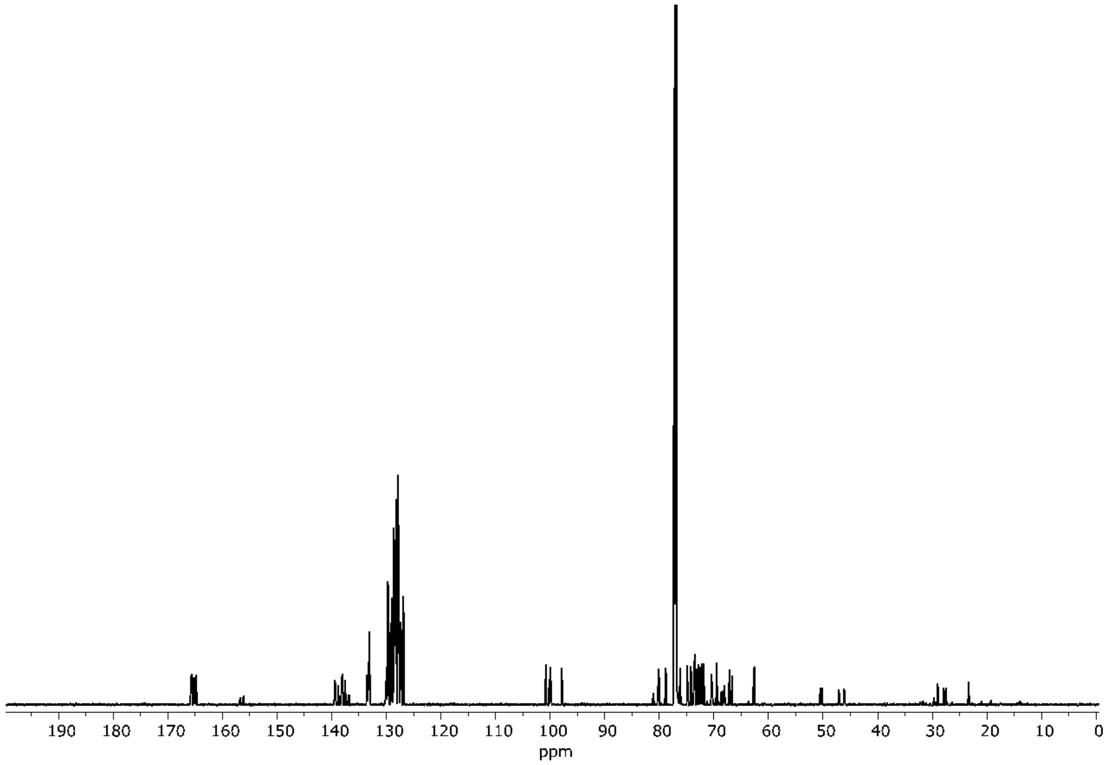

^{13}C NMR (200 MHz, $CDCl_3$) of **45**.

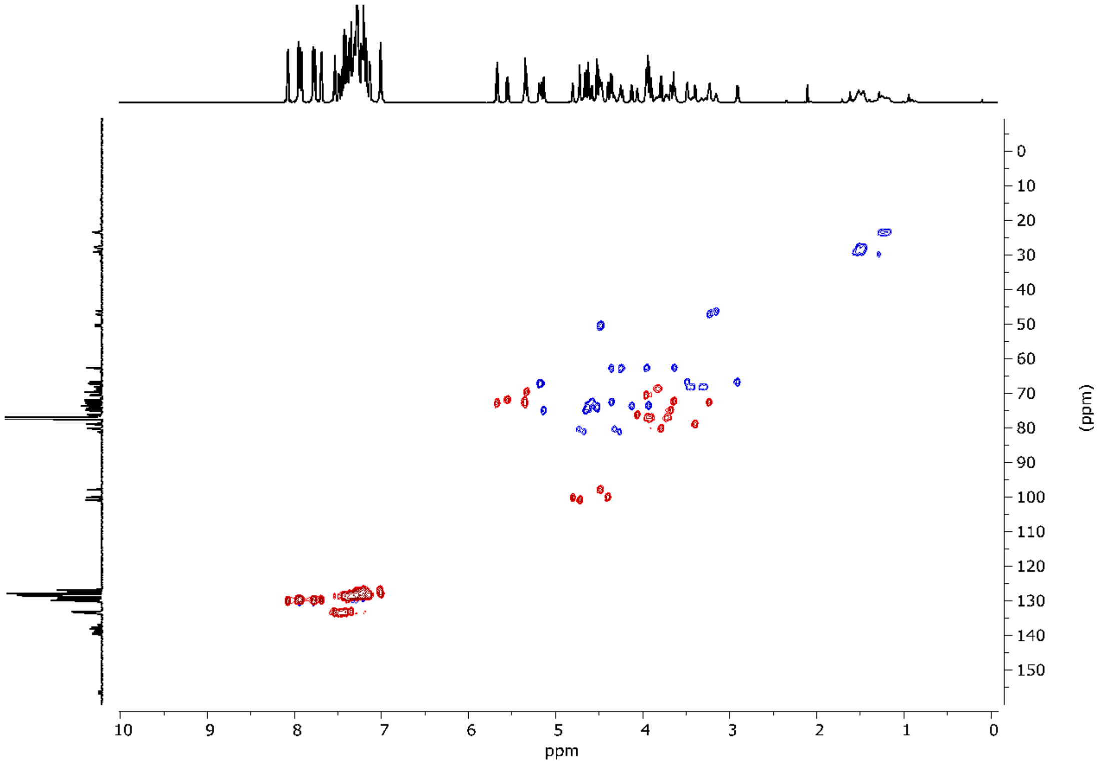

HSQC ($CDCl_3$) of **45**.

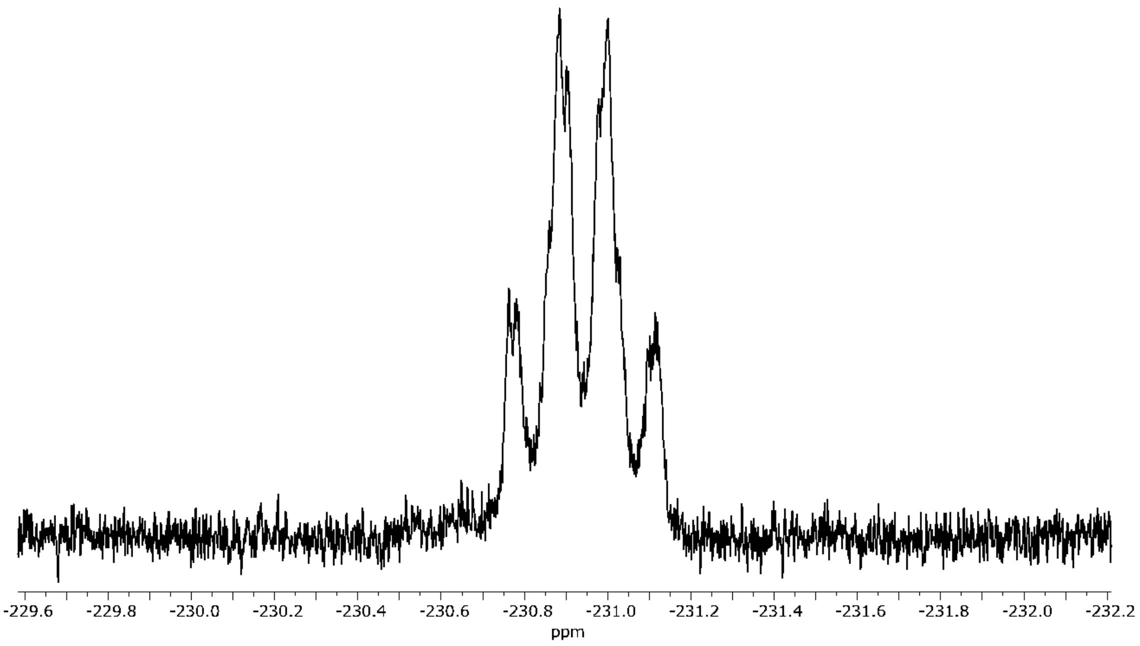

^{19}F NMR (377 MHz, $CDCl_3$) of **45**.

5-Aminopentyl-(β-D-glucopyranosyl)-(1→4)-(β-D-glucopyranosyl)-(1→4)-(α-D-gluco-pyranosyl)-(1→4)-6-deoxy-6-fluoro-α-D-galactopyranoside (9)

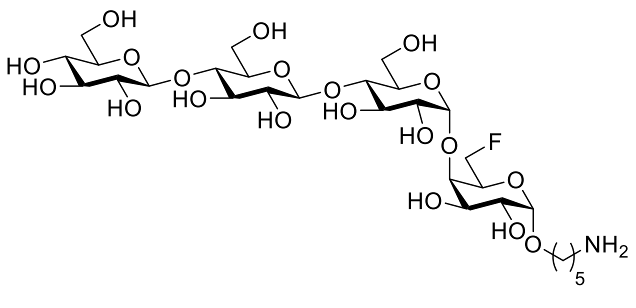

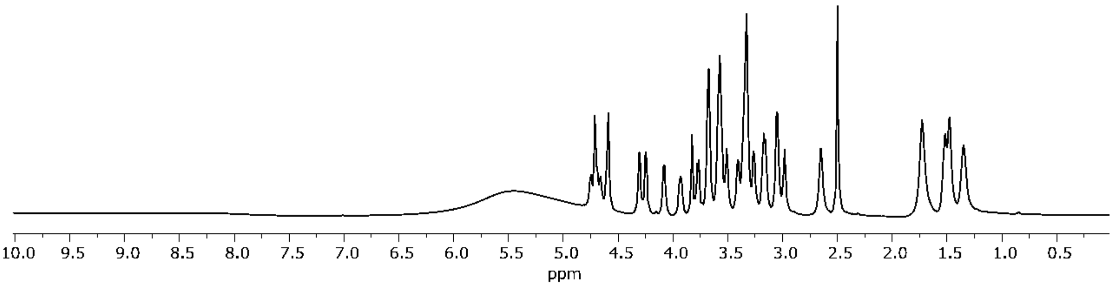

^{1}H NMR (800 MHz, DMSO-d_6) of **9**.

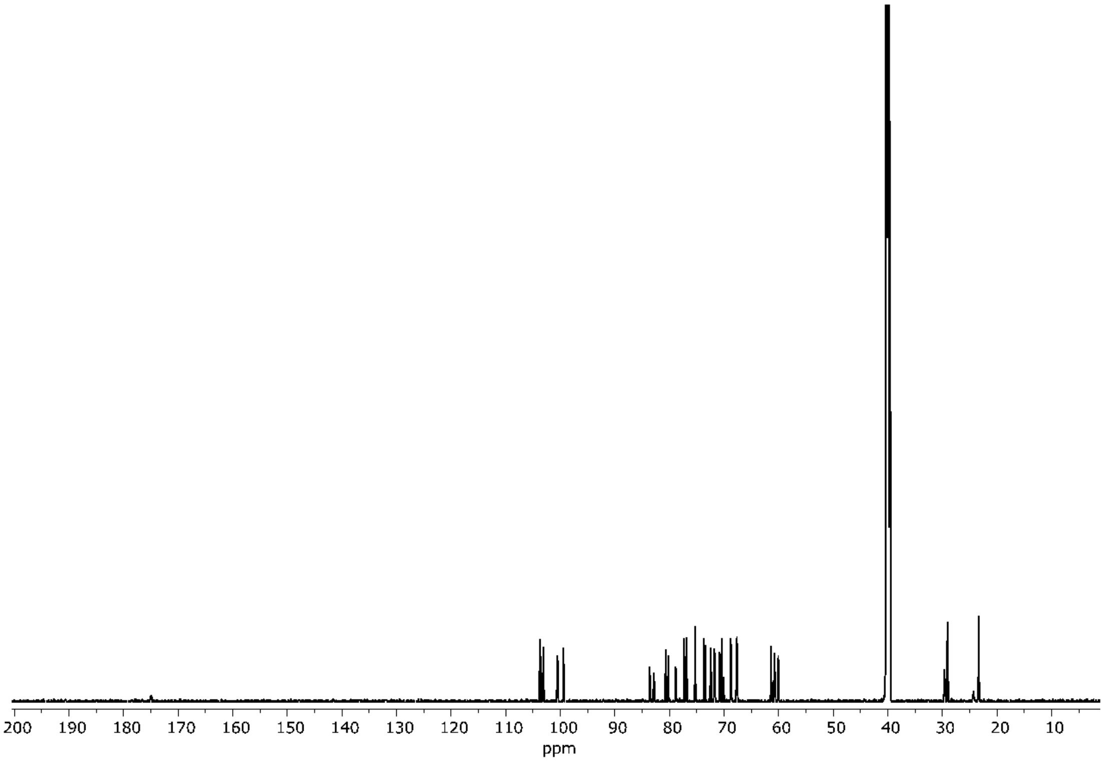

^{13}C NMR (200 MHz, DMSO-d_6) of **9**.

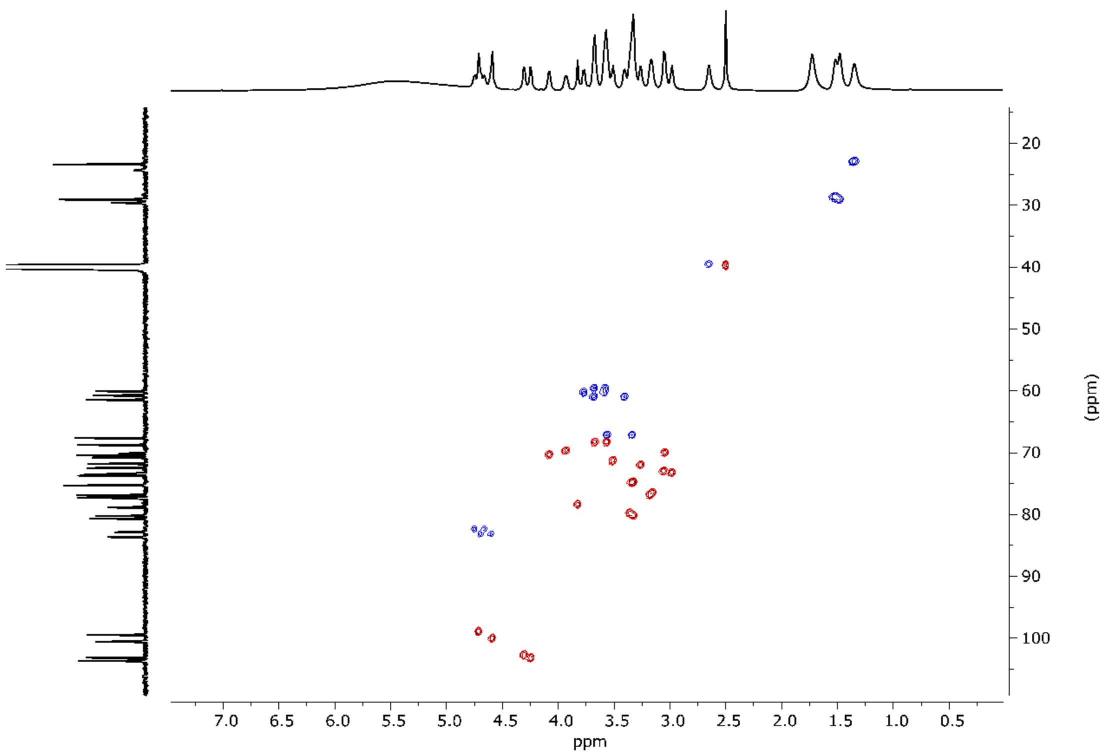

HSQC (DMSO-d_6) of **9**.

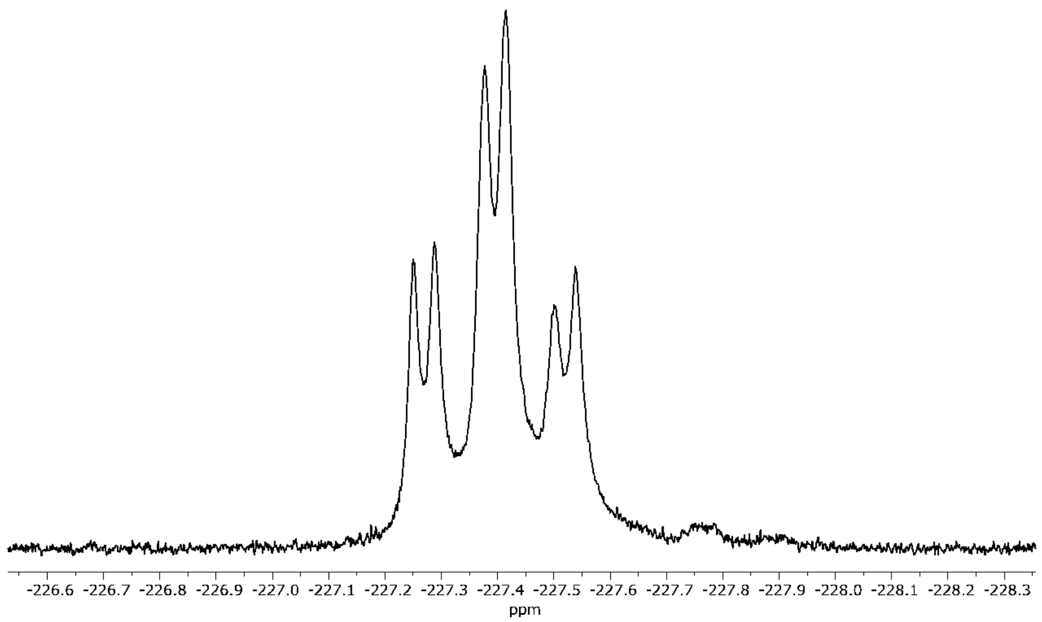

^{19}F NMR (377 MHz, DMSO-d_6) of **9**.

_N_-(Benzyl)-benzyloxycarbonyl-5-aminopentyl-(2,3,4,6-tetra-_O_-benzoyl-β-D-gluco-pyranosyl)-(1→4)-(2,3,6-tri-_O_-benzoyl-β-D-glucopyranosyl)-(1→4)-(2,3-di-_O_-ben-zyl-6-deoxy-6-fluoro-_α_-D-glucopyranosyl)-(1→4)-2,3,6-tri-_O_-benzyl-_α_-D-galacto-pyranoside (48)

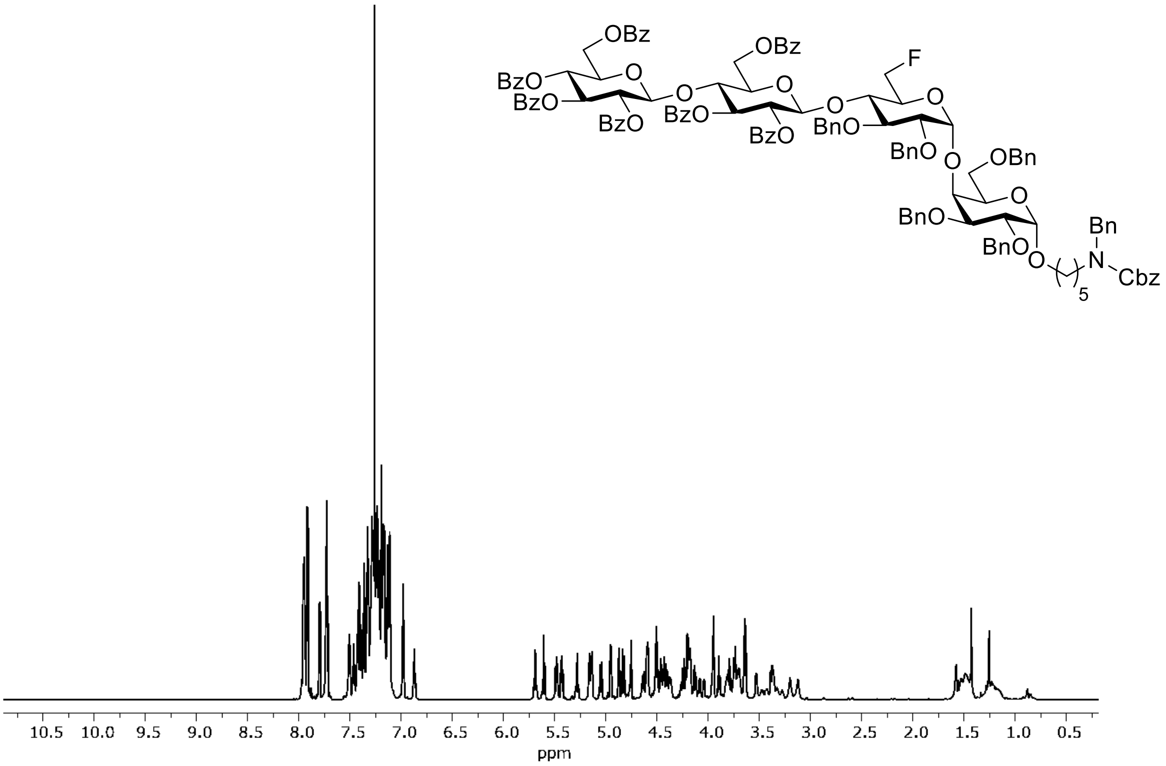

^{1}H NMR (800 MHz, $CDCl_3$) of **48**.

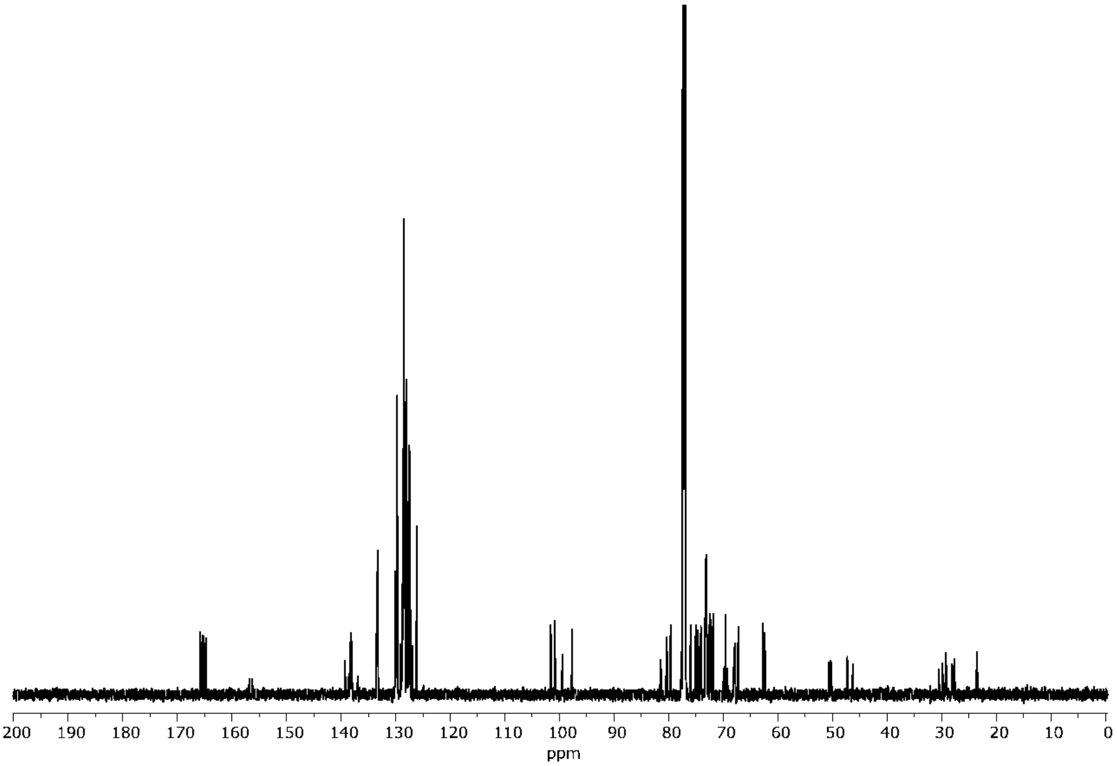

^{13}C NMR (200 MHz, $CDCl_3$) of **48**.

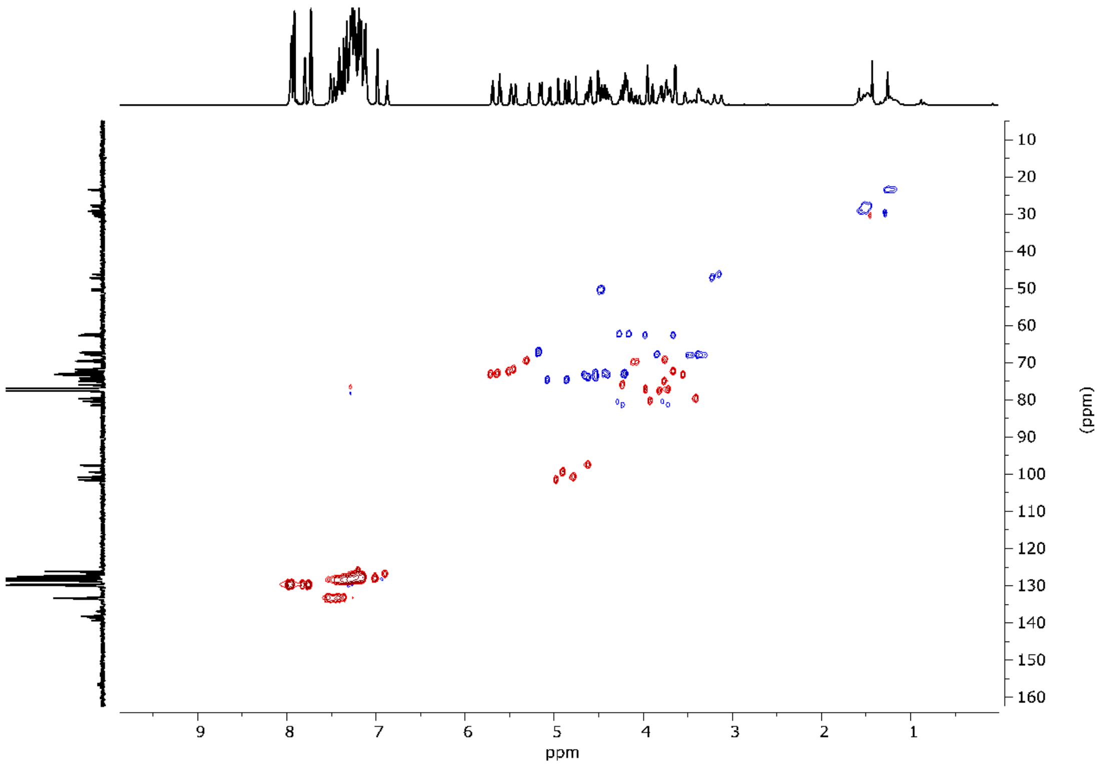

HSQC (CDCl3) of **48**.

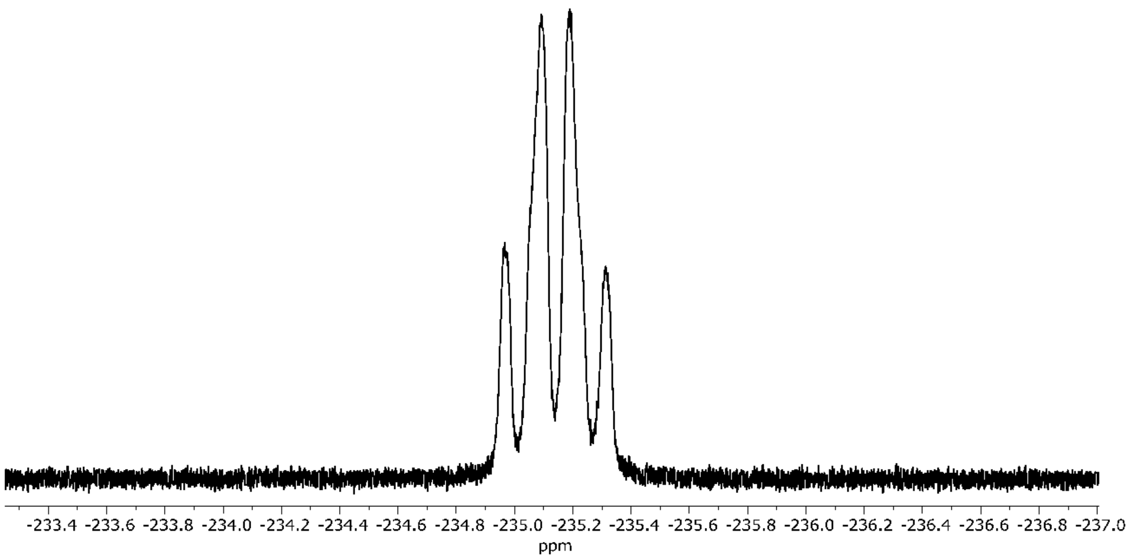

^{19}F NMR (377 MHz, CDCl3) of **48.**

5-Aminopentyl-(β-D-glucopyranosyl)-(1→4)-(β-D-glucopyranosyl)-(1→4)-(6-deoxy-6-fluoro-α-D-gluco-pryranosyl)-(1→4)-α-D-galactopyranoside (8)

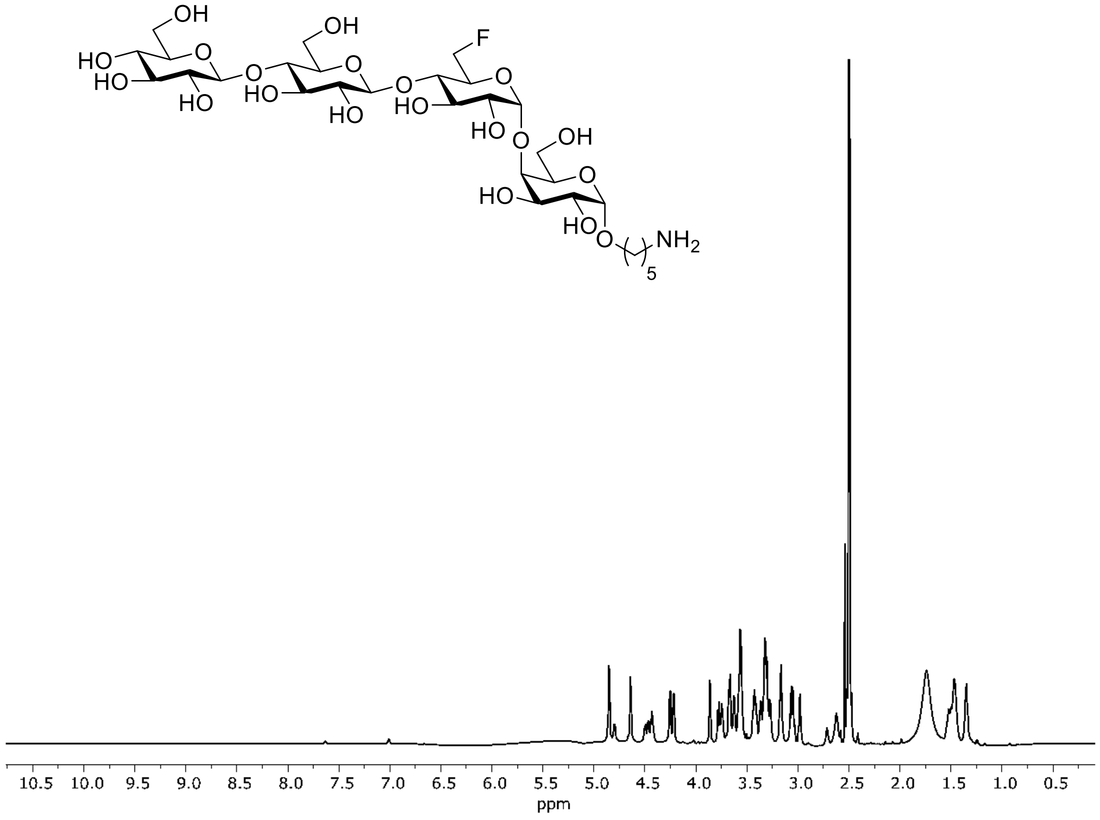

^{1}H NMR (800 MHz, DMSO-d$_6$) of **8**.

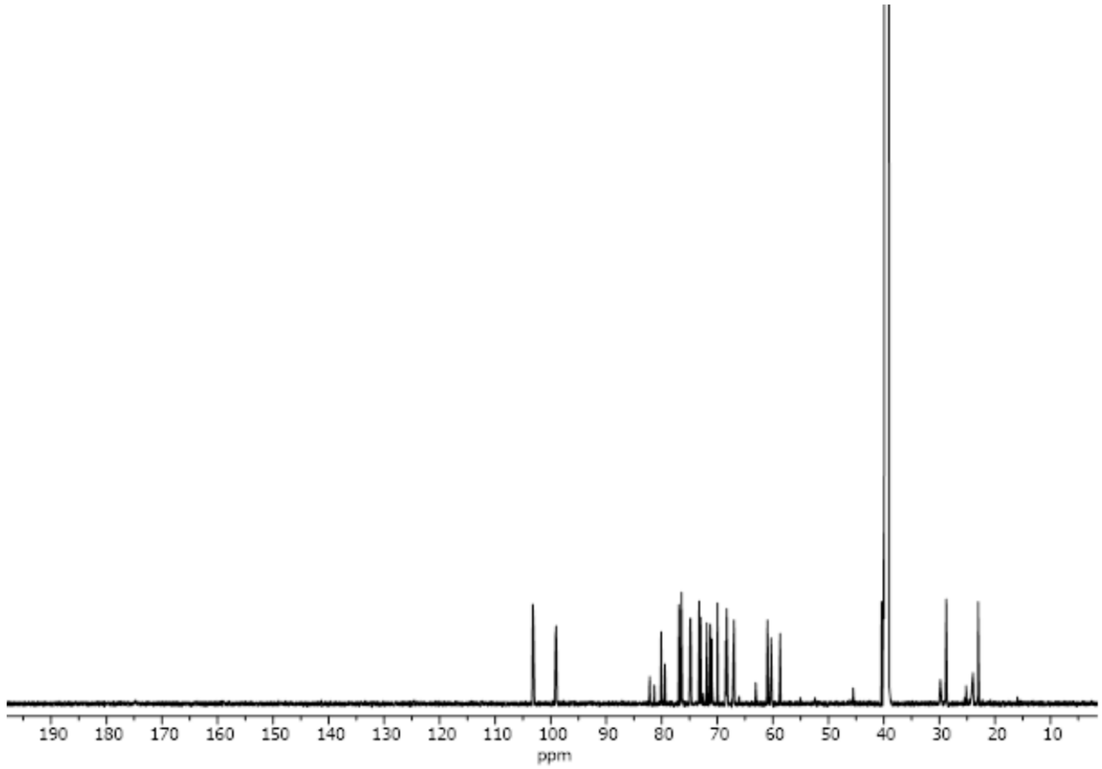

^{13}C NMR (200 MHz, DMSO-d$_6$) of **8**.

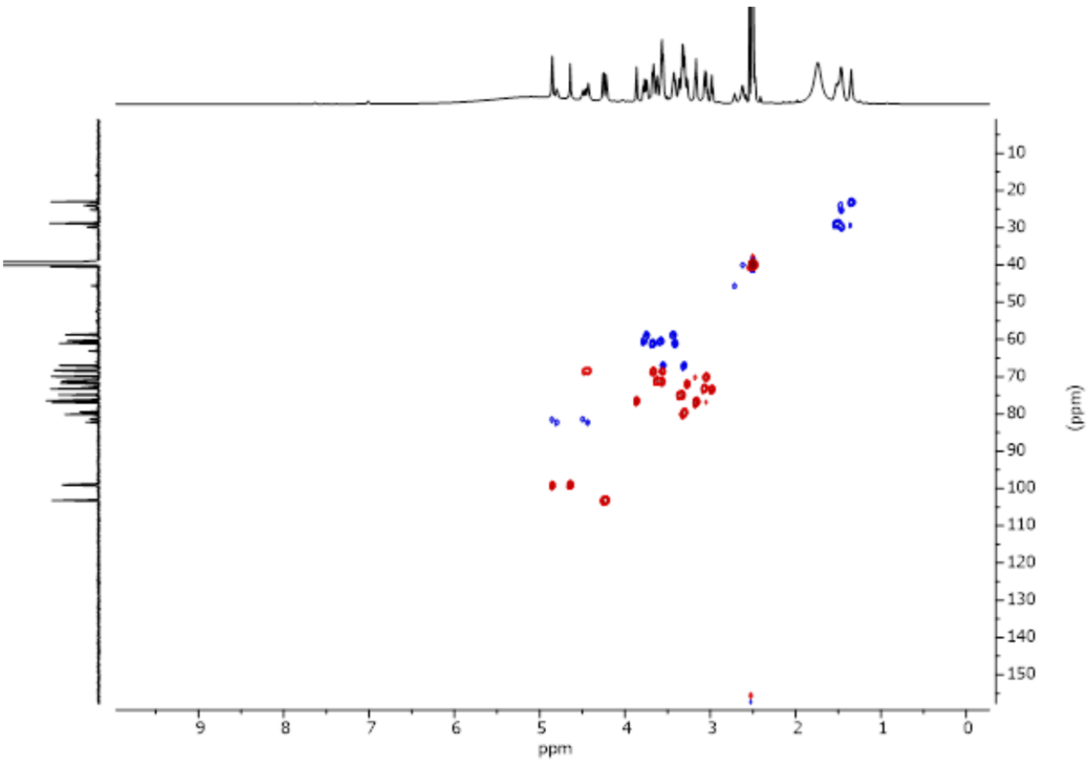

HSQC (DMSO-d$_6$) of **8**.

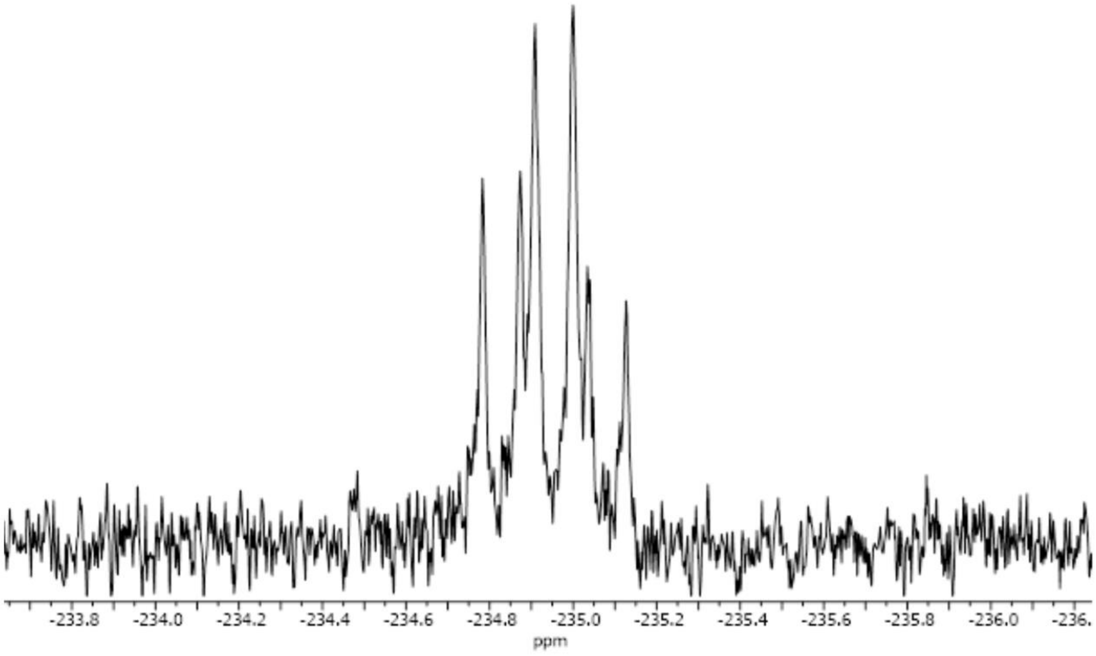

^{19}F NMR (377 MHz, DMSO-d$_6$) of **8**.
